# Coupling Suspect and Nontarget Screening with Mass Balance Modeling to Characterize Organic Micropollutants in the Onondaga Lake–Three Rivers System

**DOI:** 10.1021/acs.est.1c04699

**Published:** 2021-11-03

**Authors:** Shiru Wang, MaryGail Perkins, David A. Matthews, Teng Zeng

**Affiliations:** † Department of Civil and Environmental Engineering, Syracuse University, 151 Link Hall, Syracuse, New York 13244, United States; ‡ Upstate Freshwater Institute, 224 Midler Park Drive, Syracuse, New York 13206, United States

**Keywords:** high-resolution mass spectrometry, wastewater-derived, mixed-source, AQUASIM, photolysis

## Abstract

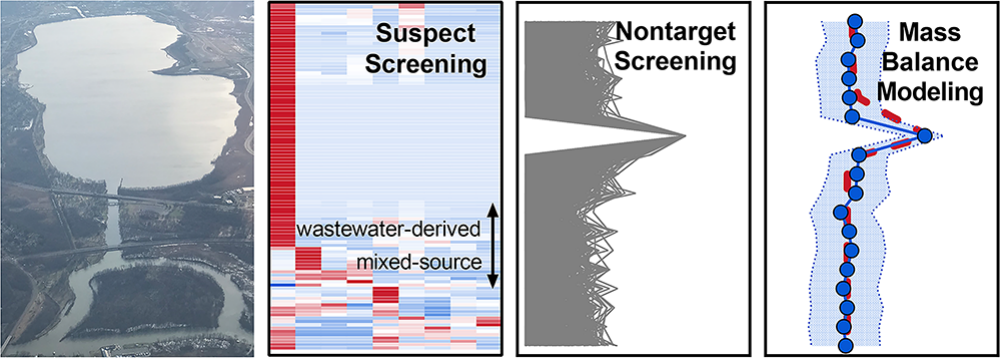

Characterizing the occurrence, sources, and fate of organic micropollutants (OMPs) in lake–river systems serves as an important foundation for constraining the potential impacts of OMPs on the ecosystem functions of these critical landscape features. In this work, we combined suspect and nontarget screening with mass balance modeling to investigate OMP contamination in the Onondaga Lake–Three Rivers system of New York. Suspect and nontarget screening enabled by liquid chromatography–high-resolution mass spectrometry led to the confirmation and quantification of 105 OMPs in water samples collected throughout the lake–river system, which were grouped by their concentration patterns into wastewater-derived and mixed-source clusters via hierarchical cluster analysis. Four of these OMPs (i.e., galaxolidone, diphenylphosphinic acid, *N*-butylbenzenesulfonamide, and triisopropanolamine) were prioritized and identified by nontarget screening based on their characteristic vertical distribution patterns during thermal stratification in Onondaga Lake. Mass balance modeling performed using the concentration and discharge data highlighted the export of OMPs from Onondaga Lake to the Three Rivers as a major contributor to the OMP budget in this lake–river system. Overall, this work demonstrated the utility of an integrated screening and modeling framework that can be adapted for OMP characterization, fate assessment, and load apportionment in similar surface water systems.

## Abstract 2

Organic micropollutants in a lake−river system of significance in the U.S. water pollution history are characterized by an integrated screening and modeling approach.

## Introduction

Water quality trends in aquatic systems often serve as sensitive indicators of environmental change.^1^ One such indicator is the widespread occurrence of organic micropollutants (OMPs) due to the increasing production and use of synthetic organic substances in the domestic, agricultural, and industrial sectors.^2^ OMPs comprise a broad suite of organic chemicals and their transformation products (TPs) that are not traditionally targeted by pollution reduction initiatives and environmental regulation.^3,4^ Closing the gaps in the environmental risk assessment of OMPs requires the joint application of high-resolution mass spectrometry (HRMS) and bioanalytical tools to streamline their identification and to quantify their mixture effects.^5^ HRMS-based suspect and nontarget screening have proven powerful for wide-scope and in-depth investigations of OMP contamination in natural and engineered environments.^6^ Suspect screening searches HRMS data against custom-curated compound lists to focus analytical efforts and exposure assessment on OMPs that are likely to occur in the systems of interest.^7−11^ Nontarget screening exploits the richness of HRMS data to achieve a more comprehensive compound coverage, thereby opening up new opportunities for the retrospective and real-time analyses, source tracking, and effect-directed annotation of so far unknown OMPs.^12−18^ Coupling suspect and nontarget screening therefore serves as a rational approach for prioritizing OMPs that warrant further research to support management plans and regulatory monitoring.

Establishing the occurrence patterns, sources, and fate of OMPs has long been the focal point of research as it constitutes a logical first step toward developing adaptive mitigation measures. For example, substantial efforts have leveraged HRMS to conduct OMP screening, load estimation, and biological effect prioritization in regionally important rivers and lakes.^19−24^ Comparatively fewer studies, however, have applied HRMS to guide OMP characterization in lake–river systems,^19,25^ which are a prevalent landscape feature across the Great Lakes Basin^26^ and in many regions of the world^27^ and influence broad-scale processes ranging from nutrient and carbon cycling^28,29^ to shifts in fish community dynamics.^30,31^ Many of the severely degraded lake–river systems in the U.S. receive wastewater discharge and runoff from adjoining developed areas with dense populations.^32−34^ One prominent example is the Onondaga Lake–Three Rivers system in New York,^34−36^ which supports ∼1.3 million people and is among the most extensively studied hydrologic networks with respect to water quality monitoring and modeling.^34^ Historically, Onondaga Lake was the most polluted lake in the U.S. due to significant inputs of domestic and industrial wastes (e.g., mercury).^37^ Over recent decades, the lake has undergone recovery as a result of phased remediation actions, but effluent discharged from a large regional wastewater treatment plant (WWTP) serving the Syracuse metropolitan area still contributes ∼20–30% of the annual hydrologic budget of Onondaga Lake.^35^ Such a contribution of wastewater effluent to the inflow of Onondaga Lake is among the highest for an inland lake of its size.^35,38^ Given the known wastewater discharge and diffuse runoff inputs from the adjoining urban corridor,^34^ OMPs likely constitute an overlooked stressor in the Onondaga Lake–Three Rivers system; however, knowledge regarding the types and concentrations of OMPs in this system is lacking.

The primary goal of this work was to apply an integrated screening and modeling approach to characterize OMP contamination in the Onondaga Lake–Three Rivers system. Our specific objectives were (i) to perform suspect screening of OMPs in water samples collected from the Onondaga Lake–Three Rivers system for source-related clustering of OMPs; (ii) to explore nontarget screening for prioritization of unknown OMPs exhibiting characteristic vertical distribution patterns during thermal stratification in Onondaga Lake; and (iii) to apply mass balance modeling for fate assessment and load apportionment of OMPs in the lake–river system.

## Materials and Methods

Chemical sources and reagent preparation are described in the Supporting Information. OMP reference standards and isotope-labeled internal standards are listed in Table S1.

### Field Sampling

**Figure 1:**
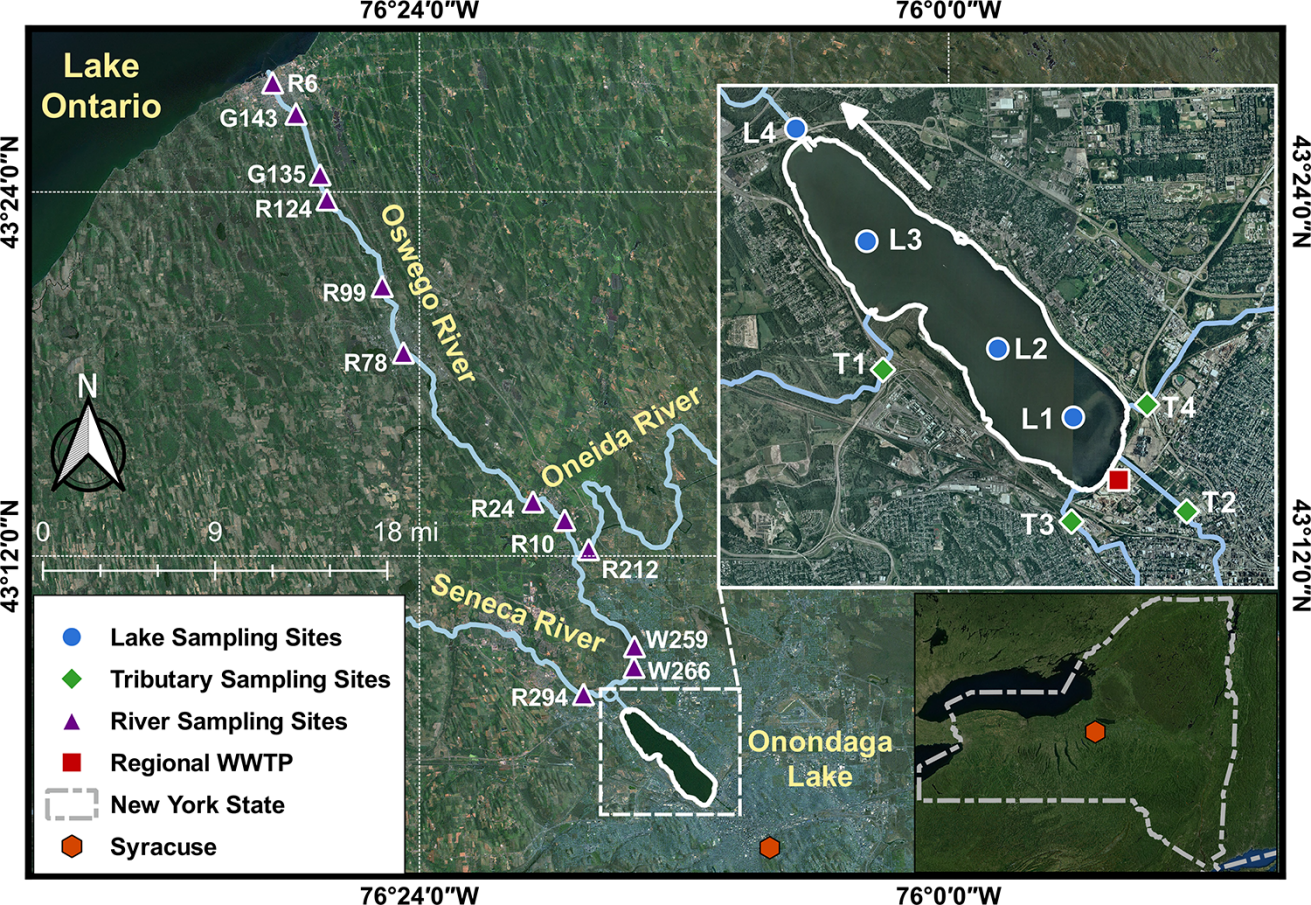
Map of the sampling sites in the Onondaga Lake–Three Rivers system. The blue circles represent the sampling sites on Onondaga Lake [i.e., L1 (south end), L2 (south deep), L3 (north deep), and L4 (outlet)]. Site L2 is considered representative of lake-wide water quality conditions and has served as a long-term water quality monitoring site for Onondaga Lake.^35^ The green diamonds represent the sampling sites near the mouths of the four major lake tributaries [i.e., T1 (Ninemile Creek), T2 (Onondaga Creek), T3 (Harbor Brook), and T4 (Ley Creek)]. The purple triangles represent the sampling sites on the Seneca–Oneida–Oswego Rivers. The red square represents the regional WWTP serving the Syracuse metropolitan area. The white arrow indicates that Onondaga Lake flows from southeast to northwest and ultimately discharges to the Seneca River. Sampling site coordinates and sampling dates are summarized in Table S2. Satellite Image Source: Esri, Maxar, GeoEye, Earthstar Geographics, CNES/Airbus DS, USDA, USGS, AeroGRID, IGN, and the GIS User Community.

Over the study period (i.e., June to October 2017), a total of 143 grab water samples (excluding field blanks) were collected from 21 sites (Table S2) in the Onondaga Lake–Three Rivers system (Figure 1) following trace-level protocols.^39^ Onondaga Lake is a dimictic, mesotrophic, and rapid flushing (typically four times per year on a completely mixed basis^35^) lake located in the metropolitan area of Syracuse, New York, and has a longitudinal axis measuring 7.6 km, a surface area of 12 km^2^, and a mean depth of 10.9 m.^37^ Onondaga Lake discharges through a single outlet at its northern end to the Seneca River that flows northerly and joins the Oneida River to form the Oswego River, which constitutes the largest river network (i.e., the Three Rivers) that drains into Lake Ontario.^34^ Eight batches of samples were collected from four sites along the longitudinal axis of Onondaga Lake and near the mouths of its four major tributaries (i.e., Ninemile Creek, Onondaga Creek, Harbor Brook, and Ley Creek). Together, these four tributaries and a regional WWTP (with an average treatment capacity of 84 million gallons per day) comprise the major hydrologic inputs to the lake. Given that Onondaga Lake is thermally stratified between mid-May and mid-October,^40^ two additional sets of vertical profile samples were collected at 1 m depth intervals from a long-term monitoring site on the lake in July and October to examine the vertical distribution patterns of OMPs. Two batches of samples were also collected from the midchannel sites along the Three Rivers in July and October to capture high and low discharge conditions. Three sites were sampled on the Seneca River, with one located upstream of the Onondaga Lake outlet and two others located downstream of the lake outlet but upstream of the Seneca–Oneida confluence. One site was sampled near the Oneida River mouth. Eight additional sites were sampled along the Oswego River downstream of the Seneca–Oneida confluence prior to its entry into Lake Ontario near the Oswego River mouth. Eight sampling sites in the Onondaga Lake–Three Rivers system were collocated with the U.S. Geological Survey gauge stations with continuous flow monitoring. Lastly, eight effluent samples were collected from the regional WWTP outfall on the same dates of lake sampling. Samples were transported to Syracuse University on the same day of collection, analyzed for dissolved organic carbon and optical properties (Tables S3 and S4), and stored under −20 °C until OMP analysis.

### Sample Analysis

Samples were extracted by mixed-mode solid-phase extraction (SPE) and analyzed by liquid chromatography–HRMS (LC–HRMS) as described in our previous work.^41^ Within 24 h of collection, duplicate samples (500 mL each) were adjusted to pH 6.8 ± 0.1 with ammonium acetate and formic acid, spiked with a mixture of isotope-labeled internal standards (200 ng/L each; Table S1), and filtered through precombusted 0.7 μm glass fiber filters. Samples were then passed through preconditioned dual SPE cartridges containing 200 mg of Sepra ZT (Phenomenex), 100 mg of Sepra ZT-SAX (Phenomenex), 100 mg of Sepra ZT-SCX (Phenomenex), and 150 mg of ISOLUTE ENV+ (Biotage) sorbents as the top layer and 200 mg of Enviro-Clean graphitized nonporous carbon (United Chemical Technologies) as the bottom layer. SPE cartridges were dried under ultrahigh-purity N_2_ following extraction, reconnected inversely (with graphitized nonporous carbon as the top layer), and eluted sequentially with 6 mL of methanol/ethyl acetate (50:50 v/v; amended with 2% ammonia), 3 mL of methanol/ethyl acetate (50:50 v/v; amended with 1.7% formic acid), and 2 mL of methanol.^7,42^ Sample extracts were concentrated to 0.1 mL under ultrahigh-purity N_2_, reconstituted with methanol/water (10:90 v/v) to a final volume of 1 mL, and transferred to amber autosampler vials for LC–HRMS analysis. Finally, sample extracts were batched by sampling events and analyzed by a Dionex UltiMate 3000 high-performance liquid chromatograph interfaced with a Thermo Scientific LTQ XL hybrid ion trap–Orbitrap high-resolution mass spectrometer under optimized instrument settings (Table S5). For chromatographic separation, 20 μL of SPE extracts were injected onto a Hypersil GOLD C18 analytical column (100 × 2.1 mm, 1.9 μm; preceded with a 10 × 2.1 mm guard cartridge) running water and methanol (both acidified with 0.1% v/v formic acid) as the mobile phases at a flow rate of 200 μL/min and a column temperature of 35 °C. For mass spectrometric analysis, full scan mass spectra were acquired from 100 to 1000 Da with a mass resolution of 60 000 at *m*/*z* 400 using both positive and negative electrospray ionization in separate runs. Full scan-triggered data-dependent tandem mass (dd-MS2) spectra were also acquired (upon reinjection of the sample extracts) with a mass resolution of 7500 at *m*/*z* 400 using higher energy collision-induced dissociation across stepped collision energies (i.e., 30, 45, and 60%) while maintaining a full scan mass resolution of 30 000 at *m*/*z* 400. Field blanks (i.e., ultrapure water taken to the field and poured into sampling bottles) were extracted and analyzed with each batch of samples to check for unintended contamination during sample collection and transport.

### Suspect and Nontarget Screening

Suspect screening was conducted in *TraceFinder 4.1* (Thermo Scientific) using a custom suspect database containing compound-specific information for 3308 OMPs, including 2135 pharmaceuticals, pesticides, personal care products, and household and industrial chemicals as well as 1173 TPs.^41^ Full scan mass spectra were processed by *TraceFinder* using predefined peak detection and isotopic pattern parameters (Table S6) for suspect database matching. Only peaks fulfilling a mass accuracy tolerance of 5 ppm and an isotopic pattern fit threshold of >50% were selected for dd-MS2 spectra acquisition. Nontarget screening was conducted using *Compound Discoverer 3.1* (Thermo Scientific) via sorting the vertical distribution patterns of OMPs during summer thermal stratification in Onondaga Lake. Full scan mass spectra collected for July vertical profile samples were first imported into *Compound Discoverer* to enable automated retention time alignment, peak componentization (i.e., grouping of isotopes, adducts, multicharged ions, and in-source fragments), background subtraction, molecular formula assignment, and intensity normalization.^43^ Hierarchical cluster analysis and peak area ratio analysis were performed to cluster and filter peaks exhibiting at least a 1.2-fold intensity increase in the samples collected at 7 m depth relative to the samples collected at 6 and 8 m depths. Filtered peaks fulfilling the following criteria were selected for dd-MS2 spectra acquisition: (a) presence in all vertical profile samples; (b) peak intensity above 10^5^; (c) reasonable peak width and symmetry;^7^ (d) normalized peak intensity profile showing Pearson’s correlation coefficient of >0.7 with the average normalized vertical concentration profile of wastewater-derived OMPs prioritized by suspect screening; and (e) reasonable molecular formula predicted from the exact mass and the isotopic pattern.^44^ Full scan-triggered dd-MS2 spectra of suspect and nontarget compounds were imported into *Compound Discoverer* for mass spectral library searching via *mzCloud* and *MassBank*.^45,46^ Suspect and nontarget compounds with a spectral match factor of >30 were prioritized for further evaluation against authentic reference standards. Complete details of *Compound Discoverer* node-based workflows (Figures S7 and S8) and node settings (Tables S7 and S8) are provided in Section S4.

Together, suspect and nontarget screening prioritized 385 compounds (Table S9), among which 105 were confirmed by verifying their chromatographic retention times and dd-MS2 spectra against those of the respective reference standards. Target analysis was performed retrospectively to quantify the concentrations of these 105 confirmed OMPs with reference to their isotope-labeled analogues or those with the closest chromatographic retention times (Table S10). On average, the absolute SPE recovery of these 105 OMPs was 93 ± 30%, and the limits of quantification for 85% of these OMPs in Onondaga Lake water were below 25 ng/L. Calibration standards of OMPs (processed by SPE and analyzed by LC–HRMS as compound mixtures) and solvent blanks were run at the beginning of each sample sequence to monitor within-run stability, between-run consistency, and potential cross-contamination, respectively. Complete details of the SPE–LC–HRMS method performance for OMP quantification are provided in Section S4.

### Data Analysis

Following the screening and quantification of OMPs, hierarchical cluster analysis was performed using the *ComplexHeatmap*(47) package in *R 4.0.3* to visualize the clustering patterns of OMPs based on their *z*-score standardized median concentrations in the samples. Partial least-squares regression analysis was performed using *SIMCA 16.0* (Umetrics) with the cumulative concentrations of OMP clusters as the response variables and a suite of 16 water quality parameters and optical properties (Tables S3 and S4) as the predictor variables to rank the predictive power of these variables for OMP occurrence.^43^ Exposure–activity ratios were calculated for OMPs with reliable exposure–effects relation data in the ToxCast and Tox21 high-throughput screening database^48−50^ using the *toxEval* package^51^ in *R* to provide a screening-level assessment of the potential for *in vitro* molecular effects associated with OMPs under the mean exposure scenario. Other statistical analyses were performed using *GraphPad Prism 8.4*.

Mass balance modeling was performed to simulate the vertical concentration profiles of 54 OMPs in Onondaga Lake using *AQUASIM 2.1g*.^52^ Onondaga Lake was configured by the lake compartment module in *AQUASIM* as a one-dimensional system with inputs from the four lake tributaries and the regional WWTP, vertical mixing, and export by flushing via the lake outlet. Such analyses assumed uniform horizontal mixing and minimal impacts of other elimination processes on the fate of OMPs.^53−55^ Vertical mixing in the lake was modeled by the turbulent diffusion coefficients derived from the vertical temperature profiles.^56^ For each OMP, the vertical concentration profiles simulated by the one-dimensional flushing model were evaluated against the measured profiles by two quantitative metrics: the percent bias (PBIAS, which quantifies the average tendency of the simulated data to over- or underestimate the measured data) and the Nash–Sutcliffe efficiency (NSE, which estimates the correspondence between the simulated and measured data).^57,58^ Onondaga Lake was also modeled with photolysis in the epilimnion as an additional elimination mechanism for OMPs.^53−55^ For nine selected OMPs (i.e., carbamazepine, fluconazole, gabapentin, lamotrigine, lidocaine, sulfamethoxazole, caffeine, atrazine, and metolachlor) known to undergo direct and/or indirect photolysis, simulated sunlight photolysis tests were conducted to estimate the magnitude of photolysis rate constants in Onondaga Lake (Figure S10) for parameterization of the one-dimensional flushing–photolysis model. Further details regarding vertical profile simulations and photolysis tests are provided in Section S5.

Mass balance modeling was also performed to enable load apportionment of 45 OMPs consistently detected in the Onondaga Lake–Three Rivers system. The Three Rivers system was configured by the river section compartment module in *AQUASIM* as a one-dimensional system, with upstream inputs from the Seneca River, Onondaga Lake, and the Oneida River, lateral input along the Oswego River reach, and export by flushing via the Oswego River mouth. Such analyses assumed no additional major flow sources in the lake–river system^34^ and conservative behavior of OMPs during the riverine transport.^59^ For each OMP, the input loads (*L*_input_) contributed by Onondaga Lake and the Three Rivers were calculated and summed for comparison with the output loads (*L*_output_) exported from the lake–river system to Lake Ontario. The fractional load contributions of Onondaga Lake and the Three Rivers to *L*_output_ were further calculated to quantify the relative importance of each hydrologic component to the OMP budget in the system. Further details regarding load apportionment analysis are provided in Section S6.

## Results and Discussion

### Occurrence and Clustering Patterns of OMPs

Overall, suspect and nontarget screening led to the confirmation and quantification of 105 OMPs in the samples collected from the Onondaga Lake–Three Rivers system (Table S11). Out of these 105 OMPs, 101 were prioritized via suspect screening, whereas the remaining four were prioritized via nontarget screening (see Identification of Nontarget OMPs below). Most of these OMPs spanned a concentration range of 5–2500 ng/L and existed as mixtures of pharmaceuticals (e.g., antiepileptics, antihypertensives, antibiotics, and antidepressants), pesticides (e.g., herbicides, fungicides, insecticides, and plant growth regulators), personal care products (including lifestyle chemicals), household and industrial chemicals, as well as their TPs. Notably, the average number of OMPs (i.e., 62) and the median cumulative concentration of OMPs (i.e., 8060 ng/L) measured in Onondaga Lake were several folds higher than those reported for other New York inland lakes with mixed-used watersheds,^41^ confirming Onondaga Lake as a regional hotspot of OMP contamination.

**Figure 2:**
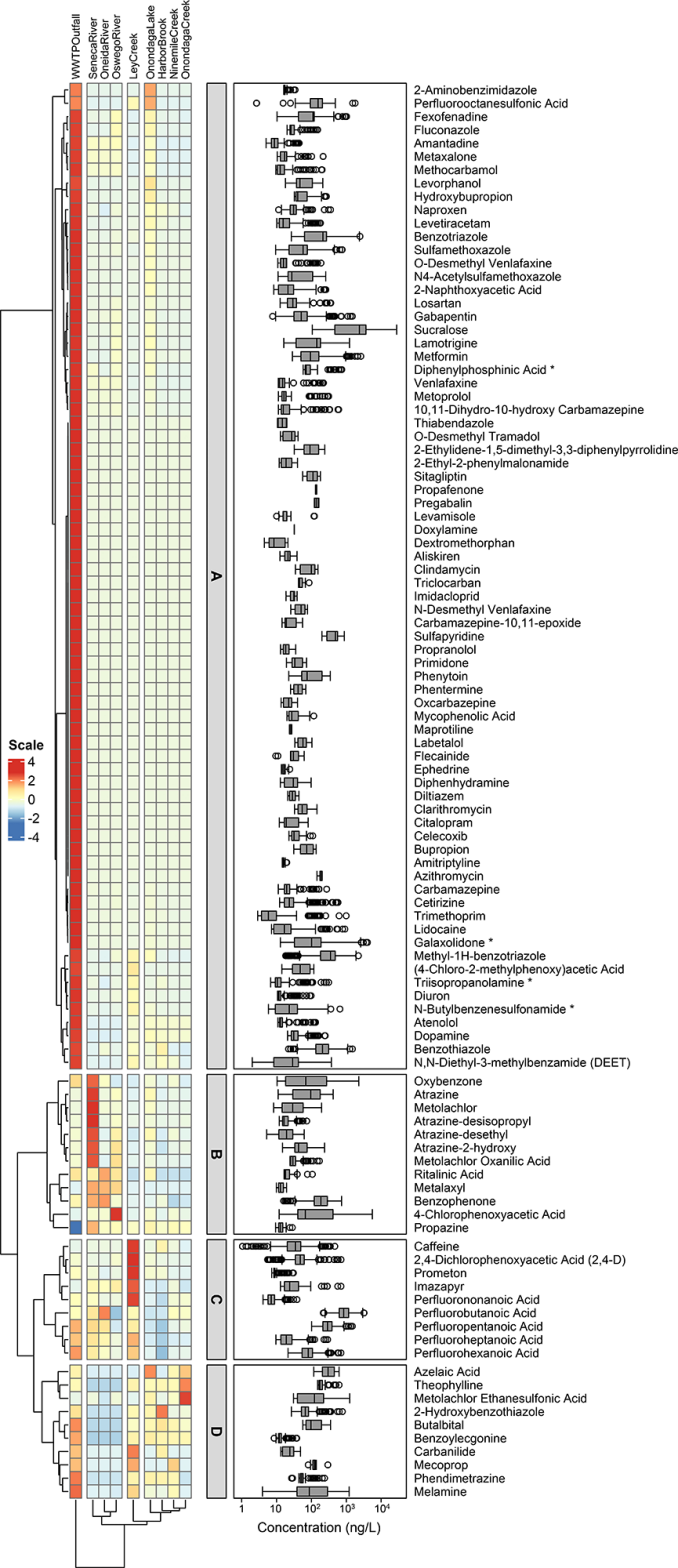
Hierarchical clustering of OMPs by the *z*-score standardized median concentrations of OMPs in the samples based on Euclidean distance with Ward’s method. The color scale (red to blue) measures the detection frequency of OMPs. OMPs are grouped into four clusters (i.e., cluster A, B, C, and D, respectively). Cluster A is designated as the wastewater-derived cluster. Clusters B, C, and D are designated as the mixed-source clusters. The row annotations correspond to the concentration ranges (in the logarithmic scale) of OMPs measured in all samples (*n* = 143). The box extends from the 25th to 75th percentiles. The whiskers extend down to the 25th percentile minus 1.5 times of the interquartile range and up to the 75th percentile plus 1.5 times of the interquartile range. The centerline in each box marks the median. Points plotted beyond the whiskers are outliers. OMPs with an asterisk (“*”) denote those prioritized via nontarget screening and confirmed by authentic reference standards. The column annotations correspond to the major site groups in the Onondaga Lake–Three Rivers system. OMP concentration ranges and detection frequencies are summarized in Table S11.

Hierarchical cluster analysis applied to the *z*-score standardized median concentrations partitioned OMPs into four clusters (Figure 2). Cluster A contains 74 wastewater-derived OMPs that occurred at higher median concentrations in the samples from the WWTP outfall than in the samples from other sites in the Onondaga Lake–Three Rivers system (Tukey’s multiple comparison test *p* = 0.0053–0.0356). Many cluster A OMPs were pharmaceuticals, pharmaceutical TPs, pesticides, personal care products, and industrial additives that have been frequently detected in aquatic systems around the world;^19,20,25,59,60^ however, several were pharmaceuticals (e.g., levamisole, maprotiline, pregabalin, propafenone, and phentermine) that have rarely or never been quantified in U.S. surface waters. Four cluster A OMPs, including lamotrigine, benzotriazole, methyl-1*H*-benzotriazole, and sucralose, occurred in 100% of the samples with median concentrations (i.e., 139–2330 ng/L) far exceeding typical levels measured in New York surface waters.^20,41,61^ Out of the 29 cluster A OMPs with a high detection frequency (i.e., ≥70%), the concentrations of 20 showed strong correlations (Pearson’s *r* = 0.806–0.972) with the cumulative concentration of all OMPs detected in the samples (Figure S11). Quantifying this subset of OMPs, therefore, provides a means to estimate the magnitude of OMP concentrations in the Onondaga Lake–Three Rivers system (Figure S12). Cluster B mainly consists of UV filters as well as herbicides and their TPs that occurred at higher median concentrations in the samples from the Seneca River than in the samples from other sites (Tukey’s multiple comparison test *p* = <0.0001–0.0175). Of the 12 cluster B OMPs, atrazine and metolachlor, both of which were herbicides heavily used in agricultural and urban settings,^62^ and three atrazine TPs (i.e., atrazine-2-hydroxy, atrazine-desethyl, and atrazine-desisopropyl) occurred in 100% of the samples at median concentrations (i.e., 16–104 ng/L) similar to those reported by statewide stream and lake reconnaissance studies in New York,^43,63^ whereas benzophenone and oxybenzone occurred in 95 ± 4% of the samples at elevated median concentrations (i.e., 69–189 ng/L). Cluster C contains caffeine, herbicides (i.e., 2,4-D, imazapyr, and prometon), as well as short- and medium-chain perfluoroalkyl carboxylic acids that occurred at higher median concentrations in the samples from Ley Creek than in the samples from other sites (Tukey’s multiple comparison test *p* = 0.0113–0.0497). Five perfluoroalkyl carboxylic acids (i.e., perfluorobutanoic acid, perfluoropentanoic acid, perfluorohexanoic acid, perfluoroheptanoic acid, and perfluorononanoic acid) co-occurred as a mixture in 100% of the samples at median concentrations (i.e., 7–833 ng/L) that overlapped with the ranges previously documented for three other perfluoroalkyl substances in Onondaga Lake.^64^ Prometon, a herbicide applied predominantly in urban and residential areas,^65^ also occurred in 100% of the samples, albeit a lower median concentration (i.e., 9 ng/L) than imazapyr and 2,4-D (i.e., 24–48 ng/L). Lastly, cluster D contains miscellaneous pharmaceuticals, herbicides, industrial additives, and TPs that occurred at lower median concentrations in the samples from the Three Rivers than in the samples from the WWTP outfall and the lake tributaries (Tukey’s multiple comparison test *p* = 0.0033–0.0459). Most cluster B, C, and D OMPs have also been detected in New York lakes and rivers with wastewater input and/or urban and agricultural influence^20,41,63,66^ and likely originated from mixed sources contributing to the lake tributaries (e.g., Ley Creek) and rivers (e.g., the Seneca River). Such source-related clustering patterns were further supported by partial least-squares regression analysis that ranked fluorescent organic matter components associated with wastewater discharge and urban/agricultural runoff as the most influential predictor variables for the cumulative concentrations of the wastewater-derived and mixed-source OMP clusters (Figure S13). Unlike wastewater-derived OMPs, the concentrations of mixed-source OMPs showed only weak or no statistically significant correlations with the cumulative concentration of all OMPs detected in the samples. Nevertheless, five mixed-source OMPs (i.e., caffeine, 2,4-D, atrazine, metolachlor, and perfluorohexanoic acid) exhibited a median site-specific exposure–activity ratio that exceeded the conservative effects–screening threshold of 0.001^67^ under the mean exposure scenario. Collectively, these five OMPs and two wastewater-derived OMPs (i.e., carbamazepine and diuron) contributed to 95 ± 10% of the site-specific cumulative exposure–activity ratios (Figure S14), highlighting their relevance for exposure assessment and effect modeling. Together, suspect screening and hierarchical cluster analysis established the general occurrence and clustering patterns of OMPs in the Onondaga Lake–Three Rivers system which in turn served to guide subsequent nontarget screening and mass balance modeling efforts.

### Identification of Nontarget OMPs

**Figure 3:**
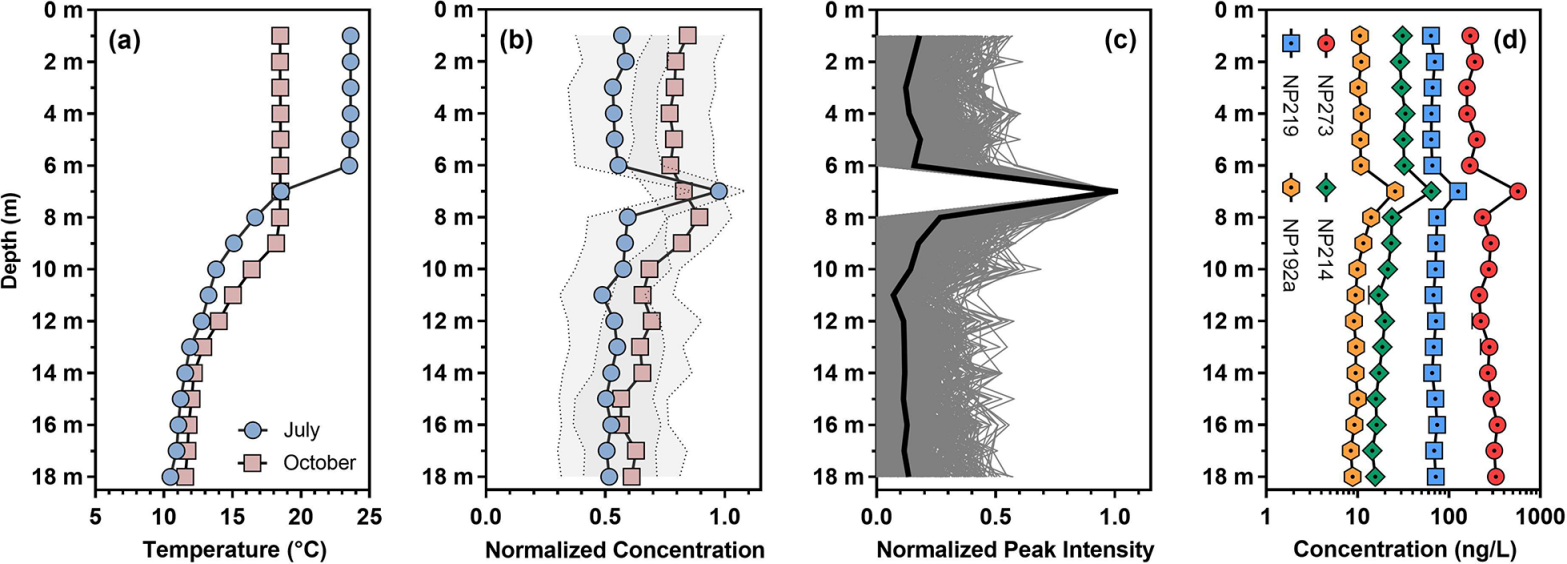
Vertical profiles of temperature, wastewater-derived OMPs, and prioritized nontarget mass spectral features in Onondaga Lake. (a) Vertical profiles of water temperature measured in Onondaga Lake measured by a YSI 6600 multiparameter sonde. (b) Vertical profiles of the mean normalized concentrations of wastewater-derived OMPs confirmed by suspect screening. The grey shade represents the error bands derived from the standard deviation of the normalized concentrations. (c) Vertical profiles of the normalized peak intensities of 589 nontarget mass spectral features in July vertical profile samples. The black solid line represents the mean normalized peak intensities of all 589 nontarget features. (d) Vertical concentration profiles of nontarget compounds NP273 (galaxolidone), NP219 (diphenylphosphinic acid), NP214 (*N*-butylbenzenesulfonamide), and NP192a (triisopropanolamine) in July vertical profile samples. Error bars represent the standard deviation of duplicate measurements; where absent, bars fall within symbols. Further information on level 1 confirmation of NP273, NP219, NP214, and NP192a is provided in Table S12 and Figure S15.

Onondaga Lake was thermally stratified during the sampling period (Figure 3a),^40^ so presumably the vertical distribution patterns of OMPs in the water column were influenced by the buoyancy of inflows and the lake’s thermal stratification regime. For example, the majority of wastewater-derived OMPs (e.g., pharmaceuticals) formed distinct concentration spikes in the metalimnion (i.e., at 7 m depth) when the lake was strongly stratified in July 2017; however, such concentration spikes diminished as the lake became weakly stratified in October 2017 (Figure 3b). Considering the plunging inflow phenomenon in Onondaga Lake,^68−71^ the concentration spikes observed in July likely developed as a result of the plunging of negatively buoyant inflows (i.e., cooler and denser WWTP effluent and tributary waters relative to the epilimnetic lake water; see temperature data in Table S3) into the metalimnion. Hypothetically, the depth-resolved concentration profiles of OMPs confirmed by suspect screening could be leveraged to filter and prioritize nontarget mass spectral features for structural elucidation. To test this hypothesis, mass spectral features with normalized peak intensity profiles that showed a high degree of shape similarity (Pearson’s *r* > 0.7) with the normalized vertical concentration profiles of wastewater-derived OMPs were extracted from July vertical profile samples, resulting in a total of 589 unique nontarget features that spread across a wide range of molecular weights (i.e., *m*/*z* from 115.0750 to 821.8796) and polarities (i.e., chromatographic retention times from 1.7 to 27.1 min). On average, the peak intensities of these nontarget features at 7 m depth were 7.4 and 8.1 times higher than those of the same features measured in the epilimnion (i.e., 1–6 m) and hypolimnion (i.e., 8–18 m), respectively (Figure 3c), confirming their vertical profile similarity with OMPs exhibiting concentration spikes.

Out of the 589 nontarget features, 62 were identified at different levels of confidence^72^ (Table S12), including four at level 1 (in addition to those confirmed via suspect screening), nine at level 3 (tentative candidates), and 49 at level 4 (unequivocal molecular formulas). Galaxolidone, diphenylphosphinic acid, *N*-butylbenzenesulfonamide, and triisopropanolamine were confirmed at level 1 by their respective reference standards (Figure S15). Like other wastewater-derived OMPs, these four compounds also featured a July concentration spike in the metalimnion (Figure 3d). Three of them (except galaxolidone) have been profiled by the ToxCast and Tox21 high-throughput screening programs. Galaxolidone is an oxidation TP of galaxolide commonly added in personal care products^73^ and was first identified as an aquatic contaminant in 1999.^74^ Galaxolidone occurred in 97% of the samples from the lake–river system at a median concentration of 102 ng/L, which fell within the concentration ranges (i.e., <10–300 ng/L) measured in wastewater-receiving rivers.^75,76^ Diphenylphosphinic acid, a flame retardant additive^77^ and a TP of triphenylphosphine oxide,^78^ occurred in 76% of the samples at a median concentration of 71 ng/L. Diphenylphosphinic acid has recently been identified by nontarget screening as a persistent and mobile contaminant in anaerobic bank filtrates extracted from a river bank filtration system in the lower Rhine,^79^ but its concentration in U.S. surface waters has not been reported. *N*-Butylbenzenesulfonamide is a high production volume sulfonamide plasticizer used in the production of industrial polymers and the synthesis of sulfonyl carbamate herbicides^80^ and was first discovered in water samples from a wastewater-impacted segment of the Delaware River in the U.S.^81^*N*-Butylbenzenesulfonamide occurred in 97% of the samples at a concentration of 6–659 ng/L, which overlapped with the ranges reported for wastewater effluent and surface waters.^82−84^ Triisopropanolamine is a tertiary alkanolamine used in various industrial applications and occurred in 99% of the samples at a median concentration of 10 ng/L. Triisopropanolamine was first detected at 6–21 ng/L in wastewater effluents,^10^ but its occurrence in riverine and estuarine waters has also been highlighted in recent nontarget screening studies.^84,85^ Other nontarget OMPs tentatively identified at level 3 (Figure S16) were likely industrial additives and synthetic cathinones following the inspection of the top-ranked structures in *mzCloud*, but authentic reference standards are required for further confirmation of these compounds. Overall, nontarget screening based on the measure of vertical profile similarity during thermal stratification in Onondaga Lake complemented suspect screening and enabled the discovery of additional OMPs that were ubiquitously present in the Onondaga Lake–Three Rivers system.

### Mass Balance Modeling of OMPs

**Figure 4:**
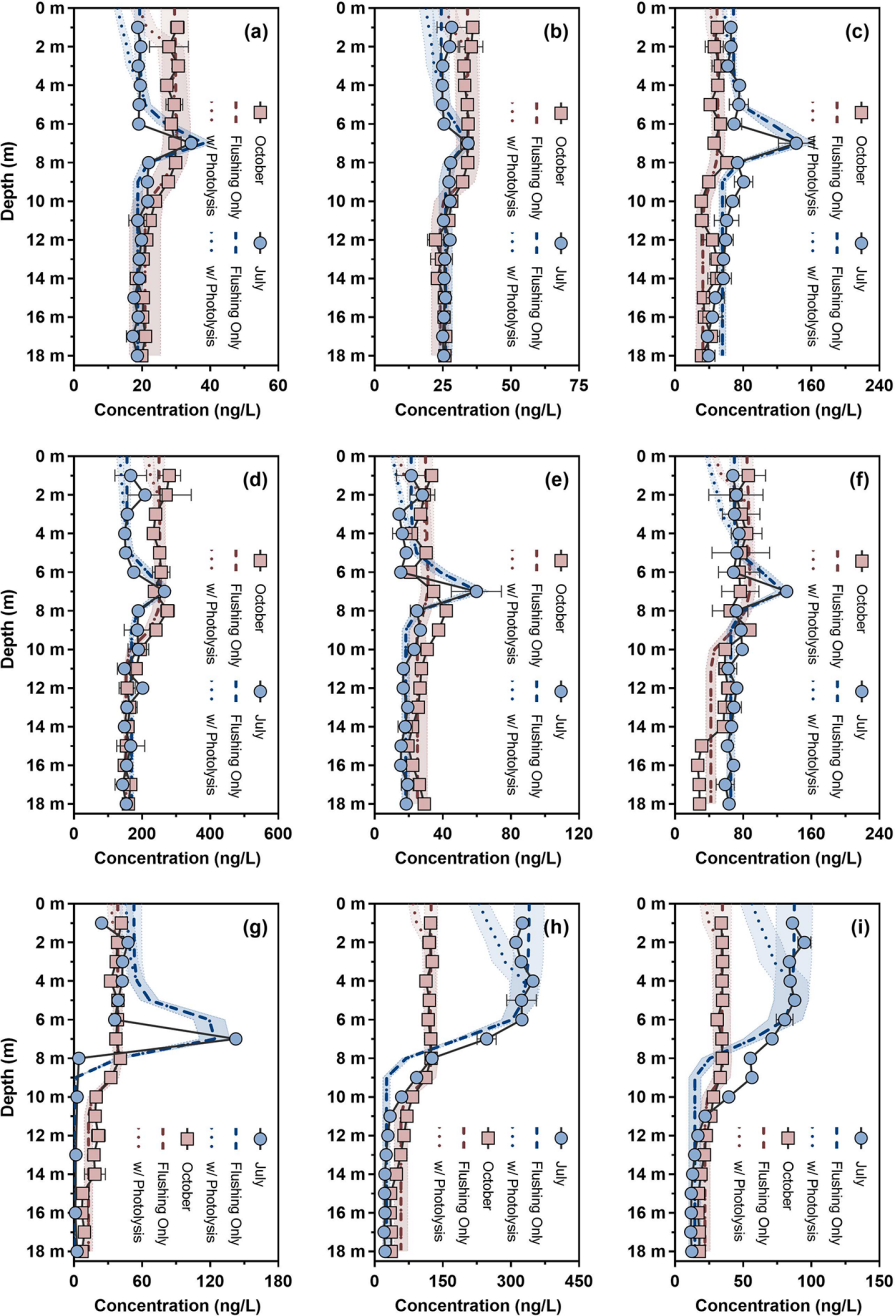
Comparison of the measured and *AQUASIM*-simulated vertical concentration profiles of nine selected OMPs in Onondaga Lake. (a) Carbamazepine. (b) Fluconazole. (c) Gabapentin. (d) Lamotrigine. (e) Lidocaine. (f) Sulfamethoxazole. (g) Caffeine. (h) Atrazine. (i) Metolachlor. Solid lines with symbols represent the measured vertical concentration profiles. Error bars represent the standard deviation of duplicate measurements; where absent, bars fall within symbols. Dashed lines and error bands represent the vertical concentration profiles simulated by the one-dimensional flushing model (“flushing only”) and the standard deviation of model simulations, respectively. Dotted lines and error bands represent the vertical concentration profiles simulated by the one-dimensional flushing–photolysis model (“w/photolysis”) and the standard deviation of model simulations, respectively. The measured and *AQUASIM*-simulated vertical concentration profiles of other 45 OMPs are provided in Figures S17 and S18. Further details regarding vertical profile simulations and photolysis tests are provided in Section S5.

Mass balance modeling was first implemented in *AQUASIM* to evaluate the fate of OMPs in Onondaga Lake given its relatively short hydraulic residence time.^86^ Onondaga Lake was strongly stratified in July 2017, during which the negatively buoyant inflows plunged to the metalimnion as interflows to varying extents,^68−71^ and the fraction of each inflow entering the metalimnion varied from 10 to 40% according to the specific conductance data measured *in situ* by rapid profiling instrumentation.^34^ On the other hand, the metalimnion descended to lower depths as the lake became weakly stratified in October 2017, during which 100% of the inflows entered the well-mixed epilimnion. Taking the inflow plunging and vertical mixing into account, a one-dimensional model assuming flushing via the lake outlet as the sole export mechanism yielded reasonable simulations of OMP concentration profiles in the water column of Onondaga Lake (Figure 4 and additional profiles in Figures S17 and S18). For 54 OMPs with a detection frequency of >80% in the vertical profile samples, the mean PBIAS value was typically within ±5% and the mean NSE value ranged from 0.01 to 0.92 with a median of 0.40 (Figure S19). On average, the compound-specific NSE values were not statistically different for wastewater-derived and mixed-source OMPs (Mann–Whitney *U* test *p* = 0.9960), confirming the consistency of model performance across different OMP clusters. Furthermore, the mean PBIAS values for either wastewater-derived or mixed-source OMPs were not statistically different from zero (Tukey’s multiple comparison test *p* = 0.5242 to >0.9999), although the *AQUASIM*-simulated vertical concentration profiles of a few OMPs (e.g., caffeine) exhibited negative or positive PBIAS values beyond ±5% due to the overestimation or underestimation of their epilimnetic concentrations.

Hypothetically, abiotic and/or biotic transformations of OMPs would lead to a negative PBIAS value, whereas unaccounted inputs of OMPs from the lake watershed would introduce a positive PBIAS value for the simulated profiles. For instance, previous studies have incorporated photolysis as an additional mechanism to more accurately predict the degradation of biocides and pharmaceuticals in the epilimnion of Lake Greifensee, Switzerland.^53−55^ To assess the role of photolysis in Onondaga Lake, the depth-dependent photolysis rate constants (Figure S10) of six representative wastewater-derived (i.e., carbamazepine, fluconazole, gabapentin, lamotrigine, lidocaine, and sulfamethoxazole) and three mixed-source OMPs (i.e., caffeine, atrazine, and metolachlor) were calculated for parameterizing a one-dimensional flushing–photolysis model. Of these nine OMPs, lamotrigine underwent direct photolysis, whereas atrazine and metolachlor primarily underwent indirect photolysis in Onondaga Lake water. Other six OMPs underwent direct and indirect photolysis to varying degrees. Nevertheless, the flushing–photolysis model did not significantly reduce the PBIAS or improve the NSE of simulated profiles compared to the flushing model (Mann–Whitney *U* test *p* = 0.1163). For example, changes in the PBIAS and NSE values for the simulated vertical concentration profiles of caffeine were <3% after incorporating the photolysis component. Collectively, these simulation results supported the fact that flushing sustained the export of OMPs from Onondaga Lake to the Three Rivers, whereas photolysis exerted only a minor influence on the fate of OMPs in the epilimnion. Transformation (e.g., biodegradation) or partitioning processes other than photolysis might serve as additional first-order controls for the persistence of OMPs, provided that their rate constants were on a similar order of magnitude to the lake flushing rate constant (e.g., 0.011 d^–1^). Complementary field measurements and laboratory tests are, however, needed to refine the model parameterization.

**Figure 5:**
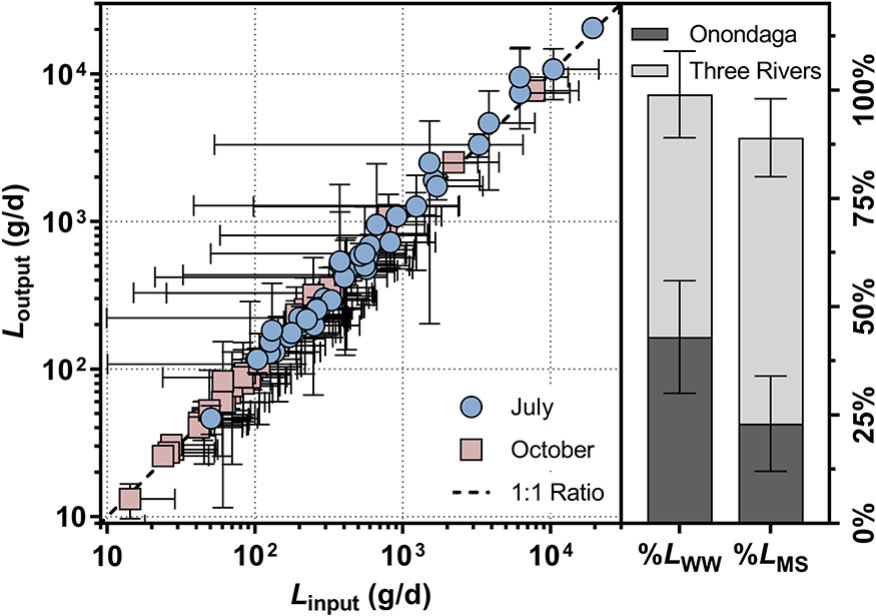
Comparison of the input (*L*_input_, the sum of input loads contributed by Onondaga Lake, the Seneca River, the Oneida River, and the Oswego River reach) and output (*L*_output_; the output loads measured at the Oswego River mouth) loads of OMPs (*n* = 45) in the Onondaga Lake–Three Rivers system (left panel) and the average fractional contributions of Onondaga Lake and the Three Rivers to the respective loads of wastewater-derived (% *L*_WW_) and mixed-source (% *L*_MS_) OMPs (right panel). Error bars represent the standard deviation of loads or fractional contributions calculated for July and October 2017. Further details regarding load apportionment analysis are provided in Section S6.

Mass flow calculations were further performed for 45 OMPs with a detection frequency of >80% at gauged sites for load apportionment in the Onondaga Lake–Three Rivers system. *L*_output_ values of these OMPs varied over 3 orders of magnitude (i.e., 13–20 710 g/d), as reported for other wastewater-impacted river systems and lake catchments.^25,59,60,87^*L*_output_ values of wastewater-derived and mixed-source OMPs were not statistically different (Mann–Whitney *U* test, *p* = 0.0783), with sucralose (i.e., 7700–10 770 g/d), perfluorobutanoic acid (i.e., 2520–20 470 g/d), and perfluoropentanoic acid (i.e., 1110–9530 g/d) contributing the highest *L*_output_. However, *L*_output_ values in July 2017 were higher than those in October 2017 for both wastewater-derived and mixed-source OMPs (Mann–Whitney *U* test *p* = 0.0001–0.0009). Regardless, *L*_input_ corresponded well to *L*_output_ (Figure 5) with a mean *L*_input_/*L*_output_ ratio of 0.95 ± 0.09 for 45 OMPs, substantiating that the cumulative OMP loads contributed by Onondaga Lake and the Three Rivers adequately approximated those observed at the Oswego River mouth despite the differences in the fate and transport properties among OMPs. Two wastewater-derived OMPs (i.e., naproxen and sulfamethoxazole; Figure S20) featured a *L*_input_/*L*_output_ ratio of >1.0 (Tukey’s multiple comparison test *p* = 0.0125–0.0157) potentially due to in-stream transformations or loss during the riverine transport.^88−90^ Conversely, the *L*_input_/*L*_output_ ratios for 10 mixed-source OMPs (i.e., ritalinic acid, 2,4-D, atrazine, imazapyr, metolachlor, metolachlor oxanilic acid, perfluoropentanoic acid, perfluorohexanoic acid, perfluoroheptanoic acid, and perfluorononanoic acid) and two wastewater-derived OMPs (i.e., diphenylphosphinic acid and triisopropanolamine) were less than 1.0 (Tukey’s multiple comparison test *p* = <0.0001–0.0381), suggesting additional inputs of these compounds from unidentified localized and/or diffuse sources within the Oswego River basin. On average, Onondaga Lake contributed to 43 ± 13% of *L*_output_ for wastewater-derived OMPs (Figure 5). Considering the relatively low flow contribution of Onondaga Lake to the lake–river system (i.e., 9 ± 3% vs 92 ± 3% of the Three Rivers), it is evident that the lake exerted a disproportionate influence on the loading of wastewater-derived OMPs to Lake Ontario. Furthermore, Onondaga Lake contributed to 23 ± 11% of *L*_output_ for mixed-source OMPs, indicating that the lake also delivered an appreciable amount of OMPs mobilized from its mixed-use watershed to Lake Ontario. Overall, mass balance modeling illustrated that Onondaga Lake not only functioned as an integrator of OMP inputs from wastewater discharge and watershed processes but also exported a comparatively high mass flow of OMPs to the Three Rivers and ultimately Lake Ontario.

### Environmental Implications

This work represents the first study that combines suspect and nontarget screening with mass balance modeling to investigate OMP contamination in a regional lake–river system of importance in the history of U.S. water pollution. Our work established the source-related occurrence patterns of OMPs in the Onondaga Lake–Three Rivers system and prioritized OMP clusters of concern to inform future monitoring efforts and reduction measures at the site-specific or system-wide scale. Our work also contributed to the growing literature on nontarget screening by exploring the application of a prioritization approach that draws on the connection of compound-specific spatial distribution patterns to lake-specific mechanisms (e.g., inflow plunging during thermal stratification). Such connections have long been recognized by studies focusing on the fate and occurrence of OMPs in lakes with external loads entering stratified depths. For example, an earlier study observed a plume phenomenon in Vidy Bay of Lake Geneva, Switzerland, where wastewater effluent discharged into the lake hypolimnion led to locally elevated concentrations of wastewater-derived OMPs beneath the epilimnion during the warmer months.^91^ Similarly, two recent studies also highlighted the differential impacts of thermal stratification on the vertical distribution of OMPs in Lake Tegel, Germany,^92^ and Lake Mälaren, Sweden,^93^ respectively. Follow-up studies on thus far unknown OMPs in these lakes and other stratified aquatic systems may therefore benefit from adapting a prioritization strategy like the one described in this work to guide nontarget screening. Lastly, our study demonstrated the utility of mass balance analyses for fate assessment and load apportionment of OMPs in lake–river systems with well-defined boundary conditions. From a methodological perspective, the modeling approach can be extended to assess the benefits of OMP load reduction when supplemented with information on potential mitigation options for point sources (e.g., WWTP upgrades^94^) and substance consumption patterns within the contributing watersheds.^25^ Furthermore, quantitative metrics such as NSE values determined in this work would facilitate model evaluation in comparable scenarios in which OMP concentration profiles are simulated based on input dynamics.^58^ Future studies on OMPs in lake–river networks should leverage more spatiotemporally resolved field sampling to capture the sources and dynamics of intermittent discharges and local emissions and consider incorporating system-specific attributes (e.g., hydrodynamic processes and meteorological conditions) and field-measured compound properties to improve model robustness.

## Supporting Information Available

Summary of water quality parameters and optical properties; LC–HRMS instrument parameters and screening workflow settings; SPE–LC–HRMS method performance for OMP analysis; mass balance modeling by *AQUASIM*; concentration ranges and detection frequencies of OMPs; partial least-squares regression analysis and exposure–activity ratio calculations; list of OMPs identified via nontarget screening; measured and simulated vertical concentration profiles of OMPs in Onondaga Lake; and load apportionment of OMPs in the Onondaga Lake–Three Rivers system (PDF)
