# Supplementary material for: Coupling Suspect and Nontarget Screening with Mass Balance Modeling to Characterize Organic Micropollutants in the Onondaga Lake–Three Rivers System
Source: Environ Sci Technol. 2021 Nov 3;55(22):15215–26. doi: 10.1021/acs.est.1c04699 (PMC8600663; doi:10.1021/acs.est.1c04699)
Supplement: Supplementary file 1 — es1c04699_si_001.pdf [file es1c04699_si_001.pdf]

Supporting Information for

Coupling Suspect and Nontarget Screening with Mass Balance Modeling to Characterize Organic Micropollutants in the Onondaga Lake-Three Rivers System

Shiru Wang[1], MaryGail Perkins[2], David A. Matthews[2], Teng Zeng[1]*

[1]Department of Civil and Environmental Engineering, Syracuse University, 151 Link Hall, Syracuse, New York 13244, United States

[2]Upstate Freshwater Institute, 224 Midler Park Drive, Syracuse, New York 13206, United States

*Corresponding Author: Teng Zeng: Email: tezeng@syr.edu; Phone: +1-315-443-1099

(Total 78 pages, 5 texts, 12 tables, 20 figures)

Table of Contents

S1. Chemical sources and reagent preparation S3

S2. Sampling site coordinates and sampling dates........ S14

S3. Analysis of water quality parameters and optical properties S15

S4. Screening and quantification of OMPs by SPE-LC-HRMS S30

S5. Mass balance modeling of OMPs in Onondaga Lake........ S49

S6. Mass balance modeling of OMPs in the Onondaga Lake-Three Rivers system........ S54

S7. Concentration ranges and detection frequencies of OMPs S56

S8. Predictors of OMP occurrence........ S61

S9. Exposure-activity ratios for OMPs S62

S10. OMPs identified by nontarget screening........ S63

S11. Measured and simulated vertical concentration profiles of OMPs in Onondaga Lake........ S68

S12. Load apportionment of OMPs in the Onondaga Lake-Three Rivers system S74

References........ S75

S1. Chemical sources and reagent preparation

Chemicals and reagents were used as received without further purification unless otherwise noted. Methanol (MeOH; HPLC and LC-MS grade), acetonitrile (HPLC and LC-MS grade); water (H_2O; HPLC and LC-MS grade), ethyl acetate (LC-MS grade), formic acid solution (FA; ≥99.0%; LC-MS grade), ammonium hydroxide solution (≥25%; LC-MS grade), ammonium acetate (LC-MS grade), boric acid (≥99.5 %), and Pierce LTQ ESI positive ion and negative ion calibration solutions were purchased from Fisher Scientific. *p*-Nitroanisole (PNA; 99+%) and pyridine (pyr; 99+%) were purchased from ACROS Organics. Five SPE sorbents, including Sepra ZT (30 μm polymer, 85 Å), Sepra ZT-SAX (30 μm polymer, 85 Å), Sepra ZT-SCX (30 μm polymer, 85 Å), ISOLUTE ENV+ (90 μm hydroxylated polystyrene-divinyl benzene copolymer, 800 Å), and Enviro-Clean graphitized carbon (non-porous 120/400 mesh), were purchased from Phenomenex, Biotage, and United Chemical Technologies, respectively. Other SPE supplies (e.g., glass vacuum manifolds, large-volume sample transfer tubes, polypropylene cartridges and frits, cartridge adapters, and stopcocks) were purchased from United Chemical Technologies. Unlabeled reference standards and isotope-labeled internal standards (ILIS) were purchased from Sigma-Aldrich, Toronto Research Chemicals, AccuStandard, ACROS Organics, TCI America, Alfa Aesar, Cayman Chemical Company, C/D/N Isotopes, and Cambridge Isotope Laboratories as high-purity substances or concentrated solutions (**Table S1**). Stock solutions were prepared by dissolving or diluting a gravimetrically weighted amount of solid or liquid reference standards into LC-MS grade methanol, acetonitrile, or pH-adjusted water when applicable and stored under -20 °C. Spike solutions and calibration standards were prepared by diluting predetermined volumes of stock solutions into LC-MS grade water. Mobile phases for LC-HRMS analysis were prepared using LC-MS grade water, methanol, and formic acid. Sampling bottles were rinsed 5 times with HPLC grade methanol followed by 5 times with ultrapure water (18.2 MΩ•cm) prior to field sampling.

**Table S1.** List of unlabeled reference standards and isotope-labeled internal standards				
Compound Name	CAS	Supplier	Catalog Number	Category
17α-Estradiol	57-91-0	Toronto Research Chemicals	E887995	PHAR
17β-Estradiol	50-28-2	Toronto Research Chemicals	E888000	PHAR
3,4-Ethylenedioxymethcathinone (3,4-EDMC)	802041-86-7	Cayman Chemical Company	15167	PHAR
3,4-Methylenedioxymethamphetamine (MDMA)	42542-10-9	Sigma-Aldrich	M-013-1ML	PHAR
3,4-Methylenedioxy-N-ethylamphetamine (MDEA)	82801-81-8	Sigma-Aldrich	M-065-1ML	PHAR
4-Methylethcathinone	1225617-18-4	Sigma-Aldrich	M-155-1ML	PHAR
Abacavir	136470-78-5	Toronto Research Chemicals	A104990	PHAR
Acamprosate	77337-76-9	Toronto Research Chemicals	A120000	PHAR
Acebutolol	37517-30-9	Sigma-Aldrich	A3669-1G	PHAR
Acetaminophen	103-90-2	Sigma-Aldrich	A3035-1VL	PHAR
Albuterol	18559-94-9	Toronto Research Chemicals	A514501	PHAR
Aliskiren	173334-57-1	Sigma-Aldrich	SML2077-50MG	PHAR
Allopurinol	315-30-0	Sigma-Aldrich	A8003-5G	PHAR
Amantadine	768-94-5	Sigma-Aldrich	A1260-5G	PHAR
Amitriptyline	50-48-6	Sigma-Aldrich	A-923-1ML	PHAR
Amobarbital	57-43-2	Sigma-Aldrich	A-020-1ML	PHAR
Amphetamine	300-62-9	Sigma-Aldrich	A-007-1ML	PHAR
Androstanolone (5α-Dihydrotestosterone; DHT)	521-18-6	Sigma-Aldrich	D-073-1ML	PHAR
Androstenedione	63-05-8	Sigma-Aldrich	A-075-1ML	PHAR
Antipyrine	60-80-0	Sigma-Aldrich	A5882-25G	PHAR
Artemisinin	63968-64-9	Toronto Research Chemicals	A777500	PHAR
Aspirin	50-78-2	Sigma-Aldrich	A2093-100G	PHAR
Astemizole	68844-77-9	Sigma-Aldrich	A2861-10MG	PHAR
Atazanavir	198904-31-3	Sigma-Aldrich	SML1796-5MG	PHAR
Atenolol	29122-68-7	Toronto Research Chemicals	A790075	PHAR
Atomoxetine	83015-26-3	Sigma-Aldrich	PHR1679-500MG	PHAR
Azelaic Acid	123-99-9	Sigma-Aldrich	95054-100MG	PHAR
Azithromycin	83905-01-5	Sigma-Aldrich	PHR1088-1G	PHAR
Bamethan	3703-79-5	Sigma-Aldrich	B0382-5G	PHAR
Benazepril	86541-75-5	Toronto Research Chemicals	B119750	PHAR
Betamethasone	378-44-9	Sigma-Aldrich	PHR1398-1G	PHAR
Betaxolol	63659-18-7	Sigma-Aldrich	B5683-10MG	PHAR
Bisoprolol	66722-44-9	Toronto Research Chemicals	B510500	PHAR
Bumetanide	28395-03-1	Sigma-Aldrich	B3023-250MG	PHAR
Bupivacaine	38396-39-3	Sigma-Aldrich	PHR1128-1G	PHAR
Buprenorphine	52485-79-7	Sigma-Aldrich	B-044-1ML	PHAR
Bupropion	34911-55-2	Toronto Research Chemicals	B689625	PHAR
Butalbital	77-26-9	Sigma-Aldrich	B-006-1ML	PHAR
Butylone	802575-11-7	Sigma-Aldrich	B-045-1ML	PHAR
Caffeine	58-08-2	Sigma-Aldrich	C0750-5G	PHAR
Carbamazepine	298-46-4	Sigma-Aldrich	C4024-1G	PHAR
Celecoxib	169590-42-5	Sigma-Aldrich	PHR1683-1G	PHAR
Celiprolol	56980-93-9	Sigma-Aldrich	SML2617-5MG	PHAR
Cetirizine	83881-51-0	Toronto Research Chemicals	C281100	PHAR
Chlonixin	17737-65-4	Toronto Research Chemicals	C587140	PHAR
Cimetidine	51481-61-9	Sigma-Aldrich	C4522-5G	PHAR
Ciprofloxacin	85721-33-1	Sigma-Aldrich	PHR1167-1G	PHAR

Table S1. List of unlabeled reference standards and isotope-labeled internal standards (continued)				
Compound Name	CAS	Supplier	Catalog Number	Category
Citalopram	59729-33-8	Toronto Research Chemicals	C505000	PHAR
Clarithromycin	81103-11-9	Sigma-Aldrich	PHR1038-500MG	PHAR
Clenbuterol	37148-27-9	Sigma-Aldrich	C5423-10MG	PHAR
Clindamycin	18323-44-9	Toronto Research Chemicals	C580000	PHAR
Clomiphene	911-45-5	Sigma-Aldrich	C6272-1G	PHAR
Codeine	76-57-3	Sigma-Aldrich	C-006-1ML	PHAR
Cortisone	53-06-5	Sigma-Aldrich	C-130-1ML	PHAR
Cycloheximide	66-81-9	AccuStandard	P-411S	PHAR
Cyclopentolate	512-15-2	Toronto Research Chemicals	C988430	PHAR
Darunavir	206361-99-1	Sigma-Aldrich	SML0937-10MG	PHAR
Desipramine	50-47-5	Sigma-Aldrich	PHR1723-400MG	PHAR
Desomorphine	427-00-9	Sigma-Aldrich	D-083-1ML	PHAR
Desoxycortone (11-Deoxycorticosterone)	64-85-7	Sigma-Aldrich	D-105-1ML	PHAR
Desvenlafaxine (O-Desmethyl Venlafaxine)	93413-62-8	Sigma-Aldrich	V-007-1ML	PHAR
Detomidine	76631-46-4	Toronto Research Chemicals	D297975	PHAR
Dexpanthenol	81-13-0	Sigma-Aldrich	PHR1228-500MG	PHAR
Dextromethorphan	125-71-3	Sigma-Aldrich	D-013-1ML	PHAR
Diazepam	439-14-5	Sigma-Aldrich	D-907-1ML	PHAR
Diclofenac	15307-86-5	Sigma-Aldrich	93484-100MG	PHAR
Dienogest	65928-58-7	Sigma-Aldrich	SML1468-10MG	PHAR
Dihydrocodeine	125-28-0	Sigma-Aldrich	D-019-1ML	PHAR
Dihydromorphine	509-60-4	Sigma-Aldrich	D-033-1ML	PHAR
Dihydrostreptomycin	128-46-1	Sigma-Aldrich	PHR1517-500MG	PHAR
Diltiazem	42399-41-7	Sigma-Aldrich	D-035-1ML	PHAR
Dimethylone (bk-MDDMA)	765231-58-1	Cayman Chemical Company	22879	PHAR
Dinoprostone (Prostaglandin E2)	363-24-6	Toronto Research Chemicals	P838610	PHAR
Diphenhydramine	58-73-1	Sigma-Aldrich	D-015-1ML	PHAR
Dobutamine	34368-04-2	Sigma-Aldrich	D0676-10MG	PHAR
Dopamine	51-61-6	Sigma-Aldrich	H8502-5G	PHAR
Doxylamine	469-21-6	Sigma-Aldrich	D3775-5G	PHAR
Drospirenone	67392-87-4	Sigma-Aldrich	SML0147-10MG	PHAR
Duloxetine	116539-59-4	Sigma-Aldrich	PHR1865-500MG	PHAR
Ecgonine	481-37-8	Sigma-Aldrich	E-004-1ML	PHAR
Efavirenz	154598-52-4	Toronto Research Chemicals	E425000	PHAR
Enalapril	75847-73-3	Sigma-Aldrich	PHR1289-1G	PHAR
Ephedrine	299-42-3	Sigma-Aldrich	E-011-1ML	PHAR
Epinephrine	51-43-4	Sigma-Aldrich	E4250-1G	PHAR
Epitestosterone	481-30-1	Sigma-Aldrich	E-058-1ML	PHAR
Erythromycin	114-07-8	Sigma-Aldrich	E5389-1G	PHAR
Estriol	50-27-1	Sigma-Aldrich	E1253-100MG	PHAR
Estrone	53-16-7	Toronto Research Chemicals	E889050	PHAR
Ethosuximide	77-67-8	Sigma-Aldrich	PHR1413-1G	PHAR
Eugenol	97-53-0	Sigma-Aldrich	E51791-5G	PHAR
Fentanyl	437-38-7	Sigma-Aldrich	F-013-1ML	PHAR
Fexofenadine	83799-24-0	Toronto Research Chemicals	F322470	PHAR
Finafloxacin	209342-40-5	Sigma-Aldrich	SML2134-10MG	PHAR
Fingolimod	162359-55-9	Sigma-Aldrich	SML0700-5MG	PHAR

**Table S1.** List of unlabeled reference standards and isotope-labeled internal standards (continued)				
Compound Name	CAS	Supplier	Catalog Number	Category
Flecainide	54143-55-4	Sigma-Aldrich	F-017-1ML	PHAR
Fluconazole	86386-73-4	Sigma-Aldrich	PHR1160-1G	PHAR
Fluoxetine	54910-89-3	Sigma-Aldrich	F-918-1ML	PHAR
Flurandrenolide	1524-88-5	Sigma-Aldrich	1284000-100MG	PHAR
Furosemide	54-31-9	Sigma-Aldrich	F4381-1G	PHAR
Gabapentin	60142-96-3	Sigma-Aldrich	PHR1049-1G	PHAR
Gatifloxacin	112811-59-3	Sigma-Aldrich	32345-50MG	PHAR
Gemfibrozil	25812-30-0	Sigma-Aldrich	PHR1286-1G	PHAR
Glutethimide	77-21-4	Sigma-Aldrich	G-005-1ML	PHAR
Griseofulvin	126-07-8	Sigma-Aldrich	G4753-5G	PHAR
Guaifenesin	93-14-1	Toronto Research Chemicals	G810500	PHAR
Hydrochlorothiazide	58-93-5	Sigma-Aldrich	PHR1032-1G	PHAR
Hydrocodone	125-29-1	Sigma-Aldrich	H-003-1ML	PHAR
Hydrocortisone	50-23-7	Sigma-Aldrich	H0888-1G	PHAR
Hydromorphone	466-99-9	Sigma-Aldrich	H-004-1ML	PHAR
Hydroxyprogesterone	68-96-2	Sigma-Aldrich	H-085-1ML	PHAR
Ibuprofen	15687-27-1	Sigma-Aldrich	I4883-1G	PHAR
Icosapent	10417-94-4	Toronto Research Chemicals	E477800	PHAR
Imipenem	64221-86-9	Toronto Research Chemicals	I465200	PHAR
Iohexol	66108-95-0	Sigma-Aldrich	74147-50MG	PHAR
Irbesartan	138402-11-6	Sigma-Aldrich	PHR1443-1G	PHAR
Isopentedrone	1429402-11-8	Cayman Chemical Company	11563	PHAR
Isoprenaline	7683-59-2	Sigma-Aldrich	I5627-5G	PHAR
Ketamine	6740-88-1	Sigma-Aldrich	K-002-1ML	PHAR
Labetalol	36894-69-6	Sigma-Aldrich	PHR1335-1G	PHAR
Lamivudine	134678-17-4	Sigma-Aldrich	PHR1365-1G	PHAR
Lamotrigine	84057-84-1	Toronto Research Chemicals	L173250	PHAR
Levamisole	14769-73-4	Toronto Research Chemicals	L331100	PHAR
Levetiracetam	102767-28-2	Toronto Research Chemicals	L331500	PHAR
Levonorgestrel	797-63-7	Sigma-Aldrich	N2260-100MG	PHAR
Levorphanol	77-07-6	Sigma-Aldrich	L-044-1ML	PHAR
Lidocaine	137-58-6	Toronto Research Chemicals	L397800	PHAR
Lisinopril	76547-98-3	Sigma-Aldrich	PHR1143-1G	PHAR
Lomefloxacin	98079-51-7	Sigma-Aldrich	L2906-1G	PHAR
Lopinavir	192725-17-0	Toronto Research Chemicals	L469480	PHAR
Losartan	114798-26-4	Sigma-Aldrich	PHR1602-1G	PHAR
Lovastatin	75330-75-5	Toronto Research Chemicals	L472225	PHAR
Maprotiline	10262-69-8	Sigma-Aldrich	M9651-1G	PHAR
Mebendazole	31431-39-7	Sigma-Aldrich	M2523-25G	PHAR
Meclofenamic Acid	644-62-2	Sigma-Aldrich	M4531-1G	PHAR
Medroxyprogesterone	520-85-4	Sigma-Aldrich	M6013-250MG	PHAR
Mefenamic Acid	61-68-7	Sigma-Aldrich	92574-250MG	PHAR
Melatonin	73-31-4	Sigma-Aldrich	M5250-250MG	PHAR
Memantine	19982-08-2	Sigma-Aldrich	M9292-25MG	PHAR
Meperidine	57-42-1	Sigma-Aldrich	M-035-1ML	PHAR
Mesalamine	89-57-6	Sigma-Aldrich	PHR1060-1G	PHAR
Metacetamol (3-Acetamidophenol)	621-42-1	TCI America	H018825G	PHAR

**Table S1.** List of unlabeled reference standards and isotope-labeled internal standards (continued)				
Compound Name	CAS	Supplier	Catalog Number	Category
Metaproterenol (Orciprenaline)	586-06-1	Sigma-Aldrich	M2398-1G	PHAR
Metaraminol	54-49-9	Toronto Research Chemicals	M225565	PHAR
Metaxalone	1665-48-1	Sigma-Aldrich	M-074-1ML	PHAR
Metformin	657-24-9	Sigma-Aldrich	PHR1084-500MG	PHAR
Methadone	76-99-3	Sigma-Aldrich	M-007-1ML	PHAR
Methamphetamine	537-46-2	Sigma-Aldrich	M-009-1ML	PHAR
Methcathinone	5650-44-2	Sigma-Aldrich	M-061-1ML	PHAR
Methocarbamol	532-03-6	Sigma-Aldrich	PHR1395-1G	PHAR
Methoxyphedrine (Methedrone)	530-54-1	Cayman Chemical Company	10529	PHAR
Methylphenidate	113-45-1	Sigma-Aldrich	M-083-1ML	PHAR
Methyltestosterone	58-18-4	Sigma-Aldrich	M-906-1ML	PHAR
Metoprolol	51384-51-1	Sigma-Aldrich	PHR1076-1G	PHAR
Minoxidil	38304-91-5	Sigma-Aldrich	M4145-25MG	PHAR
Molindone	7416-34-4	Toronto Research Chemicals	M487500	PHAR
Monuron	150-68-5	AccuStandard	M-632-14	PHAR
Morphine	57-27-2	Sigma-Aldrich	M-005-1ML	PHAR
Moxifloxacin	151096-09-2	Sigma-Aldrich	PHR1542-1G	PHAR
Mycophenolic Acid	24280-93-1	Sigma-Aldrich	M3536-50MG	PHAR
Nadolol	42200-33-9	Sigma-Aldrich	N1892-1G	PHAR
Nalidixic Acid	389-08-2	Sigma-Aldrich	97023-100MG	PHAR
Nalorphine	62-67-9	Sigma-Aldrich	N-924-1ML	PHAR
Naloxone	465-65-6	Sigma-Aldrich	N-004-1ML	PHAR
Naproxen	22204-53-1	Toronto Research Chemicals	N377520	PHAR
Nebivolol	99200-09-6	Sigma-Aldrich	N1915-10MG	PHAR
N-Ethylamphetamine	33817-11-7	Sigma-Aldrich	E-018-1ML	PHAR
Nevirapine	129618-40-2	Toronto Research Chemicals	N391275	PHAR
Nicotine	54-11-5	Sigma-Aldrich	N-008-1ML	PHAR
Norepinephrine	51-41-2	Sigma-Aldrich	A7256-1G	PHAR
Norfloxacin	70458-96-7	Sigma-Aldrich	N9890-1G	PHAR
Nortriptyline	72-69-5	Sigma-Aldrich	N7261-10G	PHAR
Oxcarbazepine	28721-07-5	Sigma-Aldrich	O-025-1ML	PHAR
Oxprenolol	6452-71-7	Toronto Research Chemicals	O870500	PHAR
Oxycodone	76-42-6	Sigma-Aldrich	O-002-1ML	PHAR
Oxymorphone	76-41-5	Sigma-Aldrich	O-004-1ML	PHAR
Paliperidone	144598-75-4	Sigma-Aldrich	P0099-10MG	PHAR
Pentazocine	359-83-1	Sigma-Aldrich	P-073-1ML	PHAR
Pentedrone	879722-57-3	Sigma-Aldrich	P-087-1ML	PHAR
Pentobarbital	76-74-4	Sigma-Aldrich	P-010-1ML	PHAR
Perindopril	82834-16-0	Toronto Research Chemicals	P287500	PHAR
Phencyclidine	77-10-1	Sigma-Aldrich	P-007-1ML	PHAR
Phendimetrazine	634-03-7	Sigma-Aldrich	P-127-1ML	PHAR
Phenmetrazine	134-49-6	Sigma-Aldrich	P-128-1ML	PHAR
Phentermine	122-09-8	Sigma-Aldrich	P-023-1ML	PHAR
Phenylephrine	59-42-7	Sigma-Aldrich	PHR1017-500MG	PHAR
Phenytoin	57-41-0	Sigma-Aldrich	P-063-1ML	PHAR
Pilocarpine	92-13-7	Sigma-Aldrich	PHR1493-500MG	PHAR
Pirlimycin	79548-73-5	Toronto Research Chemicals	P509305	PHAR

Table S1. List of unlabeled reference standards and isotope-labeled internal standards (continued)

Compound Name	CAS	Supplier	Catalog Number	Category
Prasterone (Dehydroepiandrosterone; DHEA)	53-43-0	Sigma-Aldrich	D-063-1ML	PHAR
Pregabalin	148553-50-8	Sigma-Aldrich	P-066-1ML	PHAR
Prilocaine	721-50-6	Sigma-Aldrich	P9547-1G	PHAR
Primidone	125-33-7	Sigma-Aldrich	P-075-1ML	PHAR
Propafenone	54063-53-5	Sigma-Aldrich	P4670-5G	PHAR
Propoxyphene	469-62-5	Sigma-Aldrich	P-011-1ML	PHAR
Propranolol	525-66-6	Sigma-Aldrich	P0884-1G	PHAR
Protriptyline	438-60-8	Sigma-Aldrich	P8813-100MG	PHAR
Pseudoephedrine	90-82-4	Sigma-Aldrich	P-035-1ML	PHAR
Pyridoxine	65-23-6	Sigma-Aldrich	PHR1036-500MG	PHAR
Pyrimethamine	58-14-0	Sigma-Aldrich	46706-250MG	PHAR
Pyrovalerone	3563-49-3	Sigma-Aldrich	P-081-1ML	PHAR
Quinapril	85441-61-8	Toronto Research Chemicals	Q670000	PHAR
Ramipril	87333-19-5	Sigma-Aldrich	PHR1446-1G	PHAR
Ranitidine	66357-35-5	Sigma-Aldrich	R101-1G	PHAR
Rimantadine	13392-28-4	Toronto Research Chemicals	R517000	PHAR
Ropivacaine	84057-95-4	Sigma-Aldrich	R0283-10MG	PHAR
Sarafloxacin	98105-99-8	Sigma-Aldrich	33497-100MG-R	PHAR
Secobarbital	76-73-3	Sigma-Aldrich	S-002-1ML	PHAR
Sertraline	79617-96-2	Sigma-Aldrich	S-021-1ML	PHAR
Sitagliptin	486460-32-6	Sigma-Aldrich	PHR1857-1G	PHAR
Sotalol	3930-20-9	Toronto Research Chemicals	S677300	PHAR
Stavudine	3056-17-5	Toronto Research Chemicals	S685250	PHAR
Streptomycin	57-92-1	Sigma-Aldrich	46754-250MG	PHAR
Sulfadoxine	2447-57-6	Toronto Research Chemicals	S699070	PHAR
Sulfamethazine	57-68-1	Sigma-Aldrich	S6256-25G	PHAR
Sulfamethoxazole	723-46-6	Sigma-Aldrich	S7507-10G	PHAR
Sulfapyridine	144-83-2	Sigma-Aldrich	31738-250MG	PHAR
Sulfisomidine	515-64-0	Sigma-Aldrich	46908-250MG-R	PHAR
Tacrolimus	104987-11-3	Sigma-Aldrich	T-049-1ML	PHAR
Tapentadol	175591-23-8	Sigma-Aldrich	T-058-1ML	PHAR
Telmisartan	144701-48-4	Sigma-Aldrich	PHR1855-500MG	PHAR
Terbutaline	23031-25-6	Sigma-Aldrich	T2528-1G	PHAR
Testosterone	58-22-0	Sigma-Aldrich	T5411-1ML	PHAR
Thebaine	115-37-7	Sigma-Aldrich	T-116-1ML	PHAR
Theophylline	58-55-9	Toronto Research Chemicals	T343850	PHAR
Tiletamine	14176-49-9	Sigma-Aldrich	1667359-200MG	PHAR
Tolperisone	728-88-1	Sigma-Aldrich	T3577-50MG	PHAR
Tramadol	27203-92-5	Sigma-Aldrich	T-027-1ML	PHAR
Tranexamic Acid	1197-18-8	Toronto Research Chemicals	T714505	PHAR
Trenbolone	10161-33-8	Sigma-Aldrich	T-043-1ML	PHAR
Tretinoin (Retinoic Acid)	302-79-4	Sigma-Aldrich	R2625-50MG	PHAR
Triamterene	396-01-0	Sigma-Aldrich	T4143-10G	PHAR
Trihexyphenidyl	144-11-6	Sigma-Aldrich	T1516-5G	PHAR
Trimethoprim	738-70-5	Sigma-Aldrich	PHR1056-1G	PHAR
Valsartan	137862-53-4	Toronto Research Chemicals	V095750	PHAR
Varenicline	249296-44-4	Sigma-Aldrich	PZ0004-5MG	PHAR

**Table S1.** List of unlabeled reference standards and isotope-labeled internal standards (continued)				
Compound Name	CAS	Supplier	Catalog Number	Category
Venlafaxine	93413-69-5	Sigma-Aldrich	PHR1736-1G	PHAR
Xylazine	7361-61-7	Sigma-Aldrich	46995-100MG	PHAR
Zidovudine	30516-87-1	Toronto Research Chemicals	A825000	PHAR
1,7-Dimethyluric Acid	33868-03-0	Cayman Chemical Company	19584	PHAR TP
10,11-Dihydro-10-hydroxy Carbamazepine	29331-92-8	Sigma-Aldrich	D-091-1ML	PHAR TP
1-Carboxycyclohexaneacetic Acid	67950-95-2	Toronto Research Chemicals	G117290	PHAR TP
1-Methylxanthine	6136-37-4	Toronto Research Chemicals	M338575	PHAR TP
2-Ethyl-2-phenylmalonamide (PEMA)	7206-76-0	Sigma-Aldrich	95923-25MG	PHAR TP
2-Ethylidene-1,5-dimethyl-3,3-diphenylpyrrolidine (EDDP)	30223-73-5	Sigma-Aldrich	E-022-1ML	PHAR TP
3-Hydroxy Cotinine	34834-67-8	Sigma-Aldrich	H-101-1ML	PHAR TP
Androsterone	53-41-8	Sigma-Aldrich	31579-250MG	PHAR TP
Anhydroecgonine Methyl Ester	43021-26-7	Sigma-Aldrich	A-034-1ML	PHAR TP
Benzoylecgonine	519-09-5	Sigma-Aldrich	B-004-1ML	PHAR TP
Carbamazepine-10,11-epoxide	36507-30-9	Sigma-Aldrich	C-121-1ML	PHAR TP
Cotinine	486-56-6	Toronto Research Chemicals	C725000	PHAR TP
Ecgonine Methyl Ester	7143-09-1	Sigma-Aldrich	E-001-1ML	PHAR TP
Hydroxybupropion	92264-81-8	Sigma-Aldrich	H-066-1ML	PHAR TP
Metoprolol Acid (Atenolol Acid)	56392-14-4	Toronto Research Chemicals	M338785	PHAR TP
N4-Acetylsulfamethoxazole	21312-10-7	Toronto Research Chemicals	A187885	PHAR TP
N-Desmethyl Citalopram	62498-67-3	Sigma-Aldrich	D-047-1ML	PHAR TP
N-Desmethyl Tramadol	73806-55-0	Sigma-Aldrich	D-023-1ML	PHAR TP
N-Desmethyl Venlafaxine	149289-30-5	Toronto Research Chemicals	M266250	PHAR TP
Norcocaine	18717-72-1	Sigma-Aldrich	N-003-1ML	PHAR TP
Norcodeine	467-15-2	Sigma-Aldrich	N-005-1ML	PHAR TP
Norfentanyl	1609-66-1	Sigma-Aldrich	N-031-1ML	PHAR TP
Norhydrocodone	5083-62-5	Sigma-Aldrich	N-053-1ML	PHAR TP
Noroxycodone	57664-96-7	Sigma-Aldrich	N-011-1ML	PHAR TP
O-Desmethyl Tramadol	73986-53-5	Sigma-Aldrich	T-035-1ML	PHAR TP
Paraxanthine (1,7-Dimethylxanthine)	611-59-6	Sigma-Aldrich	IMPC-051-03-1ML	PHAR TP
Ritalinic Acid	19395-41-6	Sigma-Aldrich	R-011-1ML	PHAR TP
Salicylic Acid	69-72-7	Sigma-Aldrich	PHR1013-1G	PHAR TP
Tramadol N-Oxide	147441-56-3	Toronto Research Chemicals	T712530	PHAR TP
(4-Chloro-2-methylphenoxy)acetic Acid (MPCA)	94-74-6	AccuStandard	P-153S	PEST
2,4-Dichlorophenoxyacetic Acid (2,4-D)	94-75-7	Sigma-Aldrich	31518-250MG	PEST
2-Naphthoxyacetic Acid (NOA)	120-23-0	Sigma-Aldrich	N3019-25G	PEST
4-Chlorophenoxyacetic Acid (4-CPA)	122-88-3	Sigma-Aldrich	45391-250MG	PEST
8-Hydroxyquinoline	148-24-3	Sigma-Aldrich	H6878-25G	PEST
Abscisic Acid	21293-29-8	Sigma-Aldrich	90769-25MG	PEST
Acetamiprid	135410-20-7	AccuStandard	P-820S-CN	PEST
Aldicarb	116-06-3	AccuStandard	M-8318-01	PEST
Ametryn	834-12-8	AccuStandard	M-619-01	PEST
Atrazine	1912-24-9	Sigma-Aldrich	45330-250MG-R	PEST
Bentazon	25057-89-0	Sigma-Aldrich	32052-250MG	PEST
Bifenazate	149877-41-8	AccuStandard	P-772S	PEST
Bromacil	314-40-9	AccuStandard	P-181S	PEST
Butralin	33629-47-9	Sigma-Aldrich	36528-250MG	PEST
Camphor	76-22-2	AccuStandard	CP-TER-016S	PEST

**Table S1.** List of unlabeled reference standards and isotope-labeled internal standards (continued)				
Compound Name	CAS	Supplier	Catalog Number	Category
Carbaryl	63-25-2	AccuStandard	M-8318-03	PEST
Carbendazim	10605-21-7	AccuStandard	P-278S	PEST
Carbofuran	1563-66-2	AccuStandard	M-8318-04	PEST
Chloridazon	1698-60-8	AccuStandard	P-395S	PEST
Clofibric Acid	882-09-7	Sigma-Aldrich	90323-100MG	PEST
Clothianidin	210880-92-5	AccuStandard	P-947S	PEST
Cyprodinil	121552-61-2	Sigma-Aldrich	34389-100MG	PEST
Dichlorvos	62-73-7	AccuStandard	P-036S	PEST
Dimethachlor	50563-36-5	AccuStandard	P-642S	PEST
Dinotefuran	165252-70-0	AccuStandard	P-986S-CN	PEST
Dinoterb	1420-07-1	AccuStandard	P-524S	PEST
Diuron	330-54-1	AccuStandard	Z-031-07	PEST
Ethoxyquin	91-53-2	AccuStandard	P-388S-CN	PEST
Ethyl Butylacetylaminopropionate	52304-36-6	Toronto Research Chemicals	E900625	PEST
Fenamidone	161326-34-7	AccuStandard	P-850S-CN	PEST
Fipronil	120068-37-3	AccuStandard	P-738S	PEST
Fluometuron	2164-17-2	AccuStandard	P-014S	PEST
Fluridone	59756-60-4	AccuStandard	P-193S	PEST
Imazapyr	81334-34-1	AccuStandard	P-589S	PEST
Imidacloprid	138261-41-3	AccuStandard	P-596S	PEST
Indole-3-butyric Acid	133-32-4	Sigma-Aldrich	I5386-1G	PEST
Isoproturon	34123-59-6	AccuStandard	P-302S	PEST
Malathion	121-75-5	AccuStandard	P-060S	PEST
Mecoprop (MCPP)	93-65-2	AccuStandard	P-1053S-A	PEST
Metalaxyl	57837-19-1	AccuStandard	P-120S	PEST
Metolachlor	51218-45-2	Sigma-Aldrich	36163-100MG	PEST
Metsulfuron-methyl	74223-64-6	AccuStandard	P-463S	PEST
Napropamide	15299-99-7	AccuStandard	P-179S	PEST
Nitenpyram	150824-47-8	AccuStandard	P-858S-CN	PEST
Oxamyl	23135-22-0	AccuStandard	P-161S	PEST
Pendimethalin	40487-42-1	Sigma-Aldrich	36191-100MG	PEST
Piperonyl Butoxide	51-03-6	AccuStandard	P-348S	PEST
Pirimicarb	23103-98-2	AccuStandard	P-304S	PEST
Prometon	1610-18-0	Sigma-Aldrich	45635-50MG	PEST
Prometryn	7287-19-6	Sigma-Aldrich	45636-50MG	PEST
Propachlor	1918-16-7	AccuStandard	P-215S	PEST
Propamocarb	24579-73-5	AccuStandard	P-312S	PEST
Propazine	139-40-2	Sigma-Aldrich	45640-250MG	PEST
Pyracarbolid	24691-76-7	AccuStandard	P-792S-CN	PEST
Pyrimethanil	53112-28-0	Sigma-Aldrich	31577-250MG	PEST
Siduron	1982-49-6	AccuStandard	P-063S	PEST
Simazine	122-34-9	Sigma-Aldrich	32059-250MG	PEST
Sulfoxaflor	946578-00-3	AccuStandard	P-1133S	PEST
Terbumeton	33693-04-8	Sigma-Aldrich	31527-250MG	PEST
Terbuthylazine	5915-41-3	AccuStandard	M-619-10	PEST
Thiabendazole	148-79-8	AccuStandard	P-068S	PEST
Thiacloprid	111988-49-9	AccuStandard	P-838S-CN	PEST

**Table S1.** List of unlabeled reference standards and isotope-labeled internal standards (continued)				
Compound Name	CAS	Supplier	Catalog Number	Category
Thiamethoxam	153719-23-4	AccuStandard	P-866S-CN	PEST
Tridemorph	24602-86-6	AccuStandard	P-307S	PEST
Trinexapac-ethyl	95266-40-3	AccuStandard	P-1034S	PEST
2-Aminobenzimidazole	934-32-7	Sigma-Aldrich	171778-5G	PEST TP
Acetochlor Ethanesulfonic Acid (Acetochlor ESA)	187022-11-3	Sigma-Aldrich	34145-10MG	PEST TP
Alachlor Ethanesulfonic Acid (Alachlor ESA)	142363-53-9	Sigma-Aldrich	34147-10MG	PEST TP
Atrazine-2-hydroxy	2163-68-0	AccuStandard	P-326S	PEST TP
Atrazine-desethyl	6190-65-4	AccuStandard	P-343S	PEST TP
Atrazine-desisopropyl	1007-28-9	AccuStandard	P-345S	PEST TP
Carbofuran-3-hydroxy	16655-82-6	AccuStandard	M-8318-06	PEST TP
Metolachlor Ethanesulfonic Acid (Metolachlor ESA)	171118-09-5	Sigma-Aldrich	34149-10MG	PEST TP
Metolachlor Oxanilic Acid (Metolachlor OA)	152019-73-3	Sigma-Aldrich	34148-10MG	PEST TP
N-(2,4-Dimethylphenyl)formamide	60397-77-5	Sigma-Aldrich	592587-1G	PEST TP
Propachlor Oxanilic Acid (Propachlor OA)	70628-36-3	AccuStandard	P-921S	PEST TP
2-Hydroxybenzothiazole	934-34-9	Sigma-Aldrich	407607-5G	PCHI
4-Methyl-1H-benzotriazole	29878-31-7	Sigma-Aldrich	14593-50MG	PCHI
5-Methyl-1H-benzotriazole	136-85-6	Sigma-Aldrich	196304-10G	PCHI
4-Propylbenzoic Acid	2438-05-3	Alfa Aesar	AAA1453804	PCHI
Acesulfame	33665-90-6	Sigma-Aldrich	47134	PCHI
Benzophenone	119-61-9	Sigma-Aldrich	B9300-25G-A	PCHI
Oxybenzone (Benzophenone-3)	131-57-7	Sigma-Aldrich	H36206-5G	PCHI
Benzothiazole	95-16-9	Sigma-Aldrich	101338-5G	PCHI
Benzotriazole	95-14-7	Sigma-Aldrich	B11400-100G	PCHI
Benzyl Butyl Phthalate	85-68-7	Sigma-Aldrich	308501-5ML	PCHI
Butylparaben	94-26-8	AccuStandard	ALR-085S	PCHI
Dibutyl Phthalate	84-74-2	Sigma-Aldrich	524980-25ML	PCHI
Diethyl Phthalate	84-66-2	Sigma-Aldrich	524972-5ML	PCHI
Diisobutyl Phthalate	84-69-5	Sigma-Aldrich	152641-100ML	PCHI
Dimethyl Phthalate	131-11-3	Sigma-Aldrich	41320-1ML-F	PCHI
Diphenylphosphinic Acid	1707-03-5	Sigma-Aldrich	43153-5G	PCHI
Ethylparaben	120-47-8	AccuStandard	ALR-113S	PCHI
Icaridin	119515-38-7	AccuStandard	BIOC-228S-CN	PCHI
Indole-4-carboxaldehyde	1074-86-8	Alfa Aesar	AAH28019MD	PCHI
Isopropylparaben	4191-73-5	AccuStandard	ALR-122S	PCHI
Melamine	108-78-1	Sigma-Aldrich	M2659-5G	PCHI
N-Butylbenzenesulfonamide	3622-84-2	TCI America	B071625G	PCHI
N,N-Diethyl-3-methylbenzamide (DEET)	134-62-3	Sigma-Aldrich	36542-250MG	PCHI
Osthole	484-12-8	Cayman Chemical Company	19195	PCHI
Perfluorobutanoic Acid (PFBA)	375-22-4	AccuStandard	PFOA-002S	PCHI
Perfluoroheptanoic Acid (PFHpA)	375-85-9	AccuStandard	PFOA-005S	PCHI
Perfluorohexanoic Acid (PFHxA)	307-24-4	AccuStandard	PFOA-006S	PCHI
Perfluorononanoic Acid (PFNA)	375-95-1	AccuStandard	PFOA-007S	PCHI
Perfluorooctanesulfonic Acid (PFOS)	1763-23-1	AccuStandard	PFOS-001S	PCHI
Perfluoropentanoic Acid (PFPeA)	2706-90-3	AccuStandard	PFOA-008S	PCHI
Propylparaben	94-13-3	AccuStandard	ALR-153S	PCHI
Sucralose	56038-13-2	Toronto Research Chemicals	S692500	PCHI
Tributyl Phosphate	126-73-8	Sigma-Aldrich	240494-5ML	PCHI

Table S1. List of unlabeled reference standards and isotope-labeled internal standards (continued)

Compound Name	CAS	Supplier	Catalog Number	Category
Triclocarban	101-20-2	Sigma-Aldrich	PHR1303-500MG	PCHI
Triclosan	3380-34-5	Sigma-Aldrich	72779-5G-F	PCHI
Triisopropanolamine	122-20-3	ACROS Organics	AC245761000	PCHI
Tris(1,3-dichloro-2-propyl) Phosphate (TDCPP)	13674-87-8	Toronto Research Chemicals	T876305	PCHI
Tris(2-chloroethyl) Phosphate (TCEP)	115-96-8	Sigma-Aldrich	119660-25G	PCHI
1H-Benzotriazole-5-carboxylic Acid	23814-12-2	Sigma-Aldrich	304239-5G	PCHI TP
1-Methyl-1H-benzotriazole	13351-73-0	Toronto Research Chemicals	M289810	PCHI TP
Carbanilide	102-07-8	Sigma-Aldrich	142158-25G	PCHI TP
Galaxolidone	507442-49-1	Toronto Research Chemicals	G189005	PCHI TP
3,4-Methylenedioxymethamphetamine-d_5	136765-43-0	Sigma-Aldrich	M-011-1ML	ILIS
Acetaminophen-d_3 (N-(4-Hydroxyphenyl)acetamide-2,2,2-d_3)	60902-28-5	C/D/N Isotopes	D-6324	ILIS
Amphetamine-d_{10}	169565-17-7	Cambridge Isotope Laboratories	A-038-1ML	ILIS
Atenolol-d_7	1202864-50-3	C/D/N Isotopes	D-6202	ILIS
Buprenorphine-d_4	136781-89-0	Sigma-Aldrich	B-901-1ML	ILIS
Bupropion-d_9	1189725-26-5	Sigma-Aldrich	B-052-1ML	ILIS
Caffeine-d_9	72238-85-8	C/D/N Isotopes	D-5972	ILIS
Carbamazepine-d_{10}	132183-78-9	C/D/N Isotopes	D-3542	ILIS
Cimetidine-d_3	1185237-29-9	C/D/N Isotopes	D-6876	ILIS
Codeine-d_3	70420-71-2	Sigma-Aldrich	C-005-1ML	ILIS
Cotinine-d_3	110952-70-0	C/D/N Isotopes	D-3518	ILIS
Diclofenac-d_4	153466-65-0	C/D/N Isotopes	D-6183	ILIS
Diphenhydramine-d_3	170082-18-5	Sigma-Aldrich	D-017-1ML	ILIS
Dopamine-d_4	203633-19-6	Cambridge Isotope Laboratories	D-072-1ML	ILIS
Ephedrine-d_3	285979-73-9	Sigma-Aldrich	E-025-1ML	ILIS
Fentanyl-d_5	118357-29-2	Cambridge Isotope Laboratories	F-001-1ML	ILIS
Fluconazole-$^{13}C_3$	NA	Sigma-Aldrich	F-035-1ML	ILIS
Gabapentin-d_{10}	1126623-20-8	Cambridge Isotope Laboratories	G-901-1ML	ILIS
Gemfibrozil-d_6	1184986-45-5	C/D/N Isotopes	D-6144	ILIS
Hydrocodone-d_3	136765-36-1	Sigma-Aldrich	H-005-1ML	ILIS
Hydromorphone-d_3	136765-37-2	Sigma-Aldrich	H-006-1ML	ILIS
Lamotrigine-$^{13}C,^{15}N_4$	NA	Cambridge Isotope Laboratories	L-022-1ML	ILIS
Levetiracetam-d_6	1435933-72-4	Cambridge Isotope Laboratories	L-023-1ML	ILIS
Lidocaine-d_{10}	851528-09-1	C/D/N Isotopes	D-6745	ILIS
Meperidine-d_4	53484-73-4	Sigma-Aldrich	M-036-1ML	ILIS
Metaxalone-d_6	NA	Sigma-Aldrich	M-143-1ML	ILIS
Metformin-d_6	1185166-01-1	Sigma-Aldrich	53183-5MG	ILIS
Methadone-d_3	60263-63-0	Cambridge Isotope Laboratories	M-008-1ML	ILIS
Methamphetamine-d_8	136765-40-7	Cambridge Isotope Laboratories	M-016-1ML	ILIS
Methocarbamol-d_3	1346600-86-9	Sigma-Aldrich	M-202-1ML	ILIS
Metoprolol-d_7	1219798-61-4	C/D/N Isotopes	D-6682	ILIS
Morphine-d_3	67293-88-3	Cambridge Isotope Laboratories	M-003-1ML	ILIS
Naproxen-d_3	958293-77-1	C/D/N Isotopes	D-6523	ILIS
Nicotine-d_4	350818-69-8	Cambridge Isotope Laboratories	N-048-1ML	ILIS
Oxycodone-d_3	160227-46-3	Cambridge Isotope Laboratories	O-005-1ML	ILIS
Oxymorphone-d_3	145225-03-2	Sigma-Aldrich	O-003-1ML	ILIS
Phendimetrazine-d_5	NA	Sigma-Aldrich	P-132-1ML	ILIS
Phentermine-d_5	1330236-21-9	Sigma-Aldrich	P-034-1ML	ILIS

**Table S1.** List of unlabeled reference standards and isotope-labeled internal standards (continued)				
Compound Name	CAS	Supplier	Catalog Number	Category
Pregabalin-d_6	NA	Cambridge Isotope Laboratories	P-072-1ML	ILIS
Protriptyline-d_3	1435934-21-6	Cambridge Isotope Laboratories	P-088-1ML	ILIS
Sulfamethoxazole-d_4	1020719-86-1	C/D/N Isotopes	D-7398	ILIS
Tramadol-$^{13}C,d_3$	NA	Cambridge Isotope Laboratories	T-029-1ML	ILIS
Trimethoprim-d_9	1189460-62-5	Sigma-Aldrich	32414-10MG	ILIS
Venlafaxine-d_6	1062606-12-5	C/D/N Isotopes	D-6826	ILIS
2-Ethylidene-1,5-dimethyl-3,3-diphenylpyrrolidine-d_3 (EDDP-d_3)	136765-23-6	Cambridge Isotope Laboratories	E-021-1ML	ILIS
3-Hydroxy Cotinine-d_3	159956-78-2	Cambridge Isotope Laboratories	H-108-1ML	ILIS
Benzoylecgonine-d_3	115732-68-8	Cambridge Isotope Laboratories	B-001-1ML	ILIS
Ecgonine Methyl Ester-d_3	136765-34-9	Sigma-Aldrich	E-002-1ML	ILIS
Hydroxybupropion-d_6	1184984-06-2	Cambridge Isotope Laboratories	H-062-1ML	ILIS
Norcodeine-d_3	NA	Sigma-Aldrich	N-082-1ML	ILIS
Norfentanyl-d_5	1211527-23-9	Sigma-Aldrich	N-030-1ML	ILIS
Norhydrocodone-d_3	NA	Sigma-Aldrich	N-054-1ML	ILIS
Noroxycodone-d_3	1426174-79-9	Cambridge Isotope Laboratories	N-032-1ML	ILIS
Ritalinic Acid-d_{10}	NA	Cambridge Isotope Laboratories	R-014-1ML	ILIS
2,4-Dichlorophenoxyacetic Acid-d_3 (2,4-D-d_3)	202480-67-9	C/D/N Isotopes	D-5750	ILIS
Atrazine-d_5	163165-75-1	C/D/N Isotopes	D-4389	ILIS
Clofibric Acid-d_4	1184991-14-7	C/D/N Isotopes	D-6005	ILIS
Diuron-d_6	1007536-67-5	Sigma-Aldrich	34018-10MG-R	ILIS
Imidacloprid-d_4	1015855-75-0	C/D/N Isotopes	D-7456	ILIS
Mecoprop-d_3	352431-15-3	C/D/N Isotopes	D-5321	ILIS
Metalaxyl-d_3	NA	Sigma-Aldrich	08963-5MG	ILIS
Metolachlor-d_6	1219803-97-0	C/D/N Isotopes	D-5647	ILIS
Prometon-d_3	1219803-43-6	C/D/N Isotopes	D-6802	ILIS
Benzotriazole-d_4	1185072-03-0	C/D/N Isotopes	D-7358	ILIS
N,N-Diethyl-3-methyl-d_3-benzamide-2,4,5,6-d_4 (DEET-d_7)	1219799-37-7	C/D/N Isotopes	D-6756	ILIS
Oxybenzone-d_5	1219798-54-5	Sigma-Aldrich	73875-10MG	ILIS
Sucralose-d_6	1459161-55-7	Toronto Research Chemicals	S692502	ILIS
Triclosan-d_3	1020719-98-5	C/D/N Isotopes	D-6983	ILIS
Perfluorooctanoic Acid-$^{13}C_8$	1350614-84-4	Cambridge Isotope Laboratories	ES-5610	ILIS
Sodium Perfluorooctanesulfonate-$^{13}C_8$	NA	Cambridge Isotope Laboratories	ES-5610	ILIS
N-Methylperfluorooctanesulfonamidoacetic Acid-d_3	1400690-70-1	Cambridge Isotope Laboratories	ES-5610	ILIS

"PHAR" = pharmaceutical; "PEST" = pesticide; "PCHI" = personal care, household and industrial chemical; "TP" = transformation product; "ILIS" = isotope-labeled internal standard.

S2. Sampling site coordinates and sampling dates

Table S2. Summary of sampling site coordinates and sampling dates

Site ID	Site Name	Site Coordinates	Sampling Dates	USGS Gauge Station
L1	Onondaga Lake at South End	43.088765, -76.202514	6/28/2017, 7/11/2017, 7/25/2017, 8/10/2017, 8/22/2017, 9/6/2017, 9/28/2017, 10/16/2017	-
L2	Onondaga Lake at South Deep	43.078282, -76.198445	6/28/2017, 7/11/2017, 7/25/2017, 8/10/2017, 8/22/2017, 9/6/2017, 9/28/2017, 10/16/2017	-
L3	Onondaga Lake at North Deep	43.084590, -76.193514	6/28/2017, 7/11/2017, 7/25/2017, 8/10/2017, 8/22/2017, 9/6/2017, 9/28/2017, 10/16/2017	-
L4	Onondaga Lake at Outlet	43.117151, -76.244931	6/28/2017, 7/11/2017, 7/25/2017, 8/10/2017, 8/22/2017, 9/6/2017, 9/28/2017, 10/16/2017	04240503 (discontinued)
T1	Ninemile Creek	43.080750, -76.225935	6/28/2017, 7/11/2017, 7/25/2017, 8/10/2017, 8/22/2017, 9/6/2017, 9/28/2017, 10/16/2017	04240300
T2	Onondaga Creek	43.059965, -76.163322	6/28/2017, 7/11/2017, 7/25/2017, 8/10/2017, 8/22/2017, 9/6/2017, 9/28/2017, 10/16/2017	04240010
T3	Harbor Brook	43.057910, -76.182777	6/28/2017, 7/11/2017, 7/25/2017, 8/10/2017, 8/22/2017, 9/6/2017, 9/28/2017, 10/16/2017	04240120
T4	Ley Creek	43.057910, -76.182777	6/28/2017, 7/11/2017, 7/25/2017, 8/10/2017, 8/22/2017, 9/6/2017, 9/28/2017, 10/16/2017	04240105
R294	Seneca River	43.124006, -76.264286	7/11/2017, 10/16/2017	04237496
W266	Seneca River	43.131334, -76.242741	7/11/2017, 10/16/2017	-
W259	Seneca River	43.150243, -76.234730	7/11/2017, 10/16/2017	-
R212	Oneida River	43.202939, -76.274370	7/11/2017, 10/16/2017	04247000
R10	Oswego River	43.218982, -76.278499	7/11/2017, 10/16/2017	04247055
R24	Oswego River	43.230055, -76.295944	7/11/2017, 10/16/2017	-
R78	Oswego River	43.314649, -76.410616	7/11/2017, 10/16/2017	-
R99	Oswego River	43.358322, -76.415021	7/11/2017, 10/16/2017	-
R124	Oswego River	43.396077, -76.459584	7/11/2017, 10/16/2017	-
G135	Oswego River	43.408510, -76.464358	7/11/2017, 10/16/2017	-
G143	Oswego River	43.440819, -76.480331	7/11/2017, 10/16/2017	-
R6	Oswego River	43.459983, -76.500604	7/11/2017, 10/16/2017	04249000
WWTP	Regional WWTP Outfall	43.064167, -76.178056	6/28/2017, 7/11/2017, 7/25/2017, 8/10/2017, 8/22/2017, 9/6/2017, 9/28/2017, 10/16/2017	-

S3. Analysis of water quality parameters and optical properties

Water quality parameters for lake, tributary, and river water samples (i.e., temperature, specific conductance, pH, dissolved oxygen (DO), turbidity, and fluorometric chlorophyll *a*) were measured *in situ* at each sampling site by the UFI sampling crew with a YSI 6600 multiparameter sonde.[1, 2] Water quality parameters (i.e., temperature, specific conductance, 5-day biochemical oxygen demand (BOD_5), DO, turbidity, and ammonia-nitrogen (NH_3-N)) for wastewater effluent samples were monitored by the regional WWTP in compliance with the New York State Pollutant Discharge Elimination System Permit.

Upon return to the laboratory, small aliquots of samples (~40 mL) were filtered through precombusted 0.45-μm glass fiber filters and analyzed for dissolved organic carbon (DOC) and optical properties. Briefly, DOC was measured by high-temperature catalytic combustion using a Teledyne-Tekmar Torch total organic carbon analyzer. Fluorescence excitation-emission matrices (EEMs) and UV-visible absorbance spectra were simultaneously measured in a Starna Cells 3-Q-10 quartz cuvette (1-cm pathlength) using a Horiba Scientific Aqualog spectrofluorometer as described in our previous work.[3] Optical indices, such as Napierian absorption coefficients (*a*)[4] at 254 nm, 280 nm, and 440 nm,[5-7] *E2*:*E3* (the ratio of absorption coefficients at 250 and 365 nm; an inverse proxy of dissolved organic matter (DOM) molecular size),[8] spectral slope ratio (S_R; the ratio of spectral slope coefficient $S_{275\text{-}295}$ to spectral slope coefficient $S_{290\text{-}350}$; an indicator of environmental processing of DOM),[9] $CDOM_{250\text{-}450}$ (the integrated absorption of chromophoric DOM from 250 to 450 nm),[9, 10] $SUVA_{254}$ (the specific UV absorbance at 254 nm; a proxy of DOM aromaticity),[11] fluorescence index (FI; an indicator of the source of DOM, which is either microbially derived from bacteria and algae or terrestrially derived from plant litter and soil),[12, 13] humification index (HIX; an indicator of the degree of DOM humification),[14-16] freshness index ($\beta{:}\alpha$; an indicator of the contribution of recently plant-derived or autochthonous DOM, where β represents more recently produced DOM and α represents more decomposed DOM),[17-19] and FDOM (the integrated volumetric fluorescence intensity of fluorescent DOM with excitation wavelengths from 240 to 550 nm and emission wavelengths from 248.2 to 600.9 nm normalized to the Raman peak area of ultrapure water),[20, 21] were extracted from the absorbance and fluorescence data using *MATLAB R2019a*.

Parallel factor analysis (PARAFAC) was performed to deconvolute EEMs using the *drEEM* toolbox (v. 0.5.1)[22] in *MATLAB*. Five PARAFAC models (3-7 components) were evaluated using non-negativity constraints on all modes with 50 iterations and a convergence criterion of 10^{-10} to locate the minimum sum of squared errors.[23] Prior to PARAFAC modeling, raw EEMs (n = 143) were trimmed to the excitation wavelength range of 240 to 450 nm and the emission wavelength range of 248.242 to 598.548 nm, smoothed following the removal of primary and secondary Rayleigh and Raman scatter, and normalized to minimize the concentration-dependent co-linearity.[22] Upon examination of the core consistencies (**Figure S1**), sum of squared errors (**Figure S2**), and $S_4C_6T_3$ split-half validations (**Figure S3**),[22] a 4-component model was identified as the most appropriate model that explained 99.7% of the measured spectral variation across reverse-normalized EEMs (**Figures S4-S5**). A 6-component model was also validated but was excluded from further consideration due to atypical features (e.g., abrupt changes over narrow wavelength ranges[22]) in certain spectral loadings. Following the model validation, the true scores were converted to the maximum fluorescence intensity (F_{max}) to generate intensities in water Raman unit (R.U.) for each component,[22] and the excitation and emission wavelengths of individual components (**Figure S6**) were queried against those published in the *OpenFluor* database[24] using a Tucker's congruence coefficient criterion of 0.95. Four PARAFAC components were operationally defined as component 1 (C1), component 2 (C2), component 3 (C3), and component 4 (C4), respectively. For C4, the emission feature above 430 nm likely stemmed from leftover physical scatter.[25] C2 had the highest number of matches in the *OpenFluor* database at the time of access (October 2021), followed by C1, C4, and C3, respectively. C1 is a microbial humic-like component (aliphatic, low molecular weight),[26, 27] C2 is a terrestrial humic-like component (high aromaticity, high molecular weight),[26, 27] C3 is a humic-like component (low aromaticity, low molecular weight) associated with urban and/or agricultural nonpoint source runoff,[26-28] and C4 is a tryptophan-like component (aliphatic, low molecular weight) associated with wastewater discharge.[26, 27, 29] Water quality parameters are summarized in **Table S2**. Optical indices and the F_{max} values of PARAFAC components are summarized in **Table S3**.

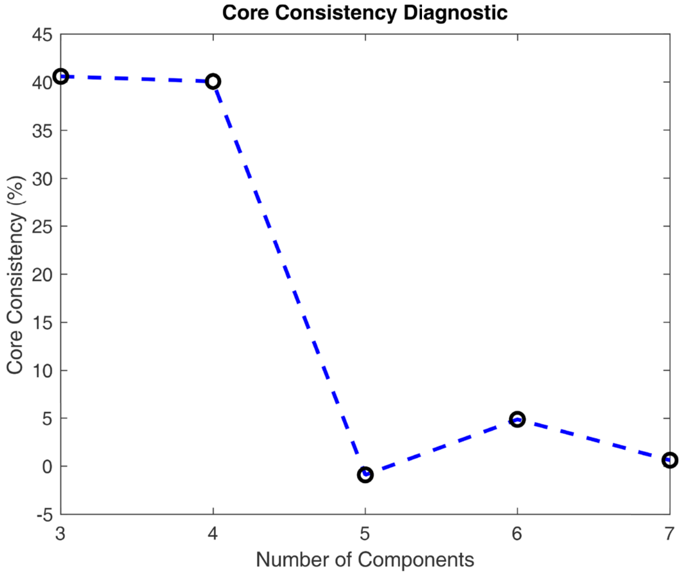

Figure S1. Core consistencies of the 3-, 4-, 5-, 6-, and 7-component PARAFAC models.

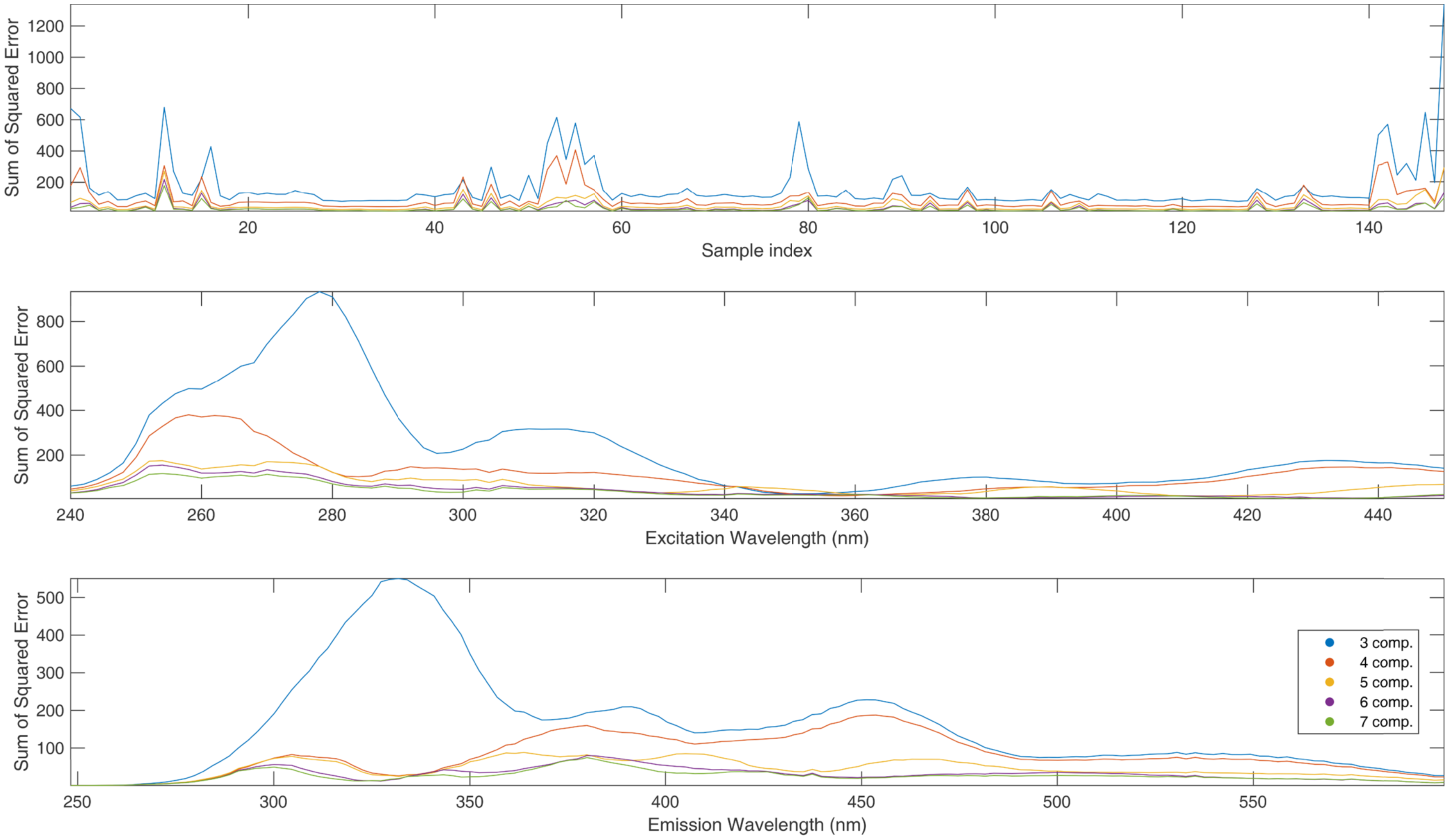

Figure S2. Sum of squared errors for the 3-, 4-, 5-, 6-, and 7-component PARAFAC models.

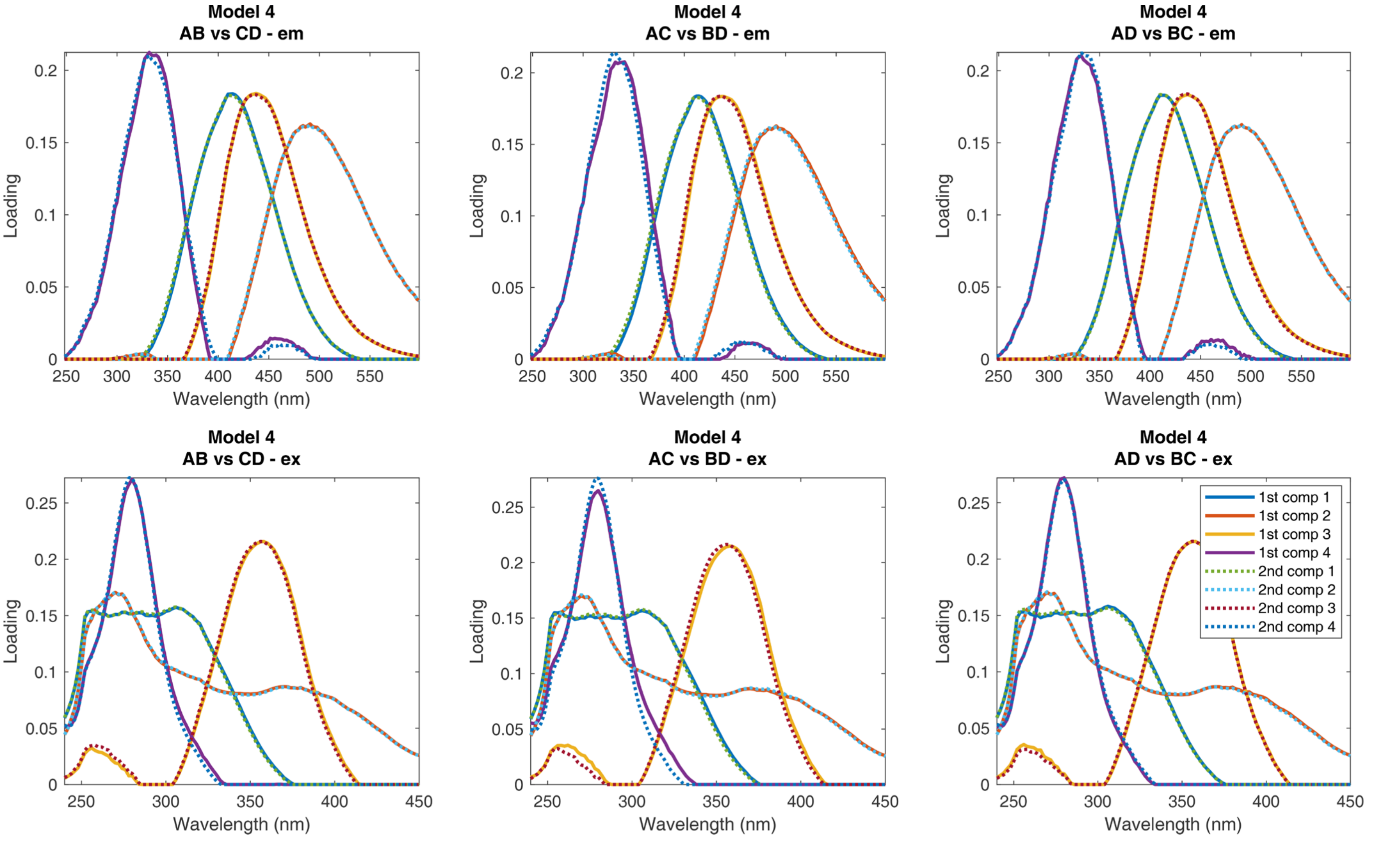

Figure S3. Split-half comparisons of the excitation and emission spectra from the 4-component PARAFAC model.

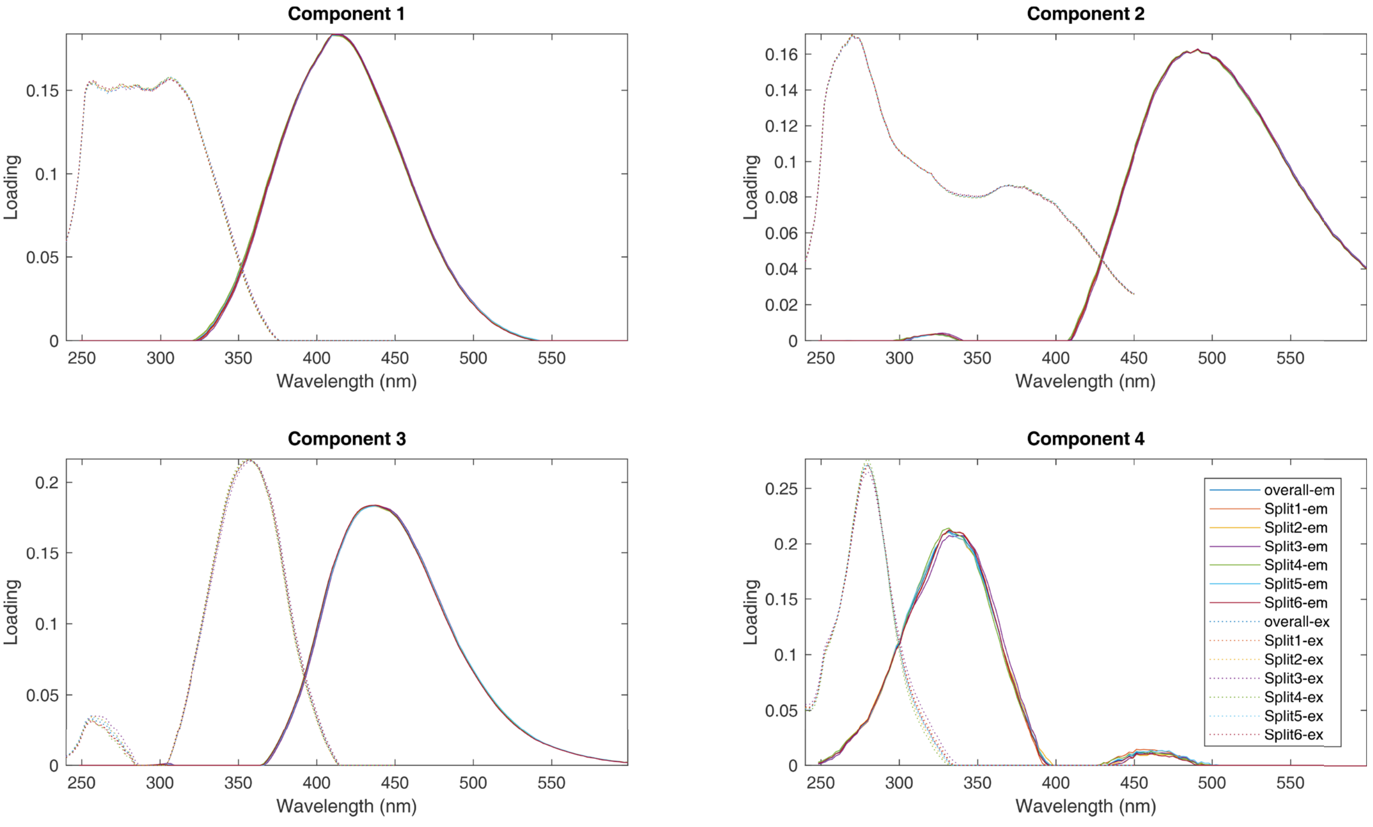

Figure S4. Overlaid spectral loadings of the 4-component PARAFAC model versus the overall model.

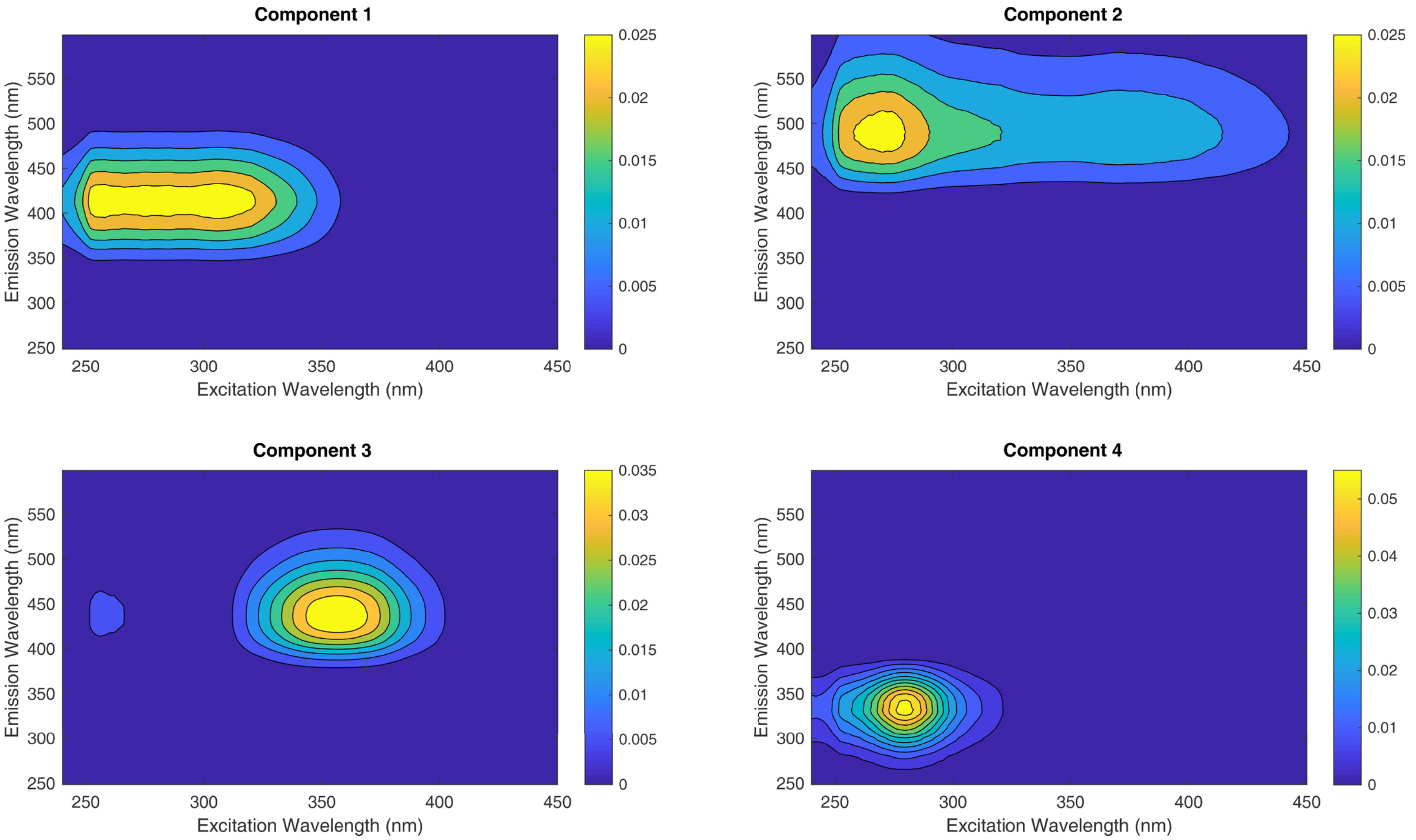

Figure S5. Contour plots of 4 fluorescent components validated for the 4-component PARAFAC model.

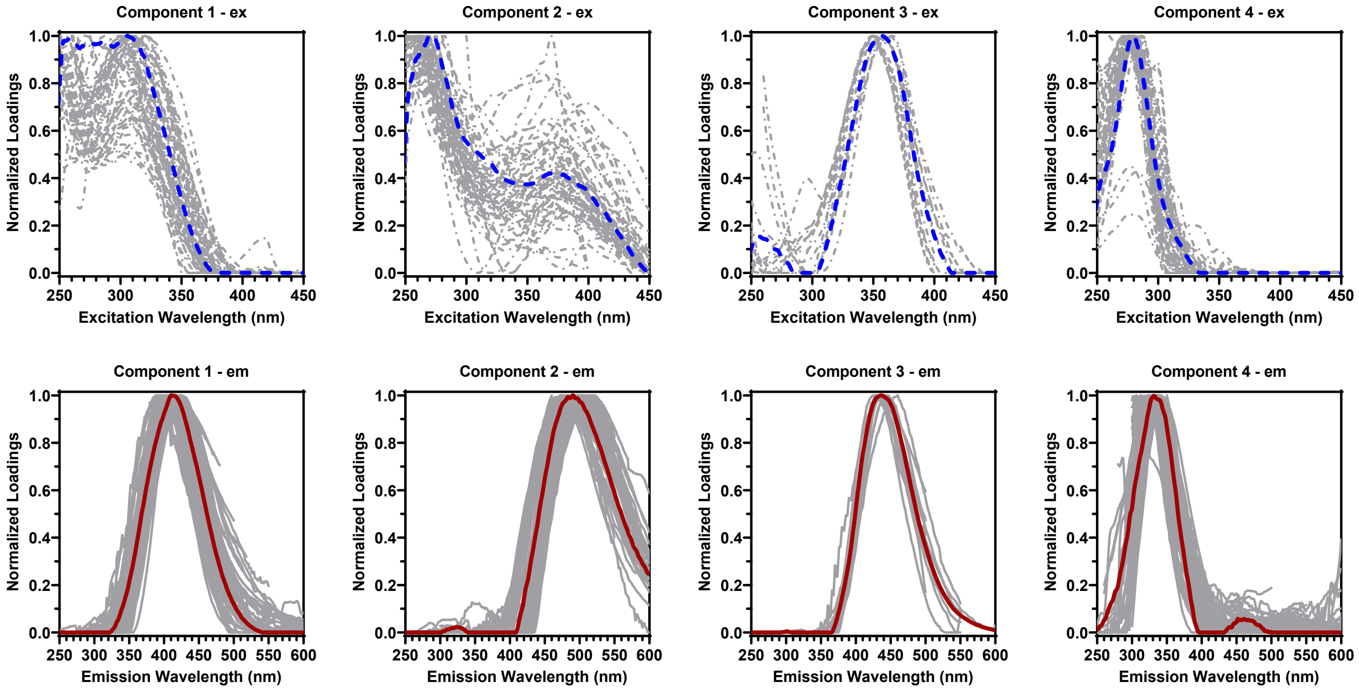

Figure S6. Spectral comparisons of the 4-component PARAFAC model with those published in the *OpenFluor* database.

**Table S3.** Water quality of samples from the Onondaga Lake-Three Rivers system										
Site ID	Site Coordinates	Sampling Date	Depth (m)	Temperature (°C)	Specific Cond. (µS/cm)	pH	DO (mg/L)	Turbidity (NTU)	Chlorophyll *a* (µg/L)	DOC (mg-C/L)
Onondaga Lake at South End										
L1	43.088765, -76.202514	6/28/2017	3.0	21.91	1612	8.05	9.73	2.7	5.2	1.81
		7/11/2017	3.0	23.65	1446	8.24	9.30	3.2	8.2	2.15
		7/25/2017	3.0	23.06	1377	8.09	8.43	2.6	6.1	2.02
		8/10/2017	3.0	23.66	1526	8.39	10.83	2.3	9.5	1.85
		8/22/2017	3.0	24.48	1632	8.09	9.10	2.2	9.6	1.70
		9/6/2017	3.0	20.04	1709	8.07	9.19	2.8	15.2	1.83
		9/28/2017	3.0	24.20	1810	8.10	11.90	2.5	20.0	2.00
		10/16/2017	3.0	18.45	1871	7.71	7.78	1.5	5.5	1.73
Onondaga Lake at South Deep										
L2	43.078282, -76.198445	6/28/2017	1.0	22.11	1604	8.14	9.60	3.7	6.6	1.84
		6/28/2017	17.0	10.70	1544	7.35	0.55	10.7	0.4	1.77
		7/11/2017	1.0	23.44	1445	8.04	8.19	NA	5.6	2.12
		7/11/2017	17.0	10.83	1551	7.37	0.42	NA	1.6	1.72
		7/25/2017	1.0	23.63	1416	8.05	8.81	2.2	5.5	1.71
		7/25/2017	2.0	23.63	1416	8.05	8.89	2.2	5.7	2.00
		7/25/2017	3.0	23.63	1416	8.05	8.86	2.0	5.8	2.01
		7/25/2017	4.0	23.62	1416	8.05	8.85	2.0	4.7	2.03
		7/25/2017	5.0	23.59	1418	8.03	8.74	2.3	6.9	2.03
		7/25/2017	6.0	23.53	1420	8.00	8.42	2.4	4.8	2.07
		7/25/2017	7.0	18.55	1724	7.36	1.32	6.9	2.8	1.69
		7/25/2017	8.0	16.66	1624	7.29	0.61	2.6	4.9	1.58
		7/25/2017	9.0	15.11	1593	7.28	0.53	1.9	2.1	NA
		7/25/2017	10.0	13.81	1578	7.27	0.50	1.6	1.5	1.68
		7/25/2017	11.0	13.26	1573	7.26	0.49	1.3	2.4	NA
		7/25/2017	12.0	12.75	1571	7.27	0.47	1.6	3.3	1.62
		7/25/2017	13.0	11.91	1566	7.28	0.46	2.3	2.6	1.64
		7/25/2017	14.0	11.55	1564	7.29	0.47	1.2	2.2	0.93
		7/25/2017	15.0	11.23	1565	7.30	0.44	1.8	1.1	1.79
		7/25/2017	16.0	11.05	1566	7.32	0.44	1.4	2.0	NA
		7/25/2017	17.0	10.92	1567	7.33	0.44	1.5	1.3	1.71
		7/25/2017	18.0	10.47	1586	7.34	0.46	2.7	1.8	0.96
		8/10/2017	1.0	24.23	1517	8.41	8.77	3.8	9.4	NA
		8/10/2017	17.0	11.09	1575	7.45	0.42	3.5	3.1	1.64
		8/22/2017	1.0	24.61	1605	7.69	8.35	5.0	8.5	1.50
		8/22/2017	17.0	11.03	1575	6.99	0.32	5.0	1.2	1.81
		9/6/2017	1.0	19.87	1725	8.02	9.04	2.5	10.2	1.73
		9/6/2017	17.0	11.26	1578	7.30	0.48	5.9	2.6	1.81
		9/28/2017	1.0	23.24	1796	8.19	12.75	2.1	15.0	2.03
		9/28/2017	17.0	11.41	1683	7.31	0.51	3.6	1.8	1.72
		10/16/2017	1.0	18.49	1868	7.95	8.17	1.9	7.8	1.83
		10/16/2017	2.0	18.48	1867	7.95	8.16	1.8	6.7	1.81
		10/16/2017	3.0	18.49	1868	7.95	8.10	1.8	8.1	1.77
		10/16/2017	4.0	18.50	1868	7.94	8.18	1.9	7.2	1.75
		10/16/2017	5.0	18.50	1867	7.93	8.07	1.8	8.2	1.74
		10/16/2017	6.0	18.49	1868	7.93	8.14	2.0	7.0	1.75
		10/16/2017	7.0	18.49	1868	7.93	8.09	2.0	8.8	1.76
		10/16/2017	8.0	18.48	1868	7.92	8.01	2.0	7.0	1.71
		10/16/2017	9.0	18.19	1882	7.73	5.97	2.0	4.7	1.67
		10/16/2017	10.0	16.41	1918	7.39	1.28	2.3	6.4	1.48
		10/16/2017	11.0	15.04	1875	7.38	0.60	1.9	0.9	1.46
		10/16/2017	12.0	14.00	1821	7.38	0.53	1.9	3.9	1.46
		10/16/2017	13.0	12.88	1773	7.38	0.52	2.2	3.2	1.46
		10/16/2017	14.0	12.20	1726	7.38	0.51	2.6	2.5	1.47
		10/16/2017	15.0	12.03	1716	7.38	0.48	3.4	0.1	1.47
		10/16/2017	16.0	11.81	1710	7.39	0.48	3.5	2.1	1.49
		10/16/2017	17.0	11.72	1706	7.39	0.48	4.2	2.1	1.49
		10/16/2017	18.0	11.56	1711	7.39	0.48	4.3	2.3	1.46
Onondaga Lake at North Deep										
L3	43.084590, -76.193514	6/28/2017	1.0	21.89	1595	8.08	10.16	2.1	4.3	1.86
		6/28/2017	17.0	10.65	1554	7.32	0.44	2.0	2.1	NA
		7/11/2017	1.0	23.36	1437	8.19	9.29	3.1	8.6	2.09
		7/11/2017	17.0	10.78	1555	7.37	0.57	1.9	1.0	1.72
		7/25/2017	1.0	23.24	1336	8.32	9.76	1.9	7.8	NA
		7/25/2017	17.0	10.64	1532	7.46	0.55	1.3	2.5	1.74
		8/10/2017	1.0	23.11	1523	8.28	10.31	1.5	6.8	NA

**Table S3.** Water quality of samples from the Onondaga Lake-Three Rivers system (continued)										
Site ID	Site Coordinates	Sampling Date	Depth (m)	Temperature (°C)	Specific Cond. (μS/cm)	pH	DO (mg/L)	Turbidity (NTU)	Chlorophyll *a* (μg/L)	DOC (mg-C/L)
L3	43.084590, -76.193514	8/10/2017	17.0	10.75	1582	7.45	0.56	2.9	2.4	1.59
		8/22/2017	1.0	24.79	1578	8.26	10.55	1.6	10.7	2.78
		8/22/2017	17.0	11.02	1586	7.35	0.61	2.4	3.5	1.45
		9/6/2017	1.0	20.40	1720	8.24	10.02	1.7	15.8	1.77
		9/6/2017	17.0	11.20	1582	7.36	0.60	5.4	3.2	1.59
		9/28/2017	1.0	19.70	1796	7.92	9.72	1.6	12.5	NA
		9/28/2017	17.0	11.50	1653	7.37	0.62	3.7	2.6	1.47
		10/16/2017	1.0	18.34	1866	7.84	7.95	1.7	6.2	1.80
		10/16/2017	17.0	11.76	1717	7.28	0.43	4.2	2.2	1.52
Onondaga Lake at Outlet										
L4	43.117151, -76.244931	6/28/2017	3.0	20.97	1592	7.82	7.74	2.5	4.0	2.01
		7/11/2017	3.0	22.99	1442	8.06	8.46	4.6	5.9	2.09
		7/25/2017	3.0	21.34	1530	7.50	3.80	2.4	5.2	1.95
		8/10/2017	3.0	22.65	1500	8.04	9.06	2.5	7.4	2.44
		8/22/2017	3.0	25.02	1572	8.22	10.91	1.4	7.9	NA
		9/6/2017	3.0	20.25	1264	7.92	7.73	3.6	8.0	3.54
		9/28/2017	3.0	18.50	1823	7.55	4.00	1.3	7.8	3.22
		10/16/2017	3.0	18.32	1656	7.69	7.48	3.8	4.7	2.03
Ninemile Creek										
T1	43.080750, -76.225935	6/28/2017	1.0	17.92	696	8.00	12.19	6.6	1.6	1.62
		7/11/2017	1.0	20.98	1330	8.02	9.48	17.9	1.7	1.84
		7/25/2017	1.0	19.23	1261	7.97	9.80	5.9	5.6	1.80
		8/10/2017	1.0	18.48	2309	7.96	13.22	5.7	1.7	0.65
		8/22/2017	1.0	19.64	2021	7.92	10.30	7.2	2.7	0.91
		9/6/2017	1.0	15.52	1774	7.99	10.24	6.0	4.1	0.91
		9/28/2017	1.0	17.50	1474	7.95	11.00	3.7	1.1	1.79
		10/16/2017	1.0	13.70	1978	7.90	10.20	8.9	2.2	0.76
Onondaga Creek										
T2	43.059965, -76.163322	6/28/2017	1.0	17.71	2548	8.01	10.13	25.7	1.9	0.96
		7/11/2017	1.0	19.68	2400	7.99	8.59	85.7	4.1	1.04
		7/25/2017	1.0	17.98	2045	7.88	9.06	49.6	5.1	1.01
		8/10/2017	1.0	18.32	3919	8.03	10.37	8.4	1.5	0.47
		8/22/2017	1.0	19.87	2930	7.87	8.28	79.5	7.8	0.82
		9/6/2017	1.0	16.67	2839	7.92	8.93	63.1	6.8	1.20
		9/28/2017	1.0	16.80	2355	7.90	9.60	5.8	1.8	0.68
		10/16/2017	1.0	14.40	4185	7.86	9.50	21.9	2.1	0.84
Harbor Brook										
T3	43.057910, -76.182777	6/28/2017	1.0	17.11	2284	7.87	10.12	13.3	1.5	0.86
		7/11/2017	1.0	17.94	2165	7.91	9.56	5.9	1.1	0.98
		7/25/2017	1.0	15.59	2280	7.82	9.89	4.9	2.6	1.86
		8/10/2017	1.0	16.44	2409	8.01	10.90	2.5	0.6	0.63
		8/22/2017	1.0	17.30	2336	7.91	9.85	4.2	1.5	0.95
		9/6/2017	1.0	15.82	2254	7.96	9.60	16.5	2.6	1.12
		9/28/2017	1.0	15.60	2458	8.10	11.00	3.6	1.1	0.68
		10/16/2017	1.0	13.80	2193	7.90	10.50	3.0	1.0	1.10
Ley Creek										
T4	43.057910, -76.182777	6/28/2017	1.0	20.73	1277	7.74	9.18	16.2	3.7	3.29
		7/11/2017	1.0	23.62	856	7.63	7.24	23.3	5.9	3.35
		7/25/2017	1.0	19.89	999	7.46	6.07	17.1	12.3	3.51
		8/10/2017	1.0	19.90	2091	7.70	6.71	15.2	6.4	2.04
		8/22/2017	1.0	21.52	913	7.47	5.38	12.5	9.2	3.55
		9/6/2017	1.0	17.99	1166	7.62	7.33	13.8	8.5	1.41
		9/28/2017	1.0	19.60	1192	7.68	7.00	9.5	5.0	3.59
		10/16/2017	1.0	14.50	1054	7.66	7.70	16.9	4.1	2.62
Seneca-Oneida-Oswego Rivers										
R294	43.124006, -76.264286	7/11/2017	1.0	25.31	574	8.33	9.57	9.6	16.8	2.93
		10/16/2017	1.0	19.39	816	7.79	7.59	4.6	7.1	4.52
W266	43.131334, -76.242741	7/11/2017	1.0	25.38	716	8.36	10.16	7.8	17.5	3.02
		10/16/2017	1.0	19.47	874	7.80	8.26	2.7	13.3	3.85
W259	43.150243, -76.234730	7/11/2017	1.0	25.73	708	8.49	10.98	7.0	23.7	2.84
		10/16/2017	1.0	19.47	888	7.76	8.17	2.5	11.4	4.35
R212	43.202939, -76.274370	7/11/2017	1.0	24.90	338	8.13	8.34	1.7	6.7	3.74
		10/16/2017	1.0	19.49	372	8.06	8.68	3.2	6.5	4.37
R10	43.218982, -76.278499	7/11/2017	1.0	25.00	574	8.16	8.49	4.8	8.4	3.27
		10/16/2017	1.0	19.55	770	7.68	7.56	2.1	7.8	4.20
R24	43.230055, -76.295944	7/11/2017	1.0	25.06	578	8.16	8.40	4.6	8.2	2.88
		10/16/2017	1.0	19.52	804	7.64	7.88	0.8	4.0	4.19

**Table S3.** Water quality of samples from the Onondaga Lake-Three Rivers system (continued)										
Site ID	Site Coordinates	Sampling Date	Depth (m)	Temperature (°C)	Specific Cond. (µS/cm)	pH	DO (mg/L)	Turbidity (NTU)	Chlorophyll *a* (µg/L)	DOC (mg-C/L)
R78	43.314649, -76.410616	7/11/2017	1.0	25.00	611	8.05	7.94	3.0	7.4	3.22
		10/16/2017	1.0	19.76	839	7.62	7.27	1.4	6.1	3.87
R99	43.358322, -76.415021	7/11/2017	1.0	24.98	601	8.05	8.09	3.3	6.2	3.19
		10/16/2017	1.0	19.50	845	7.78	8.57	1.5	3.1	3.96
R124	43.396077, -76.459584	7/11/2017	1.0	24.62	592	7.96	7.61	3.3	6.1	3.22
		10/16/2017	1.0	19.59	828	7.81	8.48	2.3	4.4	3.19
G135	43.408510, -76.464358	7/11/2017	1.0	24.59	592	7.93	7.51	3.7	6.1	2.12
		10/16/2017	1.0	19.53	826	7.85	8.65	2.1	3.7	2.88
G143	43.440819, -76.480331	7/11/2017	1.0	24.88	573	7.99	7.81	3.2	7.5	3.28
		10/16/2017	1.0	19.59	813	7.90	8.88	2.2	5.4	3.13
R6	43.459983, -76.500604	7/11/2017	1.0	24.86	567	7.96	8.00	4.1	6.0	3.43
		10/16/2017	1.0	19.31	795	7.97	8.86	2.6	4.4	4.18
Regional WWTP Outfall										
Site ID	Site Coordinates	Sampling Date	Depth (m)	Temperature (°C)	Specific Cond. (µS/cm)	BOD_5 (mg/L)	DO (mg/L)	Turbidity (NTU)	NH_3-N (mg/L)	DOC (mg-C/L)
WWTP	43.064167, -76.178056	6/28/2017	1.0	18.67	1864	3	9.58	2.8	0.634	2.60
		7/11/2017	1.0	20.07	1536	6	9.15	5.8	0.375	2.28
		7/25/2017	1.0	20.60	1662	3	9.34	2.4	0.424	2.54
		8/10/2017	1.0	21.33	2185	3	9.18	3.6	0.271	2.31
		8/22/2017	1.0	22.40	1877	3	8.99	1.8	0.328	2.15
		9/6/2017	1.0	20.98	1527	3	10.33	2.8	0.375	2.28
		9/28/2017	1.0	21.87	1777	3	9.06	2.0	0.624	3.72
		10/16/2017	1.0	19.70	1833	3	9.32	1.9	0.634	2.60

Table S4. Optical properties and PARAFAC components of samples from the Onondaga Lake-Three Rivers system

Site ID	Sampling Date	Depth (m)	a_{254} (m^{-1})	a_{280} (m^{-1})	a_{440} (m^{-1})	*E2:E3*	$S_{275–295}$ (nm^{-1})	$S_{350–400}$ (nm^{-1})	$S_{290–350}$ (nm^{-1})	S_R	$CDOM_{250-450}$ (m^{-1})	$SUVA_{254}$ (L/mg-C•m)	FI	HIX	$\beta:\alpha$
Onondaga Lake at South End															
L1	6/28/2017	3.0	16.47	11.68	0.51	7.52	0.0181	0.0198	0.0193	0.92	508.8	3.95	1.69	0.88	0.70
	7/11/2017	3.0	19.15	13.64	0.55	7.30	0.0178	0.0203	0.0188	0.88	597.1	3.87	1.67	0.89	0.66
	7/25/2017	3.0	18.83	13.36	0.50	7.40	0.0182	0.0205	0.0189	0.89	582.7	4.05	1.74	0.88	0.66
	8/10/2017	3.0	16.84	11.75	0.52	8.02	0.0191	0.0195	0.0198	0.98	507.4	3.95	1.67	0.85	0.71
	8/22/2017	3.0	14.63	10.39	0.67	7.32	0.0197	0.0163	0.0190	1.21	454.5	3.74	1.73	0.81	0.74
	9/6/2017	3.0	17.24	12.10	0.50	7.92	0.0187	0.0195	0.0197	0.96	522.3	4.09	1.73	0.87	0.71
	9/28/2017	3.0	15.13	10.32	0.14	9.81	0.0208	0.0229	0.0218	0.91	424.3	3.29	1.89	0.85	0.78
	10/16/2017	3.0	15.66	10.98	0.39	8.15	0.0190	0.0197	0.0201	0.97	469.4	3.93	1.78	0.87	0.76
Onondaga Lake at South Deep															
L2	6/28/2017	1.0	15.74	11.21	0.54	7.07	0.0179	0.0195	0.0185	0.92	494.0	3.71	1.71	0.88	0.70
	6/28/2017	17.0	14.31	10.21	0.35	7.34	0.0167	0.0216	0.0189	0.77	446.6	3.51	1.76	0.90	0.64
	7/11/2017	1.0	19.96	14.21	0.59	7.21	0.0179	0.0199	0.0185	0.90	626.4	4.08	1.67	0.89	0.66
	7/11/2017	17.0	16.03	11.52	0.52	6.95	0.0165	0.0200	0.0184	0.82	516.0	4.05	1.72	0.90	0.64
	7/25/2017	1.0	18.33	12.99	0.47	7.42	0.0191	0.0197	0.0191	0.97	565.3	4.65	1.66	0.88	0.68
	7/25/2017	2.0	19.21	13.69	0.64	7.13	0.0185	0.0190	0.0186	0.98	601.7	4.17	1.65	0.86	0.67
	7/25/2017	3.0	18.77	13.39	0.58	7.23	0.0186	0.0197	0.0187	0.94	585.4	4.05	1.71	0.87	0.68
	7/25/2017	4.0	18.46	13.11	0.48	7.51	0.0185	0.0205	0.0191	0.91	568.7	3.95	1.71	0.88	0.68
	7/25/2017	5.0	18.44	13.08	0.47	7.53	0.0184	0.0207	0.0191	0.89	567.9	3.94	1.69	0.88	0.66
	7/25/2017	6.0	18.55	13.20	0.55	7.34	0.0182	0.0200	0.0189	0.91	576.8	3.89	1.70	0.88	0.66
	7/25/2017	7.0	18.64	13.51	0.74	6.26	0.0163	0.0179	0.0178	0.92	612.2	4.79	1.80	0.89	0.67
	7/25/2017	8.0	16.77	12.14	0.49	6.80	0.0165	0.0202	0.0181	0.81	540.0	4.61	1.74	0.89	0.66
	7/25/2017	9.0	17.43	12.47	0.41	7.32	0.0171	0.0212	0.0188	0.81	546.6	NA	1.73	0.89	0.66
	7/25/2017	10.0	16.84	12.14	0.57	6.67	0.0168	0.0194	0.0179	0.87	542.5	4.35	1.74	0.89	0.66
	7/25/2017	11.0	15.42	10.95	0.19	7.87	0.0169	0.0239	0.0196	0.71	470.0	NA	1.74	0.92	0.65
	7/25/2017	12.0	15.86	11.43	0.53	6.79	0.0167	0.0195	0.0181	0.86	509.9	4.25	1.74	0.90	0.70
	7/25/2017	13.0	16.62	11.98	0.57	6.82	0.0170	0.0190	0.0181	0.89	532.8	4.40	1.76	0.88	0.65
	7/25/2017	14.0	16.12	11.58	0.44	7.02	0.0166	0.0219	0.0184	0.76	512.4	7.53	1.74	0.90	0.64
	7/25/2017	15.0	16.78	12.00	0.56	6.98	0.0169	0.0197	0.0182	0.86	536.3	4.07	1.72	0.89	0.65
	7/25/2017	16.0	17.40	12.55	0.64	6.88	0.0165	0.0171	0.0183	0.96	564.9	NA	1.70	0.89	0.64
	7/25/2017	17.0	16.17	11.57	0.48	6.99	0.0166	0.0203	0.0183	0.82	514.0	4.11	1.71	0.90	0.65
	7/25/2017	18.0	16.75	12.04	0.46	7.05	0.0162	0.0208	0.0185	0.78	533.1	7.57	1.71	0.90	0.64
	8/10/2017	1.0	16.23	11.19	0.14	9.51	0.0198	0.0236	0.0214	0.84	462.4	NA	1.75	0.88	0.71
	8/10/2017	17.0	16.91	12.17	1.13	5.92	0.0159	0.0148	0.0168	1.07	575.1	4.48	1.71	0.91	0.64
	8/22/2017	1.0	15.17	10.99	0.60	6.82	0.0165	0.0184	0.0182	0.89	490.7	4.39	1.76	0.88	0.65
	8/22/2017	17.0	14.67	10.25	0.47	8.27	0.0199	0.0187	0.0203	1.06	437.3	3.52	1.74	0.84	0.75
	9/6/2017	1.0	16.37	11.50	0.51	7.87	0.0192	0.0188	0.0196	1.02	496.3	4.11	1.70	0.87	0.71
	9/6/2017	17.0	17.35	12.48	0.69	6.50	0.0164	0.0181	0.0176	0.91	565.6	4.16	1.72	0.91	0.62
	9/28/2017	1.0	15.79	10.90	0.43	8.12	0.0195	0.0178	0.0202	1.09	468.3	3.38	1.85	0.85	0.77
	9/28/2017	17.0	16.09	11.39	0.30	7.60	0.0176	0.0222	0.0191	0.79	493.0	4.06	1.75	0.91	0.64
	10/16/2017	1.0	15.58	10.92	0.44	8.11	0.0191	0.0192	0.0200	0.99	468.2	3.69	1.77	0.86	0.76
	10/16/2017	2.0	15.53	10.90	0.44	7.97	0.0190	0.0195	0.0198	0.98	468.2	3.72	1.74	0.85	0.76
	10/16/2017	3.0	15.40	10.79	0.43	8.10	0.0190	0.0194	0.0200	0.98	463.1	3.78	1.76	0.86	0.76
	10/16/2017	4.0	15.49	10.88	0.42	8.10	0.0190	0.0195	0.0200	0.97	466.1	3.85	1.73	0.87	0.75
	10/16/2017	5.0	15.51	10.87	0.42	8.05	0.0190	0.0197	0.0199	0.96	466.4	3.87	1.77	0.86	0.75
	10/16/2017	6.0	15.49	10.89	0.47	7.89	0.0188	0.0191	0.0197	0.98	469.9	3.85	1.73	0.86	0.75
	10/16/2017	7.0	16.15	11.55	1.07	6.28	0.0179	0.0136	0.0175	1.31	532.3	3.98	1.74	0.86	0.75
	10/16/2017	8.0	15.50	10.86	0.40	8.21	0.0190	0.0197	0.0201	0.97	464.2	3.94	1.74	0.86	0.76
	10/16/2017	9.0	15.71	11.08	0.58	7.56	0.0184	0.0179	0.0193	1.03	484.4	4.08	1.75	0.86	0.74

Table S4. Optical properties and PARAFAC components of samples from the Onondaga Lake-Three Rivers system (continued)

Site ID	Sampling Date	Depth (m)	a_{254} (m^{-1})	a_{280} (m^{-1})	a_{440} (m^{-1})	*E2:E3*	$S_{275–295}$ (nm^{-1})	$S_{350–400}$ (nm^{-1})	$S_{290–350}$ (nm^{-1})	S_R	$CDOM_{250-450}$ (m^{-1})	$SUVA_{254}$ (L/mg-C•m)	FI	HIX	$\beta:\alpha$
Onondaga Lake at South End															
L2	10/16/2017	10.0	15.71	11.12	0.42	7.65	0.0179	0.0203	0.0193	0.88	483.0	4.61	1.72	0.89	0.69
	10/16/2017	11.0	16.05	11.41	0.52	7.29	0.0176	0.0194	0.0188	0.91	502.0	4.77	1.75	0.89	0.67
	10/16/2017	12.0	15.86	11.25	0.43	7.55	0.0175	0.0206	0.0191	0.85	490.7	4.72	1.73	0.89	0.66
	10/16/2017	13.0	16.20	11.52	0.48	7.38	0.0174	0.0200	0.0189	0.87	505.6	4.82	1.73	0.88	0.67
	10/16/2017	14.0	16.28	11.56	0.39	7.62	0.0172	0.0214	0.0192	0.80	503.4	4.81	1.72	0.90	0.65
	10/16/2017	15.0	16.77	11.97	0.54	7.15	0.0167	0.0198	0.0186	0.84	530.5	4.95	1.74	0.90	0.63
	10/16/2017	16.0	17.11	12.27	0.72	6.71	0.0164	0.0180	0.0180	0.91	554.1	4.99	1.70	0.90	0.62
	10/16/2017	17.0	16.76	11.95	0.46	7.35	0.0166	0.0208	0.0189	0.80	526.0	4.89	1.70	0.91	0.62
	10/16/2017	18.0	17.06	12.19	0.58	7.05	0.0167	0.0195	0.0184	0.85	542.7	5.08	1.69	0.90	0.63
Onondaga Lake at North Deep															
L3	6/28/2017	1.0	16.41	11.61	0.45	7.72	0.0183	0.0203	0.0195	0.90	502.3	3.83	1.72	0.88	0.69
	6/28/2017	17.0	NA	NA	NA	NA	NA	NA	NA	NA	NA	NA	NA	NA	NA
	7/11/2017	1.0	19.47	13.86	0.53	7.22	0.0181	0.0202	0.0187	0.90	607.2	4.04	1.65	0.88	0.65
	7/11/2017	17.0	16.26	11.65	0.47	7.14	0.0170	0.0206	0.0186	0.82	513.1	4.10	1.70	0.89	0.64
	7/25/2017	1.0	17.88	12.53	0.31	8.06	0.0189	0.0223	0.0198	0.84	535.3	NA	1.70	0.88	0.66
	7/25/2017	17.0	16.01	11.49	0.43	7.14	0.0168	0.0208	0.0186	0.81	505.5	3.99	1.72	0.90	0.63
	8/10/2017	1.0	17.61	12.46	0.94	6.02	0.0175	0.0160	0.0172	1.09	581.1	NA	1.70	0.86	0.70
	8/10/2017	17.0	16.36	11.78	0.75	6.54	0.0160	0.0176	0.0178	0.91	535.9	4.47	1.74	0.92	0.63
	8/22/2017	1.0	12.21	8.51	0.12	9.39	0.0204	0.0237	0.0214	0.86	348.1	1.91	1.82	0.84	0.72
	8/22/2017	17.0	14.21	10.20	0.62	6.91	0.0174	0.0177	0.0183	0.98	454.6	4.26	1.77	0.87	0.68
	9/6/2017	1.0	16.13	11.32	0.44	7.93	0.0197	0.0197	0.0197	1.00	485.3	3.95	1.70	0.87	0.73
	9/6/2017	17.0	17.23	12.33	0.56	7.01	0.0166	0.0196	0.0184	0.85	548.7	4.70	1.71	0.91	0.62
	9/28/2017	1.0	17.21	12.31	0.56	6.96	0.0172	0.0197	0.0184	0.87	543.7	NA	1.74	0.87	0.64
	9/28/2017	17.0	16.57	11.78	0.49	7.24	0.0173	0.0201	0.0186	0.86	518.7	4.89	1.71	0.91	0.63
	10/16/2017	1.0	15.70	10.98	0.41	8.09	0.0193	0.0198	0.0199	0.97	470.1	3.79	1.77	0.87	0.76
	10/16/2017	17.0	16.83	11.97	0.57	7.10	0.0170	0.0195	0.0184	0.87	531.4	4.81	1.70	0.90	0.64
Onondaga Lake at Outlet															
L4	6/28/2017	3.0	17.78	12.60	0.56	7.50	0.0183	0.0196	0.0191	0.93	549.7	3.84	1.73	0.87	0.68
	7/11/2017	3.0	19.23	13.76	0.61	6.99	0.0178	0.0196	0.0184	0.91	607.7	4.00	1.68	0.88	0.66
	7/25/2017	3.0	18.98	13.70	0.57	6.81	0.0166	0.0202	0.0181	0.82	610.1	4.23	1.75	0.90	0.65
	8/10/2017	3.0	21.35	15.15	0.67	7.24	0.0183	0.0196	0.0187	0.94	665.3	3.80	1.67	0.91	0.64
	8/22/2017	3.0	16.22	11.45	0.44	7.33	0.0185	0.0199	0.0194	0.93	497.8	NA	1.74	0.88	0.65
	9/6/2017	3.0	28.93	20.59	0.88	7.07	0.0187	0.0197	0.0184	0.95	905.8	3.55	1.63	0.91	0.62
	9/28/2017	3.0	28.30	20.13	0.93	7.09	0.0188	0.0192	0.0185	0.98	885.7	3.82	1.63	0.91	0.63
	10/16/2017	3.0	18.31	12.88	0.50	7.86	0.0192	0.0197	0.0196	0.97	554.8	3.92	1.68	0.89	0.71
Ninemile Creek															
T1	6/28/2017	1.0	15.67	11.26	0.49	6.86	0.0168	0.0205	0.0181	0.82	499.7	4.20	1.68	0.92	0.61
	7/11/2017	1.0	15.80	11.36	0.52	6.64	0.0176	0.0195	0.0177	0.90	507.1	3.73	1.63	0.90	0.61
	7/25/2017	1.0	17.10	12.24	0.68	6.45	0.0166	0.0182	0.0173	0.91	556.7	4.13	1.69	0.91	0.59
	8/10/2017	1.0	9.23	6.76	0.51	6.06	0.0183	0.0154	0.0171	1.19	309.3	6.17	1.73	0.87	0.66
	8/22/2017	1.0	10.28	7.48	0.37	6.49	0.0157	0.0192	0.0177	0.82	336.3	5.57	1.72	0.90	0.63
	9/6/2017	1.0	21.60	15.74	0.75	6.35	0.0161	0.0197	0.0173	0.82	710.8	4.91	1.67	0.93	0.59
	9/28/2017	1.0	7.86	5.69	0.05	7.34	0.0174	0.0240	0.0188	0.72	245.1	5.24	1.80	0.92	0.63
	10/16/2017	1.0	15.51	11.38	0.52	6.33	0.0160	0.0196	0.0174	0.81	512.0	4.49	1.70	0.92	0.60
Onondaga Creek															
T2	6/28/2017	1.0	11.85	8.62	0.42	6.67	0.0159	0.0197	0.0180	0.81	384.6	5.36	1.74	0.91	0.61
	7/11/2017	1.0	13.74	10.17	0.69	5.71	0.0153	0.0174	0.0164	0.88	472.3	5.74	1.68	0.91	0.60
	7/25/2017	1.0	12.72	9.47	0.80	5.70	0.0144	0.0157	0.0165	0.92	443.9	5.47	1.72	0.93	0.58

Table S4. Optical properties and PARAFAC components of samples from the Onondaga Lake-Three Rivers system (continued)

Site ID	Sampling Date	Depth (m)	a_{254} (m^{-1})	a_{280} (m^{-1})	a_{440} (m^{-1})	$E2:E3$	$S_{275–295}$ (nm^{-1})	$S_{350–400}$ (nm^{-1})	$S_{290–350}$ (nm^{-1})	S_R	$CDOM_{250-450}$ (m^{-1})	$SUVA_{254}$ (L/mg-C•m)	FI	HIX	$\beta:\alpha$
Onondaga Creek															
T2	8/10/2017	1.0	7.04	5.13	0.16	7.14	0.0174	0.0216	0.0188	0.81	223.5	6.51	1.72	0.91	0.64
	8/22/2017	1.0	12.69	9.42	0.51	6.08	0.0152	0.0189	0.0171	0.81	428.7	6.72	1.75	0.91	0.60
	9/6/2017	1.0	17.40	12.80	0.65	6.06	0.0157	0.0193	0.0170	0.82	583.3	6.29	1.73	0.93	0.58
	9/28/2017	1.0	9.09	6.67	0.13	6.66	0.0161	0.0227	0.0179	0.71	292.6	5.81	1.80	0.93	0.61
	10/16/2017	1.0	13.87	10.38	0.68	5.54	0.0150	0.0174	0.0162	0.86	483.3	7.17	1.74	0.91	0.62
Harbor Brook															
T3	6/28/2017	1.0	5.74	4.07	0.09	8.24	0.0170	0.0232	0.0202	0.73	174.5	2.90	1.83	0.88	0.73
	7/11/2017	1.0	6.80	4.90	0.12	7.14	0.0168	0.0225	0.0186	0.75	215.2	3.01	1.74	0.88	0.69
	7/25/2017	1.0	10.22	7.11	0.37	8.02	0.0181	0.0188	0.0196	0.96	311.1	2.39	1.74	0.72	0.70
	8/10/2017	1.0	5.12	3.77	0.29	6.30	0.0158	0.0149	0.0178	1.06	173.5	3.52	1.85	0.81	0.75
	8/22/2017	1.0	6.60	4.62	0.01	7.95	0.0178	0.0266	0.0195	0.67	197.0	3.01	1.72	0.87	0.70
	9/6/2017	1.0	7.87	5.60	0.12	7.41	0.0175	0.0227	0.0189	0.77	243.8	3.05	1.76	0.89	0.69
	9/28/2017	1.0	5.44	4.09	0.21	6.15	0.0151	0.0201	0.0169	0.75	181.9	3.47	1.91	0.77	0.72
	10/16/2017	1.0	10.96	7.95	0.39	6.40	0.0152	0.0187	0.0175	0.81	360.6	4.33	1.71	0.85	0.68
Ley Creek															
T4	6/28/2017	1.0	33.62	23.57	1.19	6.68	0.0164	0.0198	0.0174	0.82	1075.2	4.44	1.61	0.91	0.64
	7/11/2017	1.0	33.83	23.67	1.79	5.97	0.0160	0.0183	0.0155	0.88	1134.1	4.39	1.59	0.91	0.61
	7/25/2017	1.0	39.63	28.17	1.63	6.34	0.0157	0.0193	0.0170	0.81	1299.5	4.90	1.63	0.92	0.61
	8/10/2017	1.0	23.64	16.83	0.87	6.49	0.0165	0.0187	0.0174	0.88	764.3	5.03	1.65	0.92	0.64
	8/22/2017	1.0	34.75	24.46	1.47	6.48	0.0159	0.0187	0.0172	0.85	1127.2	4.25	1.63	0.91	0.65
	9/6/2017	1.0	36.76	25.81	1.42	6.42	0.0162	0.0192	0.0169	0.84	1188.9	11.32	1.68	0.89	0.67
	9/28/2017	1.0	17.58	12.45	0.40	6.84	0.0170	0.0216	0.0178	0.79	555.1	2.13	1.70	0.91	0.65
	10/16/2017	1.0	28.45	20.30	1.18	6.21	0.0156	0.0187	0.0168	0.83	939.0	4.72	1.65	0.90	0.67
Seneca-Oneida-Oswego Rivers															
R294	7/11/2017	1.0	18.25	13.03	0.46	7.28	0.0186	0.0205	0.0187	0.91	566.3	2.70	1.65	0.90	0.59
W266	7/11/2017	1.0	18.26	12.96	0.31	7.77	0.0189	0.0225	0.0193	0.84	554.0	2.63	1.67	0.89	0.60
W259	7/11/2017	1.0	19.50	13.77	0.94	6.47	0.0202	0.0158	0.0173	1.28	626.5	2.98	1.62	0.89	0.59
R212	7/11/2017	1.0	26.94	19.36	0.75	6.90	0.0180	0.0204	0.0182	0.88	853.6	3.13	1.65	0.91	0.55
R10	7/11/2017	1.0	23.20	16.52	0.73	6.89	0.0186	0.0191	0.0182	0.97	729.0	3.08	1.60	0.86	0.55
R24	7/11/2017	1.0	19.12	13.78	0.70	6.70	0.0177	0.0175	0.0181	1.01	614.7	2.88	1.64	0.90	0.60
R78	7/11/2017	1.0	21.22	15.18	0.46	7.34	0.0184	0.0214	0.0188	0.86	657.2	2.86	1.62	0.90	0.59
R99	7/11/2017	1.0	20.82	14.86	0.42	7.45	0.0184	0.0217	0.0190	0.85	641.8	2.83	1.64	0.91	0.58
R124	7/11/2017	1.0	21.90	15.67	0.59	7.13	0.0183	0.0205	0.0185	0.89	685.4	2.95	1.65	0.90	0.59
G135	7/11/2017	1.0	18.95	14.09	2.03	4.63	0.0171	0.0112	0.0140	1.54	703.1	3.88	1.59	0.80	0.55
G143	7/11/2017	1.0	21.08	15.03	0.43	7.48	0.0186	0.0217	0.0190	0.86	648.6	2.79	1.70	0.91	0.61
R6	7/11/2017	1.0	22.50	16.09	0.57	7.21	0.0183	0.0207	0.0187	0.89	702.4	2.85	1.64	0.90	0.58
R294	10/16/2017	1.0	31.62	22.24	1.15	7.06	0.0189	0.0182	0.0183	1.04	989.6	3.04	1.63	0.91	0.61
W266	10/16/2017	1.0	30.54	21.68	0.95	7.14	0.0190	0.0195	0.0185	0.97	952.1	3.44	1.60	0.92	0.63
W259	10/16/2017	1.0	30.35	21.52	0.85	7.32	0.0189	0.0199	0.0188	0.95	939.3	3.03	1.65	0.92	0.63
R212	10/16/2017	1.0	28.50	19.84	0.56	8.27	0.0205	0.0218	0.0199	0.94	841.5	2.83	1.63	0.91	0.61
R10	10/16/2017	1.0	28.22	19.84	0.66	7.74	0.0196	0.0208	0.0193	0.94	854.2	2.92	1.60	0.90	0.63
R24	10/16/2017	1.0	28.96	20.49	0.79	7.49	0.0193	0.0201	0.0190	0.96	889.0	3.00	1.65	0.91	0.62
R78	10/16/2017	1.0	28.05	19.84	0.84	7.38	0.0194	0.0197	0.0188	0.99	863.7	3.15	1.65	0.91	0.63
R99	10/16/2017	1.0	27.27	19.22	0.62	7.80	0.0196	0.0210	0.0194	0.93	824.4	2.99	1.64	0.91	0.63
R124	10/16/2017	1.0	27.81	19.68	0.81	7.45	0.0196	0.0199	0.0190	0.98	853.2	3.79	1.65	0.91	0.64
G135	10/16/2017	1.0	28.03	19.83	0.82	7.43	0.0196	0.0198	0.0189	0.99	860.6	4.23	1.62	0.91	0.63
G143	10/16/2017	1.0	28.31	20.07	0.91	7.33	0.0195	0.0194	0.0188	1.00	874.4	3.93	1.63	0.90	0.63
R6	10/16/2017	1.0	28.54	20.21	0.82	7.48	0.0193	0.0198	0.0190	0.97	876.6	2.96	1.63	0.90	0.63

Table S4. Optical properties and PARAFAC components of samples from the Onondaga Lake-Three Rivers system (continued)

Site ID	Sampling Date	Depth (m)	a_{254} (m^{-1})	a_{280} (m^{-1})	a_{440} (m^{-1})	*E2:E3*	$S_{275–295}$ (nm^{-1})	$S_{350–400}$ (nm^{-1})	$S_{290–350}$ (nm^{-1})	S_R	$CDOM_{250-450}$ (m^{-1})	$SUVA_{254}$ (L/mg-C•m)	FI	HIX	$\beta:\alpha$
Regional WWTP Outfall															
WWTP	6/28/2017	1.0	14.58	10.43	0.40	7.99	0.0141	0.0209	0.0200	0.68	457.4	2.43	2.17	0.78	0.71
	7/11/2017	1.0	17.22	12.39	0.52	7.40	0.0134	0.0208	0.0190	0.64	554.1	3.28	2.25	0.83	0.64
	7/25/2017	1.0	16.03	11.32	0.25	8.62	0.0142	0.0240	0.0205	0.59	490.0	2.74	2.17	0.84	0.72
	8/10/2017	1.0	14.61	10.93	0.48	7.07	0.0120	0.0201	0.0191	0.60	489.0	2.74	2.33	0.83	0.68
	8/22/2017	1.0	9.04	6.59	0.18	7.41	0.0143	0.0223	0.0191	0.64	291.2	1.83	2.32	0.81	0.64
	9/6/2017	1.0	24.06	18.27	0.95	6.48	0.0119	0.0197	0.0175	0.60	831.0	4.58	2.19	0.82	0.71
	9/28/2017	1.0	22.46	16.76	0.66	7.24	0.0125	0.0211	0.0193	0.59	744.7	2.62	2.16	0.83	0.72
	10/16/2017	1.0	21.77	16.07	0.74	6.65	0.0122	0.0203	0.0182	0.60	732.2	2.70	2.12	0.84	0.72
Site ID	Sampling Date	Depth (m)	Peak A (R.U.)	Peak B (R.U.)	Peak C (R.U.)	Peak M (R.U.)	Peak T (R.U.)	Peak A : Peak T	Peak C : Peak A	Peak C : Peak T	FDOM (R.U.)	C1 (R.U.)	C2 (R.U.)	C3 (R.U.)	C4 (R.U.)
Onondaga Lake at South End															
L1	6/28/2017	3.0	2.09	0.23	1.79	1.65	0.62	3.40	0.86	2.91	40265	1.67	1.31	0.81	0.49
	7/11/2017	3.0	2.41	0.20	2.02	1.87	0.63	3.82	0.84	3.20	45799	1.88	1.55	0.89	0.48
	7/25/2017	3.0	2.40	0.23	2.04	1.88	0.67	3.61	0.85	3.06	46034	1.86	1.55	0.93	0.51
	8/10/2017	3.0	2.03	0.26	1.71	1.60	0.68	2.96	0.84	2.50	38697	1.61	1.27	0.73	0.58
	8/22/2017	3.0	1.58	0.44	1.36	1.29	0.87	1.82	0.86	1.57	31132	1.29	0.95	0.60	0.80
	9/6/2017	3.0	2.30	0.32	1.92	1.84	0.77	2.97	0.84	2.48	44122	1.86	1.40	0.86	0.68
	9/28/2017	3.0	1.82	0.39	1.48	1.40	0.70	2.62	0.81	2.13	34344	1.41	1.08	0.75	0.66
	10/12/2017	3.0	1.93	0.30	1.70	1.62	0.66	2.94	0.88	2.60	38138	1.58	1.21	0.78	0.58
Onondaga Lake at South Deep															
L2	6/28/2017	1.0	2.03	0.25	1.73	1.60	0.58	3.52	0.85	2.99	39043	1.61	1.27	0.80	0.47
	6/28/2017	17.0	2.30	0.22	2.02	1.81	0.52	4.46	0.88	3.91	45023	1.75	1.53	1.02	0.37
	7/11/2017	1.0	2.44	0.21	2.03	1.88	0.69	3.53	0.83	2.93	47142	1.88	1.62	0.92	0.53
	7/11/2017	17.0	2.47	0.15	2.16	1.94	0.57	4.31	0.88	3.78	48586	1.89	1.66	1.08	0.39
	7/25/2017	1.0	2.27	0.23	1.96	1.81	0.67	3.38	0.86	2.92	44076	1.78	1.48	0.88	0.53
	7/25/2017	2.0	2.27	0.35	1.93	1.79	0.82	2.77	0.85	2.36	44127	1.78	1.47	0.86	0.68
	7/25/2017	3.0	2.23	0.29	1.89	1.77	0.75	2.98	0.85	2.52	43094	1.74	1.43	0.85	0.62
	7/25/2017	4.0	2.22	0.21	1.90	1.76	0.63	3.53	0.86	3.02	42662	1.74	1.44	0.84	0.49
	7/25/2017	5.0	2.24	0.18	1.93	1.77	0.69	3.26	0.86	2.81	43548	1.76	1.46	0.86	0.53
	7/25/2017	6.0	2.25	0.20	1.92	1.78	0.62	3.64	0.85	3.10	43353	1.76	1.46	0.87	0.49
	7/25/2017	7.0	2.68	0.31	2.42	2.17	0.80	3.34	0.91	3.03	55026	2.11	1.77	1.37	0.65
	7/25/2017	8.0	2.40	0.23	2.16	1.93	0.66	3.63	0.90	3.26	48955	1.85	1.63	1.19	0.49
	7/25/2017	9.0	2.41	0.29	2.07	1.89	0.70	3.44	0.86	2.96	48340	1.83	1.65	1.06	0.55
	7/25/2017	10.0	2.37	0.21	2.06	1.87	0.61	3.88	0.87	3.37	47091	1.81	1.61	1.05	0.46
	7/25/2017	11.0	2.31	0.13	1.98	1.79	0.44	5.28	0.86	4.52	45473	1.74	1.58	1.01	0.31
	7/25/2017	12.0	2.59	0.20	2.27	2.10	0.65	3.96	0.88	3.47	51086	2.09	1.64	1.15	0.49
	7/25/2017	13.0	2.45	0.30	2.11	1.92	0.80	3.06	0.86	2.64	48313	1.87	1.62	1.07	0.61
	7/25/2017	14.0	2.44	0.19	2.14	1.93	0.57	4.28	0.88	3.75	47866	1.87	1.63	1.07	0.43
	7/25/2017	15.0	2.49	0.21	2.16	1.96	0.69	3.62	0.87	3.13	49100	1.90	1.68	1.10	0.50
	7/25/2017	16.0	2.45	0.34	2.11	1.90	0.77	3.18	0.86	2.73	48988	1.94	1.68	1.10	0.43
	7/25/2017	17.0	2.52	0.20	2.22	2.02	0.62	4.04	0.88	3.56	49451	1.97	1.72	1.12	0.42
	7/25/2017	18.0	2.55	0.20	2.28	2.06	0.60	4.21	0.90	3.78	50409	2.09	1.64	1.15	0.49
	8/10/2017	1.0	1.95	0.23	1.62	1.55	0.56	3.46	0.83	2.88	36685	1.55	1.20	0.68	0.48
	8/10/2017	17.0	2.53	0.18	2.21	1.98	0.55	4.58	0.87	4.00	49366	1.90	1.72	1.08	0.37
	8/22/2017	1.0	2.22	0.41	1.95	1.79	0.76	2.93	0.88	2.58	44259	1.71	1.46	0.99	0.62
	8/22/2017	17.0	1.71	0.40	1.43	1.37	0.79	2.16	0.84	1.81	32993	1.37	1.03	0.64	0.72
	9/6/2017	1.0	1.93	0.30	1.66	1.55	0.68	2.85	0.86	2.45	37326	1.55	1.21	0.73	0.59
	9/6/2017	17.0	2.60	0.20	2.25	2.01	0.63	4.13	0.87	3.58	50367	1.97	1.73	1.12	0.42

Table S4. Optical properties and PARAFAC components of samples from the Onondaga Lake-Three Rivers system (continued)

Site ID	Sampling Date	Depth (m)	Peak A (R.U.)	Peak B (R.U.)	Peak C (R.U.)	Peak M (R.U.)	Peak T (R.U.)	Peak A : Peak T	Peak C : Peak A	Peak C : Peak T	FDOM (R.U.)	C1 (R.U.)	C2 (R.U.)	C3 (R.U.)	C4 (R.U.)
Onondaga Lake at South Deep															
L2	9/28/2017	1.0	1.98	0.37	1.58	1.48	0.66	3.00	0.80	2.39	37181	1.47	1.20	0.83	0.63
	9/28/2017	17.0	2.43	0.20	2.09	1.89	0.49	4.92	0.86	4.23	47649	1.83	1.64	1.06	0.38
	10/12/2017	1.0	1.88	0.27	1.65	1.57	0.62	3.02	0.88	2.64	36953	1.55	1.17	0.74	0.57
	10/12/2017	2.0	1.88	0.31	1.67	1.58	0.65	2.92	0.89	2.59	37180	1.55	1.18	0.75	0.59
	10/12/2017	3.0	1.86	0.26	1.65	1.56	0.61	3.06	0.89	2.71	36896	1.54	1.17	0.74	0.54
	10/12/2017	4.0	1.84	0.29	1.65	1.56	0.67	2.77	0.89	2.47	37032	1.54	1.18	0.75	0.57
	10/12/2017	5.0	1.89	0.29	1.68	1.59	0.65	2.92	0.89	2.60	37208	1.55	1.17	0.76	0.56
	10/12/2017	6.0	1.89	0.27	1.65	1.56	0.64	2.96	0.87	2.58	37016	1.54	1.17	0.75	0.55
	10/12/2017	7.0	1.91	0.37	1.65	1.58	0.67	2.86	0.86	2.47	37511	1.56	1.18	0.76	0.62
	10/12/2017	8.0	1.88	0.29	1.64	1.60	0.66	2.85	0.87	2.49	37226	1.55	1.18	0.75	0.57
	10/12/2017	9.0	1.93	0.30	1.71	1.61	0.64	3.03	0.89	2.68	38652	1.59	1.23	0.79	0.58
	10/12/2017	10.0	2.17	0.23	1.91	1.79	0.57	3.78	0.88	3.32	42917	1.71	1.42	0.93	0.47
	10/12/2017	11.0	2.27	0.23	1.99	1.83	0.61	3.74	0.87	3.27	44752	1.77	1.50	0.97	0.48
	10/12/2017	12.0	2.31	0.24	2.02	1.84	0.59	3.94	0.87	3.44	45319	1.79	1.53	0.99	0.45
	10/12/2017	13.0	2.37	0.23	2.04	1.89	0.66	3.59	0.86	3.09	46562	1.82	1.57	1.01	0.52
	10/12/2017	14.0	2.44	0.20	2.12	1.94	0.58	4.22	0.87	3.67	47790	1.86	1.64	1.04	0.43
	10/12/2017	15.0	2.57	0.20	2.21	2.01	0.62	4.17	0.86	3.60	50145	1.93	1.73	1.10	0.44
	10/12/2017	16.0	2.59	0.22	2.23	2.03	0.65	4.01	0.86	3.46	50574	1.95	1.75	1.11	0.45
	10/12/2017	17.0	2.57	0.20	2.23	2.03	0.59	4.38	0.87	3.79	50488	1.95	1.75	1.11	0.41
	10/12/2017	18.0	2.57	0.22	2.26	2.03	0.63	4.07	0.88	3.58	50781	1.95	1.75	1.12	0.45
Onondaga Lake at North Deep															
L3	6/28/2017	1.0	2.07	0.24	1.78	1.66	0.62	3.36	0.86	2.89	39918	1.66	1.31	0.79	0.48
	6/28/2017	17.0	NA	NA	NA	NA	NA	NA	NA	NA	NA	NA	NA	NA	NA
	7/11/2017	1.0	2.32	0.23	1.96	1.83	0.67	3.47	0.84	2.93	44824	1.81	1.52	0.88	0.52
	7/11/2017	17.0	2.41	0.17	2.09	1.88	0.61	3.96	0.87	3.44	46683	1.84	1.59	1.02	0.43
	7/25/2017	1.0	2.17	0.43	1.83	1.70	0.63	3.43	0.84	2.90	41862	1.69	1.42	0.80	0.55
	7/25/2017	17.0	2.44	0.18	2.13	1.89	0.61	3.98	0.88	3.48	47479	1.85	1.62	1.06	0.44
	8/10/2017	1.0	2.02	0.38	1.74	1.62	0.71	2.84	0.86	2.45	39966	1.63	1.31	0.75	0.68
	8/10/2017	17.0	2.51	0.15	2.16	1.98	0.54	4.65	0.86	4.00	48756	1.89	1.69	1.07	0.36
	8/22/2017	1.0	1.48	0.69	1.22	1.16	0.58	2.54	0.83	2.10	28546	1.17	0.90	0.55	0.63
	8/22/2017	17.0	2.01	0.33	1.75	1.60	0.66	3.03	0.87	2.63	39536	1.57	1.28	0.87	0.59
	9/6/2017	1.0	1.88	0.25	1.58	1.49	0.61	3.08	0.84	2.58	35919	1.50	1.17	0.69	0.53
	9/6/2017	17.0	2.59	0.22	2.25	2.02	0.62	4.18	0.87	3.63	51194	1.95	1.79	1.11	0.44
	9/28/2017	1.0	2.54	0.62	2.24	2.05	0.96	2.64	0.88	2.33	51010	1.94	1.71	1.11	0.82
	9/28/2017	17.0	2.49	0.20	2.12	1.94	0.59	4.24	0.85	3.61	48446	1.87	1.69	1.07	0.42
	10/12/2017	1.0	1.95	0.30	1.66	1.60	0.66	2.96	0.85	2.52	37661	1.56	1.20	0.76	0.57
	10/12/2017	17.0	2.53	0.22	2.17	1.97	0.61	4.16	0.86	3.57	49161	1.91	1.70	1.07	0.44
Onondaga Lake at Outlet															
L4	6/28/2017	3.0	2.32	0.31	2.01	1.86	0.73	3.15	0.87	2.74	44911	1.85	1.46	0.92	0.59
	7/11/2017	3.0	2.34	0.20	1.99	1.85	0.67	3.51	0.85	2.98	45341	1.82	1.54	0.90	0.51
	7/25/2017	3.0	2.61	0.18	2.34	2.07	0.62	4.18	0.89	3.74	52503	2.01	1.77	1.26	0.44
	8/10/2017	3.0	2.60	0.18	2.20	2.01	0.58	4.45	0.84	3.76	50070	1.95	1.79	1.00	0.39
	8/22/2017	3.0	1.90	0.24	1.59	1.47	0.50	3.83	0.84	3.21	36518	1.44	1.27	0.70	0.43
	9/6/2017	3.0	3.49	0.15	2.97	2.73	0.74	4.71	0.85	4.01	67463	2.62	2.44	1.33	0.48
	9/28/2017	3.0	3.40	0.18	2.86	2.61	0.77	4.42	0.84	3.72	65502	2.54	2.36	1.28	0.52
	10/12/2017	3.0	2.22	0.28	1.90	1.79	0.66	3.34	0.86	2.86	43311	1.75	1.45	0.86	0.55

Table S4. Optical properties and PARAFAC components of samples from the Onondaga Lake-Three Rivers system (continued)

Site ID	Sampling Date	Depth (m)	Peak A (R.U.)	Peak B (R.U.)	Peak C (R.U.)	Peak M (R.U.)	Peak T (R.U.)	Peak A : Peak T	Peak C : Peak A	Peak C : Peak T	FDOM (R.U.)	C1 (R.U.)	C2 (R.U.)	C3 (R.U.)	C4 (R.U.)
Ninemile Creek															
T1	6/28/2017	1.0	2.44	0.05	2.09	1.86	0.44	5.57	0.86	4.78	47059	1.82	1.68	1.00	0.24
	7/11/2017	1.0	2.10	0.10	1.80	1.61	0.48	4.41	0.86	3.79	40932	1.54	1.46	0.88	0.32
	7/25/2017	1.0	2.46	0.09	2.13	1.89	0.41	6.07	0.86	5.24	49317	1.78	1.81	1.08	0.24
	8/10/2017	1.0	1.22	0.24	1.10	0.99	0.57	2.14	0.90	1.93	24765	0.93	0.81	0.56	0.45
	8/22/2017	1.0	1.61	0.15	1.39	1.28	0.37	4.34	0.86	3.75	31927	1.26	1.08	0.68	0.28
	9/6/2017	1.0	3.16	0.10	2.70	2.39	0.59	5.36	0.86	4.58	62627	2.30	2.25	1.42	0.35
	9/28/2017	1.0	1.11	0.07	1.01	0.91	0.21	5.31	0.91	4.83	22320	0.85	0.76	0.53	0.15
	10/12/2017	1.0	2.27	0.13	1.96	1.73	0.50	4.58	0.86	3.95	44868	1.66	1.59	1.02	0.32
Onondaga Creek															
T2	6/28/2017	1.0	1.81	0.08	1.59	1.41	0.33	5.40	0.88	4.76	34907	1.34	1.22	0.79	0.21
	7/11/2017	1.0	2.03	0.06	1.76	1.54	0.39	5.20	0.87	4.53	39718	1.45	1.41	0.95	0.23
	7/25/2017	1.0	2.07	0.08	1.81	1.60	0.32	6.47	0.88	5.67	41634	1.48	1.49	1.01	0.16
	8/10/2017	1.0	1.12	0.08	1.02	0.92	0.20	5.48	0.91	4.97	22453	0.86	0.76	0.54	0.15
	8/22/2017	1.0	1.88	0.10	1.67	1.47	0.35	5.37	0.88	4.75	38225	1.37	1.34	0.95	0.22
	9/6/2017	1.0	2.49	0.08	2.21	1.93	0.46	5.47	0.89	4.85	50033	1.82	1.77	1.23	0.26
	9/28/2017	1.0	1.27	0.08	1.12	1.01	0.22	5.71	0.89	5.06	25425	0.92	0.89	0.62	0.14
	10/12/2017	1.0	1.91	0.10	1.70	1.49	0.52	3.67	0.89	3.27	39198	1.40	1.36	0.96	0.35
Harbor Brook															
T3	6/28/2017	1.0	1.22	0.15	1.00	0.93	0.34	3.63	0.82	2.96	22872	0.99	0.70	0.47	0.29
	7/11/2017	1.0	1.34	0.07	1.11	1.01	0.30	4.41	0.83	3.67	25265	1.05	0.81	0.54	0.24
	7/25/2017	1.0	2.06	3.63	1.66	1.63	1.46	1.41	0.80	1.13	42202	1.66	1.15	0.68	2.00
	8/10/2017	1.0	0.93	0.55	0.81	0.77	0.43	2.13	0.87	1.87	18953	0.77	0.54	0.41	0.48
	8/22/2017	1.0	1.41	0.20	1.13	1.07	0.43	3.25	0.80	2.61	25953	1.15	0.78	0.50	0.38
	9/6/2017	1.0	1.58	0.16	1.27	1.20	0.45	3.47	0.81	2.81	29344	1.27	0.92	0.59	0.38
	9/28/2017	1.0	0.95	0.66	0.84	0.74	0.66	1.43	0.88	1.27	19833	0.72	0.58	0.48	0.67
	10/12/2017	1.0	2.03	0.59	1.58	1.53	0.81	2.51	0.78	1.95	39660	1.62	1.26	0.71	0.78
Ley Creek															
T4	6/28/2017	1.0	6.88	0.35	5.14	4.91	1.50	4.58	0.75	3.42	124032	5.27	4.32	1.97	1.11
	7/11/2017	1.0	6.40	0.29	4.74	4.46	1.53	4.19	0.74	3.11	118229	4.76	4.25	1.87	1.10
	7/25/2017	1.0	8.19	0.31	6.10	5.75	1.55	5.28	0.75	3.94	151204	6.12	5.44	2.49	1.05
	8/10/2017	1.0	4.37	0.23	3.51	3.26	0.95	4.59	0.80	3.69	82435	3.31	2.87	1.58	0.67
	8/22/2017	1.0	7.51	0.47	5.44	5.52	1.78	4.21	0.72	3.05	136122	5.87	4.65	2.02	1.42
	9/6/2017	1.0	7.37	0.54	5.52	5.46	1.91	3.87	0.75	2.90	136194	5.80	4.58	2.20	1.58
	9/28/2017	1.0	3.12	0.27	2.52	2.33	0.73	4.28	0.81	3.44	59646	2.36	2.05	1.18	0.57
	10/12/2017	1.0	5.88	0.43	4.23	4.26	1.49	3.96	0.72	2.85	107833	4.55	3.67	1.68	1.24
Seneca-Oneida-Oswego Rivers															
R294	7/11/2017	1.0	2.14	0.11	1.89	1.69	0.40	5.34	0.89	4.73	42265	1.58	1.54	0.87	0.27
W266	7/11/2017	1.0	2.23	0.15	1.95	1.78	0.45	4.95	0.87	4.33	43306	1.66	1.54	0.92	0.31
W259	7/11/2017	1.0	2.09	0.19	1.86	1.68	0.52	4.02	0.89	3.57	41621	1.58	1.49	0.83	0.38
R212	7/11/2017	1.0	2.89	0.09	2.46	2.22	0.51	5.65	0.85	4.82	54776	2.09	2.06	1.06	0.30
R10	7/11/2017	1.0	2.58	0.48	2.37	2.41	0.77	3.35	0.92	3.08	53372	1.91	1.92	1.13	0.63
R24	7/11/2017	1.0	2.15	0.14	1.88	1.71	0.42	5.10	0.87	4.45	41808	1.59	1.52	0.85	0.32
R78	7/11/2017	1.0	2.39	0.14	2.08	1.89	0.54	4.39	0.87	3.83	46501	1.78	1.68	0.95	0.37
R99	7/11/2017	1.0	2.39	0.12	2.09	1.91	0.49	4.92	0.87	4.30	46131	1.78	1.67	0.94	0.31
R124	7/11/2017	1.0	2.49	0.13	2.18	1.98	0.48	5.23	0.88	4.58	48571	1.84	1.78	0.99	0.31
G135	7/11/2017	1.0	1.76	0.61	1.67	1.35	0.98	1.80	0.95	1.71	36666	1.28	1.26	0.76	0.92
G143	7/11/2017	1.0	2.45	0.12	2.16	1.96	0.45	5.41	0.88	4.78	47343	1.84	1.69	1.00	0.30
R6	7/11/2017	1.0	2.57	0.16	2.26	2.03	0.57	4.51	0.88	3.96	50352	1.91	1.83	1.01	0.41

Table S4. Optical properties and PARAFAC components of samples from the Onondaga Lake-Three Rivers system (continued)

Site ID	Sampling Date	Depth (m)	Peak A (R.U.)	Peak B (R.U.)	Peak C (R.U.)	Peak M (R.U.)	Peak T (R.U.)	Peak A : Peak T	Peak C : Peak A	Peak C : Peak T	FDOM (R.U.)	C1 (R.U.)	C2 (R.U.)	C3 (R.U.)	C4 (R.U.)
Seneca-Oneida-Oswego Rivers															
R294	10/16/2017	1.0	3.62	0.15	3.07	2.81	0.68	5.31	0.85	4.49	70766	2.69	2.61	1.35	0.44
W266	10/16/2017	1.0	3.61	0.13	3.04	2.80	0.74	4.89	0.84	4.12	69644	2.70	2.53	1.35	0.46
W259	10/16/2017	1.0	3.49	0.15	2.98	2.73	0.66	5.32	0.86	4.55	68386	2.63	2.49	1.33	0.42
R212	10/16/2017	1.0	2.85	0.12	2.41	2.24	0.61	4.69	0.85	3.97	53845	2.15	1.96	0.98	0.41
R10	10/16/2017	1.0	3.12	0.15	2.63	2.40	0.64	4.90	0.84	4.13	59962	2.33	2.17	1.15	0.44
R24	10/16/2017	1.0	3.26	0.17	2.77	2.53	0.69	4.73	0.85	4.02	62767	2.42	2.28	1.22	0.47
R78	10/16/2017	1.0	3.18	0.15	2.71	2.51	0.72	4.38	0.86	3.75	61117	2.42	2.18	1.18	0.48
R99	10/16/2017	1.0	3.08	0.15	2.63	2.40	0.62	4.98	0.85	4.24	59108	2.32	2.13	1.13	0.42
R124	10/16/2017	1.0	3.18	0.16	2.68	2.49	0.74	4.30	0.84	3.62	60336	2.40	2.15	1.16	0.49
G135	10/16/2017	1.0	3.12	0.20	2.67	2.47	0.76	4.09	0.86	3.50	60279	2.39	2.15	1.16	0.52
G143	10/16/2017	1.0	3.18	0.17	2.70	2.49	0.77	4.10	0.85	3.48	61123	2.43	2.17	1.17	0.54
R6	10/16/2017	1.0	3.14	0.17	2.68	2.50	0.67	4.71	0.86	4.03	61008	2.39	2.19	1.17	0.47
Regional WWTP Outfall															
WWTP	6/28/2017	1.0	2.75	1.03	3.42	2.60	1.98	1.39	1.24	1.73	65546	2.56	1.49	2.31	1.80
	7/11/2017	1.0	3.46	1.00	5.15	3.41	1.86	1.86	1.49	2.77	86132	3.19	1.95	3.73	1.65
	7/25/2017	1.0	3.23	0.82	3.76	3.01	1.44	2.24	1.17	2.61	74059	3.00	1.80	2.49	1.36
	8/10/2017	1.0	2.34	0.80	3.82	2.55	1.32	1.77	1.63	2.88	63573	2.24	1.41	2.85	1.20
	8/22/2017	1.0	1.68	0.64	2.71	1.70	0.87	1.93	1.61	3.11	44450	1.55	1.01	2.02	0.83
	9/6/2017	1.0	3.81	1.56	5.66	4.02	2.16	1.77	1.48	2.62	104270	3.47	2.52	4.27	2.00
	9/28/2017	1.0	3.46	1.19	5.19	3.78	1.87	1.85	1.50	2.78	94505	3.30	2.20	3.83	1.70
	10/16/2017	1.0	3.99	0.93	5.05	3.83	1.84	2.17	1.27	2.75	97477	3.68	2.40	3.50	1.69

S4. Screening and quantification of OMPs by SPE-LC-HRMS

**Table S5.** LC-HRMS instrument settings		
Dionex UltiMate 3000 High-Performance Liquid Chromatograph		
Time (min)	Mobile Phase A (H_2O + 0.1% FA)	Mobile Phase B (MeOH + 0.1% FA)
0	90%	10%
1	90%	10%
8	50%	50%
21	5%	95%
29	5%	95%
29.5	90%	10%
36	90%	10%
Thermo Scientific LTQ XL Hybrid Ion Trap-Orbitrap High-Resolution Mass Spectrometer		
	Positive ESI	Negative ESI
	Ion Source	
Source Voltage (V)	3,900 (+)	3,500 (-)
Capillary Temperature (°C)	270.00	310.00
Capillary Voltage (V)	38.00 (+)	35.00 (-)
Tube Lens (V)	95.00 (+)	85.00 (-)
Sheath Gas Flow (arb)	25.00	25.00
Auxiliary Gas Flow (arb)	5.00	3.00
Sweep Gas Flow (arb)	0.00	0.00
	ITMS (Linear Ion Trap MS)	
Full MS Scan Range (*m/z*)	100.0-1000.0	100.0-1000.0
Full MS AGC Target	1,000,000	1,000,000
Full MS Maximum Injection Time (ms)	100	100
dd-MS2 Isolation Width	2.0	2.0
HCD dd-MS2 AGC Target	100,000	100,000
HCD dd-MS2 Maximum Injection Time (ms)	250.00	250.00
HCD dd-MS2 Normalized Collision Energy (%)	30, 45, 60	30, 45, 60
dd-MS2 Dynamic Exclusion (s)	4.0	4.0
	FTMS (Orbitrap MS)	
Full MS Scan Range (*m/z*)	100.0-1000.0	100.0-1000.0
Full MS Scan Resolution (at *m/z* 400)	60,000	60,000
dd-MS2 Scan Resolution (at *m/z* 400)	7,500	7,500

**Table S6.** *TraceFinder 4.1* settings	
Peak Detection	
Threshold Override	5 E5
S/N Ratio Threshold	100
Mass Tolerance (ppm)	5
Detection Algorithm	ICIS
Detection Method	Highest peak
Smoothing	1
Area Noise Factor	100
Peak Noise Factor	50
Baseline Window	5
Min Peak Height (S/N)	5
Noise Method	Incos
Min Peak Width	5
Multiplet Resolution	10
Area Tail Extension	5
Area Scan Window	0
Isotopic Pattern	
Fit Threshold (%)	50
Allowed Mass Deviation (ppm)	10
Allowed Intensity Deviation (%)	10

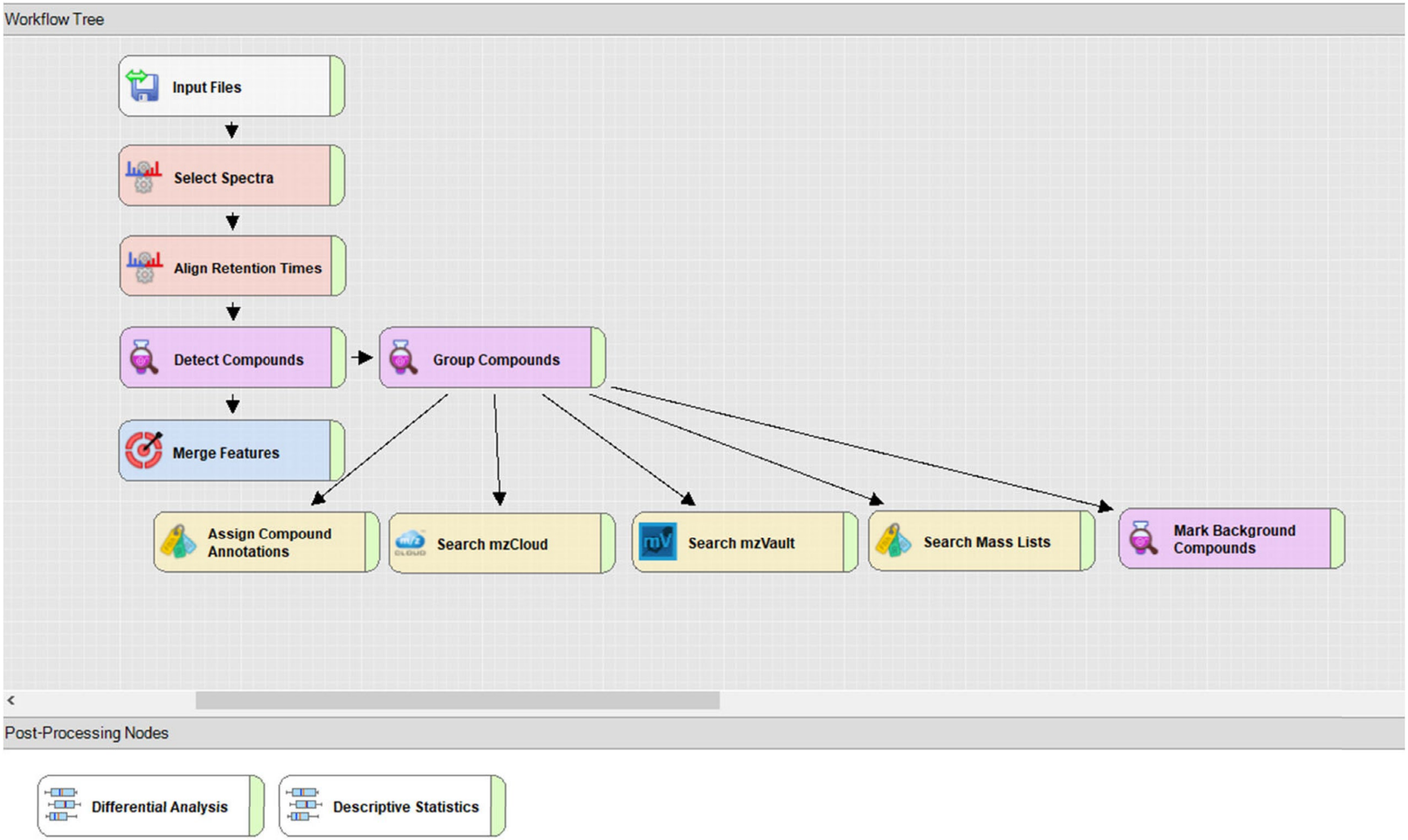

Figure S7. Screenshot of *Compound Discoverer 3.1* suspect screening workflow with node settings detailed in Table S7. The nodes represent the process steps in the workflow tree. The arrows represent the direction of connections between nodes. *MassBank* records (i.e., MassBank/MassBank-data: Release version 2020.09[30]) were imported as an offline *mzVault* 2.3 library.

Table S7. *Compound Discoverer 3.1* settings for suspect screening

Select Spectra	Align Retention Times	Search mzVault
1. General Settings:<br>-Precursor Selection: Use MS(n-1) Precursor;<br>2. Spectrum Properties Filters:<br>-Lower RT Limit: 0.5;<br>-Upper RT Limit: 0;<br>-First Scan: 0;<br>-Last Scan: 0;<br>-Lowest Charge State: 0;<br>-Highest Charge State: 0;<br>-Min. Precursor Mass: 100 Da;<br>-Max. Precursor Mass: 1000 Da;<br>-Total Intensity Threshold: 0;<br>-Minimum Peak Count: 1;<br>3. Scan Event Filters:<br>-Mass Analyzer: (Not specified);<br>-MS Order: Any;<br>-Activation Type: Not specified;<br>-Min. Collision Energy: 0,<br>-Max. Collision Energy: 1000;<br>-Scan Type: Any;<br>-Polarity Mode: (Not specified);<br>4. Peak Filters:<br>-S/N Threshold (FT-only): 1.5;<br>5. Replacements for Unrecognized Properties:<br>-Unrecognized Charge Replacements: 1;<br>-Unrecognized Mass Analyzer Replacements: ITMS;<br>-Unrecognized MS Order Replacements: MS2;<br>-Unrecognized Activation Type Replacements: CID;<br>-Unrecognized Polarity Replacements: +;<br>-Unrecognized MS Resolution @ 200 Replacements: 60000;<br>-Unrecognized MSn Resolution@ 200 Replacements: 7500.	1. General Settings:<br>-Alignment Model: Adaptive Curve;<br>-Mass Tolerance: 5.0 ppm;<br>-Maximum Shift (min): 2;	1. Search Settings:<br>-mzVault Library: \ mzVault September 2020.db; \MassBankNA.db; \Eawag.db; \Eawag_Additional_Specs.db;<br>-Compound Classes: All;<br>-Match Ion Activation Type: True;<br>-Match Ion Activation Energy: Match with Tolerance;<br>-Ion Activation Energy Tolerance: 20;<br>-Match Ionization Method: True;<br>-Apply Intensity Threshold: True;<br>-Precursor Mass Tolerance: 5.0 ppm;<br>-Match Analyzer Type; True;<br>-Search Algorithm: HighChem HighRes;<br>-Match Factor Threshold: 30;<br>-RT Tolerance [min]: 2;<br>-Use Retention Time: False;
	Detect Compounds	
	1. General Settings:<br>-Mass Tolerance [ppm]: 5.0 ppm;<br>-Intensity Tolerance (%): 30; -S/N Threshold: 3;<br>-Min. Peak Intensity: 1000;<br>-Ions: [M+H]+1; [M-H]-1; [M+Na]+1;<br>-Min Element Counts: C2 H4 O;<br>-Max Element Counts: C50 H190 Br3 Cl6 F21 N12 O40 P3 S4;	
	Group Compounds	Search Mass Lists
	1. Compound Consolidation: -Mass Tolerance [ppm]: 5.0 ppm;<br>-RT Tolerance [min]: 0.5;<br>-Preferred Ions: [M+H]+1; [M-H]-1; [M+Na]+1; [M+FA-H]-1; [M+Cl]-1 [M]+1; [M]-1;	1. Search Settings:<br>-Mass Lists: Shiru_SuspectDatabase;<br>-Use Retention Time: True;<br>-RT Tolerance [min]: 0.5;<br>-Mass Tolerance: 5.0 ppm;
	Merge Features	Search mzCloud
	1. Peak Consolidation:<br>-Mass Tolerance [ppm]: 5.0 ppm;<br>-RT Tolerance [min]: 0.5;	1. Search Settings:<br>-Compound Classes: All;<br>-Match Ion Activation Type: True;<br>-Match Ion Activation Energy: Match with Tolerance;<br>-Ion Activation Energy Tolerance: 20;<br>-Match Ionization Method: True;<br>-Apply Intensity Threshold: True;<br>-Identity Search: Cosine;<br>-Similarity Search: Similarity Forward;<br>-Match Factor Threshold: 30;
	Mark Background Compounds	
	1. General Settings:<br>-Max. Sample/Blank: 5;<br>-Max. Blank/Sample: 0;<br>-Hide Background: True;	
	Differential Analysis	Assign Compound Annotations
	1. General Settings:<br>-Log Transformation Values: True;	1. General Settings:<br>-Mass Tolerance [ppm]: 5.0 ppm;<br>2. Data Sources:<br>-Data Source #1: mzCloud Search;<br>-Data Source #2: mzVault Search;

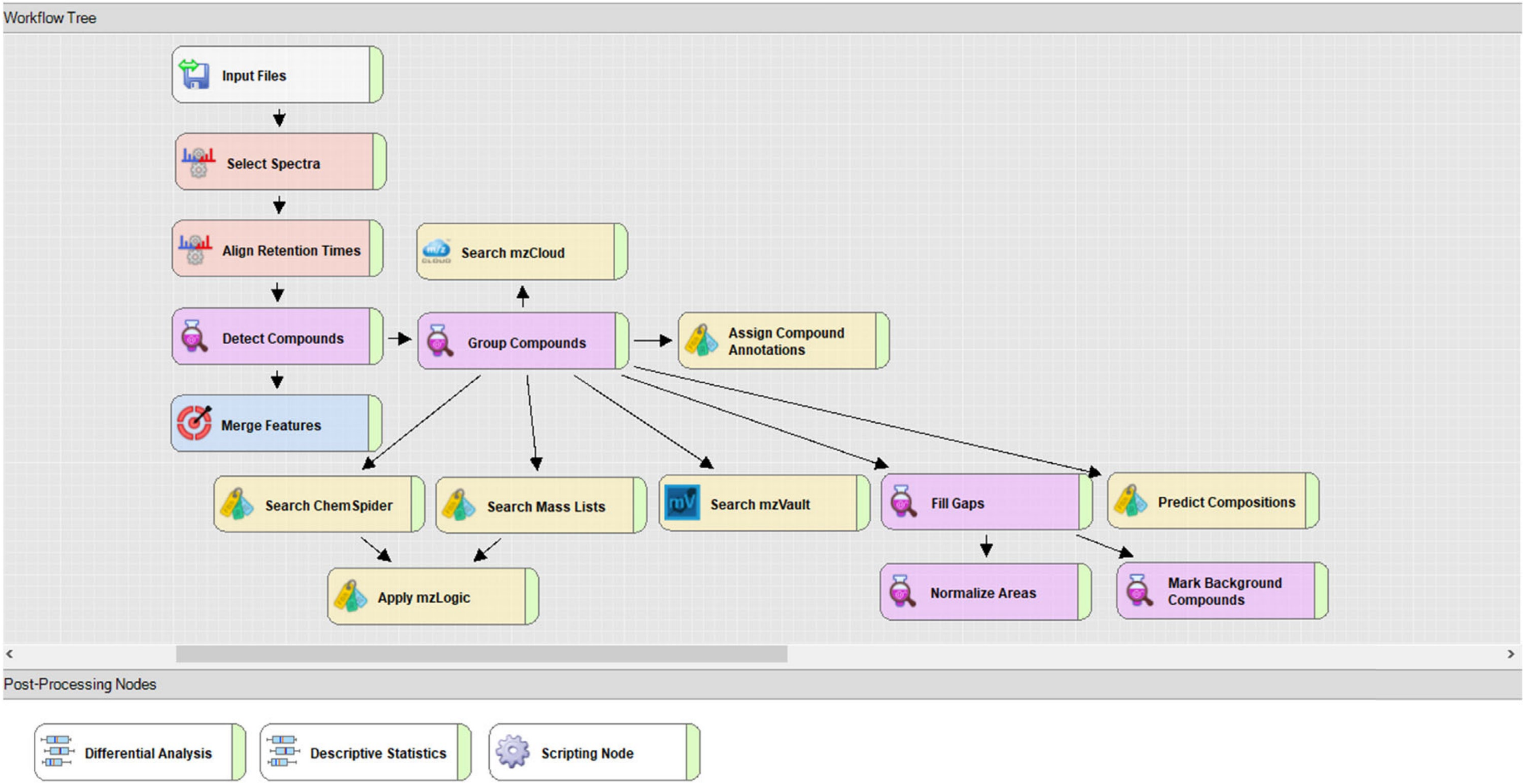

Figure S8. Screenshot of *Compound Discoverer 3.1* nontarget screening workflow with node settings detailed in Table S8. The nodes represent the process steps in the workflow tree. The arrows represent the direction of connections between nodes. *MassBank* records (i.e., MassBank/MassBank-data: Release version 2020.09[30]) were imported as an offline *mzVault* 2.3 library.

Table S8. *Compound Discoverer 3.1* settings for nontarget screening

Select Spectra	Align Retention Times	Search mzVault
1. General Settings:<br>-Precursor Selection: Use MS(n-1) Precursor;<br>2. Spectrum Properties Filters:<br>-Lower RT Limit: 0.5;<br>-Upper RT Limit: 0;<br>-First Scan: 0;<br>-Last Scan: 0;<br>-Lowest Charge State: 0;<br>-Highest Charge State: 0;<br>-Min. Precursor Mass: 100 Da;<br>-Max. Precursor Mass: 1000 Da;<br>-Total Intensity Threshold: 0;<br>-Minimum Peak Count: 1;<br>3. Scan Event Filters:<br>-Mass Analyzer: (Not specified);<br>-MS Order: Any;<br>-Activation Type: Not specified;<br>-Min. Collision Energy: 0,<br>-Max. Collision Energy: 1000;<br>-Scan Type: Any;<br>-Polarity Mode: (Not specified);<br>4. Peak Filters:<br>-S/N Threshold (FT-only): 1.5;<br>5. Replacements for Unrecognized Properties:<br>-Unrecognized Charge Replacements: 1;<br>-Unrecognized Mass Analyzer Replacements: ITMS;<br>-Unrecognized MS Order Replacements: MS2;<br>-Unrecognized Activation Type Replacements: CID;<br>-Unrecognized Polarity Replacements: +;<br>-Unrecognized MS Resolution @ 200 Replacements: 60000;<br>-Unrecognized MSn Resolution@ 200 Replacements: 7500.	1. General Settings:<br>-Alignment Model: Adaptive Curve;<br>-Mass Tolerance: 5.0 ppm;<br>-Maximum Shift (min): 2;<br>**Detect Compounds**<br>1. General Settings:<br>-Mass Tolerance [ppm]: 5.0 ppm;<br>-Intensity Tolerance (%): 30; -S/N Threshold: 3;<br>-Min. Peak Intensity: 1000;<br>-Ions: [M+H]+1; [M-H]-1; [M+Na]+1;<br>-Min Element Counts: C4 H4 O;<br>-Max Element Counts: C50 H100 Cl N3 O40 P S;	1. Search Settings:<br>-mzVault Library: \ mzVault September 2019.db; \MassBankNA.db; \Eawag.db; \Eawag_Additional_Specs.db;<br>-Compound Classes: All;<br>-Match Ion Activation Type: True;<br>-Match Ion Activation Energy: Match with Tolerance;<br>-Ion Activation Energy Tolerance: 20;<br>-Match Ionization Method: True;<br>-Apply Intensity Threshold: True;<br>-Precursor Mass Tolerance: 5.0 ppm;<br>-Match Analyzer Type; True;<br>-Search Algorithm: HighChem HighRes;<br>-Match Factor Threshold: 30;<br>-RT Tolerance [min]: 2;<br>-Use Retention Time: False;
	**Group Compounds**	**Search Mass Lists**
	1. Compound Consolidation: -Mass Tolerance [ppm]: 5.0 ppm;<br>-RT Tolerance [min]: 0.5;<br>-Preferred Ions: [M+H]+1; [M-H]-1; [M+Na]+1; [M+FA-H]-1; [M+Cl]-1 [M]+1; [M]-1;	1. Search Settings:<br>-Mass Lists: NORMANSusDat;<br>-Use Retention Time: True;<br>-RT Tolerance [min]: 0.5;<br>-Mass Tolerance: 5.0 ppm;
	**Merge Features**	**Search mzCloud**
	1. Peak Consolidation:<br>-Mass Tolerance [ppm]: 5.0 ppm;<br>-RT Tolerance [min]: 0.5;<br>**Apply mzLogic**<br>1. Search Settings:<br>-Max. #Compounds: 10;<br>-Max. #mzCloud Similarity Results to consider per compound: 10;<br>-Match Factor Threshold: 10;	1. Search Settings:<br>-Compound Classes: All;<br>-Match Ion Activation Type: True;<br>-Match Ion Activation Energy: Match with Tolerance;<br>-Ion Activation Energy Tolerance: 20;<br>-Match Ionization Method: True;<br>-Apply Intensity Threshold: True;<br>-Identity Search: Cosine;<br>-Similarity Search: Similarity Forward;<br>-Match Factor Threshold: 30;<br>**Search ChemSpider**<br>1. Search Settings:<br>-Database(s): EPA DSSTox; EPA Toxcast;<br>-Search Mode: By Formula or Mass;<br>-Mass Tolerance: 5.0 ppm;<br>-Max. # of results per compound: 10;<br>-Max. # of Predicted Compositions to be searched: 3;

<table>
<tr><th colspan="3">Table S8. Compound Discoverer 3.1 settings for nontarget screening (continued)</th></tr>
<tr><th>Predict Compositions</th><th>Fill Gaps</th><th>Assign Compound Annotations</th></tr>
<tr><td rowspan="7">1. General Settings:
-Mass Tolerance: 5.0 ppm;
-Min. Element Counts: C4 H4 O;
-Max Element Counts: C50 H100 Cl N3 O40 P S;
-Min. RDBE: 0;
-Max. RDBE: 40;
-Min. H/C: 0.3;
-Max. H/C: 2.3;
-Max. # Candidates: 10;
2. Pattern Matching
-Intensity Tolerance [%]: 30;
-Intensity Threshold: 0.1;
-S/N Threshold: 3;
-Use Dynamic Recalibration: True;
3. Fragments Matching
-Use Fragments Matching: True;
-Mass Tolerance; 10 ppm;
-S/N Threshold: 3;</td><td>1. General Settings:
-Mass Tolerance: 5.0 ppm;
-S/N Threshold: 3;</td><td rowspan="3">1. General Settings:
-Mass Tolerance [ppm]: 5.0 ppm;
2. Data Sources:
-Data Source #1: mzCloud Search;
-Data Source #2: mzVault Search;
-Data Source #3: Predicted Composition;</td></tr>
<tr><th>Normalize Areas</th></tr>
<tr><td>1. QC-based Area Correction:
-Min. QC Coverage [%]: 50;
-Max. QC Area RSD [%]: 30;
2. Area Normalization:
-Normalization Type: Constant Sum;
-Exclude Blanks: True</td></tr>
<tr><th>Mark Background Compounds</th><th>Scripting Node</th></tr>
<tr><td>1. General Settings:
-Max. Sample/Blank: 5;
-Max. Blank/Sample: 0;
-Hide Background: True;</td><td rowspan="3">1. Executable and Parameters:
-Path to Executable: C:\Program Files\R\R-4.1.0\bin\Rscript.exe;
-Command Line Arguments: C:\Rscripts\OCRatio\OCRatio.R %NODEARGS%;
-Requested Tables and Columns: Compounds: Formula;
-Use R-Friendly Columns: True;
-Archive Datafiles: False;</td></tr>
<tr><th>Differential Analysis</th></tr>
<tr><td>1. General Settings:
-Log Transformation Values: True;</td></tr>
</table>

Overall, suspect and nontarget screening prioritized 385 compounds. For each suspect or nontarget compound, the absolute SPE recovery, ion suppression, matrix factor, and limits of quantification (LOQs) were determined as detailed in our previous work[3] and summarized in **Table S9** and **Figure S9**. Target analysis was performed retrospectively to quantify the concentrations of confirmed OMPs in the samples. Briefly, twelve calibration standards (triplicate; each 500 mL) prepared in HPLC grade water containing target OMPs (typically 0-3000 ng/L) and ILIS (200 ng/L) were extracted and analyzed following the SPE-LC-HRMS method described in the *Main Text*. Calibration curves were constructed in *TraceFinder* by the non-weighted linear least squares regression algorithm (average R^2 = 0.9945±0.0055 for 326 OMPs with absolute SPE recoveries). Quantification was performed using the peak area ratios of target OMPs and structurally identical ILIS or ILIS with the closest chromatographic retention times. On average, the absolute spike recovery of ILIS was 84±27%. Method reproducibility was monitored based on the percent relative standard deviations (%RSDs) of the ILIS peak areas in calibration standards. Over the project period, the average %RSDs of the ILIS peak areas ranged from 4.1% to 21.1% with a mean of 9.4±3.0% (**Table S10**). None of the target OMPs was detected in the field blanks. Nondetects were assigned a concentration of 0 ng/L, while concentrations below the LOQs were used as is if peaks contained at least five consecutive full scan data points and one diagnostic MS2 fragment.

**Table S9.** SPE-LC-HRMS method performance for 385 suspect and nontarget OMPs											
Compound Name	CAS	Molecular Formula	Adduct	Exact Mass (*m/z*)	Diagnostic Fragment (*m/z*)	RT (min)	SPE Recovery	Ion Suppression	Matrix Factor	LOQs (ng/L)	R^2
17α-Estradiol	57-91-0	$C_{18}H_{24}O_2$	[M+H]+	273.1849	255.1745	17.81	NA	NA	NA	NA	NA
17β-Estradiol	50-28-2	$C_{18}H_{24}O_2$	[M+H]+	273.1849	255.1745	17.46	NA	NA	NA	NA	NA
3,4-Ethylenedioxymethcathinone *	802041-86-7	$C_{12}H_{15}NO_3$	[M+H]+	222.1125	204.1008	8.02	66.5%	121.4%	147.2%	6.8 – 10	0.9843
3,4-Methylenedioxymethamphetamine	42542-10-9	$C_{11}H_{15}NO_2$	[M+H]+	194.1176	163.0760	8.00	90.1%	97.3%	87.9%	50 – 57	0.9990
3,4-Methylenedioxy-N-ethylamphetamine	82801-81-8	$C_{12}H_{17}NO_2$	[M+H]+	208.1332	163.0746	8.93	71.0%	103.0%	83.4%	25 – 30	0.9866
4-Methylethcathinone	1225617-18-4	$C_{12}H_{17}NO$	[M+H]+	192.1383	146.0962	9.83	89.9%	90.9%	100.6%	1.0 – 1.0	0.9974
Abacavir	136470-78-5	$C_{14}H_{18}N_6O$	[M+H]+	287.1615	191.1030	8.65	102.7%	118.1%	89.2%	1.0 – 1.1	0.9980
Acamprosate	77337-76-9	$C_5H_{11}NO_4S$	[M+H]+	182.0482	140.0371	1.62	NA	NA	NA	NA	NA
Acebutolol	37517-30-9	$C_{18}H_{28}N_2O_4$	[M+H]+	337.2122	116.1064	10.45	57.7%	117.9%	126.9%	20 – 25	0.9969
Acetaminophen	103-90-2	$C_8H_9NO_2$	[M+H]+	152.0706	126.0100	3.65	93.5%	95.1%	99.4%	25 – 25	0.9995
Albuterol	18559-94-9	$C_{13}H_{21}NO_3$	[M+H]+	240.1594	148.0749	3.18	98.1%	203.3%	83.7%	10 – 12	0.9978
Aliskiren	173334-57-1	$C_{30}H_{53}N_3O_6$	[M+H]+	552.4007	436.3054	16.44	47.2%	141.6%	77.8%	1.0 – 1.3	0.9928
Allopurinol	315-30-0	$C_5H_4N_4O$	[M+H]+	137.0458	94.0648	1.92	NA	NA	NA	NA	NA
Amantadine	768-94-5	$C_{10}H_{17}N$	[M+H]+	152.1434	135.1162	9.78	103.1%	82.9%	92.2%	5.0 – 5.4	0.9952
Amitriptyline	50-48-6	$C_{20}H_{23}N$	[M+H]+	278.1903	233.1315	15.40	87.2%	106.9%	85.1%	10 – 12	0.9963
Amobarbital	57-43-2	$C_{11}H_{18}N_2O_3$	[M+H]+	227.1390	157.0600	14.83	NA	NA	NA	NA	NA
Amphetamine	300-62-9	$C_9H_{13}N$	[M+H]+	136.1121	119.0859	7.32	73.0%	108.4%	78.8%	50 – 63	0.9956
Androstanolone (5α-Dihydrotestosterone)	521-18-6	$C_{19}H_{30}O_2$	[M+H]+	291.2319	273.2212	19.71	92.5%	95.0%	53.2%	100 – 188	0.9969
Androstenedione	63-05-8	$C_{19}H_{26}O_2$	[M+H]+	287.2006	269.1892	17.38	107.7%	98.9%	102.1%	4.9 – 5.0	0.9965
Antipyrine	60-80-0	$C_{11}H_{12}N_2O$	[M+H]+	189.1022	147.0912	10.00	105.5%	66.3%	70.1%	1.0 – 1.4	0.9913
Artemisinin	63968-64-9	$C_{15}H_{22}O_5$	[M+H]+	283.1540	219.1373	17.84	103.0%	98.7%	92.1%	25 – 27	0.9935
Aspirin	50-78-2	$C_9H_8O_4$	[M-H]-	179.0350	134.9879	11.33	NA	NA	NA	NA	NA
Astemizole	68844-77-9	$C_{28}H_{31}FN_4O$	[M+H]+	459.2555	218.1548	12.10	76.6%	97.4%	188.7%	53 – 100	0.9822
Atazanavir	198904-31-3	$C_{38}H_{52}N_6O_7$	[M+H]+	705.3970	335.1963	18.84	97.4%	105.4%	110.3%	9.1 – 10	0.9936
Atenolol	29122-68-7	$C_{14}H_{22}N_2O_3$	[M+H]+	267.1703	190.0851	3.20	100.7%	97.2%	97.2%	10 – 10	0.9985
Atomoxetine	83015-26-3	$C_{17}H_{21}NO$	[M+H]+	256.1696	147.0796	14.65	35.4%	93.2%	119.8%	1.7 – 2.0	0.9987
Azelaic Acid	123-99-9	$C_9H_{16}O_4$	[M+H]+	189.1121	102.0546	13.17	84.9%	67.5%	44.3%	10 – 23	0.9978
Azithromycin	83905-01-5	$C_{38}H_{72}N_2O_{12}$	[M+H]+	749.5158	158.1171	12.20	75.2%	83.4%	72.1%	100 – 139	0.9869
Bamethan	3703-79-5	$C_{12}H_{19}NO_2$	[M+H]+	210.1489	192.1375	5.28	39.7%	103.7%	96.1%	10 – 10	0.9973
Benazepril	86541-75-5	$C_{24}H_{28}N_2O_5$	[M+H]+	425.2071	351.1690	14.73	43.7%	95.3%	113.1%	4.4 – 5.0	0.9994
Betamethasone	378-44-9	$C_{22}H_{29}FO_5$	[M+H]+	393.2072	355.1892	16.00	95.9%	92.4%	86.6%	10 – 12	0.9935
Betaxolol	63659-18-7	$C_{18}H_{29}NO_3$	[M+H]+	308.2220	116.1066	13.32	104.9%	110.0%	86.7%	5.0 – 5.8	0.9921
Bisoprolol	66722-44-9	$C_{18}H_{31}NO_4$	[M+H]+	326.2326	116.1066	12.33	93.3%	134.5%	90.3%	5.0 – 5.5	0.9968
Bumetanide	28395-03-1	$C_{17}H_{20}N_2O_5S$	[M+H]+	365.1166	184.0751	16.90	39.7%	94.5%	145.8%	6.9 – 10	0.9910
Bupivacaine	38396-39-3	$C_{18}H_{28}N_2O$	[M+H]+	289.2274	140.1430	12.42	98.1%	111.8%	87.4%	1.0 – 1.1	0.9976
Buprenorphine	52485-79-7	$C_{29}H_{41}NO_4$	[M+H]+	468.3108	414.2650	13.15	88.5%	94.6%	85.3%	1.0 – 1.2	0.9995
Bupropion	34911-55-2	$C_{13}H_{18}ClNO$	[M+H]+	240.1150	184.0517	11.83	94.7%	76.7%	51.8%	5.0 – 10	0.9984
Butalbital	77-26-9	$C_{11}H_{16}N_2O_3$	[M+H]+	225.1234	182.1168	9.05	88.7%	110.8%	145.6%	69 – 100	0.9861
Butylone	802575-11-7	$C_{12}H_{15}NO_3$	[M+H]+	222.1125	204.1011	8.85	95.5%	124.0%	104.5%	10 – 10	0.9889
Caffeine	58-08-2	$C_8H_{10}N_4O_2$	[M+H]+	195.0877	138.0656	8.46	107.7%	99.1%	102.1%	1.0 – 1.0	0.9922
Carbamazepine	298-46-4	$C_{15}H_{12}N_2O$	[M+H]+	237.1022	194.0955	14.68	107.8%	91.0%	92.0%	10 – 11	0.9970
Celecoxib	169590-42-5	$C_{17}H_{14}F_3N_3O_2S$	[M+H]+	382.0832	362.0757	19.42	96.8%	69.4%	45.8%	10 – 22	0.9978
Celiprolol	56980-93-9	$C_{20}H_{33}N_3O_4$	[M+H]+	380.2544	324.1909	11.42	74.7%	101.5%	138.8%	7.2 – 10	0.9987
Cetirizine	83881-51-0	$C_{21}H_{25}ClN_2O_3$	[M+H]+	389.1627	201.0458	16.24	102.2%	100.4%	93.4%	10 – 11	0.9989
Chlonixin	17737-65-4	$C_{13}H_{11}ClN_2O_2$	[M+H]+	263.0582	NA	NA	NA	NA	NA	NA	NA
Cimetidine	51481-61-9	$C_{10}H_{16}N_6S$	[M+H]+	253.1230	159.0690	3.44	27.0%	62.4%	68.9%	10 – 15	0.9913
Ciprofloxacin	85721-33-1	$C_{17}H_{18}FN_3O_3$	[M+H]+	332.1405	315.1294	9.38	NA	NA	NA	NA	NA

Table S9. SPE-LC-HRMS method performance for 385 suspect and nontarget OMPs (continued)											
Compound Name	CAS	Molecular Formula	Adduct	Exact Mass (*m/z*)	Diagnostic Fragment (*m/z*)	RT (min)	SPE Recovery	Ion Suppression	Matrix Factor	LOQs (ng/L)	R^2
Citalopram	59729-33-8	$C_{20}H_{21}FN_2O$	[M+H]+	325.1711	262.1021	13.11	99.5%	86.0%	77.7%	5.0 – 6.4	0.9958
Clarithromycin	81103-11-9	$C_{38}H_{69}NO_{13}$	[M+H]+	748.4842	158.1170	16.50	88.3%	103.3%	78.1%	10 – 13	0.9953
Clenbuterol	37148-27-9	$C_{12}H_{18}Cl_2N_2O$	[M+H]+	277.0869	203.0132	10.12	46.7%	100.7%	91.9%	10 – 11	0.9977
Clindamycin	18323-44-9	$C_{18}H_{33}ClN_2O_5S$	[M+H]+	425.1872	126.1272	13.30	103.6%	102.7%	78.4%	25 – 32	0.9927
Clomiphene	911-45-5	$C_{26}H_{28}ClNO$	[M+H]+	406.1932	100.1116	18.87	13.9%	90.8%	86.3%	10 – 12	0.9910
Codeine	76-57-3	$C_{18}H_{21}NO_3$	[M+H]+	300.1594	215.1057	4.67	100.8%	98.3%	107.0%	9.3 – 10	0.9948
Cortisone	53-06-5	$C_{21}H_{28}O_5$	[M+H]+	361.2010	163.1112	14.33	84.0%	87.4%	79.4%	10 – 13	0.9978
Cycloheximide	66-81-9	$C_{15}H_{23}NO_4$	[M+H]+	282.1700	247.1324	12.83	95.8%	112.3%	56.6%	10 – 18	0.9972
Cyclopentolate	512-15-2	$C_{17}H_{25}NO_3$	[M+H]+	292.1907	274.1794	12.50	82.4%	141.0%	102.7%	4.9 – 5.0	0.9983
Darunavir	206361-99-1	$C_{27}H_{37}N_3O_7S$	[M+H]+	548.2425	392.1997	15.71	106.5%	99.2%	85.9%	10 – 12	0.9915
Desipramine	50-47-5	$C_{18}H_{22}N_2$	[M+H]+	267.1856	238.9279	15.18	34.9%	99.8%	142.0%	3.5 – 5.0	0.9963
Desomorphine	427-00-9	$C_{17}H_{21}NO_2$	[M+H]+	272.1645	215.1058	7.44	104.7%	94.0%	101.3%	1.0 – 1.0	0.9902
Desoxycortone	64-85-7	$C_{21}H_{30}O_3$	[M+H]+	331.2268	313.2154	17.88	62.7%	104.2%	88.5%	10 – 11	0.9974
Desvenlafaxine (O-Desmethyl Venlafaxine)	93413-62-8	$C_{16}H_{25}NO_2$	[M+H]+	264.1958	246.1841	10.40	108.8%	77.1%	83.4%	10 – 12	0.9958
Detomidine	76631-46-4	$C_{12}H_{14}N_2$	[M+H]+	187.1230	81.0443	11.64	92.4%	75.6%	58.6%	1.0 – 1.7	0.9925
Dexpanthenol	81-13-0	$C_9H_{19}NO_4$	[M+H]+	206.1387	189.1111	3.81	88.6%	119.0%	44.5%	10 – 22	0.9985
Dextromethorphan	125-71-3	$C_{18}H_{25}NO$	[M+H]+	272.2009	215.1421	13.00	95.4%	77.6%	70.7%	1.0 – 1.4	0.9993
Diazepam	439-14-5	$C_{16}H_{13}ClN_2O$	[M+H]+	285.0789	154.0410	17.45	104.3%	99.2%	93.7%	1.0 – 1.1	0.9968
Diclofenac	15307-86-5	$C_{14}H_{11}Cl_2NO_2$	[M+H]+	296.0240	215.0491	19.88	110.3%	77.2%	62.7%	10 – 16	0.9988
Dienogest	65928-58-7	$C_{20}H_{25}NO_2$	[M+H]+	312.1958	161.0960	15.35	99.8%	85.5%	93.8%	10 – 11	0.9990
Dihydrocodeine	125-28-0	$C_{18}H_{23}NO_3$	[M+H]+	302.1751	284.1279	4.66	91.1%	84.8%	75.1%	50 – 67	0.9990
Dihydromorphine	509-60-4	$C_{17}H_{21}NO_3$	[M+H]+	288.1594	231.1027	1.95	88.0%	102.2%	84.2%	50 – 59	0.9964
Dihydrostreptomycin	128-46-1	$C_{21}H_{41}N_7O_{12}$	[M+H]+	584.2886	263.1454	1.24	NA	NA	NA	NA	NA
Diltiazem	42399-41-7	$C_{22}H_{26}N_2O_4S$	[M+H]+	415.1686	397.1172	13.88	96.1%	109.1%	78.5%	10 – 13	0.9960
Dimethylone (bk-MDDMA) *	765231-58-1	$C_{12}H_{15}NO_3$	[M+H]+	222.1125	177.0553	6.88	82.9%	91.2%	78.4%	50 – 64	0.9978
Dinoprostone	363-24-6	$C_{20}H_{32}O_5$	[M-H]-	351.2177	333.2072	17.83	106.2%	71.6%	85.2%	5.0 – 5.9	0.9927
Diphenhydramine	58-73-1	$C_{17}H_{21}NO$	[M+H]+	256.1696	224.0828	13.22	96.9%	114.7%	90.4%	10 – 11	0.9988
Dobutamine	34368-04-2	$C_{18}H_{23}NO_3$	[M+H]+	302.1751	137.0593	9.09	NA	NA	NA	NA	NA
Dopamine	51-61-6	$C_8H_{11}NO_2$	[M+H]+	154.0863	137.0590	1.48	107.2%	147.3%	101.1%	25 – 25	0.9986
Doxylamine	469-21-6	$C_{17}H_{22}N_2O$	[M+H]+	271.1805	182.0959	8.14	80.1%	93.0%	370.6%	2.7 – 10	0.9964
Drospirenone	67392-87-4	$C_{24}H_{30}O_3$	[M+H]+	367.2268	171.1163	17.90	93.2%	110.1%	105.5%	0.9 – 1.0	0.9963
Duloxetine	116539-59-4	$C_{18}H_{19}NOS$	[M+H]+	298.1260	183.0793	15.08	NA	NA	NA	NA	NA
Ecgonine	481-37-8	$C_9H_{15}NO_3$	[M+H]+	186.1125	168.1015	1.58	NA	NA	NA	NA	NA
Efavirenz	154598-52-4	$C_{14}H_9ClF_3NO_2$	[M+H]+	316.0347	244.0128	20.04	100.6%	78.5%	63.3%	10 – 16	0.9950
Enalapril	75847-73-3	$C_{20}H_{28}N_2O_5$	[M+H]+	377.2071	234.1479	13.15	38.5%	100.5%	68.9%	50 – 73	0.9982
Ephedrine	299-42-3	$C_{10}H_{15}NO$	[M+H]+	166.1226	148.1156	5.19	97.2%	130.2%	45.1%	5.0 – 11	0.9975
Epinephrine	51-43-4	$C_9H_{13}NO_3$	[M+H]+	184.0968	166.0855	1.60	NA	NA	NA	NA	NA
Epitestosterone	481-30-1	$C_{19}H_{28}O_2$	[M+H]+	289.2162	109.0644	19.08	100.8%	83.4%	87.7%	10 – 11	0.9950
Erythromycin	114-07-8	$C_{37}H_{67}NO_{13}$	[M+H]+	734.4685	591.3411	14.99	68.1%	86.9%	147.1%	6.8 – 10	0.9913
Estriol	50-27-1	$C_{18}H_{24}O_3$	[M+H]+	289.1798	253.1577	13.48	NA	NA	NA	NA	NA
Estrone	53-16-7	$C_{18}H_{22}O_2$	[M+H]+	271.1693	197.0951	17.54	NA	NA	NA	NA	NA
Ethosuximide	77-67-8	$C_7H_{11}NO_2$	[M-H]-	140.0717	NA	13.81	NA	NA	NA	NA	NA
Eugenol	97-53-0	$C_{10}H_{12}O_2$	[M+H]+	165.0910	123.0436	13.90	70.4%	69.9%	134.0%	75 – 100	0.9987
Fentanyl	437-38-7	$C_{22}H_{28}N_2O$	[M+H]+	337.2274	188.1443	12.37	91.0%	88.7%	105.9%	47 – 50	0.9952
Fexofenadine	83799-24-0	$C_{32}H_{39}NO_4$	[M+H]+	502.2952	466.2721	14.98	104.7%	99.1%	98.9%	10 – 10	0.9935
Finafloxacin	209342-40-5	$C_{20}H_{19}FN_4O_4$	[M+H]+	399.1463	178.1215	10.36	NA	NA	NA	NA	NA
Fingolimod	162359-55-9	$C_{19}H_{33}NO_2$	[M+H]+	308.2584	255.2100	20.53	16.5%	85.6%	78.0%	10 – 13	0.9831

Table S9. SPE-LC-HRMS method performance for 385 suspect and nontarget OMPs (continued)											
Compound Name	CAS	Molecular Formula	Adduct	Exact Mass (*m/z*)	Diagnostic Fragment (*m/z*)	RT (min)	SPE Recovery	Ion Suppression	Matrix Factor	LOQs (ng/L)	R^2
Flecainide	54143-55-4	$C_{17}H_{20}F_6N_2O_3$	[M+H]+	415.1451	398.1173	13.11	104.7%	89.8%	82.4%	5.0 – 6.1	0.9947
Fluconazole	86386-73-4	$C_{13}H_{12}F_2N_6O$	[M+H]+	307.1113	238.0774	11.06	106.0%	70.0%	61.9%	1.0 – 1.6	0.9914
Fluoxetine	54910-89-3	$C_{17}H_{18}F_3NO$	[M+H]+	310.1413	202.4488	16.11	77.3%	101.8%	70.0%	5.0 – 7.1	0.9918
Flurandrenolide	1524-88-5	$C_{24}H_{33}FO_6$	[M+H]+	437.2334	285.1645	16.94	97.1%	88.6%	79.7%	10 – 13	0.9985
Furosemide	54-31-9	$C_{12}H_{11}ClN_2O_5S$	[M+H]+	331.0150	312.9846	13.10	NA	NA	NA	NA	NA
Gabapentin	60142-96-3	$C_9H_{17}NO_2$	[M+H]+	172.1332	154.1221	7.34	8.2%	113.1%	10.9%	0.2 – 1.8	0.9975
Gatifloxacin	112811-59-3	$C_{19}H_{22}FN_3O_4$	[M+H]+	376.1667	332.1759	10.52	NA	NA	NA	NA	NA
Gemfibrozil	25812-30-0	$C_{15}H_{22}O_3$	[M+H]+	251.1642	202.5128	21.84	102.7%	87.6%	90.7%	10 – 11	0.9950
Glutethimide	77-21-4	$C_{13}H_{15}NO_2$	[M+H]+	218.1176	175.0986	14.38	107.0%	75.2%	48.1%	50 – 104	0.9934
Griseofulvin	126-07-8	$C_{17}H_{17}ClO_6$	[M+H]+	353.0786	165.0543	15.62	117.2%	83.3%	93.3%	10 – 11	0.9954
Guaifenesin	93-14-1	$C_{10}H_{14}O_4$	[M+H]+	199.0965	125.0591	10.84	104.7%	91.6%	95.6%	10 – 10	0.9922
Hydrochlorothiazide	58-93-5	$C_7H_8ClN_3O_4S_2$	[M+H]+	297.9718	280.9443	3.29	NA	NA	NA	NA	NA
Hydrocodone	125-29-1	$C_{18}H_{21}NO_3$	[M+H]+	300.1594	199.0760	6.96	89.4%	101.9%	81.4%	50 – 61	0.9985
Hydrocortisone	50-23-7	$C_{21}H_{30}O_5$	[M+H]+	363.2166	327.1955	14.90	110.3%	61.9%	57.6%	10 – 17	0.9987
Hydromorphone	466-99-9	$C_{17}H_{19}NO_3$	[M+H]+	286.1438	185.0608	2.41	88.4%	98.2%	87.5%	50 – 57	0.9988
Hydroxyprogesterone	68-96-2	$C_{21}H_{30}O_3$	[M+H]+	331.2268	109.0644	18.41	97.5%	103.7%	95.5%	5.0 – 5.2	0.9968
Ibuprofen	15687-27-1	$C_{13}H_{18}O_2$	[M+H]+	207.1380	189.1629	20.37	NA	NA	NA	NA	NA
Icosapent	10417-94-4	$C_{20}H_{30}O_2$	[M+H]+	303.2319	201.1102	10.23	NA	NA	NA	NA	NA
Imipenem	64221-86-9	$C_{12}H_{17}N_3O_4S$	[M+H]+	300.1013	NA	8.39	NA	NA	NA	NA	NA
Iohexol	66108-95-0	$C_{19}H_{26}I_3N_3O_9$	[M+H]+	821.8876	803.8738	2.30	NA	NA	NA	NA	NA
Irbesartan	138402-11-6	$C_{25}H_{28}N_6O$	[M+H]+	429.2397	207.0907	16.48	101.4%	97.9%	78.5%	10 – 13	0.9841
Isopentedrone *	1429402-11-8	$C_{12}H_{17}NO$	[M+H]+	192.1383	174.1275	10.61	35.2%	97.1%	29.7%	5.0 – 17	0.9864
Isoprenaline	7683-59-2	$C_{11}H_{17}NO_3$	[M+H]+	212.1281	194.1167	1.92	NA	NA	NA	NA	NA
Ketamine	6740-88-1	$C_{13}H_{16}ClNO$	[M+H]+	238.0993	179.0615	9.80	113.2%	82.9%	97.6%	1.0 – 1.0	0.9930
Labetalol	36894-69-6	$C_{19}H_{24}N_2O_3$	[M+H]+	329.1860	162.0545	12.07	43.6%	97.0%	67.3%	10 – 15	0.9971
Lamivudine	134678-17-4	$C_8H_{11}N_3O_3S$	[M+H]+	230.0594	112.0502	1.94	NA	NA	NA	NA	NA
Lamotrigine	84057-84-1	$C_9H_7Cl_2N_5$	[M+H]+	256.0151	210.9816	10.90	107.2%	66.0%	62.7%	10 – 16	0.9962
Levamisole	14769-73-4	$C_{11}H_{12}N_2S$	[M+H]+	205.0794	178.0677	5.83	100.9%	109.2%	114.6%	8.7 – 10	0.9968
Levetiracetam	102767-28-2	$C_8H_{14}N_2O_2$	[M+H]+	171.1128	126.0908	6.14	102.2%	128.4%	97.9%	10 – 10	0.9986
Levonorgestrel	797-63-7	$C_{21}H_{28}O_2$	[M+H]+	313.2162	245.1892	18.90	97.8%	83.3%	85.0%	10 – 12	0.9987
Levorphanol	77-07-6	$C_{17}H_{23}NO$	[M+H]+	258.1852	199.1114	10.29	98.1%	70.1%	97.1%	2.0 – 2.1	0.9985
Lidocaine	137-58-6	$C_{14}H_{22}N_2O$	[M+H]+	235.1805	86.0958	8.90	106.1%	98.6%	101.4%	2.0 – 2.0	0.9991
Lisinopril	76547-98-3	$C_{21}H_{31}N_3O_5$	[M-H]-	404.2191	114.0562	8.92	NA	NA	NA	NA	NA
Lomefloxacin	98079-51-7	$C_{17}H_{19}F_2N_3O_3$	[M+H]+	352.1467	265.1138	9.73	NA	NA	NA	NA	NA
Lopinavir	192725-17-0	$C_{37}H_{48}N_4O_5$	[M+H]+	629.3698	155.1174	21.42	101.2%	104.5%	107.6%	4.6 – 5.0	0.9918
Losartan	114798-26-4	$C_{22}H_{23}ClN_6O$	[M+H]+	423.1695	207.0908	16.20	81.9%	96.6%	72.2%	10 – 14	0.9947
Lovastatin	75330-75-5	$C_{24}H_{36}O_5$	[M+H]+	405.2636	199.1475	22.12	361.9%	91.5%	354.8%	7.0 – 25	0.9971
Maprotiline	10262-69-8	$C_{20}H_{23}N$	[M+H]+	278.1903	250.1584	15.41	86.1%	98.0%	85.1%	5.0 – 5.9	0.9969
Mebendazole	31431-39-7	$C_{16}H_{13}N_3O_3$	[M+H]+	296.1030	264.0763	14.49	83.1%	89.4%	76.3%	10 – 13	0.9992
Meclofenamic Acid	644-62-2	$C_{14}H_{11}Cl_2NO_2$	[M+H]+	296.0240	278.0127	21.16	32.5%	88.4%	105.4%	9.5 – 10	0.9920
Medroxyprogesterone	520-85-4	$C_{22}H_{32}O_3$	[M+H]+	345.2424	123.0800	19.54	93.1%	87.2%	70.9%	10 – 14	0.9952
Mefenamic Acid	61-68-7	$C_{15}H_{15}NO_2$	[M+H]+	242.1176	224.1064	21.53	35.8%	91.6%	128.9%	7.8 – 10	0.9863
Melatonin	73-31-4	$C_{13}H_{16}N_2O_2$	[M+H]+	233.1285	174.0907	11.39	97.5%	75.7%	64.9%	10 – 15	0.9972
Memantine	19982-08-2	$C_{12}H_{21}N$	[M+H]+	180.1747	163.1476	13.39	101.7%	105.1%	74.8%	1.0 – 1.3	0.9956
Meperidine	57-42-1	$C_{15}H_{21}NO_2$	[M+H]+	248.1645	220.1328	11.27	102.2%	71.3%	84.1%	10 – 12	0.9944
Mesalamine	89-57-6	$C_7H_7NO_3$	[M+H]+	154.0499	136.0390	1.55	NA	NA	NA	NA	NA
Metacetamol	621-42-1	$C_8H_9NO_2$	[M+H]+	152.0706	138.0338	5.87	81.3%	87.9%	94.1%	10 – 11	0.9983

Table S9. SPE-LC-HRMS method performance for 385 suspect and nontarget OMPs (continued)											
Compound Name	CAS	Molecular Formula	Adduct	Exact Mass (*m/z*)	Diagnostic Fragment (*m/z*)	RT (min)	SPE Recovery	Ion Suppression	Matrix Factor	LOQs (ng/L)	R^2
Metaproterenol	586-06-1	$C_{11}H_{17}NO_3$	[M+H]+	212.1281	194.1170	1.87	20.6%	90.4%	58.6%	10 – 17	0.9988
Metaraminol	54-49-9	$C_9H_{13}NO_2$	[M+H]+	168.1019	150.0907	1.92	59.6%	112.7%	80.8%	25 – 31	0.9973
Metaxalone	1665-48-1	$C_{12}H_{15}NO_3$	[M+H]+	222.1125	161.0956	15.11	109.3%	78.5%	86.4%	10 – 12	0.9970
Metformin	657-24-9	$C_4H_{11}N_5$	[M+H]+	130.1087	113.0817	1.64	72.1%	66.5%	21.7%	10 – 46	0.9670
Methadone	76-99-3	$C_{21}H_{27}NO$	[M+H]+	310.2165	223.1107	15.22	102.9%	102.4%	107.4%	0.9 – 1.0	0.9985
Methamphetamine	537-46-2	$C_{10}H_{15}N$	[M+H]+	150.1277	91.0538	7.77	41.8%	103.5%	139.3%	3.6 – 5.0	0.9942
Methcathinone	5650-44-2	$C_{10}H_{13}NO$	[M+H]+	164.1070	146.0961	4.99	27.4%	95.7%	116.6%	4.3 – 5.0	0.9969
Methocarbamol	532-03-6	$C_{11}H_{15}NO_5$	[M+H]+	242.1023	163.0748	11.28	111.2%	85.6%	104.4%	10 – 10	0.9944
Methoxyphedrine (Methedrone) *	530-54-1	$C_{11}H_{15}NO_2$	[M+H]+	194.1176	176.1068	8.30	66.4%	113.8%	93.0%	5.0 – 5.4	0.9880
Methylphenidate	113-45-1	$C_{14}H_{19}NO_2$	[M+H]+	234.1489	117.0694	10.78	41.2%	99.0%	101.2%	10 – 10	0.9986
Methyltestosterone	58-18-4	$C_{20}H_{30}O_2$	[M+H]+	303.2319	202.4471	19.13	44.6%	95.4%	159.9%	3.1 – 5.0	0.9977
Metoprolol	51384-51-1	$C_{15}H_{25}NO_3$	[M+H]+	268.1907	191.1058	10.60	101.6%	92.6%	96.8%	10 – 10	0.9970
Minoxidil	38304-91-5	$C_9H_{15}N_5O$	[M+H]+	210.1349	193.1314	9.67	33.4%	110.2%	104.3%	10 – 10	0.9987
Molindone	7416-34-4	$C_{16}H_{24}N_2O_2$	[M+H]+	277.1911	100.0753	10.26	42.4%	71.1%	19.3%	10 – 52	0.9874
Monuron	150-68-5	$C_9H_{11}ClN_2O$	[M+H]+	199.0633	126.0099	13.22	43.7%	92.5%	123.0%	4.1 – 5.0	0.9997
Morphine	57-27-2	$C_{17}H_{19}NO_3$	[M+H]+	286.1438	201.0904	1.92	98.8%	92.6%	107.8%	46 – 50	0.9836
Moxifloxacin	151096-09-2	$C_{21}H_{24}FN_3O_4$	[M+H]+	402.1824	384.1705	11.16	NA	NA	NA	NA	NA
Mycophenolic Acid	24280-93-1	$C_{17}H_{20}O_6$	[M+H]+	321.1333	207.0648	16.60	89.5%	97.7%	79.8%	10 – 13	0.9985
Nadolol	42200-33-9	$C_{17}H_{27}NO_4$	[M+H]+	310.2013	254.1376	9.06	104.2%	128.8%	96.4%	10 – 10	0.9991
Nalidixic Acid	389-08-2	$C_{12}H_{12}N_2O_3$	[M+H]+	233.0921	215.0808	13.56	NA	NA	NA	NA	NA
Nalorphine	62-67-9	$C_{19}H_{21}NO_3$	[M+H]+	312.1594	251.0690	10.00	49.7%	95.2%	145.3%	17 – 25	0.9963
Naloxone	465-65-6	$C_{19}H_{21}NO_4$	[M+H]+	328.1543	310.1455	4.68	96.6%	89.5%	81.4%	50 – 61	0.9995
Naproxen	22204-53-1	$C_{14}H_{14}O_3$	[M+H]+	231.1016	188.0688	17.24	103.1%	92.7%	87.9%	10 – 11	0.9874
Nebivolol	99200-09-6	$C_{22}H_{25}F_2NO_4$	[M+H]+	406.1824	151.0549	15.62	32.9%	100.4%	145.3%	6.9 – 10	0.9995
N-Ethylamphetamine	33817-11-7	$C_{11}H_{17}N$	[M+H]+	164.1434	119.0850	8.84	77.2%	108.0%	80.4%	25 – 31	0.9896
Nevirapine	129618-40-2	$C_{15}H_{14}N_4O$	[M+H]+	267.1240	226.0842	12.07	96.9%	103.0%	56.8%	10 – 18	0.9978
Nicotine	54-11-5	$C_{10}H_{14}N_2$	[M+H]+	163.1230	132.0802	1.45	NA	NA	NA	NA	NA
Norepinephrine	51-41-2	$C_8H_{11}NO_3$	[M+H]+	170.0812	152.0699	1.63	31.0%	58.9%	82.5%	25 – 30	0.9943
Norfloxacin	70458-96-7	$C_{16}H_{18}FN_3O_3$	[M+H]+	320.1405	233.1077	9.13	NA	NA	NA	NA	NA
Nortriptyline	72-69-5	$C_{19}H_{21}N$	[M+H]+	264.1747	233.1318	15.15	36.8%	96.7%	151.9%	6.6 – 10	0.9944
Oxcarbazepine	28721-07-5	$C_{15}H_{12}N_2O_2$	[M+H]+	253.0972	210.0908	13.22	102.0%	95.6%	82.3%	5.0 – 6.1	0.9922
Oxprenolol	6452-71-7	$C_{15}H_{23}NO_3$	[M+H]+	266.1751	116.1065	11.83	40.9%	108.4%	94.1%	10 – 11	0.9988
Oxycodone	76-42-6	$C_{18}H_{21}NO_4$	[M+H]+	316.1543	298.1457	6.06	88.0%	102.2%	86.8%	50 – 58	0.9985
Oxymorphone	76-41-5	$C_{17}H_{19}NO_4$	[M+H]+	302.1387	284.1294	2.16	95.2%	97.6%	99.0%	50 – 51	0.9940
Paliperidone	144598-75-4	$C_{23}H_{27}FN_4O_3$	[M+H]+	427.2140	207.1126	11.25	105.8%	73.1%	74.6%	10 – 13	0.9915
Pentazocine	359-83-1	$C_{19}H_{27}NO$	[M+H]+	286.2165	218.1537	11.83	107.1%	69.0%	78.8%	1.0 – 1.3	0.9946
Pentedrone	879722-57-3	$C_{12}H_{17}NO$	[M+H]+	192.1383	174.1271	10.32	88.1%	81.9%	60.8%	50 – 82	0.9954
Pentobarbital	76-74-4	$C_{11}H_{18}N_2O_3$	[M-H]-	225.1245	182.1187	14.89	123.6%	123.4%	137.5%	18 – 25	0.9951
Perindopril	82834-16-0	$C_{19}H_{32}N_2O_5$	[M+H]+	369.2384	172.1325	13.94	43.4%	98.6%	118.3%	8.5 – 10	0.9998
Phencyclidine	77-10-1	$C_{17}H_{25}N$	[M+H]+	244.2060	159.1160	12.10	89.0%	108.4%	125.9%	20 – 25	0.9948
Phendimetrazine	634-03-7	$C_{12}H_{17}NO$	[M+H]+	192.1383	174.1271	15.75	92.6%	120.1%	106.6%	47 – 50	0.9914
Phenmetrazine	134-49-6	$C_{11}H_{15}NO$	[M+H]+	178.1226	134.0956	7.54	41.2%	98.6%	85.6%	10 – 12	0.9991
Phentermine	122-09-8	$C_{10}H_{15}N$	[M+H]+	150.1277	133.1006	9.43	86.9%	109.3%	92.6%	10 – 11	0.9974
Phenylephrine	59-42-7	$C_9H_{13}NO_2$	[M+H]+	168.1019	150.0906	9.76	116.9%	90.7%	122.0%	0.2 – 0.2	0.9973
Phenytoin	57-41-0	$C_{15}H_{12}N_2O_2$	[M+H]+	253.0972	182.0958	14.25	116.9%	90.7%	73.9%	10 – 14	0.9973
Pilocarpine	92-13-7	$C_{11}H_{16}N_2O_2$	[M+H]+	209.1285	163.1226	2.10	NA	NA	NA	NA	NA
Pirlimycin	79548-73-5	$C_{17}H_{31}ClN_2O_5S$	[M+H]+	411.1715	363.1671	13.02	97.3%	83.8%	61.3%	10 – 16	0.9770

Table S9. SPE-LC-HRMS method performance for 385 suspect and nontarget OMPs (continued)											
Compound Name	CAS	Molecular Formula	Adduct	Exact Mass (*m/z*)	Diagnostic Fragment (*m/z*)	RT (min)	SPE Recovery	Ion Suppression	Matrix Factor	LOQs (ng/L)	R^2
Prasterone	53-43-0	$C_{19}H_{28}O_2$	[M+H]+	289.2162	271.2043	18.77	158.5%	93.2%	54.9%	50 – 91	0.9935
Pregabalin	148553-50-8	$C_8H_{17}NO_2$	[M+H]+	160.1332	142.1221	7.12	6.8%	114.1%	9.8%	10 – 102	0.9907
Prilocaine	721-50-6	$C_{13}H_{20}N_2O$	[M+H]+	221.1648	136.0756	9.63	109.1%	78.4%	92.2%	1.0 – 1.1	0.9961
Primidone	125-33-7	$C_{12}H_{14}N_2O_2$	[M+H]+	219.1128	162.0905	10.90	110.5%	53.7%	58.4%	10 – 17	0.9953
Propafenone	54063-53-5	$C_{21}H_{27}NO_3$	[M+H]+	342.2064	116.1064	14.93	64.0%	99.6%	114.6%	8.7 – 10	0.9988
Propoxyphene	469-62-5	$C_{22}H_{29}NO_2$	[M+H]+	340.2271	266.1893	14.70	109.7%	98.6%	74.3%	5.0 – 6.7	0.9965
Propranolol	525-66-6	$C_{16}H_{21}NO_2$	[M+H]+	260.1645	183.0797	13.05	97.3%	100.9%	91.8%	5.0 – 5.4	0.9949
Protriptyline	438-60-8	$C_{19}H_{21}N$	[M+H]+	264.1747	233.1318	15.68	32.2%	101.2%	138.2%	3.6 – 5.0	0.9967
Pseudoephedrine	90-82-4	$C_{10}H_{15}NO$	[M+H]+	166.1226	148.1156	5.61	61.9%	106.8%	92.6%	25 – 27	0.9968
Pyridoxine	65-23-6	$C_8H_{11}NO_3$	[M+H]+	170.0812	152.0699	1.63	31.0%	58.9%	82.5%	25 – 30	0.9940
Pyrimethamine	58-14-0	$C_{12}H_{13}ClN_4$	[M+H]+	249.0902	233.0581	12.10	94.3%	112.7%	61.1%	5.0 – 8.2	0.9988
Pyrovalerone	3563-49-3	$C_{16}H_{23}NO$	[M+H]+	246.1852	175.1111	12.36	86.8%	108.7%	77.5%	1.0 – 1.3	0.9961
Quinapril	85441-61-8	$C_{25}H_{30}N_2O_5$	[M+H]+	439.2228	234.1488	15.98	42.7%	96.3%	118.9%	8.4 – 10	0.9995
Ramipril	87333-19-5	$C_{23}H_{32}N_2O_5$	[M+H]+	417.2384	234.1480	15.41	42.9%	104.5%	148.0%	3.4 – 5.0	0.9974
Ranitidine	66357-35-5	$C_{13}H_{22}N_4O_3S$	[M+H]+	315.1485	176.0480	3.12	47.5%	33.6%	73.0%	10 – 14	0.9765
Rimantadine	13392-28-4	$C_{12}H_{21}N$	[M+H]+	180.1747	163.1476	13.46	92.7%	105.1%	74.8%	10 – 13	0.9956
Ropivacaine	84057-95-4	$C_{17}H_{26}N_2O$	[M+H]+	275.2118	126.1272	11.19	105.9%	66.2%	73.0%	10 – 14	0.9918
Sarafloxacin	98105-99-8	$C_{20}H_{17}F_2N_3O_3$	[M+H]+	386.1311	299.0985	10.23	NA	NA	NA	NA	NA
Secobarbital	76-73-3	$C_{12}H_{18}N_2O_3$	[M+H]+	239.1390	182.1282	10.60	108.1%	69.0%	60.0%	25 – 42	0.9849
Sertraline	79617-96-2	$C_{17}H_{17}Cl_2N$	[M+H]+	306.0811	275.0381	16.60	95.4%	110.8%	79.5%	5.0 – 6.3	0.9455
Sitagliptin	486460-32-6	$C_{16}H_{15}F_6N_5O$	[M+H]+	408.1254	235.0792	10.89	77.8%	60.2%	44.4%	1.0 – 2.3	0.9956
Sotalol	3930-20-9	$C_{12}H_{20}N_2O_3S$	[M+H]+	273.1267	213.0684	2.76	102.6%	116.0%	29.3%	25 – 85	0.9967
Stavudine	3056-17-5	$C_{10}H_{12}N_2O_4$	[M+H]+	225.0870	182.1168	4.24	110.8%	126.3%	130.5%	77 – 100	0.9945
Streptomycin	57-92-1	$C_{21}H_{39}N_7O_{12}$	[M+H]+	582.2730	263.1453	1.26	NA	NA	NA	NA	NA
Sulfadoxine	2447-57-6	$C_{12}H_{14}N_4O_4S$	[M+H]+	311.0809	156.0109	10.11	98.9%	102.1%	80.4%	10 – 12	0.9955
Sulfamethazine	57-68-1	$C_{12}H_{14}N_4O_2S$	[M+H]+	279.0910	204.0431	8.43	23.1%	63.5%	119.6%	8.4 – 10	0.9974
Sulfamethoxazole	723-46-6	$C_{10}H_{11}N_3O_3S$	[M+H]+	254.0594	156.0107	9.72	103.2%	92.3%	104.9%	1.0 – 1.0	0.9991
Sulfapyridine	144-83-2	$C_{11}H_{11}N_3O_2S$	[M+H]+	250.0645	156.0108	5.69	90.2%	72.8%	15.6%	25 – 160	0.9899
Sulfisomidine	515-64-0	$C_{12}H_{14}N_4O_2S$	[M+H]+	279.0910	186.0326	3.89	107.9%	109.9%	44.0%	10 – 23	0.9933
Tacrolimus	104987-11-3	$C_{44}H_{69}NO_{12}$	[M+H]+	804.4893	202.4592	22.27	NA	NA	NA	NA	NA
Tapentadol	175591-23-8	$C_{14}H_{23}NO$	[M+H]+	222.1852	121.0643	11.03	95.9%	74.7%	76.7%	1.0 – 1.3	0.9929
Telmisartan	144701-48-4	$C_{33}H_{30}N_4O_2$	[M+H]+	515.2442	497.2315	16.23	94.4%	122.7%	173.1%	2.9 – 5.0	0.9982
Terbutaline	23031-25-6	$C_{12}H_{19}NO_3$	[M+H]+	226.1438	152.0699	2.79	36.0%	97.7%	77.9%	10 – 13	0.9978
Testosterone	58-22-0	$C_{19}H_{28}O_2$	[M+H]+	289.2162	109.0643	18.32	107.9%	81.7%	78.4%	10 – 13	0.9936
Thebaine	115-37-7	$C_{19}H_{21}NO_3$	[M+H]+	312.1594	201.0883	4.09	69.4%	100.2%	93.0%	10 – 11	0.9979
Theophylline	58-55-9	$C_7H_8N_4O_2$	[M+H]+	181.0720	124.0500	6.10	105.9%	75.4%	28.7%	50 – 174	0.9899
Tiletamine	14176-49-9	$C_{12}H_{17}NOS$	[M+H]+	224.1104	179.0518	8.78	40.0%	101.4%	115.4%	4.3 – 5.0	0.9994
Tolperisone	728-88-1	$C_{16}H_{23}NO$	[M+H]+	246.1852	98.0959	11.45	77.3%	88.4%	109.3%	23 – 25	0.9961
Tramadol	27203-92-5	$C_{16}H_{25}NO_2$	[M+H]+	264.1958	246.1840	10.22	NA	NA	NA	NA	NA
Tranexamic Acid	1197-18-8	$C_8H_{15}NO_2$	[M+H]+	158.1176	148.1024	1.69	113.3%	120.3%	59.0%	200 – 339	0.9746
Trenbolone	10161-33-8	$C_{18}H_{22}O_2$	[M+H]+	271.1693	253.1580	17.00	92.1%	108.7%	99.1%	10 – 10	0.9980
Tretinoin (Retinoic Acid)	302-79-4	$C_{20}H_{28}O_2$	[M-H]-	299.2017	255.2120	25.61	NA	NA	NA	NA	NA
Triamterene	396-01-0	$C_{12}H_{11}N_7$	[M+H]+	254.1149	237.0874	9.63	NA	NA	NA	NA	NA
Trihexyphenidyl	144-11-6	$C_{20}H_{31}NO$	[M+H]+	302.2478	98.0959	14.95	42.3%	97.0%	135.7%	3.7 – 5.0	0.9960
Trimethoprim	738-70-5	$C_{14}H_{18}N_4O_3$	[M+H]+	291.1452	230.1152	8.45	105.8%	96.0%	69.6%	1.0 – 1.4	0.9988
Valsartan	137862-53-4	$C_{24}H_{29}N_5O_3$	[M+H]+	436.2343	207.0911	18.09	98.2%	91.1%	67.8%	100 – 147	0.9971
Varenicline	249296-44-4	$C_{13}H_{13}N_3$	[M+H]+	212.1182	169.0757	6.64	85.3%	95.2%	69.8%	50 – 72	0.9937

Table S9. SPE-LC-HRMS method performance for 385 suspect and nontarget OMPs (continued)											
Compound Name	CAS	Molecular Formula	Adduct	Exact Mass (*m/z*)	Diagnostic Fragment (*m/z*)	RT (min)	SPE Recovery	Ion Suppression	Matrix Factor	LOQs (ng/L)	R^2
Venlafaxine	93413-69-5	$C_{17}H_{27}NO_2$	[M+H]+	278.2115	260.2000	12.49	102.2%	90.6%	87.9%	10 – 11	0.9981
Xylazine	7361-61-7	$C_{12}H_{16}N_2S$	[M+H]+	221.1107	164.0523	10.03	40.6%	101.6%	98.5%	5.0 – 5.1	0.9942
Zidovudine	30516-87-1	$C_{10}H_{13}N_5O_4$	[M+H]+	268.1040	139.0306	8.88	99.9%	93.7%	62.4%	25 – 40	0.9984
1,7-Dimethyluric Acid	33868-03-0	$C_7H_8N_4O_3$	[M+H]+	197.0669	140.0459	5.26	45.1%	85.0%	68.3%	50 – 73	0.9959
10,11-Dihydro-10-hydroxy Carbamazepine	29331-92-8	$C_{15}H_{14}N_2O_2$	[M+H]+	255.1128	237.1012	12.49	81.5%	104.5%	78.6%	10 – 13	0.9934
1-Carboxycyclohexaneacetic Acid	67950-95-2	$C_9H_{14}O_4$	[M+H]+	187.0965	159.0495	13.41	NA	NA	NA	NA	NA
1-Methylxanthine	6136-37-4	$C_6H_6N_4O_2$	[M+H]+	167.0564	110.0344	2.82	92.1%	109.6%	88.8%	50 – 56	0.9970
2-Ethyl-2-phenylmalonamide	7206-76-0	$C_{11}H_{14}N_2O_2$	[M+H]+	207.1128	130.9783	8.16	88.6%	119.6%	92.4%	10 – 11	0.9966
EDDP	30223-73-5	$C_{20}H_{23}N$	[M+H]+	278.1903	249.1502	13.07	25.2%	97.5%	182.4%	14 – 25	0.9809
3-Hydroxy Cotinine	34834-67-8	$C_{10}H_{12}N_2O_2$	[M+H]+	193.0972	174.1496	1.63	92.4%	55.1%	53.9%	50 – 93	0.9916
Androsterone	53-41-8	$C_{19}H_{30}O_2$	[M+H]+	291.2319	255.2090	20.42	114.5%	88.1%	144.7%	17 – 25	0.9933
Anhydroecgonine Methyl Ester	43021-26-7	$C_{10}H_{15}NO_2$	[M+H]+	182.1176	151.0760	2.25	101.2%	153.4%	86.8%	50 – 58	0.9948
Benzoylecgonine	519-09-5	$C_{16}H_{19}NO_4$	[M+H]+	290.1387	168.1012	9.77	99.5%	102.8%	102.9%	1.0 – 1.0	0.9939
Carbamazepine-10,11-epoxide	36507-30-9	$C_{15}H_{12}N_2O_2$	[M+H]+	253.0972	210.0910	12.65	104.2%	87.0%	94.0%	10 – 16	0.9725
Cotinine	486-56-6	$C_{10}H_{12}N_2O$	[M+H]+	177.1022	149.0224	1.76	92.5%	79.9%	22.3%	100 – 448	0.9986
Ecgonine Methyl Ester	7143-09-1	$C_{10}H_{17}NO_3$	[M+H]+	200.1281	182.1168	1.48	63.4%	113.8%	91.6%	25 – 27	0.9935
Hydroxybupropion	92264-81-8	$C_{13}H_{18}ClNO_2$	[M+H]+	256.1099	238.0985	11.25	80.8%	87.1%	71.2%	25 – 35	0.9953
Metoprolol Acid (Atenolol Acid)	56392-14-4	$C_{14}H_{21}NO_4$	[M+H]+	268.1543	191.0695	8.23	34.7%	98.6%	158.1%	16 – 25	0.9969
N4-Acetylsulfamethoxazole	21312-10-7	$C_{12}H_{13}N_3O_4S$	[M+H]+	296.0700	198.0212	11.31	109.3%	56.6%	67.7%	1.0 – 1.5	0.9953
N-Desmethyl Citalopram	62498-67-3	$C_{19}H_{19}FN_2O$	[M+H]+	311.1554	149.0225	8.15	NA	NA	NA	NA	NA
N-Desmethyl Tramadol	73806-55-0	$C_{15}H_{23}NO_2$	[M+H]+	250.1802	189.1264	10.86	71.8%	101.7%	80.1%	10 – 12	0.9891
N-Desmethyl Venlafaxine	149289-30-5	$C_{16}H_{25}NO_2$	[M+H]+	264.1958	215.1425	12.56	66.9%	107.9%	99.9%	25 – 25	0.9955
Norcocaine	18717-72-1	$C_{16}H_{19}NO_4$	[M+H]+	290.1387	168.1012	11.15	71.8%	89.6%	77.5%	25 – 32	0.9939
Norcodeine	467-15-2	$C_{17}H_{19}NO_3$	[M+H]+	286.1438	268.1343	4.98	82.6%	101.9%	83.8%	50 – 60	0.9996
Norfentanyl	1609-66-1	$C_{14}H_{20}N_2O$	[M+H]+	233.1648	150.0919	10.12	45.1%	98.9%	45.6%	50 – 110	0.9989
Norhydrocodone	5083-62-5	$C_{17}H_{19}NO_3$	[M+H]+	286.1438	199.0762	7.24	80.5%	102.2%	79.4%	50 – 63	0.9997
Noroxycodone	57664-96-7	$C_{17}H_{19}NO_4$	[M+H]+	302.1387	NA	15.75	NA	NA	NA	NA	NA
O-Desmethyl Tramadol	73986-53-5	$C_{15}H_{23}NO_2$	[M+H]+	250.1802	189.1264	10.86	71.8%	101.7%	80.1%	10 – 12	0.9891
Paraxanthine (1,7-Dimethylxanthine)	611-59-6	$C_7H_8N_4O_2$	[M+H]+	181.0720	124.0502	5.50	92.1%	109.6%	88.8%	25 – 28	0.9983
Ritalinic Acid	19395-41-6	$C_{13}H_{17}NO_2$	[M+H]+	220.1332	84.0804	9.87	28.5%	97.7%	30.4%	5.0 – 16	0.9980
Salicylic Acid	69-72-7	$C_7H_6O_3$	[M+H]+	139.0390	121.0278	15.33	NA	NA	NA	NA	NA
Tramadol N-Oxide	147441-56-3	$C_{16}H_{25}NO_3$	[M+H]+	280.1907	262.1790	10.95	82.1%	89.5%	70.7%	25 – 35	0.9936
(4-Chloro-2-methylphenoxy)acetic Acid	94-74-6	$C_9H_9ClO_3$	[M-H]-	199.0168	141.0114	16.54	123.3%	107.8%	45.8%	5.0 – 11	0.9967
2,4-D	94-75-7	$C_8H_6Cl_2O_3$	[M-H]-	218.9621	160.9570	16.15	61.0%	101.8%	87.0%	5.0 – 5.7	0.9969
2-Naphthoxyacetic Acid	120-23-0	$C_{12}H_{10}O_3$	[M-H]-	201.0557	143.0505	15.34	75.4%	89.7%	81.4%	5.0 – 6.1	0.9976
4-Chlorophenoxyacetic Acid	122-88-3	$C_8H_7ClO_3$	[M-H]-	185.0011	126.9957	13.96	79.4%	82.3%	69.5%	5.0 – 7.2	0.9978
8-Hydroxyquinoline	148-24-3	C_9H_7NO	[M+H]+	146.0600	118.0648	3.08	NA	NA	NA	NA	NA
Abscisic Acid	21293-29-8	$C_{15}H_{20}O_4$	[M+H]+	265.1434	222.0544	13.66	105.9%	113.5%	119.8%	21 – 25	0.9915
Acetamiprid	135410-20-7	$C_{10}H_{11}ClN_4$	[M+H]+	223.0745	126.0100	10.93	109.9%	82.7%	96.9%	5.0 – 5.2	0.9936
Aldicarb	116-06-3	$C_7H_{14}N_2O_2S$	[M+Na]+	213.0668	170.1028	12.40	83.4%	48.4%	16.0%	100 – 625	0.9940
Ametryn	834-12-8	$C_9H_{17}N_5S$	[M+H]+	228.1277	186.0801	14.06	105.5%	76.8%	56.2%	1.0 – 1.8	0.9906
Atrazine	1912-24-9	$C_8H_{14}ClN_5$	[M+H]+	216.1011	174.0532	15.46	112.4%	85.2%	77.9%	5.0 – 6.4	0.9963
Bentazon	25057-89-0	$C_{10}H_{12}N_2O_3S$	[M-H]-	239.0496	132.0308	14.23	15.2%	88.6%	16.6%	1.0 – 6.0	0.9534
Bifenazate	149877-41-8	$C_{17}H_{20}N_2O_3$	[M+H]+	301.1547	198.0908	18.47	70.1%	103.2%	132.6%	19 – 25	0.9843
Bromacil	314-40-9	$C_9H_{13}BrN_2O_2$	[M+H]+	261.0233	204.9606	13.70	105.7%	88.5%	62.7%	10 – 16	0.9922
Butralin	33629-47-9	$C_{14}H_{21}N_3O_4$	[M+H]+	296.1605	240.0972	23.40	23.8%	97.6%	101.8%	10 – 10	0.9924
Camphor	76-22-2	$C_{10}H_{16}O$	[M+H]+	153.1274	135.1162	16.35	104.7%	89.8%	82.4%	10 – 12	0.9947

**Table S9.** SPE-LC-HRMS method performance for 385 suspect and nontarget OMPs (continued)											
Compound Name	CAS	Molecular Formula	Adduct	Exact Mass (*m/z*)	Diagnostic Fragment (*m/z*)	RT (min)	SPE Recovery	Ion Suppression	Matrix Factor	LOQs (ng/L)	R^2
Carbaryl	63-25-2	$C_{12}H_{11}NO_2$	[M+H]+	202.0863	145.0642	14.47	54.1%	94.7%	57.9%	10 – 17	0.9955
Carbendazim	10605-21-7	$C_9H_9N_3O_2$	[M+H]+	192.0768	148.1115	6.76	90.3%	87.8%	58.9%	1.0 – 1.7	0.9893
Carbofuran	1563-66-2	$C_{12}H_{15}NO_3$	[M+H]+	222.1125	165.0903	13.90	100.2%	105.5%	86.8%	5.0 – 5.8	0.9922
Chloridazon	1698-60-8	$C_{10}H_8ClN_3O$	[M+H]+	222.0429	104.0491	10.72	109.4%	71.2%	59.1%	1.0 – 1.7	0.9941
Clofibric Acid	882-09-7	$C_{10}H_{11}ClO_3$	[M-H]-	213.0324	126.9959	16.96	77.8%	100.1%	91.2%	5.0 – 5.5	0.9974
Clothianidin	210880-92-5	$C_6H_8ClN_5O_2S$	[M+H]+	250.0160	169.0537	9.99	103.1%	72.2%	76.1%	10 – 13	0.9963
Cyprodinil	121552-61-2	$C_{14}H_{15}N_3$	[M+H]+	226.1339	133.0755	17.05	37.5%	96.3%	146.5%	6.8 – 10	0.9928
Dichlorvos	62-73-7	$C_4H_7Cl_2O_4P$	[M+H]+	220.9532	127.0150	13.71	48.6%	189.3%	25.9%	10 – 39	0.9952
Dimethachlor	50563-36-5	$C_{13}H_{18}ClNO_2$	[M+H]+	256.1099	224.0828	16.28	94.0%	114.2%	100.5%	10 – 10	0.9980
Dinotefuran	165252-70-0	$C_7H_{14}N_4O_3$	[M+H]+	203.1139	129.0892	4.73	114.6%	123.2%	132.0%	7.6 – 10	0.9985
Dinoterb	1420-07-1	$C_{10}H_{12}N_2O_5$	[M-H]-	239.0673	194.0456	20.28	NA	NA	NA	NA	NA
Diuron	330-54-1	$C_9H_{10}Cl_2N_2O$	[M+H]+	233.0243	159.9709	16.03	95.8%	105.9%	92.5%	10 – 11	0.9957
Ethoxyquin	91-53-2	$C_{14}H_{19}NO$	[M+H]+	218.1539	175.1114	13.92	NA	NA	NA	NA	NA
Ethyl Butylacetylaminopropionate	52304-36-6	$C_{11}H_{21}NO_3$	[M+H]+	216.1594	170.1170	14.67	105.3%	101.8%	89.2%	10 – 11	0.9974
Fenamidone	161326-34-7	$C_{17}H_{17}N_3OS$	[M+H]+	312.1165	236.1179	17.42	97.5%	86.7%	97.5%	1.0 – 1.0	0.9968
Fipronil	120068-37-3	$C_{12}H_4Cl_2F_6N_4OS$	[M+H]+	436.9460	367.9495	19.69	NA	NA	NA	NA	NA
Fluometuron	2164-17-2	$C_{10}H_{11}F_3N_2O$	[M+H]+	233.0896	160.0362	15.06	111.8%	95.8%	103.6%	10 – 10	0.9983
Fluridone	59756-60-4	$C_{19}H_{14}F_3NO$	[M+H]+	330.1100	310.1030	16.63	46.2%	94.8%	157.9%	6.3 – 10	0.9976
Imazapyr	81334-34-1	$C_{13}H_{15}N_3O_3$	[M+H]+	262.1186	149.0344	10.01	45.5%	86.4%	70.6%	5.0 – 7.1	0.9965
Imidacloprid	138261-41-3	$C_9H_{10}ClN_5O_2$	[M+H]+	256.0596	175.0970	9.97	113.3%	82.8%	86.6%	1.0 – 1.2	0.9958
Indole-3-butyric Acid	133-32-4	$C_{12}H_{13}NO_2$	[M+H]+	204.1019	186.0907	14.53	91.7%	61.9%	50.4%	25 – 50	0.9964
Isoproturon	34123-59-6	$C_{12}H_{18}N_2O$	[M+H]+	207.1492	165.1016	15.82	93.8%	107.2%	93.6%	1.0 – 1.1	0.9971
Malathion	121-75-5	$C_{10}H_{19}O_6PS_2$	[M+H]+	331.0433	109.0644	17.89	88.1%	84.9%	90.4%	5.0 – 5.5	0.9963
Mecoprop	93-65-2	$C_{10}H_{11}ClO_3$	[M-H]-	213.0324	141.0114	17.94	54.1%	142.1%	52.4%	5.0 – 10	0.9978
Metalaxyl	57837-19-1	$C_{15}H_{21}NO_4$	[M+H]+	280.1543	220.1324	15.89	90.3%	128.6%	105.7%	9.5 – 10	0.9970
Metolachlor	51218-45-2	$C_{15}H_{22}ClNO_2$	[M+H]+	284.1412	252.1139	19.10	106.1%	92.7%	89.1%	5.0 – 5.6	0.9975
Metsulfuron-methyl	74223-64-6	$C_{14}H_{15}N_5O_6S$	[M+H]+	382.0816	167.0555	13.77	78.1%	104.1%	76.7%	10 – 13	0.9981
Napropamide	15299-99-7	$C_{17}H_{21}NO_2$	[M+H]+	272.1645	171.0800	18.87	109.6%	88.1%	84.6%	10 – 12	0.9991
Nitenpyram	150824-47-8	$C_{11}H_{15}ClN_4O_2$	[M+H]+	271.0956	159.0798	6.95	108.4%	100.4%	86.4%	10 – 12	0.9991
Oxamyl	23135-22-0	$C_7H_{13}N_3O_3S$	[M+H]+	220.0750	116.9735	7.07	NA	NA	NA	NA	NA
Pendimethalin	40487-42-1	$C_{13}H_{19}N_3O_4$	[M+H]+	282.1448	212.0660	22.89	22.9%	94.4%	104.1%	10 – 10	0.9950
Piperonyl Butoxide	51-03-6	$C_{19}H_{30}O_5$	[M+H]+	339.2166	202.6737	22.37	NA	NA	NA	NA	NA
Pirimicarb	23103-98-2	$C_{11}H_{18}N_4O_2$	[M+H]+	239.1503	182.1282	10.54	104.5%	77.6%	85.9%	10 – 12	0.9927
Prometon	1610-18-0	$C_{10}H_{19}N_5O$	[M+H]+	226.1662	184.1186	13.38	100.1%	90.1%	74.8%	1.0 – 1.3	0.9988
Prometryn	7287-19-6	$C_{10}H_{19}N_5S$	[M+H]+	242.1434	200.0961	15.62	96.5%	88.0%	71.4%	1.0 – 1.4	0.9960
Propachlor	1918-16-7	$C_{11}H_{14}ClNO$	[M+H]+	212.0837	170.0359	15.68	76.1%	100.5%	95.5%	10 – 10	0.9969
Propamocarb	24579-73-5	$C_9H_{20}N_2O_2$	[M+H]+	189.1598	144.1012	4.07	93.8%	98.4%	49.8%	10 – 20	0.9798
Propazine	139-40-2	$C_9H_{16}ClN_5$	[M+H]+	230.1167	188.0691	17.07	106.9%	102.0%	88.9%	5.0 – 5.6	0.9892
Pyracarbolid	24691-76-7	$C_{13}H_{15}NO_2$	[M+H]+	218.1176	175.0985	13.84	68.4%	102.8%	119.7%	8.4 – 10	0.9958
Pyrimethanil	53112-28-0	$C_{12}H_{13}N_3$	[M+H]+	200.1182	183.0911	14.41	42.6%	92.9%	117.1%	8.5 – 10	0.9991
Siduron	1982-49-6	$C_{14}H_{20}N_2O$	[M+H]+	233.1648	137.0704	17.23	110.7%	109.2%	106.3%	9.4 – 10	0.9888
Simazine	122-34-9	$C_7H_{12}ClN_5$	[M+H]+	202.0854	132.0318	13.62	105.6%	95.8%	65.7%	5.0 – 7.6	0.9988
Sulfoxaflor	946578-00-3	$C_{10}H_{10}F_3N_3OS$	[M+H]+	278.0569	174.0518	11.26	149.4%	39.5%	37.3%	10 – 27	0.9951
Terbumeton	33693-04-8	$C_{10}H_{19}N_5O$	[M+H]+	226.1662	170.1653	13.40	74.8%	103.1%	86.1%	25 – 29	0.9969
Terbuthylazine	5915-41-3	$C_9H_{16}ClN_5$	[M+H]+	230.1167	174.0535	17.40	101.0%	109.7%	90.5%	5.0 – 5.5	0.9967
Thiabendazole	148-79-8	$C_{10}H_7N_3S$	[M+H]+	202.0433	175.0321	8.33	96.2%	94.1%	77.1%	5.0 – 6.5	0.9951
Thiacloprid	111988-49-9	$C_{10}H_9ClN_4S$	[M+H]+	253.0309	126.0099	11.78	104.8%	80.6%	70.9%	10 – 14	0.9981

**Table S9.** SPE-LC-HRMS method performance for 385 suspect and nontarget OMPs (continued)											
Compound Name	CAS	Molecular Formula	Adduct	Exact Mass (*m/z*)	Diagnostic Fragment (*m/z*)	RT (min)	SPE Recovery	Ion Suppression	Matrix Factor	LOQs (ng/L)	R^2
Thiamethoxam	153719-23-4	$C_8H_{10}ClN_5O_3S$	[M+H]+	292.0266	211.0641	8.42	109.3%	116.6%	109.5%	9.1 – 10	0.9981
Tridemorph	24602-86-6	$C_{19}H_{39}NO$	[M+H]+	298.3104	130.1221	19.30	NA	NA	NA	NA	NA
Trinexapac-ethyl	95266-40-3	$C_{13}H_{16}O_5$	[M+H]+	253.1071	207.0644	15.88	76.1%	95.5%	87.0%	10 – 11	0.9966
2-Aminobenzimidazole	934-32-7	$C_7H_7N_3$	[M+H]+	134.0713	107.0598	4.40	92.1%	101.8%	118.0%	8.5 – 10	0.9985
Acetochlor ESA	187022-11-3	$C_{14}H_{21}NO_5S$	[M-H]-	314.1068	120.9604	14.41	NA	NA	NA	NA	NA
Alachlor ESA	142363-53-9	$C_{14}H_{21}NO_5S$	[M-H]-	314.1068	120.9604	14.41	NA	NA	NA	NA	NA
Atrazine-2-hydroxy	2163-68-0	$C_8H_{15}N_5O$	[M+H]+	198.1349	156.0876	9.37	100.1%	82.4%	73.2%	10 – 14	0.9954
Atrazine-desethyl	6190-65-4	$C_6H_{10}ClN_5$	[M+H]+	188.0698	146.0224	11.44	106.4%	70.9%	63.0%	1.0 – 1.6	0.9914
Atrazine-desisopropyl	1007-28-9	$C_5H_8ClN_5$	[M+H]+	174.0541	132.0318	8.99	107.0%	96.4%	71.1%	10 – 14	0.9954
Carbofuran-3-hydroxy	16655-82-6	$C_{12}H_{15}NO_4$	[M+H]+	238.1074	163.0746	10.85	98.2%	105.5%	100.2%	10 – 10	0.9902
Metolachlor ESA	171118-09-5	$C_{15}H_{23}NO_5S$	[M+H]+	330.1370	298.1100	14.77	108.8%	42.4%	31.7%	10 – 32	0.9805
Metolachlor OA	152019-73-3	$C_{15}H_{21}NO_4$	[M+H]+	280.1543	248.1274	16.02	90.3%	125.1%	103.0%	24 – 25	0.9983
N-(2,4-Dimethylphenyl)formamide	60397-77-5	$C_9H_{11}NO$	[M+H]+	150.0913	132.0800	15.72	91.2%	104.5%	103.1%	24 – 25	0.9950
Propachlor OA	70628-36-3	$C_{11}H_{13}NO_3$	[M+H]+	208.0968	120.0438	11.35	87.9%	81.5%	64.2%	10 – 16	0.9971
2-Hydroxybenzothiazole	934-34-9	C_7H_5NOS	[M+H]+	152.0165	124.0211	12.73	91.6%	83.0%	37.8%	10 – 26	0.9984
4-Methyl-1H-benzotriazole	29878-31-7	$C_7H_7N_3$	[M+H]+	134.0713	106.0647	11.80	86.9%	76.2%	59.9%	10 – 17	0.9994
5-Methyl-1H-benzotriazole	136-85-6	$C_7H_7N_3$	[M+H]+	134.0713	106.0647	11.82	86.9%	76.2%	59.9%	10 – 17	0.9994
4-Propylbenzoic Acid *	2438-05-3	$C_{10}H_{12}O_2$	[M-H]-	163.0765	131.0136	18.09	NA	NA	NA	NA	NA
Acesulfame	33665-90-6	$C_4H_5NO_4S$	[M-H]-	161.9867	82.0303	2.38	NA	NA	NA	NA	NA
Benzophenone	119-61-9	$C_{13}H_{10}O$	[M+H]+	183.0804	105.0330	17.49	90.0%	68.5%	62.9%	10 – 16	0.9868
Oxybenzone (Benzophenone-3)	131-57-7	$C_{14}H_{12}O_3$	[M+H]+	229.0859	151.0384	19.23	90.8%	69.1%	49.7%	5.0 – 10	0.9971
Benzothiazole	95-16-9	C_7H_5NS	[M+H]+	136.0216	122.3269	12.97	91.1%	82.1%	63.7%	10 – 16	0.9853
Benzotriazole	95-14-7	$C_6H_5N_3$	[M+H]+	120.0556	120.0556	9.12	78.8%	91.3%	51.6%	10 – 19	0.9992
Benzyl Butyl Phthalate	85-68-7	$C_{19}H_{20}O_4$	[M+H]+	313.1434	149.0230	21.37	96.5%	98.3%	71.9%	10 – 14	0.9858
Butylparaben	94-26-8	$C_{11}H_{14}O_3$	[M-H]-	193.0870	137.0246	17.60	107.8%	97.5%	55.5%	50 – 90	0.9927
Dibutyl Phthalate	84-74-2	$C_{16}H_{22}O_4$	[M+H]+	279.1591	149.0227	21.34	NA	NA	NA	NA	NA
Diethyl Phthalate	84-66-2	$C_{12}H_{14}O_4$	[M+H]+	223.0965	149.0228	16.07	92.6%	138.7%	125.7%	8.0 – 10	0.9966
Diisobutyl Phthalate	84-69-5	$C_{16}H_{22}O_4$	[M+H]+	279.1591	149.0228	21.55	NA	NA	NA	NA	NA
Dimethyl Phthalate	131-11-3	$C_{10}H_{10}O_4$	[M+H]+	195.0652	163.0383	13.18	102.9%	100.1%	87.2%	0.1 – 0.1	0.9984
Diphenylphosphinic Acid *	1707-03-5	$C_{12}H_{11}O_2P$	[M+H]+	219.0569	159.0212	13.07	102.5%	91.8%	74.6%	50 – 67	0.9984
Ethylparaben	120-47-8	$C_9H_{10}O_3$	[M+H]+	167.0703	105.0328	13.85	102.9%	94.2%	48.3%	50 – 104	0.9971
Icaridin	119515-38-7	$C_{12}H_{23}NO_3$	[M+H]+	230.1751	130.1221	16.73	42.8%	97.4%	152.2%	3.3 – 5.0	0.9998
Indole-4-carboxaldehyde *	1074-86-8	C_9H_7NO	[M+H]+	146.0600	118.0648	11.47	77.6%	100.6%	59.9%	10 – 17	0.9982
Isopropylparaben	4191-73-5	$C_{10}H_{12}O_3$	[M+H]+	181.0859	139.0385	15.45	108.5%	62.3%	61.3%	50 – 82	0.9929
Melamine	108-78-1	$C_3H_6N_6$	[M+H]+	127.0727	113.0184	1.64	50.6%	9.8%	5.6%	0.2 – 3.6	0.9913
N-Butylbenzenesulfonamide *	3622-84-2	$C_{10}H_{15}NO_2S$	[M+H]+	214.0896	158.0268	15.08	94.7%	89.3%	77.2%	5.0 – 6.5	0.9988
DEET	134-62-3	$C_{12}H_{17}NO$	[M+H]+	192.1383	119.0484	15.75	92.2%	115.7%	102.2%	0.2 – 0.2	0.9988
Osthole *	484-12-8	$C_{15}H_{16}O_3$	[M+H]+	245.1172	189.0544	19.30	89.1%	92.6%	96.4%	5.0 – 5.2	0.9970
Perfluorobutanoic Acid (PFBA)	375-22-4	$C_4HF_7O_2$	[M-H]-	212.9792	168.9893	9.80	267.4%	100.6%	289.1%	8.6 – 25	0.9879
Perfluoroheptanoic Acid (PFHpA)	375-85-9	$C_7HF_{13}O_2$	[M-H]-	362.9696	318.9797	18.12	127.5%	134.4%	262.6%	10 – 25	0.9935
Perfluorohexanoic Acid (PFHxA)	307-24-4	$C_6HF_{11}O_2$	[M-H]-	312.9728	268.9829	16.24	139.8%	137.1%	263.2%	9.5 – 25	0.9919
Perfluorononanoic Acid (PFNA)	375-95-1	$C_9HF_{17}O_2$	[M-H]-	462.9632	218.9861	21.13	117.3%	149.0%	248.6%	4.0 – 10	0.9907
Perfluorooctanesulfonic Acid (PFOS)	1763-23-1	$C_8HF_{17}O_3S$	[M-H]-	498.9302	168.9893	21.10	41.9%	170.1%	1667.6%	1.5 – 25	0.9758
Perfluoropentanoic Acid (PFPeA)	2706-90-3	$C_5HF_9O_2$	[M-H]-	262.9760	218.9862	13.82	162.8%	119.7%	259.7%	10 – 25	0.9931
Propylparaben	94-13-3	$C_{10}H_{12}O_3$	[M-H]-	179.0714	137.0248	15.80	93.4%	189.8%	197.4%	5.1 – 10	0.9940
Sucralose	56038-13-2	$C_{12}H_{19}Cl_3O_8$	[M+FA-H]-	441.0128	395.0071	9.24	156.6%	220.3%	22.4%	10 – 45	0.9991
Tributyl Phosphate	126-73-8	$C_{12}H_{27}O_4P$	[M+H]+	267.1720	98.9837	21.62	160.0%	48.4%	71.6%	10 – 14	0.9937

Compound Name	CAS	Molecular Formula	Adduct	Exact Mass (*m/z*)	Diagnostic Fragment (*m/z*)	RT (min)	SPE Recovery	Ion Suppression	Matrix Factor	LOQs (ng/L)	R^2
Triclocarban	101-20-2	$C_{13}H_9Cl_3N_2O$	[M+H]+	314.9853	161.9865	21.35	64.0%	78.3%	58.9%	25 – 42	0.9937
Triclosan	3380-34-5	$C_{12}H_7Cl_3O_2$	[M-H]-	286.9439	130.3132	21.94	119.6%	66.0%	91.0%	10 – 11	0.9962
Triisopropanolamine *	122-20-3	$C_9H_{21}NO_3$	[M+H]+	192.1594	174.1497	1.62	66.7%	61.8%	52.5%	25 – 48	0.9908
Tris(1,3-dichloro-2-propyl) Phosphate	13674-87-8	$C_9H_{15}Cl_6O_4P$	[M+H]+	428.8912	208.9524	20.19	105.0%	77.5%	68.2%	50 – 73	0.9955
Tris(2-chloroethyl) Phosphate	115-96-8	$C_6H_{12}Cl_3O_4P$	[M+H]+	284.9612	222.9680	14.02	100.1%	74.6%	78.2%	10 – 13	0.9942
1H-Benzotriazole-5-carboxylic Acid	23814-12-2	$C_7H_5N_3O_2$	[M+H]+	164.0455	108.0440	7.36	NA	NA	NA	NA	NA
1-Methyl-1H-benzotriazole	13351-73-0	$C_7H_7N_3$	[M+H]+	134.0713	106.0646	10.06	NA	NA	NA	NA	NA
Carbanilide	102-07-8	$C_{13}H_{12}N_2O$	[M+H]+	213.1022	94.0646	15.01	70.9%	97.2%	76.7%	10 – 13	0.9981
Galaxolidone *	507442-49-1	$C_{18}H_{24}O_2$	[M+H]+	273.1849	240.1500	21.86	87.6%	92.8%	79.2%	10 – 13	0.9963

Table S9. SPE-LC-HRMS method performance for 385 suspect and nontarget OMPs (continued)

“RT” = retention time; “LOQs” = the limits of quantification in HPLC grade water and Onondaga Lake water; “R^2” = the coefficient of determination of the non-weighted linear least squares regression for a 12-point calibration curve; “PHAR” = pharmaceutical; “PEST” = pesticide; “PCHI” = personal care, household and industrial chemical; “TP” = transformation product. OMPs *without* an asterisk (“*”) denote those prioritized via suspect screening and confirmed or rejected by authentic reference standards. OMPs *with* an asterisk (“*”) denote those prioritized via nontarget screening and confirmed or rejected by authentic reference standards.

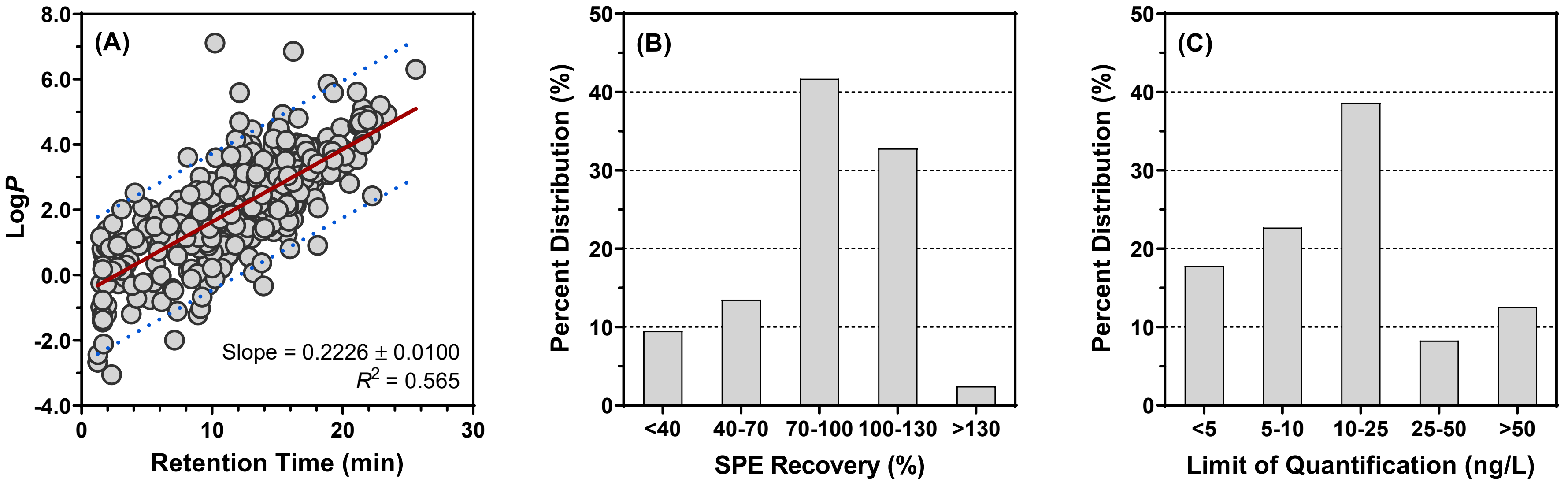

Figure S9. SPE-LC-HRMS method performance for suspect and nontarget OMPs: (A) Correlation between the chromatographic retention times of OMPs (n = 385) and the Log$P$ of OMPs predicted by *OPERA 2.6*.[31] The red solid line represents the linear regression line. The blue dotted lines represent the 95% confidence interval of the linear regression line. (B) Percent distribution of the absolute SPE recoveries (at the spike level of 100 ng/L in Onondaga Lake water) of OMPs (n = 385). (C) Percent distribution of the average limits of quantification for OMPs (n = 326 with SPE recoveries) in HPLC grade water and Onondaga Lake water.

**Table S10.** LC-HRMS method performance for isotope-labeled internal standards						
Compound Name	CAS	Molecular Formula	Adduct	Exact Mass (*m/z*)	RT (min)	%RSD
3,4-Methylenedioxymethamphetamine-d_5	136765-43-0	$C_{11}H_{10}D_5NO_2$	[M+H]+	199.1489	7.97	5.8
Acetaminophen-d_3	60902-28-5	$C_8H_6D_3NO_2$	[M+H]+	155.0894	3.56	9.4
Amphetamine-d_{10}	169565-17-7	$C_9H_3D_{10}N$	[M+H]+	146.1748	7.09	6.0
Atenolol-d_7	1202864-50-3	$C_{14}H_{15}D_7N_2O_3$	[M+H]+	274.2143	3.12	11.1
Buprenorphine-d_4	136781-89-0	$C_{29}H_{37}D_4NO_4$	[M+H]+	472.3359	13.15	4.1
Bupropion-d_9	1189725-26-5	$C_{13}H_9D_9ClNO$	[M+H]+	249.1715	11.78	4.4
Caffeine-d_9	72238-85-8	$C_8HD_9N_4O_2$	[M+H]+	204.1441	8.37	7.2
Carbamazepine-d_{10}	132183-78-9	$C_{15}H_2D_{10}N_2O$	[M+H]+	247.1650	14.54	13.5
Cimetidine-d_3	1185237-29-9	$C_{10}H_{13}D_3N_6S$	[M+H]+	256.1418	3.28	10.9
Codeine-d_3	70420-71-2	$C_{18}H_{18}D_3NO_3$	[M+H]+	303.1783	4.67	11.3
Cotinine-d_3	110952-70-0	$C_{10}H_9D_3N_2O$	[M+H]+	180.1211	1.84	8.0
Diclofenac-d_4	153466-65-0	$C_{14}H_7D_4Cl_2NO_2$	[M-H]-	298.0345	19.89	8.1
Diphenhydramine-d_3	170082-18-5	$C_{17}H_{18}D_3NO$	[M+H]+	259.1884	13.21	4.1
Dopamine-d_4	203633-19-6	$C_8H_7D_4NO_2$	[M+H]+	158.1114	1.69	NA
Ephedrine-d_3	285979-73-9	$C_{10}H_{12}D_3NO$	[M+H]+	169.1414	5.02	10.4
Fentanyl-d_5	118357-29-2	$C_{22}H_{23}D_5N_2O$	[M+H]+	342.2588	12.36	7.3
Fluconazole-$^{13}C_3$	NA	$C_{10}{}^{13}C_3H_{12}F_2N_6O$	[M+H]+	310.1214	11.00	8.7
Gabapentin-d_{10}	1126623-20-8	$C_9H_7D_{10}NO_2$	[M+H]+	182.1960	7.14	10.0
Gemfibrozil-d_6	1184986-45-5	$C_{15}H_{16}D_6O_3$	[M+H]+	257.2018	21.86	8.1
Hydrocodone-d_3	136765-36-1	$C_{18}H_{18}D_3NO_3$	[M+H]+	303.1783	6.96	5.9
Hydromorphone-d_3	136765-37-2	$C_{17}H_{16}D_3NO_3$	[M+H]+	289.1626	2.42	6.8
Lamotrigine-^{13}C,$^{15}N_4$	NA	$^{13}C_1C_8H_7Cl_2N_1{}^{15}N_4$	[M+H]+	261.0066	10.87	8.8
Levetiracetam-d_6	1435933-72-4	$C_8H_8D_6N_2O_2$	[M+H]+	177.1505	5.94	12.1
Lidocaine-d_{10}	851528-09-1	$C_{14}H_{12}D_{10}N_2O$	[M+H]+	245.2433	8.93	10.4
Meperidine-d_4	53484-73-4	$C_{15}H_{17}D_4NO_2$	[M+H]+	252.1896	11.21	5.3
Metaxalone-d_6	NA	$C_{12}H_9D_6NO_3$	[M+H]+	228.1501	15.11	NA
Metformin-d_6	1185166-01-1	$C_4H_5D_6N_5$	[M+H]+	136.1464	1.60	12.4
Methadone-d_3	60263-63-0	$C_{21}H_{24}D_3NO$	[M+H]+	313.2354	15.12	12.2
Methamphetamine-d_8	136765-40-7	$C_{10}H_7D_8N$	[M+H]+	158.1779	7.77	12.0
Methocarbamol-d_3	1346600-86-9	$C_{11}H_{12}D_3NO_5$	[M+H]+	245.1211	11.19	12.3
Metoprolol-d_7	1219798-61-4	$C_{15}H_8D_7NO_3$	[M+H]+	275.2347	10.62	10.7
Morphine-d_3	67293-88-3	$C_{17}H_{16}D_3NO_3$	[M+H]+	289.1626	1.99	5.2
Naproxen-d_3	958293-77-1	$C_{14}H_{11}D_3O_3$	[M+H]+	234.1204	17.23	11.7
Nicotine-d_4	350818-69-8	$C_{10}H_{10}D_4N_2$	[M+H]+	167.1481	1.66	15.7
Oxycodone-d_3	160227-46-3	$C_{18}H_{18}D_3NO_4$	[M+H]+	319.1732	6.12	11.6
Oxymorphone-d_3	145225-03-2	$C_{17}H_{16}D_3NO_4$	[M+H]+	305.1575	2.12	4.2
Phendimetrazine-d_5	NA	$C_{12}H_{12}D_5NO$	[M+H]+	197.1697	6.90	6.6
Phentermine-d_5	1330236-21-9	$C_{10}H_{10}D_5N$	[M+H]+	155.1591	9.24	10.0
Pregabalin-d_6	NA	$C_8H_{11}D_6NO_2$	[M+H]+	166.1709	7.01	15.4
Protriptyline-d_3	1435934-21-6	$C_{19}H_{18}D_3N$	[M+H]+	267.1935	15.11	13.2
Sulfamethoxazole-d_4	1020719-86-1	$C_{10}H_7D_4N_3O_3S$	[M+H]+	258.0845	9.68	9.7
Tramadol-^{13}C,d_3	NA	$^{13}C_1C_{15}H_{22}D_3NO_2$	[M+H]+	268.2180	10.31	9.8
Trimethoprim-d_9	1189460-62-5	$C_{14}H_9D_9N_4O_3$	[M+H]+	300.2017	8.36	9.2
Venlafaxine-d_6	1062606-12-5	$C_{17}H_{21}D_6NO_2$	[M+H]+	284.2491	12.51	10.1
2-Ethylidene-1,5-dimethyl-3,3-diphenylpyrrolidine-d_3	136765-23-6	$C_{20}H_{21}D_3N$	[M]+	281.2092	13.08	9.4
3-Hydroxy Cotinine-d_3	159956-78-2	$C_{10}H_9D_3N_2O_2$	[M+H]+	196.1160	1.63	12.2
Benzoylecgonine-d_3	115732-68-8	$C_{16}H_{16}D_3NO_4$	[M+H]+	293.1575	9.78	12.5
Ecgonine Methyl Ester-d_3	136765-34-9	$C_{10}H_{14}D_3NO_3$	[M+H]+	203.1470	1.63	7.4

**Table S10.** LC-HRMS method performance for isotope-labeled internal standards (continued)						
Compound Name	CAS	Molecular Formula	Adduct	Exact Mass (*m/z*)	RT (min)	%RSD
Hydroxybupropion-d_6	1184984-06-2	$C_{13}H_{12}D_6ClNO_2$	[M+H]+	262.1475	11.22	11.4
Norcodeine-d_3	NA	$C_{17}H_{16}D_3NO_3$	[M+H]+	289.1626	4.98	5.9
Norfentanyl-d_5	1211527-23-9	$C_{14}H_{15}D_5N_2O$	[M+H]+	238.1962	10.05	7.3
Norhydrocodone-d_3	NA	$C_{17}H_{16}D_3NO_3$	[M+H]+	289.1626	7.24	7.8
Noroxycodone-d_3	1426174-79-9	$C_{17}H_{16}D_3NO_4$	[M+H]+	305.1575	15.73	9.9
Ritalinic Acid-d_{10}	NA	$C_{13}H_7D_{10}NO_2$	[M+H]+	230.1960	9.76	8.0
2,4-Dichlorophenoxyacetic Acid-d_3 (2,4-D-d_3)	202480-67-9	$C_8H_3D_3Cl_2O_3$	[M-H]-	221.9810	16.06	10.9
Atrazine-d_5	163165-75-1	$C_8H_9D_5ClN_5$	[M+H]+	221.1324	15.40	7.4
Clofibric Acid-d_4	1184991-14-7	$C_{10}H_7D_4ClO_3$	[M-H]-	217.0575	17.04	10.2
Diuron-d_6	1007536-67-5	$C_9H_4D_6Cl_2N_2O$	[M+H]+	239.0620	15.88	12.3
Imidacloprid-d_4	1015855-75-0	$C_9H_6D_4ClN_5O_2$	[M-H]-	258.0701	9.93	8.6
Mecoprop-d_3	352431-15-3	$C_{10}H_8D_3ClO_3$	[M-H]-	216.0512	17.96	9.9
Metalaxyl-d_3	NA	$C_{15}H_{18}D_3NO_4$	[M+H]+	283.1732	15.73	10.8
Metolachlor-d_6	1219803-97-0	$C_{15}H_{16}D_6ClNO_2$	[M+H]+	290.1788	19.07	6.6
Prometon-d_3	1219803-43-6	$C_{10}H_{16}D_3N_5O$	[M+H]+	229.1851	13.38	7.0
Benzotriazole-d_4	1185072-03-0	$C_6HD_4N_3$	[M+H]+	124.0807	9.04	11.4
N,N-Diethyl-3-methyl-d_3-benzamide-2,4,5,6-d_4 (DEET-d_7)	1219799-37-7	$C_{12}H_{10}D_7NO$	[M+H]+	199.1822	15.72	8.7
Oxybenzone-d_5	1219798-54-5	$C_{14}H_7D_5O_3$	[M+H]+	234.1173	19.00	11.0
Sucralose-d_6	1459161-55-7	$C_{12}H_{13}D_6Cl_3O_8$	[M+FA-H]-	447.0504	9.17	10.3
Triclosan-d_3	1020719-98-5	$C_{12}H_4D_3Cl_3O_2$	[M-H]-	289.9627	21.87	5.1
Perfluorooctanoic Acid-$^{13}C_8$	1350614-84-4	$^{13}C_8HF_{15}O_2$	[M-H]-	420.9932	19.76	9.2
Sodium Perfluorooctanesulfonate-$^{13}C_8$	NA	$^{13}C_8HF_{17}O_3SNa$	[M-H]-	506.9570	21.16	21.1
N-Methylperfluorooctanesulfonamidoacetic Acid-d_3	1400690-70-1	$C_{11}H_3D_3F_{17}NO_4S$	[M-H]-	572.9861	23.09	8.1

"RT" = retention time; "%RSD" = the percent relative standard deviation of the isotope-labeled internal standard peak areas in calibration standards analyzed over the project period; "NA" = not available.

S5. Mass balance modeling of OMPs in Onondaga Lake

For 54 wastewater-derived and mixed-source OMPs (i.e., those with a >80% detection frequency in the vertical profile samples), their vertical concentration profiles in Onondaga Lake were simulated using the *lake compartment* module in *AQUASIM 2.1g*.[32, 33] *AQUASIM* is a computer program developed to model the physical mixing and elimination processes of chemical and biological substances in natural and engineered aquatic systems.[34] Onondaga Lake was meshed into 72 one-dimensional horizontal layers of 0.25-m thickness, for which all variables were averaged over horizontal cross sections and vertical mixing was modeled by the depth-dependent turbulent diffusion coefficients (K_z; m^2 d^{-1}) derived from the vertical profiles of lake water temperature:[35]

$$K_z = \begin{cases} \min\left(\alpha\left(N^2\right)^{-\gamma}, K_{z\_max}\right) & \text{for } N^2 > 0 \\ K_{z\_\max} & \text{for } N^2 \leq 0 \end{cases} \quad \text{(S1)}$$

where $K_{z\_\max}$ (i.e., 0.5616 m^2/d) is the maximum turbulent diffusion coefficient previously determined for Onondaga Lake,[36] γ (i.e., 0.43) is a coefficient that reflects the mode of turbulence production,[35] α is a coefficient that reflects the general level of turbulence and can be derived from the lake surface area (A_s; m^2) using the following empirical equation:[35]

$$\alpha = 8.17\times10^{-4} A_s^{0.56} \quad \text{(S2)}$$

and N^2 is the Brunt-Väisälä frequency (d^{-2}; also known as the stability frequency) defined as below:[34]

$$N^2 = -\frac{g}{\rho}\frac{\partial\rho}{\partial z} \quad \text{(S3)}$$

where g (m^3 kg^{-1} s^{-2}) is the gravitational acceleration, ρ (kg m^{-3}) is the density of lake water corrected for temperature and salinity, and z (m) is the lake depth.

For each OMP, two sets of *AQUASIM* simulations were implemented to simulate the vertical concentration profiles of OMPs in Onondaga Lake under different thermal stratification conditions. On July 25, 2017, varying fractions of the negatively buoyant inflows (i.e., those with a density greater than that of the epilimnetic water) from the regional WWTP outfall and the four tributaries plunged into the metalimnion as interflows.[37-40] Using

the specific conductance data measured *in situ*, the fraction of each inflow entering the metalimnion in July was estimated to be 40%, 10%, 30%, 10%, and 10% for the WWTP effluent, Ninemile Creek (T1), Onondaga Creek (T2), Harbor Brook (T3), and Ley Creek (T4), respectively. On October 16, 2017, 100% of the inflows were estimated to enter the well-mixed epilimnion (i.e., as overflows over the metalimnion). Note that previous research has observed an unusual bidirectional flow regime at the lake outlet during low flow periods;[41] however, such bidirectional flow conditions did not exist on our sampling dates given the uniform vertical profiles of temperature and specific conductance measured at site L4. For a subset of nine OMPs, simulated photolysis experiments were also conducted to estimate field-relevant photolysis rate constants in the epilimnion of Onondaga Lake (see below) as process inputs for *AQUASIM*.

Measurements of model performance were evaluated by two quantitative metrics proposed for benchmarking the performance of hydrological models.[42, 43] Specifically, the percent bias (PBIAS) and the Nash-Sutcliffe efficiency (NSE) were calculated using the following two equations, respectively:[42, 43]

$$\mathrm{PBIAS} = \left[\frac{\sum_{i=1}^{18}\left(c_i^{\mathrm{measured}} - c_i^{\mathrm{simulated}}\right) \times 100\%}{\sum_{i=1}^{18}\left(c_i^{\mathrm{measured}}\right)}\right] \tag{S4}$$

$$\mathrm{NSE} = 1 - \left[\frac{\sum_{i=1}^{18}\left(c_i^{\mathrm{measured}} - c_i^{\mathrm{simulated}}\right)^2}{\sum_{i=1}^{18}\left(c_i^{\mathrm{measured}} - \bar{c}_i\right)^2}\right] \tag{S5}$$

where c_i^{measured} (ng/L) is the measured OMP concentration at a given depth (z = 1 – 18 m at 1-m intervals), $c_i^{\mathrm{simulated}}$ (ng/L) is the simulated OMP concentration at a given depth (z = 0 – 18 m at 0.25-m intervals), and $\bar{c}_i$ (ng/L) is the mean of OMP concentrations measured at all depths.

For specific conductance, the one-dimensional flushing model yielded a PBIAS value of 1.2% and a NSE value of 0.84 for the July vertical profile and a PBIAS value of 1.3% and a NSE value of 0.66 for the October vertical profile, respectively.

Six wastewater-derived (i.e., carbamazepine, fluconazole, gabapentin, lamotrigine, lidocaine, and sulfamethoxazole) and three mixed-source OMPs (i.e., caffeine, atrazine, and metolachlor) were selected for steady-state photolysis experiments under simulated sunlight conditions. Onondaga Lake water samples (absorbance <0.05 at all wavelengths >290 nm with an overall light screening factor (λ = 290-400 nm) of 0.98) and buffered ultrapure water (5 mM borate; pH=8.0±0.05) were spiked with 2 μg/L (chosen to ensure field relevance[44]) of individual OMPs and irradiated in quartz test tubes (100 mm × 11 mm i.d.; held at ~30° from the horizontal) inside an Atlas Suntest XLS+(II) solar simulator equipped with a 1700 W xenon arc lamp, a daylight glass 300 nm UV filter, and an Atlas SunCool chiller for chamber temperature control at 25±1 °C. Over the course of irradiation, subsamples were withdrawn from quartz test tubes at predetermined time intervals and analyzed for OMPs using a tandem online SPE and LC-HRMS method adapted from our previous work.[45] Briefly, 1-mL of subsamples were loaded from a stainless steel sample loop onto a Hypersil GOLD aQ C18 trap column (20 × 2.1 mm i.d., 12 μm particle size), which was washed with acidified water (amended with 0.1% v/v formic acid) and subsequently eluted with the analytical pump gradient to a Hypersil GOLD C18 analytical column (100 × 2.1 mm i.d., 3 μm particle size) running acidified water and methanol as the mobile phases (both amended with 0.1% v/v formic acid). The trap and analytical columns were then re-equilibrated to their starting conditions prior to the next injection. Chromatographic separation and mass spectrometric analysis were performed following the instrument settings in **Table S5**. For each set of photolysis experiments, duplicate dark controls (foil-wrapped) and PNA/pyr actinometer solutions were also irradiated along with samples to monitor the nonphotochemical loss of OMPs and the incident light intensity, respectively. PNA was analyzed by an Agilent 1260 Infinity II high-performance liquid chromatograph with a variable wavelength detector.[46]

For each OMP, the depth-dependent photolysis rate constants in the epilimnion of Onondaga Lake, $k_{\mathrm{OMP\_epi}}$ (d^{-1}), were approximated as:

$$k_{\mathrm{OMP\_epi}} = k^0_{\mathrm{OMP}} \left(\frac{\sum_{\lambda\,=\,290\ \mathrm{nm}}^{400\ \mathrm{nm}} Z_{\lambda,\,24\ \mathrm{h}} e^{-K_{\mathrm{d},\,\lambda} z_{\mathrm{epi}}}}{\sum_{\lambda\,=\,290\ \mathrm{nm}}^{400\ \mathrm{nm}} I_{\lambda} z} \right) \quad \text{(S6)}$$

where k^0_{OMP} (d^{-1}) is the pseudo-first order rate constant for OMP photolysis measured in the quartz test tube under simulated sunlight conditions, $Z_{\lambda,\,24\,h}$ the daily average solar irradiance (10^{-3} mol-photons cm^{-2} s^{-1} nm^{-1}) modeled for July 25 and October 16, 2017 using the Simple Model of the Atmospheric Radiative Transfer of Sunshine (SMARTS)[47, 48] with adjustments made for reflection off the water's surface and the increased pathlength within the water column,[49-51] $K_{d,\,\lambda}$ (m^{-1}) is the diffuse attenuation coefficient in Onondaga Lake estimated based on the empirical relationship $K_{d,\,\lambda} = \exp(-0.01347\lambda + 5.36\,\mathrm{DOC}^{0.157})$,[52] z_{epi} is the epilimnion depth at which the attenuated solar irradiance is being calculated, z (i.e., 1.12 cm) is the optical pathlength for the quartz test tube, and I_λ (10^{-3} mol-photons cm^{-3} s^{-1} nm^{-1}) is the incident light intensity per unit volume approximated by multiplying the fractional spectral intensity of the xenon arc lamp, ρ_λ (nm^{-1}), with the total incident light intensity from 290 to 400 nm per unit volume, I_0 (mol-photons L^{-1} s^{-1}):[46]

$$I_0 = \frac{k_{PNA}}{2.303 z \Phi_{PNA} \sum_{\lambda = 290\,nm}^{400\,nm} \rho_\lambda \varepsilon_\lambda} \tag{S7}$$

where k_{PNA} (s^{-1}) is the pseudo-first order rate constant for the loss of PNA, Φ_{PNA} (1.74×10^{-3} mol mol-photons^{-1}; calculated from $\Phi_{PNA} = 0.29\,[\mathrm{pyr}] + 0.00029$)[53] is the quantum yield for the loss of PNA at a given pyridine concentration (i.e., [pyr] = 5×10^{-3} M), and ε_λ (M^{-1} cm^{-1}) is the decadic molar absorption coefficient of PNA.[53]

Out of the nine OMPs investigated, eight underwent faster photolysis in Onondaga Lake water samples than in buffered ultrapure water under simulated sunlight conditions (based on the measurements of k^0_{OMP}), suggesting the relevance of indirect photolysis for OMP transformation. Lamotrigine was the only compound that primarily underwent direct photolysis. Note that k_{OMP_epi} only served to approximate the OMP photolysis rate constants in the epilimnion of Onondaga Lake during the sampling period (**Figure S10**). More rigorous assessments of OMP photolysis in the lake would require the investigation of *in situ* irradiation and light attenuation conditions, the measurements of direct photolysis quantum yields for OMPs, the predictions of depth-dependent steady-state concentrations of photochemically produced reactive intermediates, and the determination of second-order reaction rate constants of reactive intermediates with OMPs.[44, 54, 55]

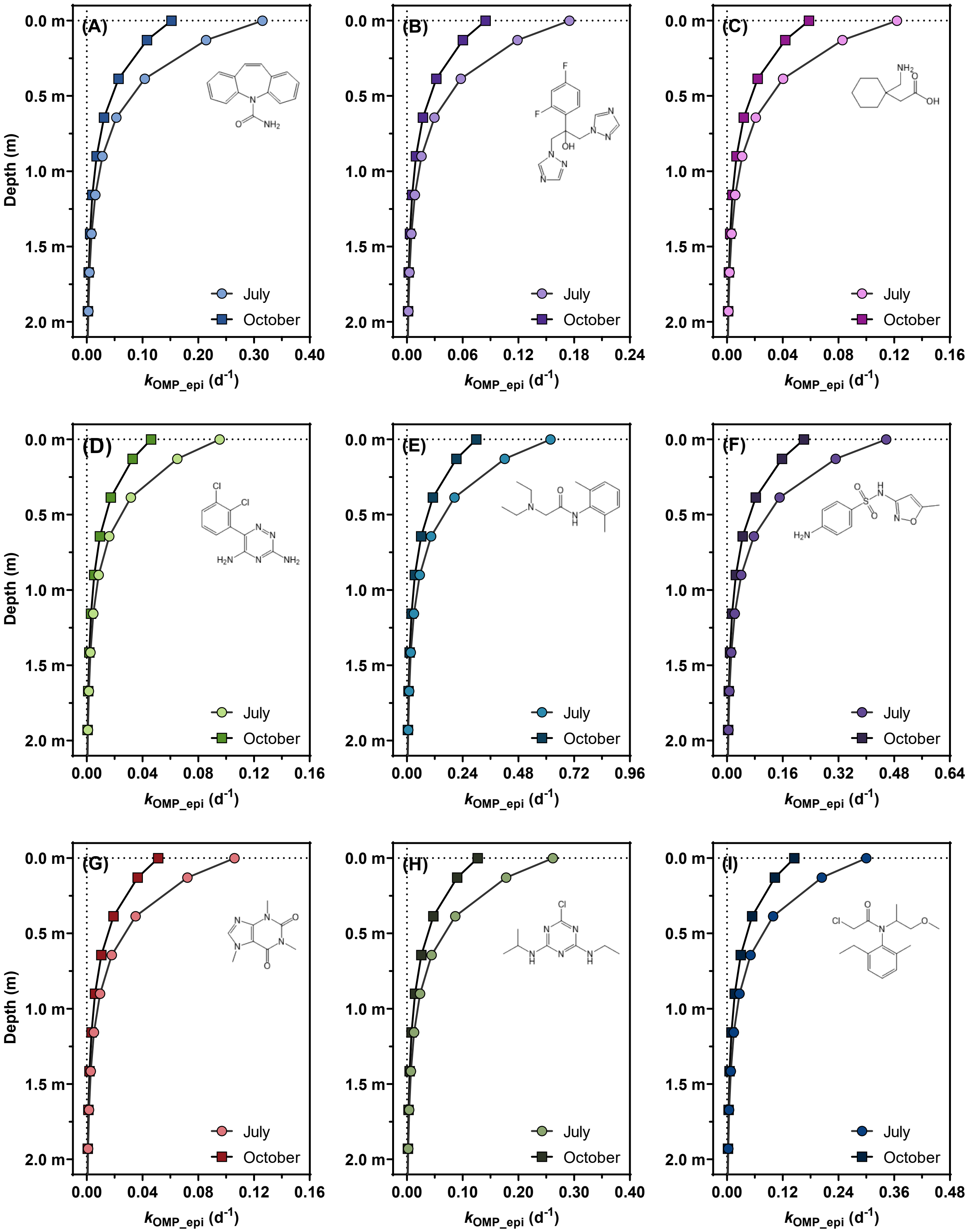

Figure S10. Predicted photolysis rate constants ($k_{\mathrm{OMP\_epi}}$) of nine selected OMPs in Onondaga Lake as a function of depth (down to 2 m): (A) Carbamazepine; (B) Fluconazole; (C) Gabapentin; (D) Lamotrigine; (E) Lidocaine; (F) Sulfamethoxazole; (G) Caffeine; (H) Atrazine; and (I) Metolachlor.

S6. Mass balance modeling of OMPs in the Onondaga Lake-Three Rivers system

For 45 wastewater-derived and mixed-source OMPs (i.e., those with a >80% detection frequency at gauged sites in the Onondaga Lake-Three Rivers system), their mass flows were determined using the *river section compartment* module in *AQUASIM 2.1g*. The Three Rivers were divided into three one-dimensional reaches, for which all variables were averaged over the river cross section and the friction slope (S_f) was calculated by:[34]

$$S_f = \frac{1}{K_{st}^2} \times \left(\frac{P}{A}\right)^{4/3} \left(\frac{\bar{Q}}{A}\right)^2 \quad \text{(S8)}$$

where K_{st} ($m^{1/3}/s$; converted to $m^{1/3}/d$ by multiplying with 86,400 s) is Strickler friction coefficient, P (m) is the wetted perimeter, A (m^2) is the river cross-sectional area, and $\bar{Q}$ (m^3/d) is the daily mean discharge measured at the gauged site. For any given reach, the river water depth was calculated as the difference between the water level elevation and the riverbed elevation.[38] Reach 1 (from site R294 to L4) was configured with an upstream input from the Seneca River. Reach 2 (from site L4 to R212) was linked to the downstream end of Reach 1 and configured with an upstream input from Onondaga Lake. Reach 3 (from site R212 to R6) was linked to the downstream end of Reach 2 and configured with an upstream input from the Oneida River and lateral input along the Oswego River reach.

Given that the outlet of Onondaga Lake (site L4) was not actively monitored by any gauge during the sampling period, the lake outflow ($\bar{Q}_{L4}$; m^3/d) was estimated as the sum of the lake inflows, including the WWTP effluent ($\bar{Q}_{WWTP}$; m^3/d), Ninemile Creek ($\bar{Q}_{T1}$; m^3/d), Onondaga Creek ($\bar{Q}_{T2}$; m^3/d), Harbor Brook ($\bar{Q}_{T3}$; m^3/d), and Ley Creek ($\bar{Q}_{T4}$; m^3/d), as described in previous studies.[1, 41] Using historical discharge data from 2004 to 2009 (during which the lake outlet was gauged), a linear relationship between the sum of lake inflows and the lake outflow was established as follows:

$$\Sigma(\bar{Q}_{T1} + \bar{Q}_{T2} + \bar{Q}_{T3} + \bar{Q}_{T4} + \bar{Q}_{WWTP}) = (0.978 \pm 0.060)\, \bar{Q}_{L4} - (15.52 \pm 33.92);\ R^2 = 0.782;\ n = 77 \quad \text{(S9)}$$

$\bar{Q}_{L4}$ was calculated for each sampling date in 2017 using the above equation given the lake inflows measured in 2017.

For each OMP, two sets of *AQUASIM* simulations were implemented to calculate the input loads (L_{input}; g/d) contributed by Onondaga Lake (L_{Onondaga}; g/d), the Seneca River (L_{Seneca}; g/d), the Oneida River (L_{Oneida}; g/d), and the Oswego River reach (L_{Oswego}; g/d), respectively, as well as the output loads (L_{output}; g/d) at the Oswego River mouth prior to entry into Lake Ontario. Similar to previous OMP studies in river and lake systems,[56, 57] the ratios of L_{input} to L_{output} were computed to evaluate to what degree unidentified sources contributed additional OMP loads to the Onondaga Lake-Three Rivers system (i.e., if $L_{\mathrm{input}}/L_{\mathrm{output}}$ was <1) or transformations or loss of OMPs occurred during the riverine transport to Lake Ontario (i.e., if $L_{\mathrm{input}}/L_{\mathrm{output}}$ was >1). The percentages of L_{Onondaga}, L_{Seneca}, L_{Oneida}, and L_{Oswego} to L_{output} were calculated to quantify the fractional contributions associated with respective hydrologic components. Comparing the system inflow ($\overline{Q}_{\mathrm{input}}$; m^3/d; $\Sigma(\overline{Q}_{\mathrm{L4}} + \overline{Q}_{\mathrm{R294}} + \overline{Q}_{\mathrm{R212}} + \overline{Q}_{\mathrm{R6}} - \overline{Q}_{\mathrm{R10}})$) with the system outflow ($\overline{Q}_{\mathrm{output}}$; m^3/d) supported the assumption that no other substantial sources of flow existed in the lake-river system during the sampling period as the ratio of $\overline{Q}_{\mathrm{input}}$ to $\overline{Q}_{\mathrm{output}}$ was 0.99±0.03 on July 11 and October 16, 2017.

S7. Concentration ranges and detection frequencies of OMPs

Table S11. Concentration ranges and detection frequencies of OMPs in samples from the Onondaga Lake-Three Rivers system

Compound Name	Category[a]	Minimum (ng/L)	Mean (ng/L)	Median (ng/L)	Maximum (ng/L)	Detection Frequency (n = 143)	Cluster[b]	Group
Aliskiren	PHAR	13	22	21	39	8	A	Wastewater-derived
Amantadine	PHAR	5	11	9	45	88	A	Wastewater-derived
Amitriptyline	PHAR	15	16	15	20	8	A	Wastewater-derived
Atenolol	PHAR	11	19	13	132	94	A	Wastewater-derived
Azelaic Acid	PHAR	117	324	302	624	9	D	Mixed-source
Azithromycin	PHAR	144	183	183	205	8	A	Wastewater-derived
Bupropion	PHAR	31	81	73	136	8	A	Wastewater-derived
Butalbital	PHAR	57	139	97	355	64	D	Mixed-source
Caffeine	PHAR	1	52	34	469	131	C	Mixed-source
Carbamazepine	PHAR	11	27	19	274	139	A	Wastewater-derived
Celecoxib	PHAR	23	43	32	110	8	A	Wastewater-derived
Cetirizine	PHAR	12	45	23	580	127	A	Wastewater-derived
Citalopram	PHAR	12	32	19	81	8	A	Wastewater-derived
Clarithromycin	PHAR	33	65	55	144	8	A	Wastewater-derived
Clindamycin	PHAR	35	91	100	154	8	A	Wastewater-derived
Desvenlafaxine (O-Desmethyl Venlafaxine)	PHAR	11	30	16	188	43	A	Wastewater-derived
Dextromethorphan	PHAR	4	11	8	21	8	A	Wastewater-derived
Diltiazem	PHAR	21	30	28	44	8	A	Wastewater-derived
Diphenhydramine	PHAR	13	35	30	98	8	A	Wastewater-derived
Dopamine	PHAR	21	44	31	250	102	A	Wastewater-derived
Doxylamine	PHAR	32	32	32	32	1	A	Wastewater-derived
Ephedrine	PHAR	14	17	16	24	8	A	Wastewater-derived
Fexofenadine	PHAR	10	115	110	1022	115	A	Wastewater-derived
Flecainide	PHAR	10	33	30	62	8	A	Wastewater-derived
Fluconazole	PHAR	20	32	26	152	99	A	Wastewater-derived
Gabapentin	PHAR	8	98	51	1499	141	A	Wastewater-derived
Labetalol	PHAR	34	58	57	104	8	A	Wastewater-derived
Lamotrigine	PHAR	16	161	143	1245	143	A	Wastewater-derived
Levamisole	PHAR	10	29	15	123	8	A	Wastewater-derived
Levetiracetam	PHAR	10	25	15	183	126	A	Wastewater-derived
Levorphanol	PHAR	18	72	47	212	13	A	Wastewater-derived
Lidocaine	PHAR	7	37	17	916	139	A	Wastewater-derived
Losartan	PHAR	13	39	29	362	129	A	Wastewater-derived
Maprotiline	PHAR	23	26	26	28	1	A	Wastewater-derived
Metaxalone	PHAR	10	21	17	218	111	A	Wastewater-derived
Metformin	PHAR	28	198	92	2621	135	A	Wastewater-derived

Table S11. Concentration ranges and detection frequencies of OMPs in samples from the Onondaga Lake-Three Rivers system (continued)								
Compound Name	Category[a]	Minimum (ng/L)	Mean (ng/L)	Median (ng/L)	Maximum (ng/L)	Detection Frequency (*n* = 143)	Cluster[b]	Group
Methocarbamol	PHAR	10	19	13	204	111	A	Wastewater-derived
Metoprolol	PHAR	11	28	16	314	111	A	Wastewater-derived
Mycophenolic Acid	PHAR	20	39	28	117	8	A	Wastewater-derived
Naproxen	PHAR	11	36	31	355	134	A	Wastewater-derived
Oxcarbazepine	PHAR	13	23	22	40	8	A	Wastewater-derived
Phendimetrazine	PHAR	27	61	53	239	54	D	Mixed-source
Phentermine	PHAR	25	43	41	69	8	A	Wastewater-derived
Phenytoin	PHAR	22	126	75	347	8	A	Wastewater-derived
Pregabalin	PHAR	121	140	138	164	2	A	Wastewater-derived
Primidone	PHAR	19	40	34	73	8	A	Wastewater-derived
Propafenone	PHAR	132	137	137	143	1	A	Wastewater-derived
Propranolol	PHAR	13	20	18	36	8	A	Wastewater-derived
Sitagliptin	PHAR	56	115	111	181	8	A	Wastewater-derived
Sulfamethoxazole	PHAR	9	75	58	768	126	A	Wastewater-derived
Sulfapyridine	PHAR	200	466	490	878	8	A	Wastewater-derived
Theophylline	PHAR	151	204	177	624	49	D	Mixed-source
Trimethoprim	PHAR	3	22	6	995	116	A	Wastewater-derived
Venlafaxine	PHAR	11	24	15	223	111	A	Wastewater-derived
10,11-Dihydro-10-hydroxy Carbamazepine	PHAR TP	11	36	16	608	111	A	Wastewater-derived
2-Ethyl-2-phenylmalonamide	PHAR TP	12	22	19	40	8	A	Wastewater-derived
2-Ethylidene-1,5-dimethyl-3,3-diphenylpyrrolidine (EDDP)	PHAR TP	32	110	91	241	8	A	Wastewater-derived
Benzoylecgonine	PHAR TP	9	13	12	38	119	D	Mixed-source
Carbamazepine-10,11-epoxide	PHAR TP	14	28	19	56	8	A	Wastewater-derived
Hydroxybupropion	PHAR TP	32	66	41	269	29	A	Wastewater-derived
N4-Acetylsulfamethoxazole	PHAR TP	11	65	27	258	19	A	Wastewater-derived
N-Desmethyl Venlafaxine	PHAR TP	26	50	50	77	8	A	Wastewater-derived
O-Desmethyl Tramadol	PHAR TP	13	26	27	41	8	A	Wastewater-derived
Ritalinic Acid	PHAR TP	16	22	18	107	72	B	Mixed-source
(4-Chloro-2-methylphenoxy)acetic Acid	PEST	14	59	48	118	5	A	Wastewater-derived
2,4-Dichlorophenoxyacetic Acid (2,4-D)	PEST	6	70	48	710	120	C	Mixed-source
2-Naphthoxyacetic Acid	PEST	8	35	21	261	78	A	Wastewater-derived
4-Chlorophenoxyacetic Acid	PEST	12	452	63	5615[c]	22	B	Mixed-source
Atrazine	PEST	11	122	95	420	143	B	Mixed-source
Diuron	PEST	11	15	12	98	133	A	Wastewater-derived
Imazapyr	PEST	13	38	24	708	116	C	Mixed-source
Imidacloprid	PEST	19	30	31	39	8	A	Wastewater-derived

**Table S11.** Concentration ranges and detection frequencies of OMPs in samples from the Onondaga Lake-Three Rivers system (continued)								
Compound Name	Category[a]	Minimum (ng/L)	Mean (ng/L)	Median (ng/L)	Maximum (ng/L)	Detection Frequency (*n* = 143)	Cluster[b]	Group
Mecoprop	PEST	81	129	122	297	15	D	Mixed-source
Metalaxyl	PEST	10	14	13	19	24	B	Mixed-source
Metolachlor	PEST	8	44	29	196	143	B	Mixed-source
Prometon	PEST	7	9	9	30	143	C	Mixed-source
Propazine	PEST	9	14	13	28	105	B	Mixed-source
Thiabendazole	PEST	11	15	15	20	2	A	Wastewater-derived
2-Aminobenzimidazole	PEST TP	16	18	17	34	37	A	Wastewater-derived
Atrazine-2-hydroxy	PEST TP	15	55	43	238	143	B	Mixed-source
Atrazine-desethyl	PEST TP	5	22	19	62	143	B	Mixed-source
Atrazine-desisopropyl	PEST TP	12	20	16	73	143	B	Mixed-source
Metolachlor Ethanesulfonic Acid (Metolachlor ESA)	PEST TP	31	199	119	1262	118	D	Mixed-source
Metolachlor Oxanilic Acid (Metolachlor OA)	PEST TP	24	34	29	170	127	B	Mixed-source
Benzotriazole	PCHI	27	252	214	2480	143	A	Wastewater-derived
2-Hydroxybenzothiazole	PCHI	27	85	67	775	102	D	Mixed-source
Methyl-1H-benzotriazole	PCHI	18	384	354	2228	143	A	Wastewater-derived
Benzophenone	PCHI	16	219	189	732	131	B	Mixed-source
Oxybenzone	PCHI	10	185	69	2299	140	B	Mixed-source
Benzothiazole	PCHI	22	266	209	1506	139	A	Wastewater-derived
Diphenylphosphinic Acid	PCHI	58	103	71	746	109	A	Wastewater-derived
Melamine	PCHI	4	195	85	1208	117	D	Mixed-source
N,N-Diethyl-3-methylbenzamide (DEET)	PCHI	2	40	29	375	108	A	Wastewater-derived
N-Butylbenzenesulfonamide	PCHI	6	39	23	659	138	A	Wastewater-derived
Perfluorobutanoic Acid (PFBA)	PCHI	219	948	842	3250[c]	143	C	Mixed-source
Perfluoroheptanoic Acid (PFHpA)	PCHI	10	26	19	292	143	C	Mixed-source
Perfluorohexanoic Acid (PFHxA)	PCHI	21	97	81	706	143	C	Mixed-source
Perfluorononanoic Acid (PFNA)	PCHI	4	8	7	38	143	C	Mixed-source
Perfluorooctanesulfonic Acid (PFOS)	PCHI	3	250	155	1794	19	A	Wastewater-derived
Perfluoropentanoic Acid (PFPeA)	PCHI	102	334	290	1494	143	C	Mixed-source
Sucralose	PCHI	106	3308[c]	2375	28122[c]	143	A	Wastewater-derived
Triclocarban	PCHI	42	52	46	86	8	A	Wastewater-derived
Triisopropanolamine	PCHI	7	20	11	313	141	A	Wastewater-derived
Carbanilide	PCHI TP	13	25	24	49	16	D	Mixed-source
Galaxolidone	PCHI TP	13	251	102	4071[c]	138	A	Wastewater-derived

[a] "PHAR" = pharmaceutical; "PEST" = pesticide; "PCHI" = personal care, household and industrial chemical; "TP" = transformation product; [b] Based on hierarchical cluster analysis (Figure 2 in the *Main Text*); [c] Estimated concentration beyond the calibration range (i.e., 0-3000 ng/L).

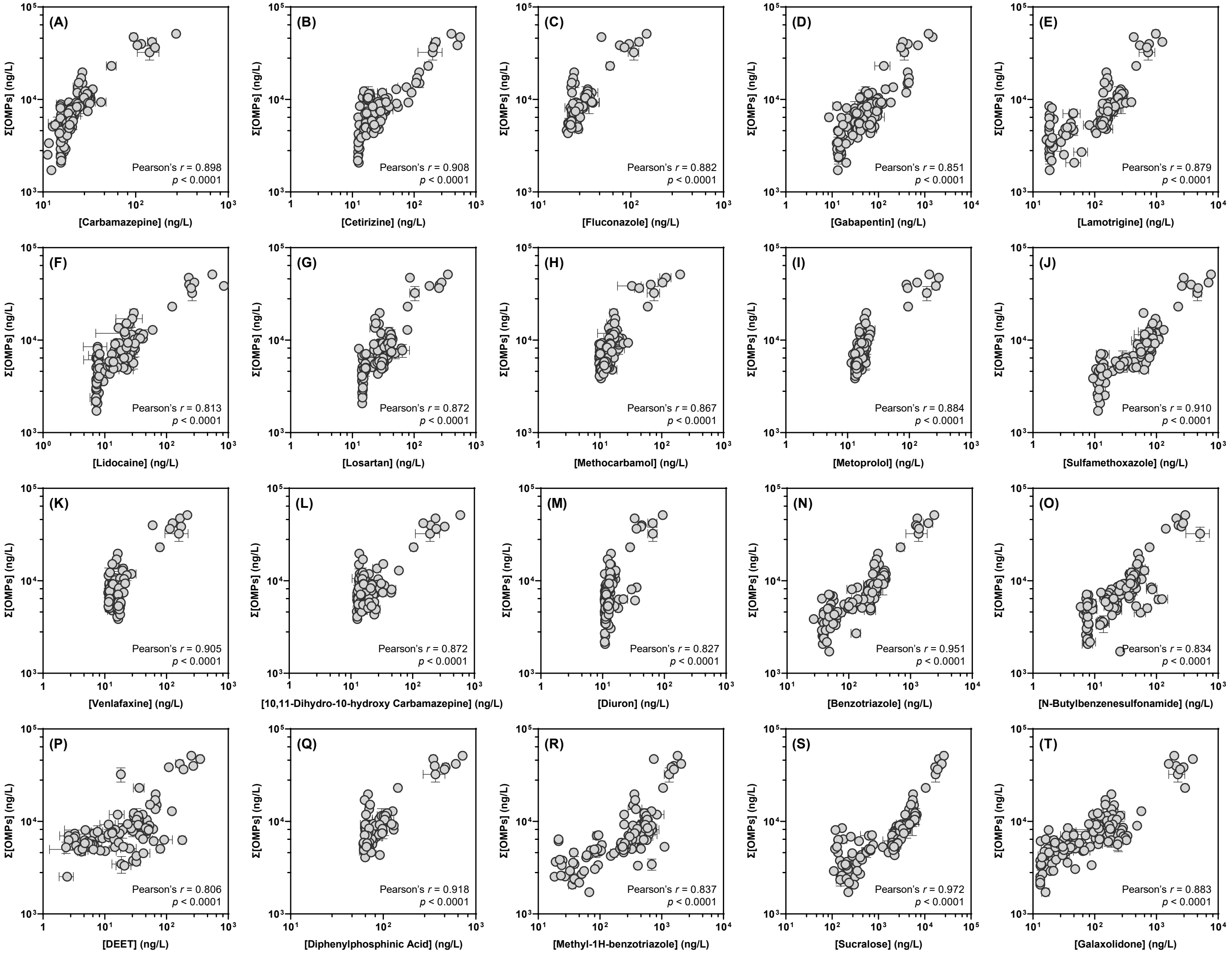

Figure S11. Pearson's correlations between the cumulative concentration of all detected OMPs (∑[OMPs]) and the concentration of 20 wastewater-derived OMPs with a high detection frequency (≥70%) in the samples from the Onondaga Lake-Three Rivers system: (A) Carbamazepine; (B) Cetirizine; (C) Fluconazole; (D) Gabapentin; (E) Lamotrigine; (F) Lidocaine; (G) Losartan; (H) Methocarbamol; (I) Metoprolol; (J) Sulfamethoxazole; (K) Venlafaxine; (L) 10,11-Dihydro-10-hydroxy carbamazepine; (M) Diuron; (N) Benzotriazole; (O) N-Butylbenzenesulfonamide; (P) DEET; (Q) Diphenylphosphinic Acid; (R) Methyl-1H-benzotriazole; (S) Sucralose; and (T) Galaxolidone. Error bars represent the standard deviations from duplicate measurements; where absent, bars fall within symbols.

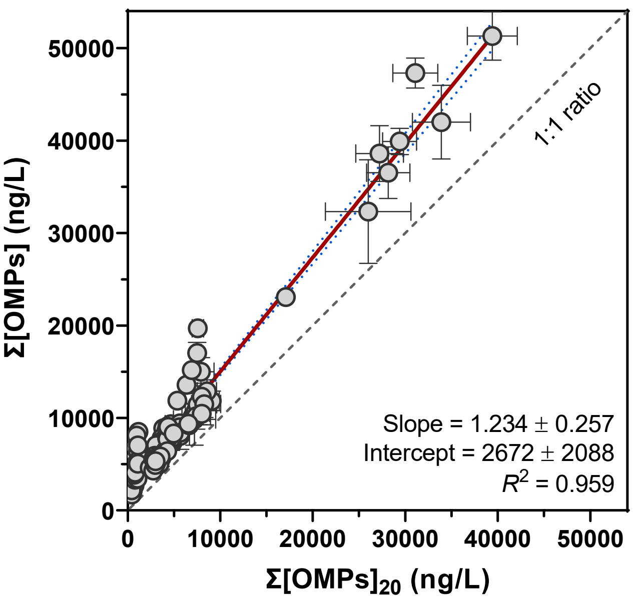

Figure S12. Correlation between the cumulative concentration of all detected OMPs (∑[OMPs]) and the cumulative concentration of 20 wastewater-derived OMPs (∑[OMPs]$_{20}$) in the samples (n = 143) from the Onondaga Lake-Three Rivers system. OMPs contributing to ∑[OMPs]$_{20}$ include carbamazepine, cetirizine, fluconazole, gabapentin, lamotrigine, lidocaine, losartan, methocarbamol, metoprolol, sulfamethoxazole, venlafaxine, 10,11-dihydro-10-hydroxy carbamazepine, diuron, benzotriazole, N-butylbenzenesulfonamide, DEET, diphenylphosphinic acid, methyl-1H-benzotriazole, sucralose, and galaxolidone. The red solid line represents the linear regression line. The blue dotted lines represent the 95% confidence interval of the linear regression line. The grey dashed line represents the 1:1 ratio. Error bars represent the standard deviations from duplicate measurements; where absent, bars fall within symbols.

S8. Predictors of OMP occurrence

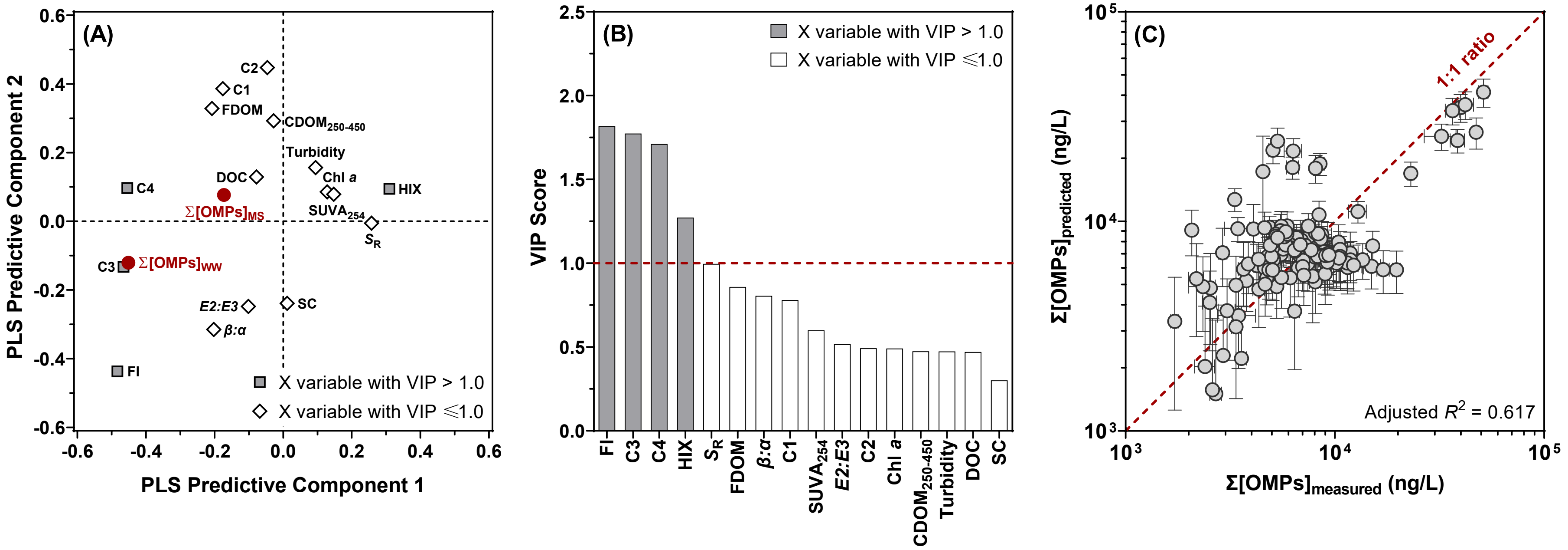

Figure S13. Partial least squares regression (PLSR) and multiple linear regression (MLR) modeling of the occurrence data of OMPs in the samples from the Onondaga Lake-Three Rivers system: (A) Loading scatter plot of PLSR analysis where *Y* variable ($\sum[OMPs]_{WW}$ or $\sum[OMPs]_{MS}$) is the response variable, *X* variable is the predictor variable, and VIP is the variable influence on projection. On the plot, "$\sum[OMPs]_{WW}$" represents the sample-specific cumulative concentration of wastewater-derived OMPs (ng/L), "$\sum[OMPs]_{MS}$" represents the sample-specific cumulative concentration of mixed-source OMPs (ng/L), "SC" represents specific conductance (µS/cm), "Chl *a*" represents the concentration of fluorometric chlorophyll *a* (µg/L), "Turbidity" represents turbidity (NTU), "DOC" represents the concentration of dissolved organic carbon, "$SUVA_{254}$" represents the specific UV absorbance at 254 nm, "*E2:E3*" represents the ratio of absorption coefficients at 250 and 365 nm, "S_R" represents the ratio of spectral slope coefficient $S_{275-295}$ to $S_{290-350}$, "$CDOM_{250-450}$" represents the integrated absorption of chromophoric DOM from 250 to 450 nm (m^{-1}), "FI" represents fluorescence index, "HIX" represents humification index, "*β:α*" represents freshness index, "FDOM" represents the integrated volumetric fluorescence intensity of fluorescent DOM (R.U.; water Raman unit), "C1" represents the maximum intensity of PARAFAC component 1 (R.U.), "C2" represents the maximum intensity of PARAFAC component 2 (R.U.), C3 represents the maximum intensity of PARAFAC component 3 (R.U.), and C4 represents the maximum intensity of PARAFAC component 4 (R.U.). (B) VIP plot of predictor variables where the red dashed line represents the VIP score threshold of 1.0 (*X* variables with a VIP score of >1.0 were most important for the PLSR model performance). (C) Cross plot of measured ∑[OMPs] (with the subscript "measured") versus ∑[OMPs] (with the subscript "predicted") predicted by $\sum[OMPs] = 6716(\pm1033)\times C3 + 7990(\pm1807)\times C4 - 3253(\pm906)$ where ∑[OMPs] is in the unit of ng/L and C3 and C4 are in the unit of water Raman unit (R.U.). Error bars indicate the standard deviation of measured ∑[OMPs] or the 95% confidence interval of predicted ∑[OMPs]. The red dashed line represents the 1:1 ratio. Note that data matrices were log-transformed, centered, and scaled to unit variance prior to PLSR and MLR analysis when applicable.

S9. Exposure-activity ratios for OMPs

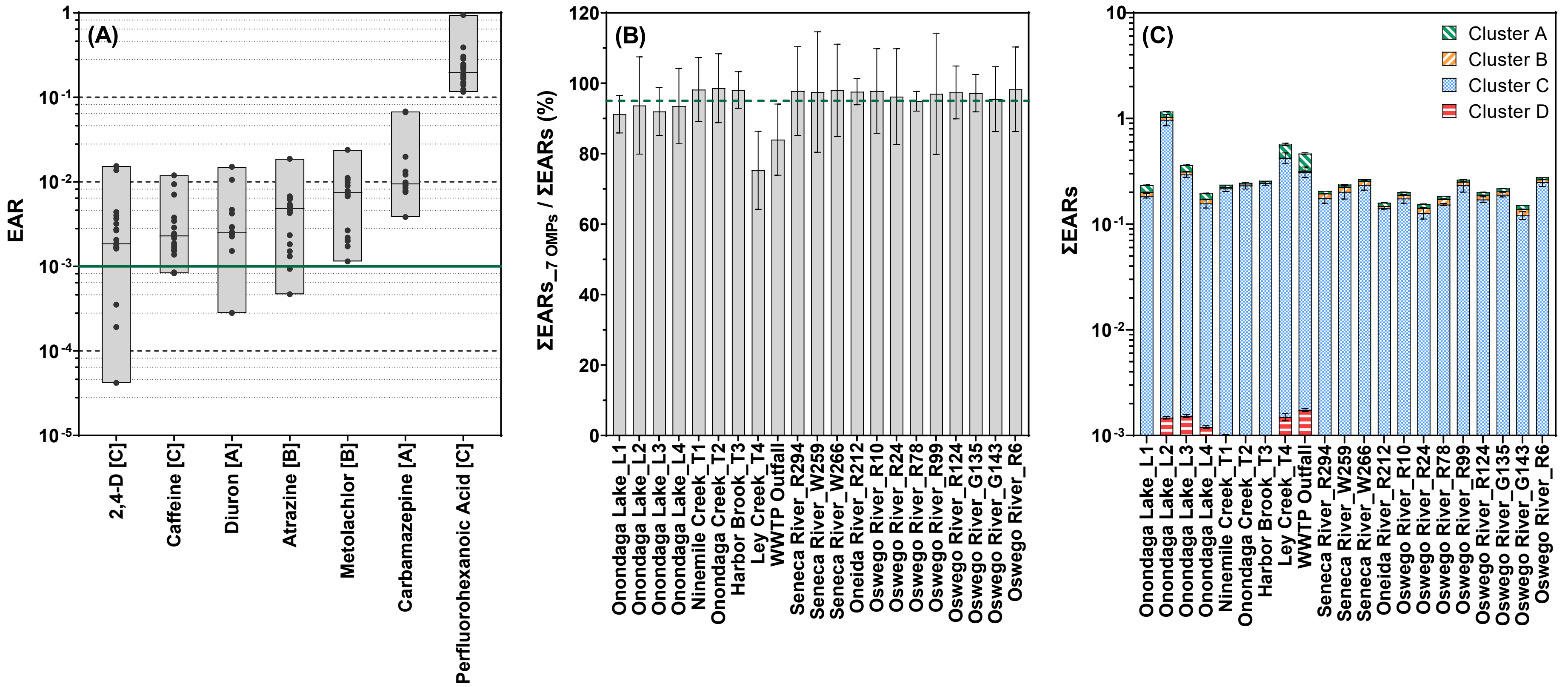

Figure S14. Exposure-activity ratios (EARs) for OMPs in the samples from the Onondaga Lake-Three Rivers system under the mean exposure scenario: (A) Compound-specific EARs for carbamazepine, diuron, caffeine, 2,4-D, atrazine, metolachlor, and perfluorohexanoic acid. Floating bars and centerlines represent the range and median of mean EARs, respectively. Black dots represent individual data points. The green solid line represents the conservative effects-screening threshold of 0.001.[58] Square brackets display the clustering of a given OMP. (B) Percent contributions of the site-specific cumulative EARs for seven OMPs (ΣEARs$_{_7\ OMPs}$) to the site-specific cumulative EARs for 55 OMPs (ΣEARs) included in the ToxCast and Tox21 high-throughput screening database.[58-63] Error bars represent the propagated errors associated with EAR calculations. The green dashed line represents the mean percent contribution of 95±10%. Two sets of assays, *Apredica* and *Bioseek*, as well as *Attagene* assays reporting signal loss and *Novascreen* assays reporting signal gain were excluded from the calculation.[59] Chemical-assay combinations with the following data quality flags were excluded: "only highest concentration above baseline, active", "only one concentration above baseline, active", "noisy data", "borderline active", "gain AC50 < lowest concentration & loss AC50 < mean concentration", and "biochemical assay with < 50% efficacy".[58-60] Endpoints categorized as "background measurement", "cell morphology", "cell cycle", and "undefined" were also excluded from the calculation.[59] (C) ∑EARs for four clusters of OMPs (i.e., Cluster A, B, C, and D). Error bars indicate the standard deviation of ∑EARs for a given cluster of OMPs.

S10. OMPs identified by nontarget screening

Table S12. Examples of OMPs identified by nontarget screening

ID	Molecular Formula	RDBE	H/C	O/C	SFit [%]	Adduct	Exact Mass (*m/z*)	MS/MS Fragment (*m/z*)	RT (min)	*mzCloud* Match Factor	Level	Compound Name
NP273	$C_{18}H_{24}O_2$	7	1.30	0.11	100	[M+H]+	273.1849	227.1776, 240.1500, 255.1726	21.87	86	1	Galaxolidone
NP219	$C_{12}H_{11}O_2P$	8	0.90	0.17	91	[M+H]+	219.0569	159.0212, 201.0473	13.16	95	1	Diphenylphosphinic Acid
NP214	$C_{10}H_{15}NO_2S$	4	1.50	0.15	99	[M+H]+	214.0888	141.0003, 158.0270	15.06	73	1	N-Butylbenzenesulfonamide
NP192a	$C_9H_{21}NO_3$	0	2.33	0.33	92	[M+H]+	192.1594	116.1068, 156.1382, 174.1497	1.68	79	1	Triisopropanolamine
NP146	C_9H_7NO	7	0.78	0.11	100	[M+H]+	146.0598	118.0639, 129.0570	10.96	69	3	-
NP152	$C_8H_9NO_2$	5	1.10	0.25	100	[M+H]+	152.0703	106.0649, 124.0755, 134.0598	3.74	56	3	-
NP165	$C_{10}H_{12}O_2$	5	1.20	0.20	95	[M+H]+	165.0907	91.0541, 119.0854, 147.0803	10.81	63	3	-
NP190	$C_{11}H_{11}NO_2$	7	1.00	0.18	95	[M+H]+	190.0861	121.0647, 147.0675, 162.0909	11.64	69	3	-
NP192b	$C_{12}H_{17}NO$	5	1.42	0.08	94	[M+H]+	192.1377	109.0644, 119.0487	15.12	57	3	-
NP194	$C_{11}H_{15}NO_2$	5	1.40	0.18	100	[M+H]+	194.1170	91.0539, 148.1118, 176.1068	14.00	57	3	-
NP222	$C_{12}H_{15}NO_3$	6	1.30	0.25	91	[M+H]+	222.1119	174.0910, 204.1014	12.25	73	3	-
NP224	$C_{13}H_{21}NO_2$	4	1.60	0.15	92	[M+H]+	224.1642	164.1070, 178.1223, 206.1538	20.27	57	3	-
NP245	$C_{15}H_{16}O_3$	8	1.10	0.20	99	[M+H]+	245.1160	161.0598, 189.0544, 227.1068	16.73	53	3	-
NP141	$C_8H_{12}O_2$	3	1.50	0.25	100	[M+H]+	141.0908	123.0798	8.73	70	4	-
NP167	$C_9H_{10}O_3$	5	1.10	0.33	100	[M+H]+	167.0699	-	11.72	47	4	-
NP181	$C_6H_{12}O_6$	1	2.00	1.00	100	[M+H]+	181.0702	119.4133	5.81	-	4	-
NP186	$C_6H_7N_3O_4$	5	1.20	0.67	100	[M+H]+	186.0511	-	9.52	-	4	-
NP189	$C_7H_{12}N_2O_4$	3	1.70	0.57	100	[M+H]+	189.0872	-	12.02	-	4	-
NP201	$C_7H_8N_2O_5$	5	1.10	0.71	100	[M+H]+	201.0508	-	12.15	-	4	-
NP207	$C_7H_{14}N_2O_5$	2	2.00	0.71	100	[M+H]+	207.0978	-	11.83	-	4	-
NP211	$C_{12}H_{18}O_3$	4	1.50	0.25	100	[M+H]+	211.1318	-	9.42	43	4	-
NP213	$C_9H_{12}N_2O_4$	5	1.30	0.44	100	[M+H]+	213.0872	-	17.21	-	4	-
NP220	$C_9H_{17}NO_5$	2	1.90	0.56	100	[M+H]+	220.1170	-	1.74	-	4	-
NP223	$C_{11}H_{11}O_3P$	7	1.00	0.27	100	[M+H]+	223.0520	-	12.50	-	4	-
NP231a	$C_9H_{14}N_2O_5$	4	1.60	0.56	100	[M+H]+	231.0980	-	16.40	-	4	-
NP231b	$C_{11}H_{19}O_3P$	3	1.70	0.27	100	[M+H]+	231.1146	-	15.06	-	4	-
NP241a	$C_9H_9N_2O_4P$	7	1.00	0.44	100	[M+H]+	241.0376	107.0549	13.17	-	4	-
NP241b	$C_{10}H_{12}N_2O_5$	6	1.20	0.50	100	[M+H]+	241.0818	-	14.28	-	4	-
NP244	$C_9H_{13}N_3O_5$	5	1.40	0.56	100	[M+H]+	244.0928	114.9319	12.72	39	4	-
NP252	$C_8H_{17}N_3O_6$	2	2.10	0.75	100	[M+H]+	252.1201	-	2.81	-	4	-
NP254	$C_{11}H_{15}N_3O_4$	6	1.40	0.36	100	[M+H]+	254.1130	-	9.71	-	4	-
NP258	$C_{12}H_{20}NO_3P$	4	1.70	0.25	100	[M+H]+	258.1241	-	16.37	-	4	-
NP266a	$C_9H_{19}N_3O_6$	2	2.10	0.70	100	[M+H]+	266.1349	-	16.26	-	4	-
NP266b	$C_{14}H_{19}NO_4$	6	1.40	0.29	100	[M+H]+	266.1380	-	6.81	31	4	-
NP269	$C_{12}H_{16}N_2O_5$	6	1.30	0.42	100	[M+H]+	269.1122	-	11.93	-	4	-
NP274	$C_9H_{11}N_3O_7$	6	1.20	0.78	100	[M+H]+	274.0669	-	11.93	-	4	-
NP277	$C_{10}H_{17}N_2O_5P$	4	1.70	0.50	100	[M+H]+	277.0937	235.1686	13.90	33	4	-

**Table S12.** Examples of OMPs identified by nontarget screening (continued)												
ID	Molecular Formula	RDBE	H/C	O/C	SFit [%]	Adduct	Exact Mass (*m/z*)	MS/MS Fragment (*m/z*)	RT (min)	*mzCloud* Match Factor	Level	Compound Name
NP279	$C_{12}H_{26}N_2O_5$	1	2.20	0.42	100	[M+H]+	279.1914	-	16.12	-	4	-
NP287a	$C_9H_{18}O_8S$	1	2.00	0.89	100	[M+H]+	287.0784	-	9.01	-	4	-
NP287b	$C_{12}H_{18}N_2O_6$	5	1.50	0.50	100	[M+H]+	287.1236	-	11.52	-	4	-
NP291	$C_{13}H_{22}O_7$	3	1.70	0.54	100	[M+H]+	291.1429	-	8.52	-	4	-
NP295	$C_{14}H_{18}N_2O_5$	7	1.30	0.36	100	[M+H]+	295.1288	-	16.59	-	4	-
NP313	$C_{14}H_{20}N_2O_6$	6	1.40	0.43	100	[M+H]+	313.1392	-	17.58	-	4	-
NP321	$C_{13}H_{21}O_7P$	4	1.60	0.54	100	[M+H]+	321.1082	-	14.15	-	4	-
NP334	$C_{14}H_{27}N_3O_6$	3	1.90	0.43	100	[M+H]+	334.1962	-	15.69	-	4	-
NP336	$C_{18}H_{25}NO_5$	7	1.40	0.28	100	[M+H]+	336.1798	-	15.18	-	4	-
NP340	$C_{17}H_{25}NO_6$	6	1.50	0.35	100	[M+H]+	340.1752	-	11.24	-	4	-
NP343	$C_{12}H_{26}N_2O_9$	1	2.20	0.75	100	[M+H]+	343.1707	-	10.51	-	4	-
NP348a	$C_{15}H_{29}N_3O_6$	3	1.90	0.40	100	[M+H]+	348.2124	-	15.69	-	4	-
NP348b	$C_{10}H_9N_2O_8PS$	8	0.90	0.80	100	[M+H]+	348.9879	-	17.95	-	4	-
NP349	$C_{19}H_{34}O_4$	3	1.80	0.21	100	[M+Na]+	349.2360	149.0233	23.43	61	4	-
NP352	$C_{18}H_{25}NO_6$	7	1.40	0.33	100	[M+H]+	352.1741	-	12.92	-	4	-
NP355	$C_{18}H_{30}N_2O_5$	5	1.70	0.28	100	[M+H]+	355.2220	-	21.81	-	4	-
NP360	$C_{15}H_{25}N_3O_7$	5	1.70	0.47	100	[M+H]+	360.1781	-	16.05	-	4	-
NP361	$C_{16}H_{28}N_2O_7$	4	1.80	0.44	100	[M+H]+	361.1962	-	22.23	49	4	-
NP362	$C_{16}H_{31}N_3O_6$	3	1.90	0.38	100	[M+H]+	362.2276	-	15.69	-	4	-
NP380	$C_{17}H_{21}N_3O_7$	9	1.20	0.41	100	[M+H]+	380.1436	-	15.18	-	4	-
NP389	$C_{19}H_{33}O_6P$	4	1.70	0.32	100	[M+H]+	389.2099	-	14.42	-	4	-
NP401	$C_{18}H_{28}N_2O_8$	6	1.60	0.44	100	[M+H]+	401.1908	-	19.02	41	4	-
NP429	$C_{17}H_{37}N_2O_8P$	1	2.20	0.47	100	[M+H]+	429.2378	-	16.42	-	4	-
NP430	$C_{13}H_{24}N_3O_{11}P$	4	1.80	0.85	100	[M+H]+	430.1227	-	17.82	35	4	-
NN165	$C_5H_{10}O_6$	1	2.00	1.20	100	[M-H]-	165.0417	-	2.88	-	4	-

"RDBE" = the rings and double bonds equivalent value for the predicted formula; "H/C" = the ratio of hydrogen to carbon atoms in the predicted formula; "O/C" = the ratio of oxygen to carbon atoms in the predicted formula; "SFit(%)" = the spectral similarity score between the theoretical and the measured isotope patterns as a percentage. Identification confidence levels adapted from Schymanski *et al.*:[64] Level 1 – the structure was confirmed via matching MS, MS/MS, and retention time with those of the authentic reference standard. Level 3 – the structure was tentatively identified via matching MS and MS/MS fragments with those of the top-ranked structure in *mzCloud*. Level 4 – the molecular formula was proposed with 100% SFit(%) but minimal or no MS/MS fragmentation information for structural elucidation. "NP" denotes nontarget compounds detected in positive electrospray ionization mode. "NN" denotes nontarget compounds detected in negative electrospray ionization mode.

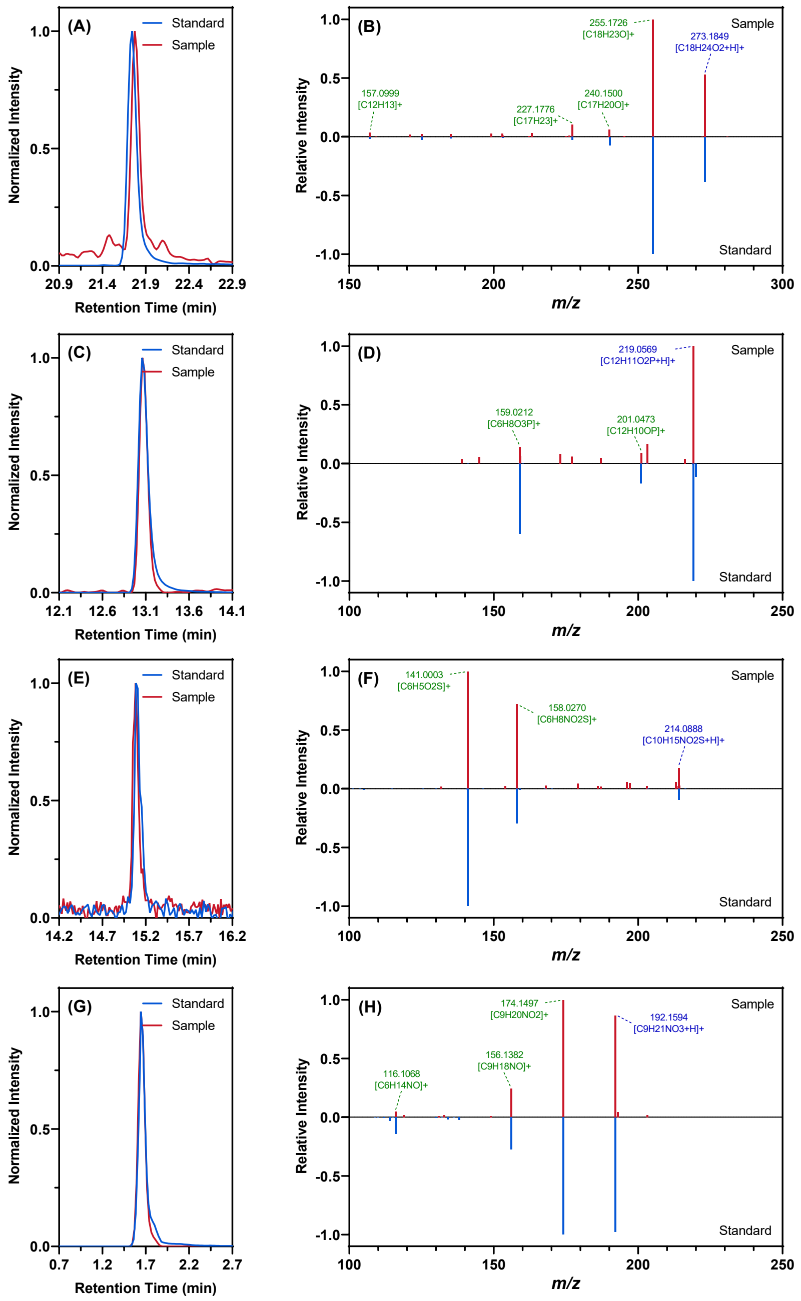

Figure S15. Level 1 confirmation of NP273 (galaxolidone), NP219 (diphenylphosphinic acid), NP214 (*N*-butylbenzenesulfonamide), and NP192a (triisopropanolamine): (A) Normalized extracted ion chromatograms of NP273 in Onondaga Lake water (retention time = 21.87 min) and galaxolidone reference standard (retention time = 21.86 min). (B) Head-to-tail plot of dd-MS2 spectra of NP273 in Onondaga Lake water (HCD 45%) and galaxolidone reference standard (HCD 45%). (C) Normalized extracted ion chromatograms of NP219 in Onondaga Lake water (retention time = 13.16 min) and diphenylphosphinic acid reference standard (retention time = 13.07 min). (D) Head-to-tail plot of dd-MS2 spectra of NP219 in Onondaga Lake water (HCD 30%) and diphenylphosphinic acid reference standard (HCD 30%). (E) Normalized extracted ion chromatograms of NP214 in Onondaga Lake water (retention time = 15.06 min) and *N*-butylbenzenesulfonamide reference standard (retention time = 15.08 min). (F) Head-to-tail plot of dd-MS2 spectra of NP214 in Onondaga Lake water (HCD 30%) and *N*-butylbenzenesulfonamide reference standard (HCD 30%). (G) Normalized extracted ion chromatograms of NP192a in Onondaga Lake water (retention time = 1.68 min) and triisopropanolamine reference standard (retention time = 1.62 min). (H) Head-to-tail plot of dd-MS2 spectra of NP192a in Onondaga Lake water (HCD 45%) and triisopropanolamine reference standard (HCD 45%). Further compound information is summarized in Table S12.

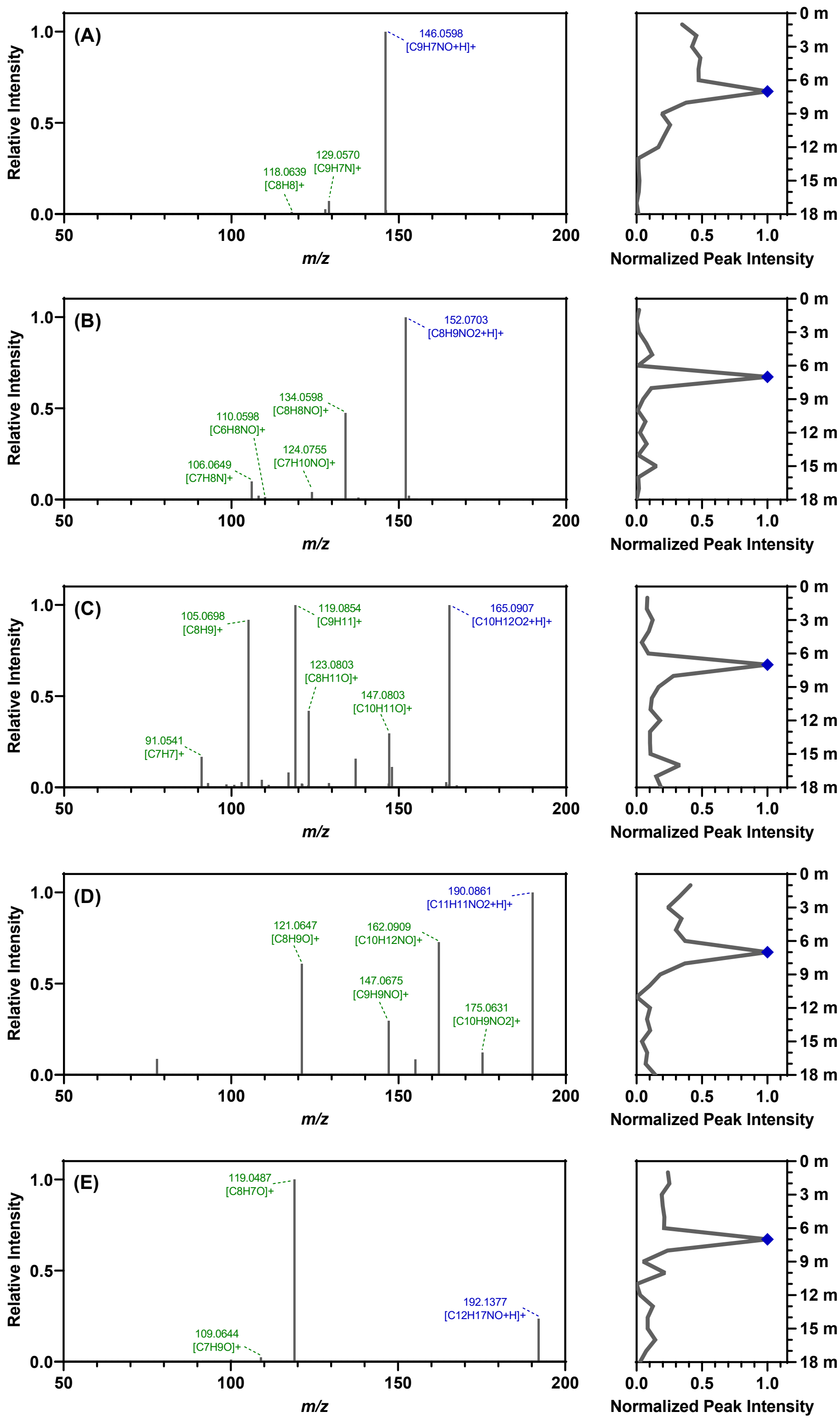

Figure S16a. Level 3 identification of nontarget compounds: (A) dd-MS2 spectrum (HCD 45%) and normalized peak intensity profile of NP146 in Onondaga Lake water. (B) dd-MS2 spectrum (HCD 60%) and normalized peak intensity profile of NP152 in Onondaga Lake water. (C) dd-MS2 spectrum (HCD 45%) and normalized peak intensity profile of NP165 in Onondaga Lake water. (D) dd-MS2 spectrum (HCD 45%) and normalized peak intensity profile of NP190 in Onondaga Lake water. (E) dd-MS2 spectrum (HCD 60%) and normalized peak intensity profile of NP192b in Onondaga Lake water. Further compound information is summarized in Table S12.

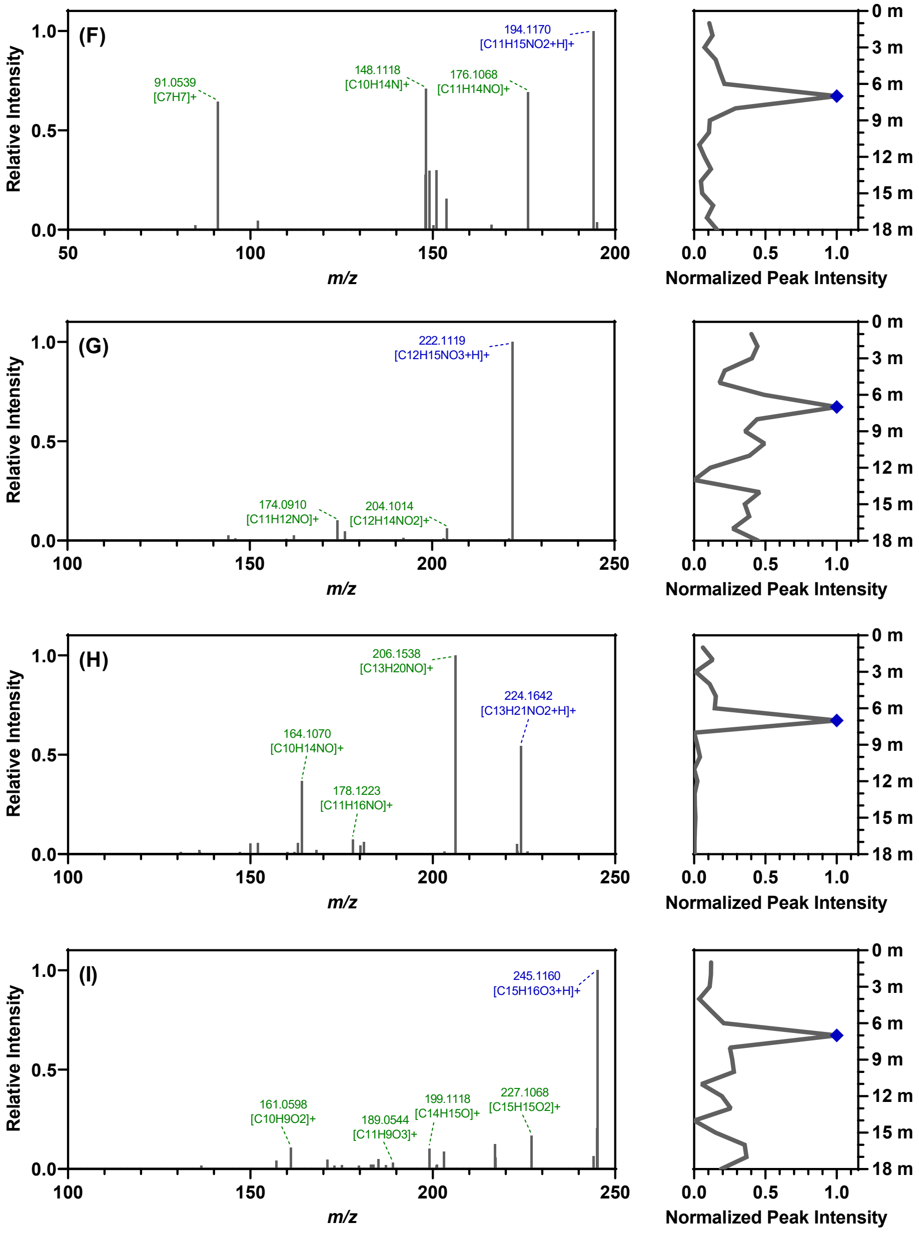

Figure S16b. Level 3 identification of nontarget compounds: (F) dd-MS2 spectrum (HCD 45%) and normalized peak intensity profile of NP194 in Onondaga Lake water. (G) dd-MS2 spectrum (HCD 45%) and normalized peak intensity profile of NP222 in Onondaga Lake water. (H) dd-MS2 spectrum (HCD 30%) and normalized peak intensity profile of NP224 in Onondaga Lake water. (I) dd-MS2 spectrum (HCD 45%) and normalized peak intensity profile of NP245 in Onondaga Lake water. Further compound information is summarized in Table S12.

S11. Measured and simulated vertical concentration profiles of OMPs in Onondaga Lake

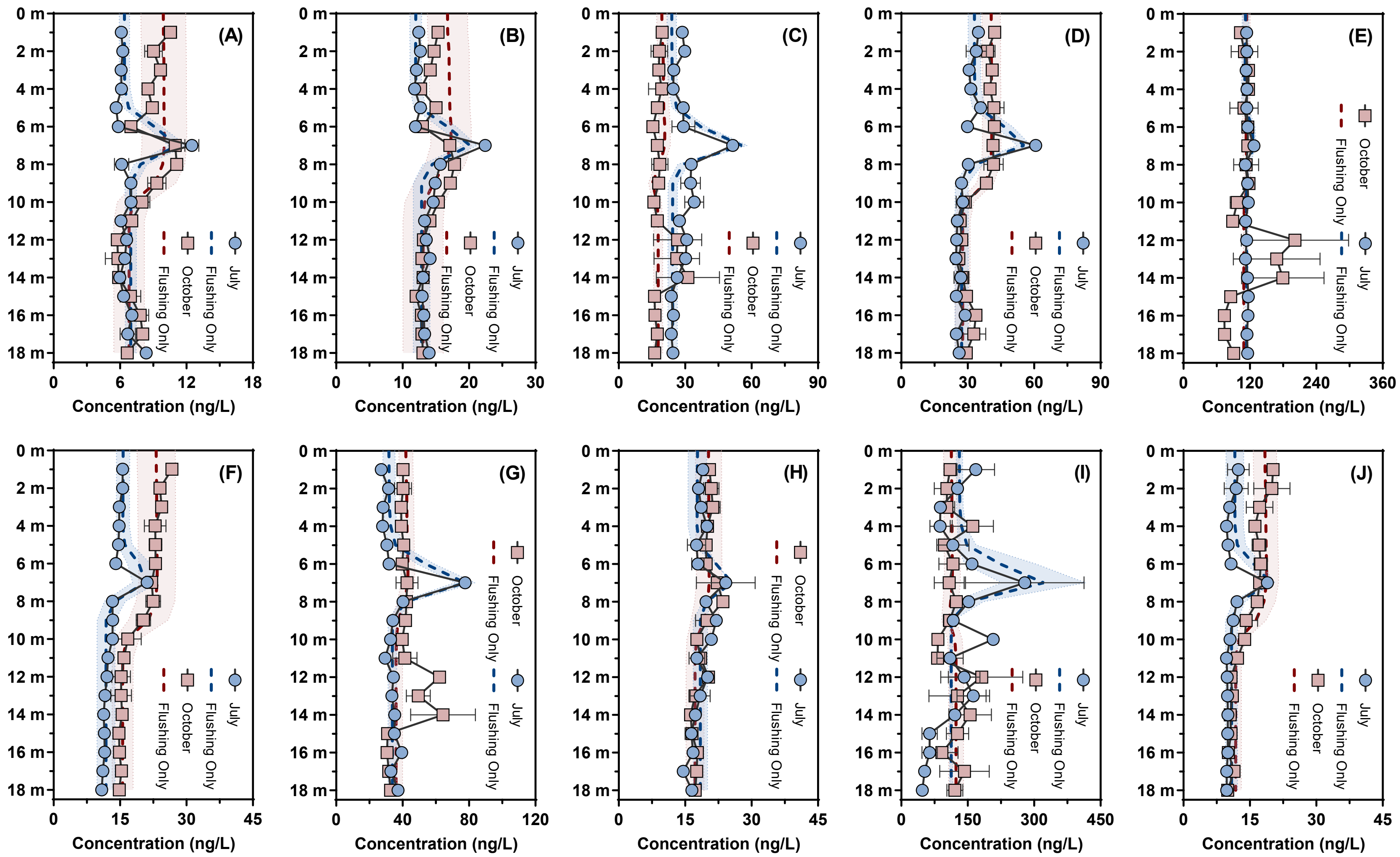

Figure S17a. Measured and *AQUASIM*-simulated vertical concentration profiles of wastewater-derived OMPs in Onondaga Lake assuming flushing as the sole elimination process: (A) Amantadine. (B) Atenolol. (C) Cetirizine. (D) Dopamine. (E) Fexofenadine. (F) Levetiracetam. (G) Losartan. (H) Metaxalone. (I) Metformin. (J) Methocarbamol. Solid lines with symbols represent the measured vertical concentration profiles in July and October 2017. Error bars represent the standard deviation of duplicate measurements; where absent, bars fall within symbols. Dashed lines and error bands represent the simulated vertical concentration profiles and the standard deviation of model simulations, respectively. PBIAS and NSE values for *AQUASIM*-simulated profiles are summarized in Figure S19.

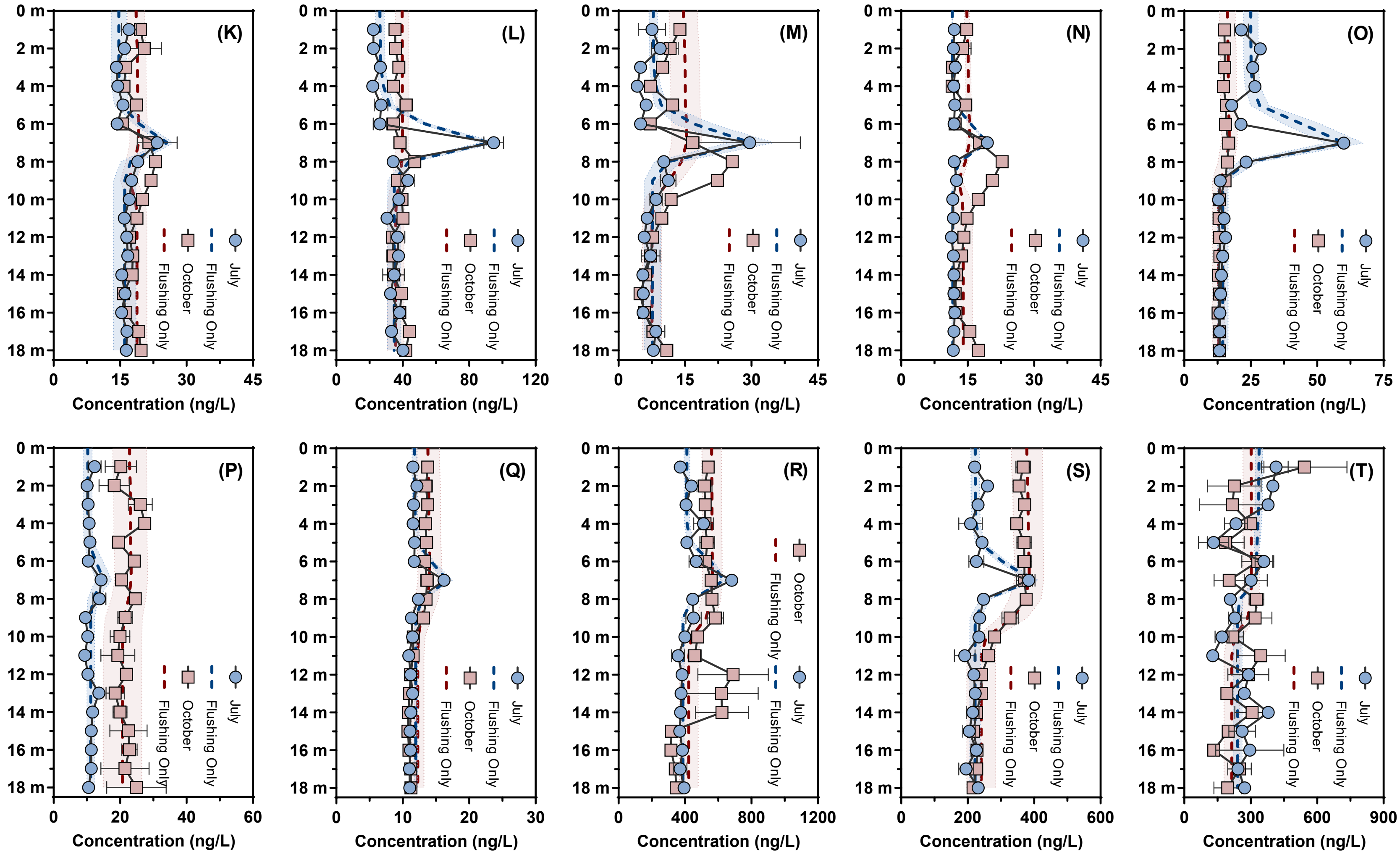

Figure S17b. Measured and *AQUASIM*-simulated vertical concentration profiles of wastewater-derived OMPs in Onondaga Lake assuming flushing as the sole elimination process: (K) Metoprolol. (L) Naproxen. (M) Trimethoprim. (N) Venlafaxine. (O) 10,11-Dihydro-10-hydroxy Carbamazepine. (P) 2-Naphthoxyacetic Acid. (Q) Diuron. (R) Methyl-1H-benzotriazole. (S) Benzotriazole. (T) Benzothiazole. Solid lines with symbols represent the measured vertical concentration profiles in July and October 2017. Error bars represent the standard deviation of duplicate measurements; where absent, bars fall within symbols. Dashed lines and error bands represent the simulated vertical concentration profiles and the standard deviation of model simulations, respectively. PBIAS and NSE values for *AQUASIM*-simulated profiles are summarized in Figure S19.

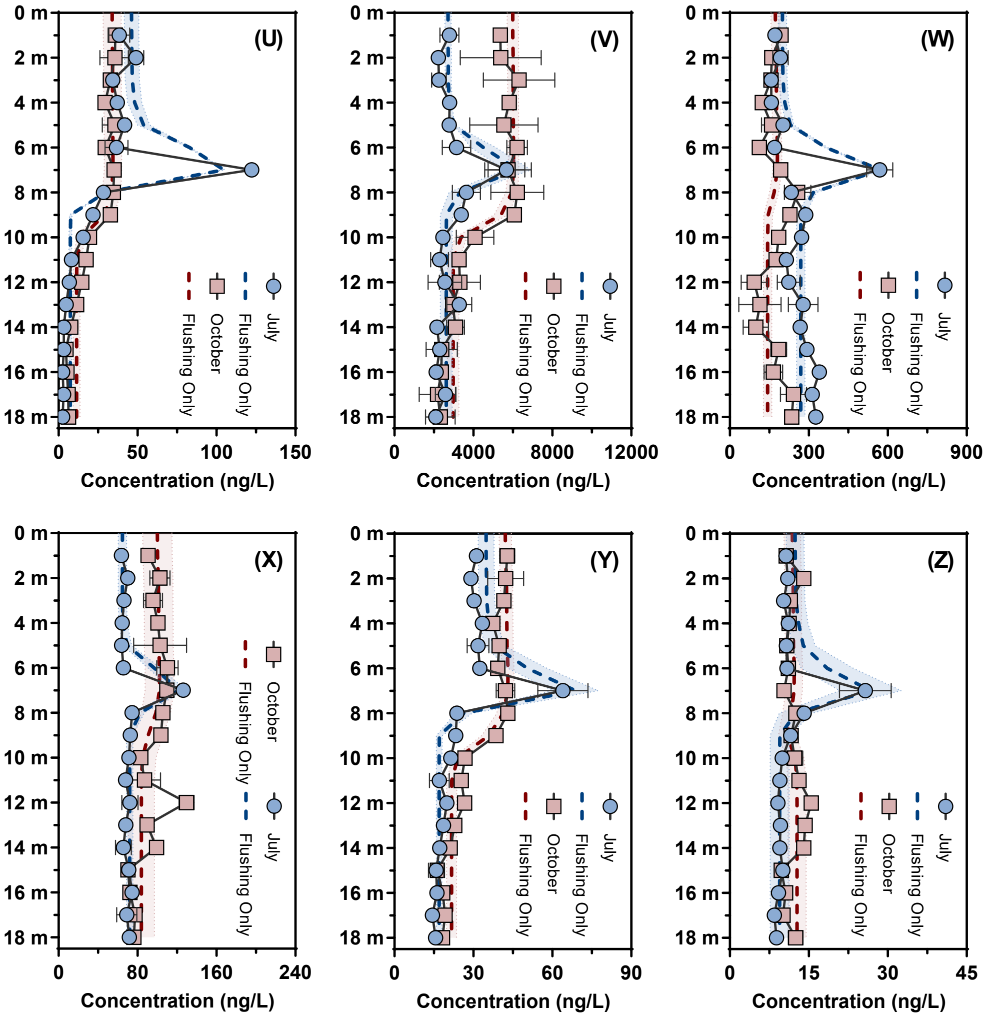

Figure S17c. Measured and *AQUASIM*-simulated vertical concentration profiles of wastewater-derived OMPs in Onondaga Lake assuming flushing as the sole elimination process: (U) DEET. (V) Sucralose. (W) Galaxolidone. (X) Diphenylphosphinic Acid. (Y) N-Butylbenzenesulfonamide. (Z) Triisopropanolamine. Solid lines with symbols represent the measured vertical concentration profiles in July and October 2017. Error bars represent the standard deviation of duplicate measurements; where absent, bars fall within symbols. Dashed lines and error bands represent the simulated vertical concentration profiles and the standard deviation of model simulations, respectively. PBIAS and NSE values for *AQUASIM*-simulated profiles are summarized in Figure S19.

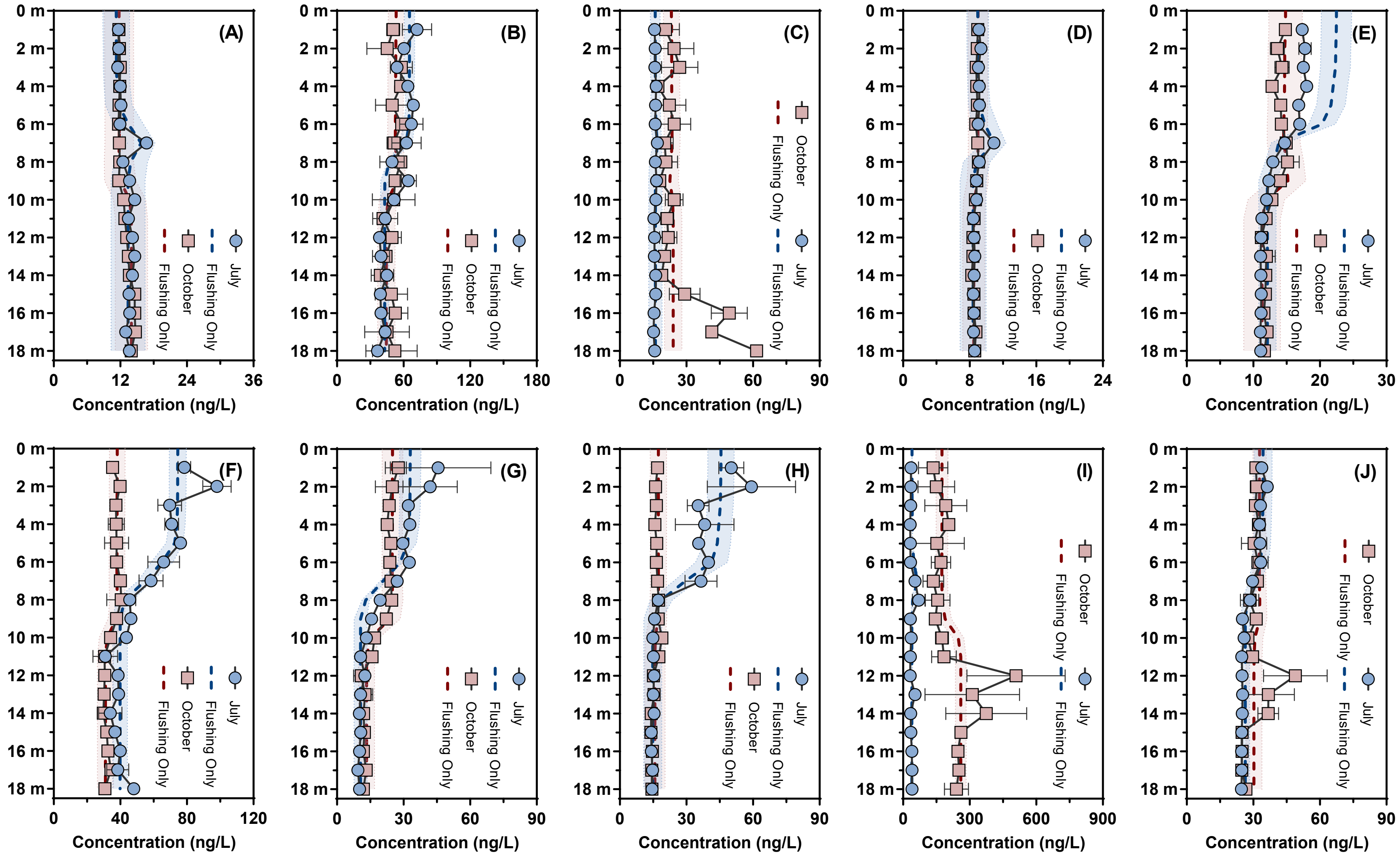

Figure S18a. Measured and *AQUASIM*-simulated vertical concentration profiles of mixed-source OMPs in Onondaga Lake assuming flushing as the sole elimination process: (A) Benzoylecgonine. (B) 2,4-D. (C) Imazapyr. (D) Prometon. (E) Propazine. (F) Atrazine-2-hydroxy. (G) Atrazine-desethyl. (H) Atrazine-desisopropyl. (I) Metolachlor ESA. (J) Metolachlor OA. Solid lines with symbols represent the measured vertical concentration profiles in July and October 2017. Error bars represent the standard deviation of duplicate measurements; where absent, bars fall within symbols. Dashed lines and error bands represent the simulated vertical concentration profiles and the standard deviation of model simulations, respectively. PBIAS and NSE values for *AQUASIM*-simulated profiles are summarized in Figure S19.

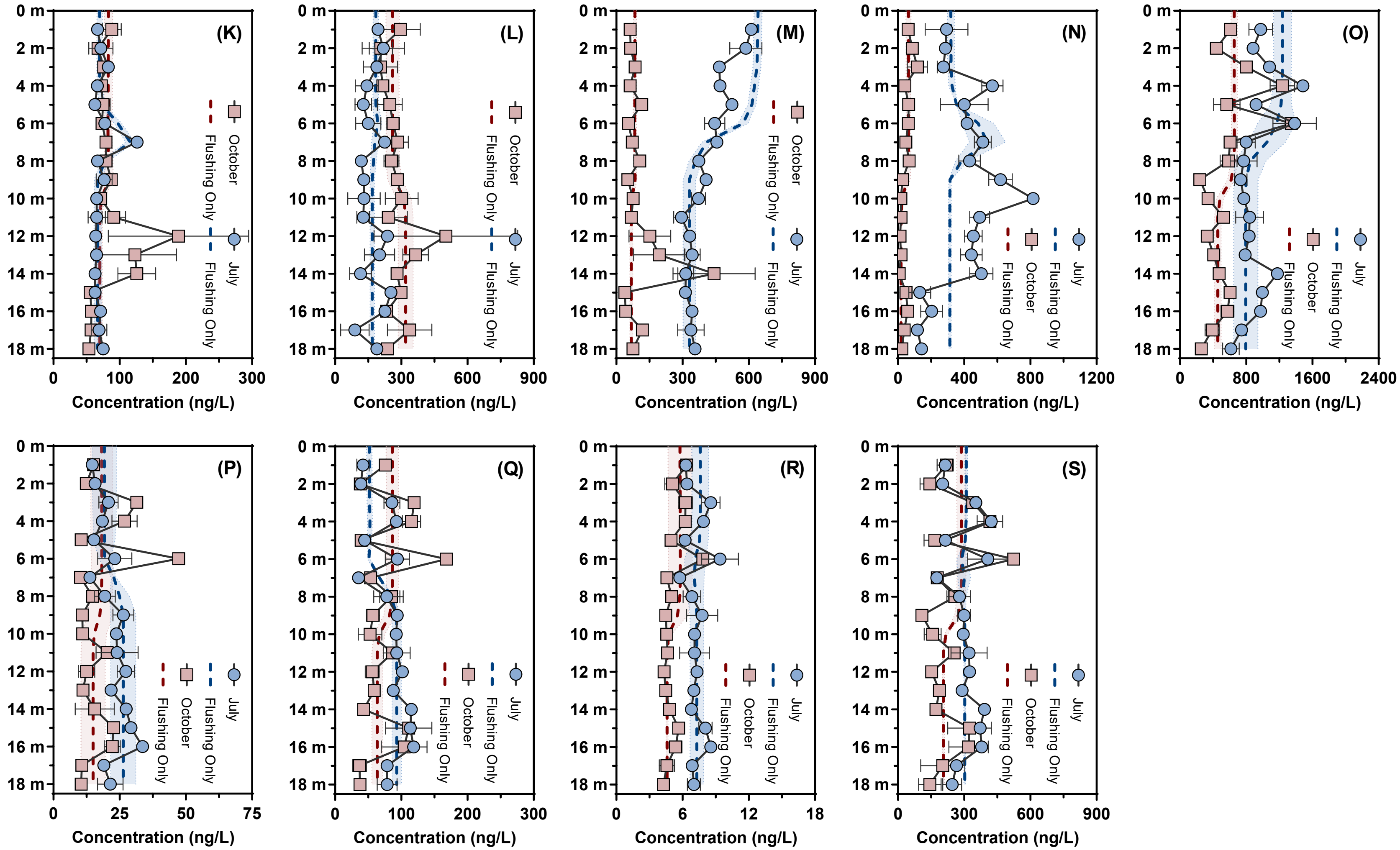

Figure S18b. Measured and *AQUASIM*-simulated vertical concentration profiles of mixed-source OMPs in Onondaga Lake assuming flushing as the sole elimination process: (K) 2-Hydroxybenzothiazole. (L) Benzophenone. (M) Oxybenzone. (N) Melamine. (O) Perfluorobutanoic Acid. (P) Perfluoroheptanoic Acid. (Q) Perfluorohexanoic Acid. (R) Perfluorononanoic Acid. (S) Perfluoropentanoic Acid. Solid lines with symbols represent the measured vertical concentration profiles in July and October 2017. Error bars represent the standard deviation of duplicate measurements; where absent, bars fall within symbols. Dashed lines and error bands represent the simulated vertical concentration profiles and the standard deviation of model simulations, respectively. PBIAS and NSE values for *AQUASIM*-simulated profiles are summarized in Figure S19.

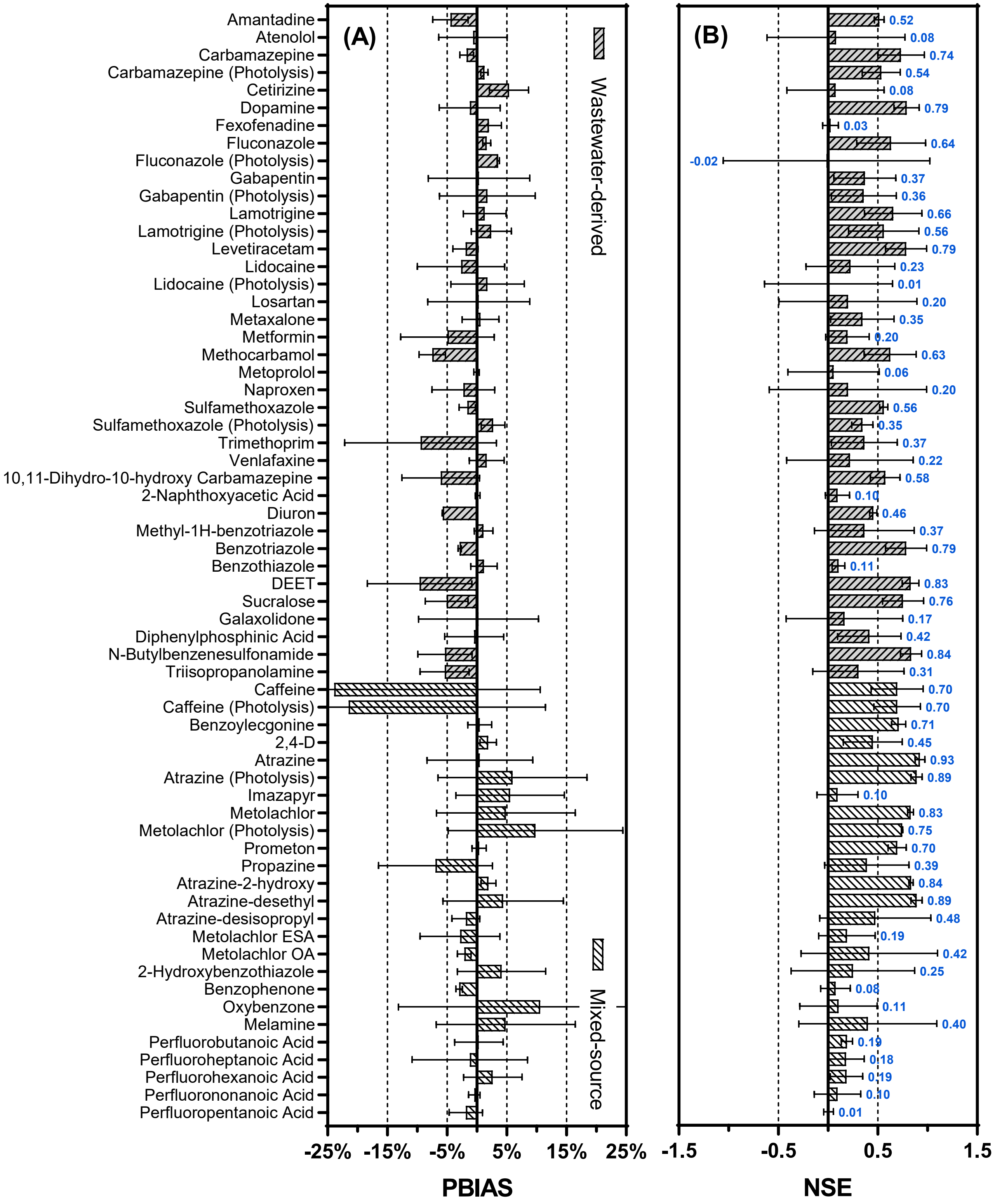

Figure S19. Model performance metrics for the *AQUASIM* simulation of vertical concentration profiles of 54 wastewater-derived and mixed-source OMPs: (A) Percent bias (PBIAS). (B) Nash-Sutcliffe efficiency (NSE). Error bars represent the standard deviation of metrics calculated for vertical concentration profiles in July and October 2017.

S12. Load apportionment of OMPs in the Onondaga Lake-Three Rivers system

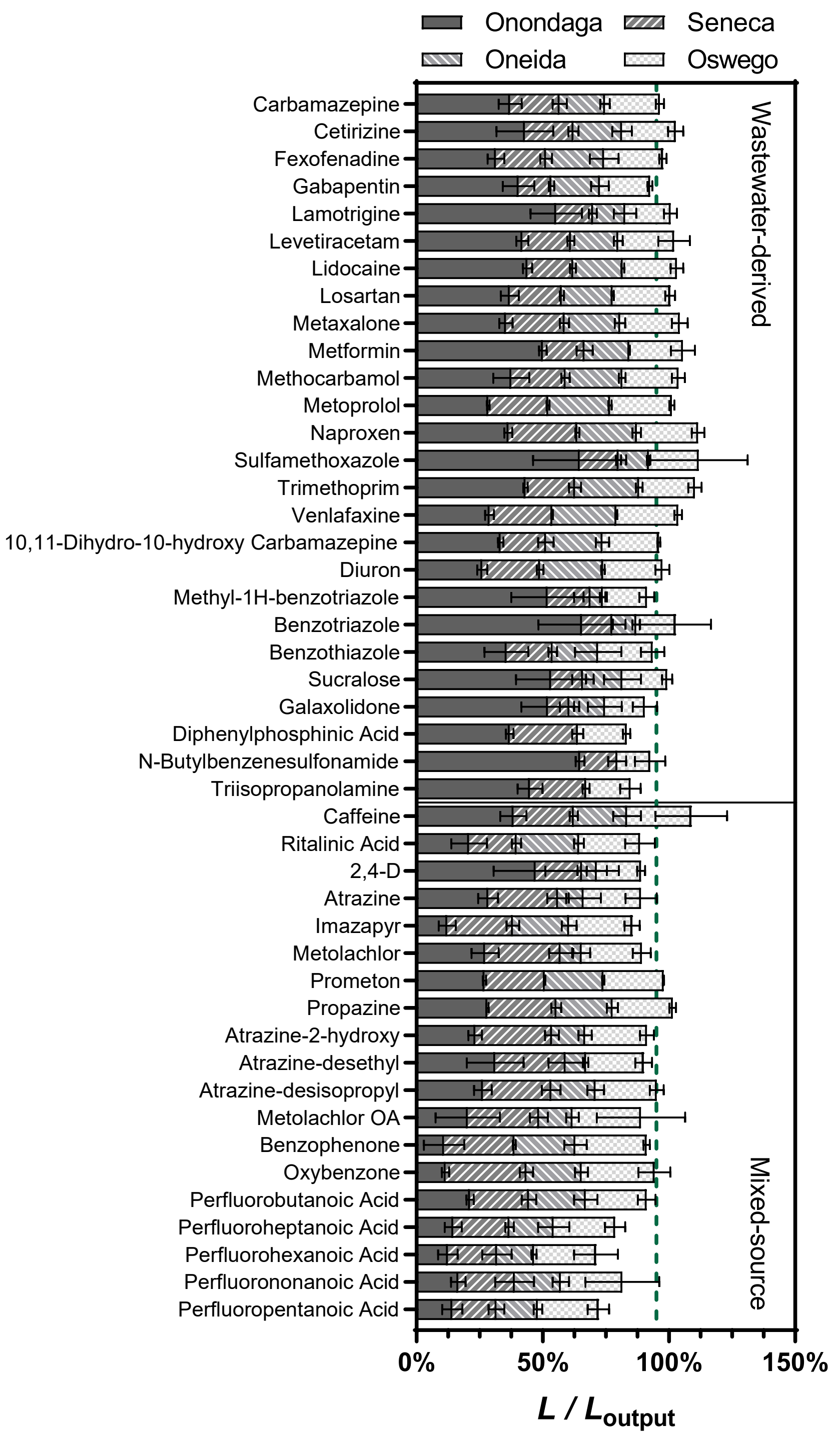

Figure S20. Fractional contributions of Onondaga Lake and the Three Rivers to the loads of 45 wastewater-derived and mixed-source OMPs entering Lake Ontario. The green dashed line represents the mean fractional load contribution of 95±9%. Error bars represent the standard deviation of fractional contributions for loads in July and October 2017.

References

1. Effler, S. W.; Prestigiacomo, A. R.; Effler, A. J. P.; Driscoll, C., Water quality patterns in a river-lake system from multiple drivers (Three Rivers, New York State). *River Systems* **2010,** *19*, (1), 75-94.
2. Prestigiacomo, A. R.; Effler, S. W.; Matthews, D. A., Resolution and analysis of spatial variations and patterns in an urban lake with rapid profiling instrumentation. *JAWRA Journal of the American Water Resources Association* **2015,** *51*, (1), 200-213.
3. Wang, S.; Matt, M.; Murphy, B. L.; Perkins, M.; Matthews, D. A.; Moran, S. D.; Zeng, T., Organic micropollutants in New York lakes: A statewide citizen science occurrence study. *Environmental Science & Technology* **2020,** *54*, (21), 13759-13770.
4. Hu, C.; Muller-Karger, F. E.; Zepp, R. G., Absorbance, absorption coefficient, and apparent quantum yield: A comment on common ambiguity in the use of these optical concepts. *Limnology and Oceanography* **2002,** *47*, (4), 1261-1267.
5. Summers, R. S.; Cornel, P. K.; Roberts, P. V., Molecular size distribution and spectroscopic characterization of humic substances. *Science of the Total Environment* **1987,** *62*, 27-37.
6. Cuthbert, I. D.; del Giorgio, P., Toward a standard method of measuring color in freshwater. *Limnology and Oceanography* **1992,** *37*, (6), 1319-1326.
7. Chin, Y.-P.; Aiken, G.; O'Loughlin, E., Molecular weight, polydispersity, and spectroscopic properties of aquatic humic substances. *Environmental Science & Technology* **1994,** *28*, (11), 1853-1858.
8. De Haan, H.; De Boer, T., Applicability of light absorbance and fluorescence as measures of concentration and molecular size of dissolved organic carbon in humic Lake Tjeukemeer. *Water Research* **1987,** *21*, (6), 731-734.
9. Helms, J. R.; Stubbins, A.; Ritchie, J. D.; Minor, E. C.; Kieber, D. J.; Mopper, K., Absorption spectral slopes and slope ratios as indicators of molecular weight, source, and photobleaching of chromophoric dissolved organic matter. *Limnology and Oceanography* **2008,** *53*, (3), 955-969.
10. Porcal, P.; Dillon, P. J.; Molot, L. A., Interaction of extrinsic chemical factors affecting photodegradation of dissolved organic matter in aquatic ecosystems. *Photochemical & Photobiological Sciences* **2014,** *13*, (5), 799-812.
11. Weishaar, J. L.; Aiken, G. R.; Bergamaschi, B. A.; Fram, M. S.; Fujii, R.; Mopper, K., Evaluation of specific ultraviolet absorbance as an indicator of the chemical composition and reactivity of dissolved organic carbon. *Environmental Science & Technology* **2003,** *37*, (20), 4702-4708.
12. McKnight, D. M.; Boyer, E. W.; Westerhoff, P. K.; Doran, P. T.; Kulbe, T.; Andersen, D. T., Spectrofluorometric characterization of dissolved organic matter for indication of precursor organic material and aromaticity. *Limnology and Oceanography* **2001,** *46*, (1), 38-48.
13. Cory, R. M.; McKnight, D. M., Fluorescence spectroscopy reveals ubiquitous presence of oxidized and reduced quinones in dissolved organic matter. *Environmental Science & Technology* **2005,** *39*, (21), 8142-8149.
14. Zsolnay, A.; Baigar, E.; Jimenez, M.; Steinweg, B.; Saccomandi, F., Differentiating with fluorescence spectroscopy the sources of dissolved organic matter in soils subjected to drying. *Chemosphere* **1999,** *38*, (1), 45-50.
15. Ohno, T., Fluorescence inner-filtering correction for determining the humification index of dissolved organic matter. *Environmental Science & Technology* **2002,** *36*, (4), 742-746.
16. Halbedel, S.; Herzsprung, P., Short communication on “Differentiating with fluorescence spectroscopy the sources of dissolved organic matter in soils subjected to drying” [Zsolnay, A.; Baigar, E.; Jimenez, M.; Steinweg, B.; Saccomandi, F.; Chemosphere 38, 45–50, 1999]. *Chemosphere* **2020,** *239*, Article Number: 124818.
17. Parlanti, E.; Wörz, K.; Geoffroy, L.; Lamotte, M., Dissolved organic matter fluorescence spectroscopy as a tool to estimate biological activity in a coastal zone submitted to anthropogenic inputs. *Organic Geochemistry* **2000,** *31*, (12), 1765-1781.
18. Wilson, H. F.; Xenopoulos, M. A., Effects of agricultural land use on the composition of fluvial dissolved organic matter. *Nature Geoscience* **2008,** *2*, 37-41.

19. Huguet, A.; Vacher, L.; Relexans, S.; Saubusse, S.; Froidefond, J. M.; Parlanti, E., Properties of fluorescent dissolved organic matter in the Gironde Estuary. *Organic Geochemistry* **2009,** *40*, (6), 706-719.
20. Chen, W.; Westerhoff, P.; Leenheer, J. A.; Booksh, K., Fluorescence excitation-emission matrix regional integration to quantify spectra for dissolved organic matter. *Environmental Science & Technology* **2003,** *37*, (24), 5701-5710.
21. Zhou, J.; Wang, J. J.; Baudon, A.; Chow, A. T., Improved fluorescence excitation-emission matrix regional integration to quantify spectra for fluorescent dissolved organic matter. *Journal of Environmental Quality* **2013,** *42*, (3), 925-930.
22. Murphy, K. R.; Stedmon, C. A.; Graeber, D.; Bro, R., Fluorescence spectroscopy and multi-way techniques. PARAFAC. *Analytical Methods* **2013,** *5*, (23), 6557-6566.
23. Murphy, K. R.; Timko, S. A.; Gonsior, M.; Powers, L. C.; Wünsch, U. J.; Stedmon, C. A., Photochemistry illuminates ubiquitous organic matter fluorescence spectra. *Environmental Science & Technology* **2018,** *52*, (19), 11243-11250.
24. Murphy, K. R.; Stedmon, C. A.; Wenig, P.; Bro, R., OpenFluor- an online spectral library of auto-fluorescence by organic compounds in the environment. *Analytical Methods* **2014,** *6*, (3), 658-661.
25. Wünsch, U. J.; Murphy, K. R.; Stedmon, C. A., The one-sample PARAFAC approach reveals molecular size distributions of fluorescent components in dissolved organic matter. *Environmental Science & Technology* **2017,** *51*, (20), 11900-11908.
26. Fellman, J. B.; Hood, E.; Spencer, R. G. M., Fluorescence spectroscopy opens new windows into dissolved organic matter dynamics in freshwater ecosystems: A review. *Limnology and Oceanography* **2010,** *55*, (6), 2452-2462.
27. Lambert, T.; Bouillon, S.; Darchambeau, F.; Massicotte, P.; Borges, A. V., Shift in the chemical composition of dissolved organic matter in the Congo River network. *Biogeosciences* **2016,** *13*, (18), 5405-5420.
28. Osburn, C. L.; Handsel, L. T.; Peierls, B. L.; Paerl, H. W., Predicting sources of dissolved organic nitrogen to an estuary from an agro-urban coastal watershed. *Environmental Science & Technology* **2016,** *50*, (16), 8473-8484.
29. Murphy, K. R.; Hambly, A.; Singh, S.; Henderson, R. K.; Baker, A.; Stuetz, R.; Khan, S. J., Organic matter fluorescence in municipal water recycling schemes: Toward a unified PARAFAC model. *Environmental Science & Technology* **2011,** *45*, (7), 2909-2916.
30. MassBank-consortium and its contributors, MassBank/MassBank-data: Release version 2020.09. In Version 2020.09 ed.; Zenodo, Ed. 2020.
31. Mansouri, K.; Grulke, C. M.; Judson, R. S.; Williams, A. J., OPERA models for predicting physicochemical properties and environmental fate endpoints. *Journal of Cheminformatics* **2018,** *10*, (1), Article Number: 10.
32. Reichert, P., AQUASIM - A tool for simulation and data analysis of aquatic systems. *Water Science and Technology* **1994,** *30*, (2), 21-30.
33. Reichert, P., Design techniques of a computer program for the identification of processes and the simulation of water quality in aquatic systems. *Environmental Software* **1995,** *10*, (3), 199-210.
34. Reichert, P., *AQUASIM 2.0 - User Manual: Computer Program for the Identification and Simulation of Aquatic Systems*. Swiss Federal Institute for Environmental Science and Technology (EAWAG): Dübendorf, Switzerland, 1998.
35. Hondzo, M.; Stefan Heinz, G., Lake water temperature simulation model. *Journal of Hydraulic Engineering* **1993,** *119*, (11), 1251-1273.
36. Wodka, M. C.; Effler, S. W.; Driscoll, C. T.; Field, S. D.; Devan, S. P., Diffusivity-based flux of phosphorus in Onondaga Lake. *Journal of Environmental Engineering* **1983,** *109*, (6), 1403-1415.
37. Matthews, D. A.; O'Donnell, S. M.; Effler, S. W.; Owens, E. M.; Hurteau, C. A.; Prestigiacomo, A. R., Density, salinity, and entry depths of municipal wastewater in an urban lake. *Water Environment Research* **2016,** *88*, (11), 2059-2069.

38. Effler, S. W.; O'Donnell, S. M.; Prestigiacomo, A. R.; O'Donnell, D. M.; Matthews, D. A.; Owens, E. M.; Effler, A. J. P., Tributary plunging in an urban lake (Onondaga Lake): Drivers, signatures, and implications. *Journal of the American Water Resources Association* **2009,** *45*, (5), 1127-1141.
39. Owens, E. M.; Effler, S. W.; Prestigiacomo, A. R.; Matthews, D. A.; O'Donnell, S. M., Observations and modeling of stream plunging in an urban lake. *Journal of the American Water Resources Association* **2012,** *48*, (4), 707-721.
40. Owens, E. M.; Effler, S. W.; O'Donnell, D. M.; Matthews, D. A., Modeling the fate and transport of plunging inflows to Onondaga Lake. *Journal of the American Water Resources Association* **2014,** *50*, (1), 205-218.
41. Doerr, S. M.; Effler, S. W.; Whitehead, K. A.; Auer, M. T.; Perkins, M.; Heidtke, T. M., Chloride model for polluted Onondaga Lake. *Water Research* **1994,** *28*, (4), 849-861.
42. Moriasi, D. N.; Arnold, J. G.; Van Liew, M. W.; Bingner, R. L.; Harmel, R. D.; Veith, T. L., Model evaluation guidelines for systematic quantification of accuracy in watershed simulations. *Transactions of the ASABE* **2007,** *50*, (3), 885-900.
43. Moser, A.; Wemyss, D.; Scheidegger, R.; Fenicia, F.; Honti, M.; Stamm, C., Modelling biocide and herbicide concentrations in catchments of the Rhine basin. *Hydrology and Earth System Sciences* **2018,** *22*, (8), 4229-4249.
44. Schmitt, M.; Wack, K.; Glaser, C.; Wei, R.; Zwiener, C., Separation of photochemical and non-photochemical diurnal in-stream attenuation of micropollutants. *Environmental Science & Technology* **2021,** *55*, (13), 8908-8917.
45. Wang, S.; Green, H. C.; Wilder, M. L.; Du, Q.; Kmush, B. L.; Collins, M. B.; Larsen, D. A.; Zeng, T., High-throughput wastewater analysis for substance use assessment in central New York during the COVID-19 pandemic. *Environmental Science: Processes & Impacts* **2020,** *22*, (11), 2147-2161.
46. Wasswa, J.; Driscoll, C. T.; Zeng, T., Photochemical characterization of surface waters from lakes in the Adirondack Region of New York. *Environmental Science & Technology* **2020,** *54*, (17), 10654-10667.
47. Gueymard, C. A., *SMARTS2, A Simple Model of the Atmospheric Radiative Transfer of Sunshine: Algorithms and Performance Assessment*. Florida Solar Energy Center/University of Central Florida: Cocoa, FL, 1995.
48. Gueymard, C. A., Parameterized transmittance model for direct beam and circumsolar spectral irradiance. *Solar Energy* **2001,** *71*, (5), 325-346.
49. Zepp, R. G.; Cline, D. M., Rates of direct photolysis in aquatic environment. *Environmental Science & Technology* **1977,** *11*, (4), 359-366.
50. Leifer, A., *The Kinetics of Environmental Aquatic Photochemistry: Theory and Practice*. American Chemical Society: Washington, DC, 1988.
51. Apell, J. N.; McNeill, K., Updated and validated solar irradiance reference spectra for estimating environmental photodegradation rates. *Environmental Science: Processes & Impacts* **2019,** *21*, (3), 427-437.
52. Morris, D. P.; Zagarese, H.; Williamson, C. E.; Balseiro, E. G.; Hargreaves, B. R.; Modenutti, B.; Moeller, R.; Queimalinos, C., The attenuation of solar UV radiation in lakes and the role of dissolved organic carbon. *Limnology and Oceanography* **1995,** *40*, (8), 1381-1391.
53. Laszakovits, J. R.; Berg, S. M.; Anderson, B. G.; O'Brien, J. E.; Wammer, K. H.; Sharpless, C. M., *p*-Nitroanisole/pyridine and *p*-nitroacetophenone/pyridine actinometers revisited: Quantum yield in comparison to ferrioxalate. *Environmental Science & Technology Letters* **2017,** *4*, (1), 11-14.
54. Gerecke, A. C.; Canonica, S.; Muller, S. R.; Scharer, M.; Schwarzenbach, R. P., Quantification of dissolved natural organic matter (DOM) mediated phototransformation of phenylurea herbicides in lakes. *Environmental Science & Technology* **2001,** *35*, (19), 3915-3923.
55. Huntscha, S.; Singer, H.; Canonica, S.; Schwarzenbach, R. P.; Fenner, K., Input dynamics and fate in surface water of the herbicide metolachlor and of its highly mobile transformation product metolachlor ESA. *Environmental Science & Technology* **2008,** *42*, (15), 5507-5513.

56. Heeb, F.; Singer, H.; Pernet-Coudrier, B.; Qi, W.; Liu, H.; Longrée, P.; Müller, B.; Berg, M., Organic micropollutants in rivers downstream of the megacity Beijing: Sources and mass fluxes in a large-scale wastewater irrigation system. *Environmental Science & Technology* **2012,** *46*, (16), 8680-8688.
57. Moschet, C.; Götz, C.; Longrée, P.; Hollender, J.; Singer, H., Multi-level approach for the integrated assessment of polar organic micropollutants in an international lake catchment: The example of Lake Constance. *Environmental Science & Technology* **2013,** *47*, (13), 7028-7036.
58. Corsi, S. R.; De Cicco, L. A.; Villeneuve, D. L.; Blackwell, B. R.; Fay, K. A.; Ankley, G. T.; Baldwin, A. K., Prioritizing chemicals of ecological concern in Great Lakes tributaries using high-throughput screening data and adverse outcome pathways. *Science of the Total Environment* **2019,** *686*, 995-1009.
59. Blackwell, B. R.; Ankley, G. T.; Corsi, S. R.; DeCicco, L. A.; Houck, K. A.; Judson, R. S.; Li, S.; Martin, M. T.; Murphy, E.; Schroeder, A. L.; Smith, E. R.; Swintek, J.; Villeneuve, D. L., An "EAR" on environmental surveillance and monitoring: A case study on the use of exposure-activity ratios (EARs) to prioritize sites, chemicals, and bioactivities of concern in Great Lakes waters. *Environmental Science & Technology* **2017,** *51*, (15), 8713-8724.
60. Rose, L. D.; Akob, D. M.; Tuberty, S. R.; Corsi, S. R.; DeCicco, L. A.; Colby, J. D.; Martin, D. J., Use of high-throughput screening results to prioritize chemicals for potential adverse biological effects within a West Virginia watershed. *Science of the Total Environment* **2019,** *677*, 362-372.
61. Bradley, P. M.; Journey, C. A.; Berninger, J. P.; Button, D. T.; Clark, J. M.; Corsi, S. R.; DeCicco, L. A.; Hopkins, K. G.; Huffman, B. J.; Nakagaki, N.; Norman, J. E.; Nowell, L. H.; Qi, S. L.; VanMetre, P. C.; Waite, I. R., Mixed-chemical exposure and predicted effects potential in wadeable southeastern USA streams. *Science of the Total Environment* **2019,** *655*, 70-83.
62. Blackwell, B. R.; Ankley, G. T.; Bradley, P. M.; Houck, K. A.; Makarov, S. S.; Medvedev, A. V.; Swintek, J.; Villeneuve, D. L., Potential toxicity of complex mixtures in surface waters from a nationwide survey of United States streams: Identifying in vitro bioactivities and causative chemicals. *Environmental Science & Technology* **2019,** *53*, (2), 973-983.
63. Bradley, P. M.; Journey, C. A.; Button, D. T.; Carlisle, D. M.; Huffman, B. J.; Qi, S. L.; Romanok, K. M.; Van Metre, P. C., Multi-region assessment of pharmaceutical exposures and predicted effects in USA wadeable urban-gradient streams. *PLoS ONE* **2020,** *15*, (1), e0228214.
64. Schymanski, E. L.; Jeon, J.; Gulde, R.; Fenner, K.; Ruff, M.; Singer, H. P.; Hollender, J., Identifying small molecules via high resolution mass spectrometry: Communicating confidence. *Environmental Science & Technology* **2014,** *48*, (4), 2097-2098.
